# Breakthrough Design of Highly Potent Antivirals Against Wild‐Type HIV‐1 and Lenacapavir‐Resistant M66I Variant

**DOI:** 10.1002/anie.2059566

**Published:** 2026-08-20

**Authors:** Nicolas Jamey, D. M. Nirosh Udayanga, Huanchun Zhang, Roshan Katekar, Wei Xie, Shreya M. Ravichandran, Xinyong Cai, Zachary C. Lorson, William M. McFadden, Won Hee Ryu, Jiashu Xie, Karen A. Kirby, Stefan G. Sarafianos, Zhengqiang Wang

**Affiliations:** ^1^ Center For Drug Design College of Pharmacy University of Minnesota Minneapolis Minnesota USA; ^2^ Center For ViroScience and Cure Laboratory of Biochemical Pharmacology Department of Pediatrics Emory University School of Medicine Children's Healthcare of Atlanta Atlanta Georgia USA

**Keywords:** HIV‐1, capsid, lenacapavir, M66I, resistance

## Abstract

Lenacapavir (LEN), the first‐in‐class HIV‐1 capsid inhibitor (CAI) with picomolar potency and long pharmacokinetics (PK), is approved for both treatment and pre‐exposure prophylaxis (PrEP). However, the frequently selected M66I mutation confers complete resistance to LEN. Potent antivirals against the M66I variant remain elusive. In this work, we have designed LEN analogs featuring a distinctive *R* ^2^ moiety to directly address the molecular mechanism of resistance. Our analog synthesis also entailed developing a new synthetic procedure for *R* ^2^ incorporation. Significantly, the newly designed analogs were highly potent against both WT HIV‐1 and the M66I mutant. Particularly, lead compound **3S** conferred 2.6‐fold higher potency than LEN against WT HIV‐1 and remained highly potent (EC_50_ = 5.8 nM) against the M66I mutant, a drastically advanced activity profile over LEN (EC_50_ > 15 µM). The unprecedented potency of our lead **3S** against M66I was corroborated by a thermal shift assay. In a head‑to‑head intravenous (IV) PK study in rats, lead **3S** demonstrated superior systemic exposure, showing a 4.6‑fold greater plasma area‐under‐curve (AUC) than LEN and largely retaining the long PK characteristic of LEN. These results strongly validate our design and represent a breakthrough in LEN‐based HIV‐1 therapy and prophylaxis.

## Graphical abstract

Lenacapavir, the first‐in‐class drug targeting HIV‐1 capsid, is a breakthrough drug in HIV‐1 treatment and prophylaxis. However, a single M66I mutation confers complete viral resistance to lenacapavir. We report herein the design and synthesis of innovative *R* ^2^ analogs demonstrating low nM potency against HIV‐1 M66I and excellent pharmacokinetics in rats.

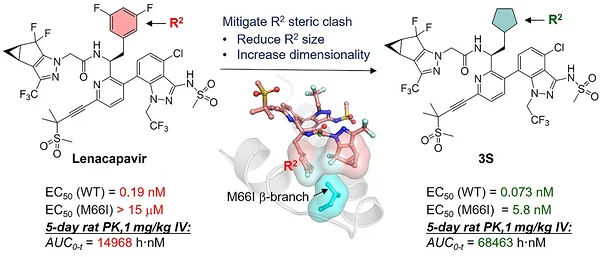

## 1 Introduction

HIV capsid protein (CA) plays a few essential roles in the viral infection cycle: in the early stages, it facilitates cytoplasmic trafficking [1], reverse transcription [2, 3], nuclear import [4], uncoating [5], and integration targeting [6], regulated by various CA‐host interactions [7, 8]; in the late stages, newly produced CA assembles into the viral core consisting of approximately 250 hexamers and exactly 12 pentamers [9, 10], enabled by the protein–protein interactions between CAs [11]. HIV CA as a drug target [12] has been clinically validated by the development [13, 14] and FDA approval of lenacapavir (LEN), a long‐acting injectable (LAI) administered twice a year. Approved as a therapeutic [15] and for pre‐exposure prophylaxis (PrEP) [16], LEN could fundamentally alter the paradigm of HIV clinical management and possibly end the pandemic. However, resistance‐associated mutations (RAMs) have emerged in LEN‐treated populations [17, 18, 19], of which the major single mutations include Q67H (low fold‐resistance, FR), N74D (intermediate FR), and M66I (very high FR) [19].

In particular, the M66I mutation is most prevalent and confers complete resistance to LEN (FR > 80 000, typically no significant antiviral activity at 10 µM) [20, 21, 22]. In addition, LEN does not produce stable binding to CA M66I in biophysical experiments [22], and the LEN‐bound M66I CA structure is unattainable. Surprisingly, although the M66I mutation substantially reduces the replication capacity of HIV, it is still selected in patients alone or in combination with other mutations, whereby the viral fitness is significantly restored [19, 23]. Mitigating the M66I mutation by designing and synthesizing novel LEN analogs is highly coveted, yet second‐generation CA‐targeting drugs with retained potency against the M66I variant remain elusive. In a recent report [20], KFA‐027, an *R* ^2^ defluorinated analog (*R* ^2^ = Ph) of LEN (*R* ^2^ = di‐F Ph), exhibited slightly reduced (threefold) potency against WT HIV and sub‐µM potency against the M66I mutant. Although the potency against M66I is a significant improvement over LEN, the structural modification is minimal, especially considering that F is a well‐known bioisostere for H [24]; and the sub‐µM potency against M66I needs improvement.

LEN‐CA structural and modeling analysis (Figure 1B) [22] showed that while the M66 (Figure 1B, left) in the WT CA interacts comfortably with the di‐F phenyl ring (*R* ^2^, subunit B) of LEN, I66 (Figure 1B, right) is predicted to create significant steric clashes via β‐branching to severely hamper LEN binding. Concurrent with the reported efforts (Figure 1), we have designed and synthesized subunit B/*R* ^2^ analogs where the di‐F phenyl ring of LEN is replaced by a cycloalkyl group (Figure 1C). Structurally, these new analogs feature a non‐flat [25] *R* ^2^ moiety with reduced size, increased saturation [26], dimensionality, and conformational flexibility. Remarkably, with these structural features, the resulting analog **3S** produced overwhelmingly superior antiviral and target‐binding profiles over LEN against the M66I mutant, while also demonstrating 2.6‐fold more potency than LEN against WT HIV‐1. In stark contrast, the diastereomeric **3R** was less active by a few orders of magnitude, confirming the importance of stereochemistry on target binding.

**FIGURE 1:**
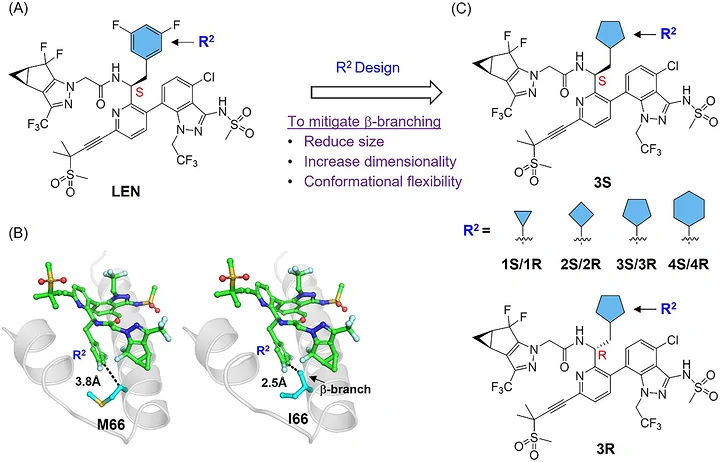
Our design of R^2^ analogs. (A) Structure of LEN; (B) mechanism of M66I resistance: LEN *R* ^2^ interacts with WT CA at an optimal distance (left), whereas β‐branching of I66 is predicted to induce steric hindrance (right) to impede binding. Reproduced from Briganti, L. et al. [22] (C) novel analogs featuring a cycloalkyl *R* ^2^ with reduced size, increased dimensionality, and conformational flexibility as compared to the flat di‐F phenyl of LEN.

## 2 Results and Discussion

### 2.1 Chemical Synthesis

The overall synthetic strategy for our newly designed analogs entails a modular synthesis, combining three subunits named A, B, and C (Figure 2). Subunits A and C are the same as those used for LEN, whereas structural changes (i^2^ group) are pursued within subunit B. The effective synthesis of our analogs requires developing a convenient procedure for subunit B. For subunit A, while reported procedures used a Simmons–Smith cyclopropanation starting with 3‐cyclopentenol [21, 27], the same preparation is not efficient for our scale‐up synthesis and does not allow control of the stereochemistry of the newly formed cyclopropane ring. Thus, a new procedure starting with (*R*)‐epichlorohydrin is used in our synthesis. Subunit C is synthesized based on reported procedures [21].

**FIGURE 2:**
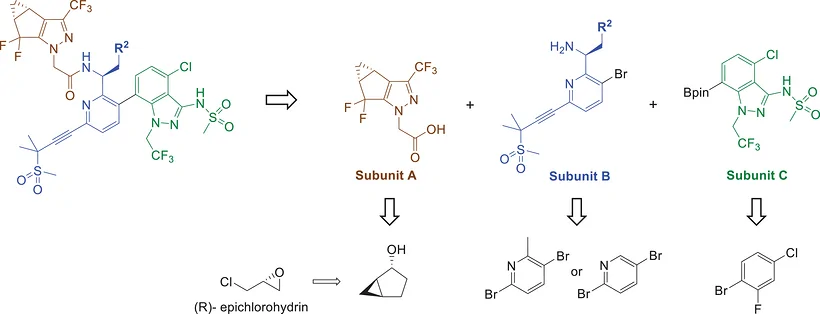
Retrosynthesis of LEN analogs based on a modular synthesis.

Our synthesis of subunit A followed a procedure [28] for all steps except for d, e, and l (Scheme 1). This procedure uses the stereochemistry of the (*R*)‐epichlorohydrin (**5**) to obtain the enantiomerically pure subunit A. In the real synthetic event, (R)‐epichlorohydrin (**5**) reacted with allylmagnesium chloride to give epoxide **7** under basic conditions via alcohol **6**. The subsequent use of lithium 2,2,6,6‐tetramethylpiperidide (LTMP) generated in situ induces an intramolecular cyclopropanation of the terminal epoxide of **7** [29, 30], providing compound **8**. Oxidation of **8** with DMP led to **9**, which was then transformed into **10** using isoamyl nitrite and TMSCl. At this step, the enantiopurity was verified by chiral HPLC, where only one peak was observed on the chromatogram, with a 99% purity (see Supporting Information). The carbonyl was then protected as a dithiane **11**. The oxime group was then reconverted into carbonyl **12**, which was deprotonated to react with ethyl trifluoroacetate (ETFA), affording 1,3‐dione **13**. This 1,3‐dione was converted into salt **14** with diisopropylamine. Then the pyrazole ring on **15** was formed by reacting **14** with hydrazinoacetate hydrochloride. The enantiopurity was once again checked at this step by proton NMR with Eu(tfc)_3_ as a chiral lanthanide shift reagent [31], and as expected, no duplication of signals was observed (see Supporting Information). The difluorination of the dithiane of **15** gave **16**, which upon saponification produced subunit A (Scheme 1).

**SCHEME 1:**
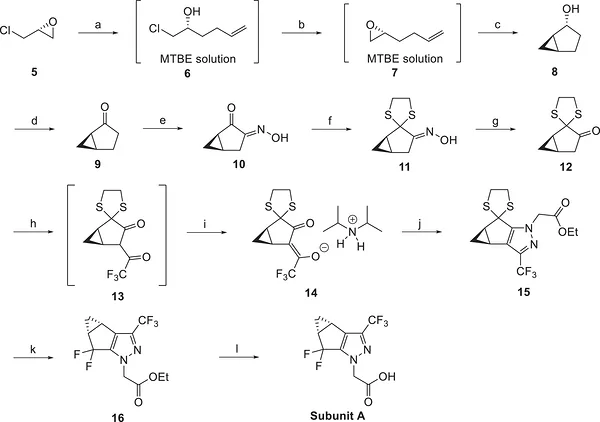
Synthesis of Subunit A^a^. ^a^Reagents and conditions: (a) Allylmagnesium chloride, THF, −30°C, 3 h; (b) KOH, MTBE, 12 h, 47% over 2 steps; (c) n‐BuLi, 2,2,6,6‐TMP, MTBE, 0°C, 4 h, 96%; (d) DMP, DCM, 0°C to rt, 12 h, 90%; (e) isoamyl nitrite, TMSCl, DCM, −30°C, 4 h, 27%; (f) 1,2‐Ethanedithiol, pTSOH, HOAc, rt, overnight, 82%; (g) pTSOH, 2‐butanone, water, 75°C, overnight, 89%; (h) LiHMDS, ETFA, THF, −10°C, 4 h; (i) DIPA, rt, 66% over 2 steps; (j) Ethyl hydrazinoacetate hydrochloride, LiCl, CH_3_COCl, EtOH, 55°C, 18 h, 78%; (k) DBDMH, HF‐Pyridine, DCM, −15°C, 80%; (l) LiOH, THF/MeOH, rt, 1 h, 95%.

The synthesis of subunit B invariably entailed advanced intermediates **22**. For intermediate **22c** featuring a cyclopentyl R^2^ group, the synthesis commenced with commercially available 3,6‐dibromo‐2‐methylpyridine **17** (Scheme 2A) following a reported procedure [13, 21, 32]. Dibromination of the methyl group on the pyridine ring with *N*‐bromosuccinimide and azobisisobutyronitrile furnished intermediate **18**, which, upon hydrolysis, yielded aldehyde **19**. Condensation of aldehyde **19** with (S)‐(‐)‐tert‐butanesulfinamide afforded N‐sulfinyl imine **20**. Diastereoselective nucleophilic addition [30] of cyclopentylmethylmagnesium bromide **21** to imine **20** provided two separable diastereomers (**22cS**/**22cR**).

**SCHEME 2:**
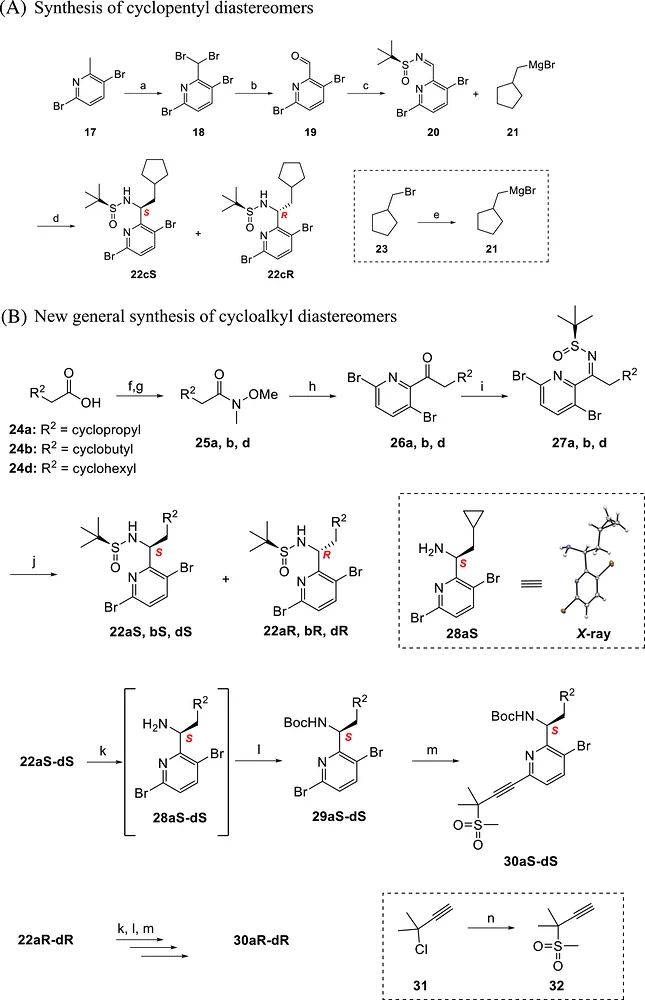
Synthesis of Cyclopentyl Subunit B. (a) Synthesis of cyclopentyl diastereomers. (b) New general synthesis of cycloalkyl diastereomers. ^a^Reagents and conditions: (a) NBS, AIBN, CCl_4_, 80°C, 12 h; (b) AgNO_3_, EtOH, H_2_O, reflux, 5 h, 49% (two steps); (c) (*S*)‐(‐)‐tert‐Butanesulfinamide, Cs_2_CO_3_, DCM, rt, 1 h, 77%; (d) THF, −78°C, 2 h; (e) Mg, I_2_, Et_2_O, rt to 35°C, 1.5 h; (f) SOCl_2_, DMF, DCM, rt, 2 h; (g) MeNHOMe.HCl, Et_3_N, DCM, 0°C to rt, 1 h, 70%–85% (two steps); (h) 2,5‐dibromopyridine, TMPMgCl.LiCl, THF, −20°C, 2 h, 70%–81%; (i) (*S*)‐(‐)‐tert‐butanesulfinamide, Ti(OEt)_4_, THF, 70°C, 24 h, 47%–50%; (j) NaBH_4_, THF, 0°C to rt, 12 h; (k) 4 M HCl (in dioxane), MeOH, rt, 3 h; (l) Boc_2_O, Et_3_N, DCM, rt, 2 h, 93%–96% (two steps); (m) **32**, Pd(PPh_3_)_2_Cl_2_, CuI, Et_3_N, DMF, rt, 12 h, 50%–82%; (n) sodium methanesulfinate, Cu(OAc)_2_, TMEDA, iPrOAc, 20°C–40°C, 12 h, 58%.

However, synthesis of the cyclopropyl intermediate **22aS** via this reported procedure was unsuccessful due to cyclopropyl ring opening during the Grignard formation [33, 34]. Consequently, we have worked out an alternative synthetic route for intermediates **22** (Scheme 2B) [35, 36, 37], which can be applied to the synthesis of all cycloalkyl analogs. The synthesis began with commercially available carboxylic acids **24a, b, d**, which were converted to the corresponding Weinreb amides **25a, b, d**. Subsequent treatment of Weinreb amides with 2,5‐dibromopyridine in the presence of TMPMgCl.LiCl afforded ketones **26a, b, d**. Condensation of ketones with Ellman's auxiliary in the presence of titanium(IV) ethoxide furnished *N*‐sulfinyl imines **27a, b, d**. Diastereoselective reduction of the imine with sodium borohydride [38, 39] in THF provided diastereomerically pure **22aS, bS, dS** and **22aR, bR, dR**, after separation.

The two methods converged in the subsequent steps. From **22aS**−**dS**, removal of the chiral auxiliary under acidic conditions produced amines **28aS**−**dS**, which were Boc‐protected to afford **29aS**−**dS**. Finally, Sonogashira coupling with the methylsulfone‐bearing alkyne **32** delivered the target subunits B **30aS**−**dS**. Following the same synthetic route, **30aR**−**dR** were obtained starting from **28aR**−**dR**. Importantly, single‐crystal x‐ray crystallographic analysis of amine **28aS** (Scheme 2b) confirmed the S configuration at the indicated stereogenic center (see Supporting Information).

Subunit C was prepared using a reported procedure (Scheme 3) [21]. The synthesis started with the formylation of 1‐bromo‐4‐chloro‐2‐fluorobenzene (**33**) to give aldehyde **34**. After the aldehyde was converted into nitrile **35**, a regioselective cyclization with hydrazine hydrate afforded 3‐aminoindazole **36**. The subsequent alkylation of N‐1 produced compound **37**, which, upon palladium‐catalyzed borylation, delivered subunit C.

**SCHEME 3:**
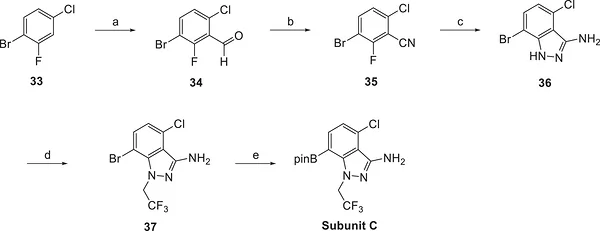
Synthesis of subunit C^a^. ^a^Reagents and conditions: (a) LDA, DMF, THF, −78*°*C, 99%; (b) NH_2_OSO_3_H, H_2_O, 50°C, overnight, 80%; (c) H_2_NNH_2_•H_2_O, EtOH, 80*°*C, overnight, 27%; (d) 2,2,2‐Trifluoroethyl trifluoromethanesulfonate, Cs_2_CO_3_, DMF, 65°C, overnight, 56%; (e) B_2_pin_2_, Pd(PPh_3_)_2_Cl_2_, KOAc, 1,4‐dioxane, 110*°*C, 70%.

The assembly of subunits for the synthesis of our analogs largely followed the procedure used for LEN synthesis (Scheme 4) [21]. For the synthesis of the desired (*S*)‐ isomers, Suzuki coupling between subunits B (**30aS**−**dS**) and C afforded intermediates **38aS**−**dS**, which were converted into the bis‐sulfonamides **39aS**−**dS** upon treatment with methanesulfonyl chloride. From these steps, the presence of two atropoisomers was observed during purification and NMR analysis. Bis‐sulfonamides **39aS**−**dS** were treated with TFA for Boc deprotection, which also removed one methane sulfonyl group, and the resulting amines were directly condensed with the acid of subunit A to give final compounds **1S–4S**. All these steps were also repeated on the *(R)*‐enantiomers of subunit B (**30aR**−**dR**) to afford stereoisomeric final compounds **1R–4R**. Similar to LEN, each final compound exists as a mixture of two atropoisomers as described below.

**SCHEME 4:**
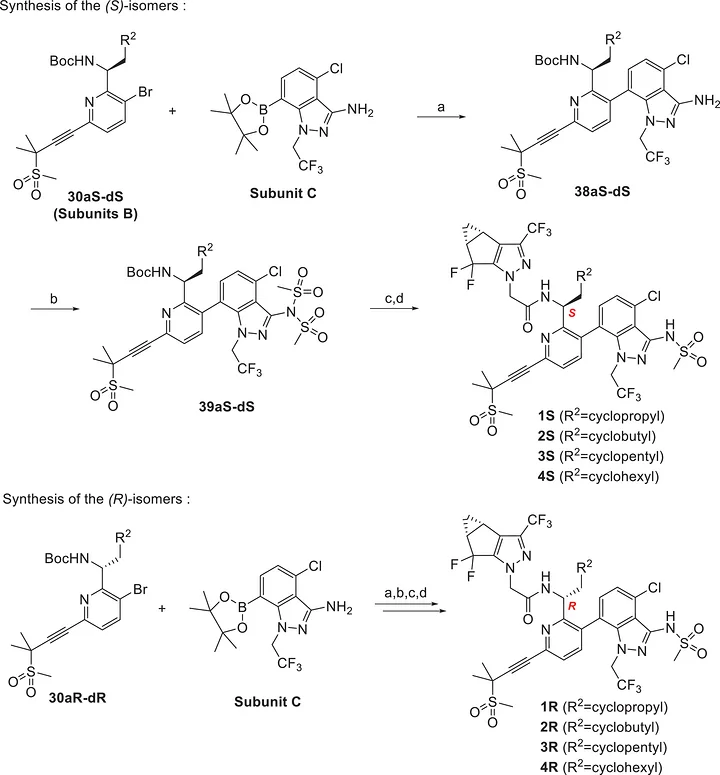
Synthesis of the LEN analogs via subunits B, C, and A^a^. *^a^* Reagents and conditions, all yields with R^2^ as cyclopentyl: (a) Pd(dppf)Cl_2_, K_2_CO_3_, 1,4‐dioxane/H_2_O, 80*°*C, 16 h, 67%; (b) methane sulfonylchloride, Et_3_N, DCM, rt, 2 h, 58%; (c) TFA, DCM, rt, 2 h, 99%; (d) Subunit A, HATU, DIPEA, DMF, rt, overnight, 37%.

The two atropoisomers are clearly discernible on HPLC as shown with compound **3S** (Figure 3, top), with an atropisomeric ratio of 1:3.8 favoring fraction 2 (Figure 3). Separation of the two fractions using preparative HPLC yielded a minor atropisomer at 10.784 min and a major one at 11.781 min. However, interconversion was observed with both atropoisomers overnight post separation, per NMR analysis (Figure 3, bottom). Specifically, the separated major atropoisomer (fraction 2) reached an atropisomeric ratio of 9:1 on ^1^H NMR, and the minor fraction reached a ratio of 6:4, both favoring the major atropisomer (right peak on HPLC and left peak on ^1^H NMR, Figure 3). These analyses are consistent with LEN [21], which indicates that the atropoisomers are separable only transiently, and interconversion occurs within hours. The assignment of **3S.2** as the major atropisomer is based on LEN atropisomerism [13, 21] and the predicted similar binding poses of **3S** and LEN in WT CA (Figure 4C,D).

**FIGURE 3:**
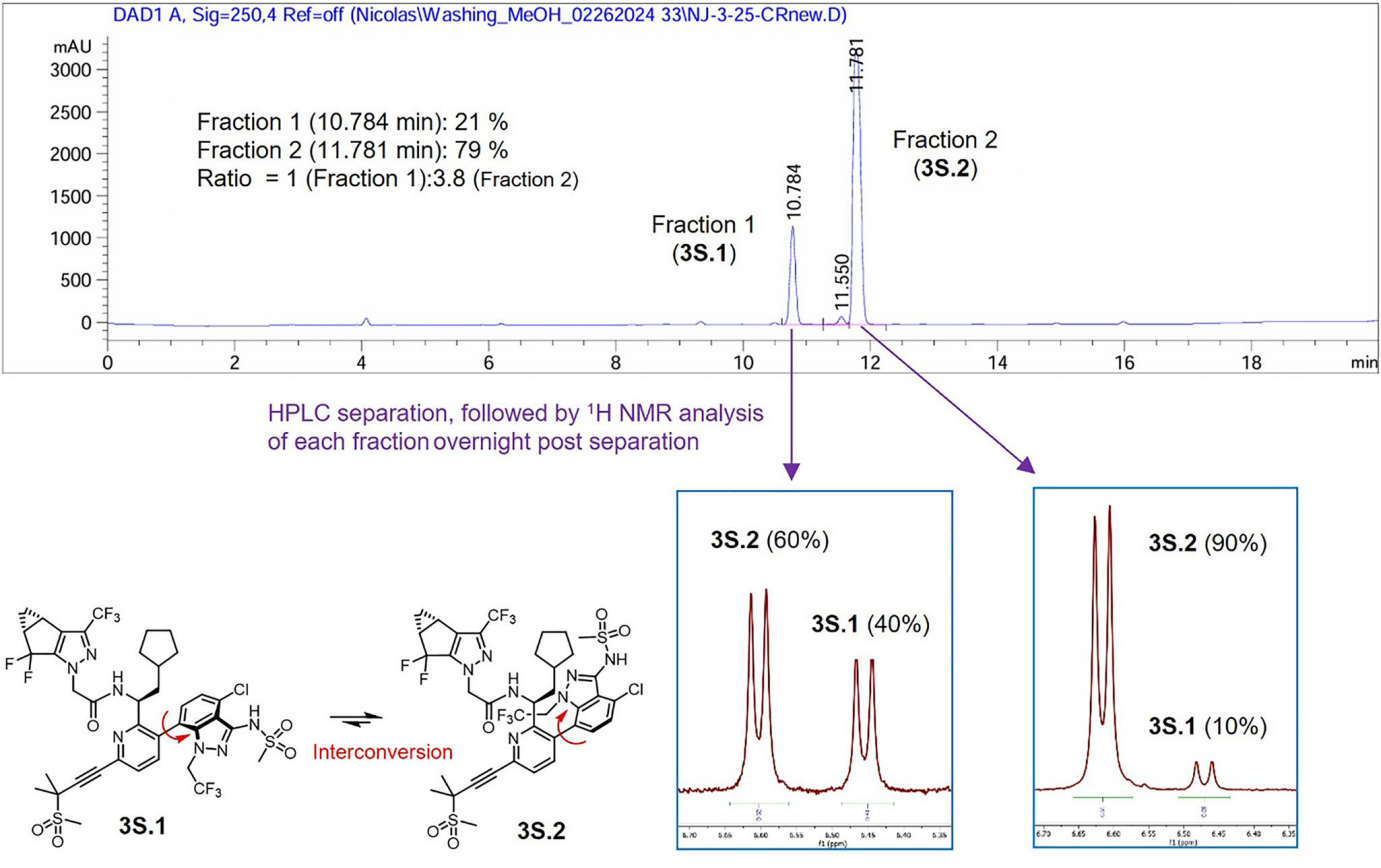
Atropisomerism of compound **3S**. HPLC chromatogram of **3S** (top) showed fractions of the two atropisomers. ^1^H NMR spectra (bottom) of the two HPLC‐separated fractions indicated interconversion overnight post separation. The right peak of HPLC (fraction 2) and the left peak of ^1^H NMR likely correspond to the energetically more favored atropisomer **3S.2**, per the assignment of LEN.

**FIGURE 4:**
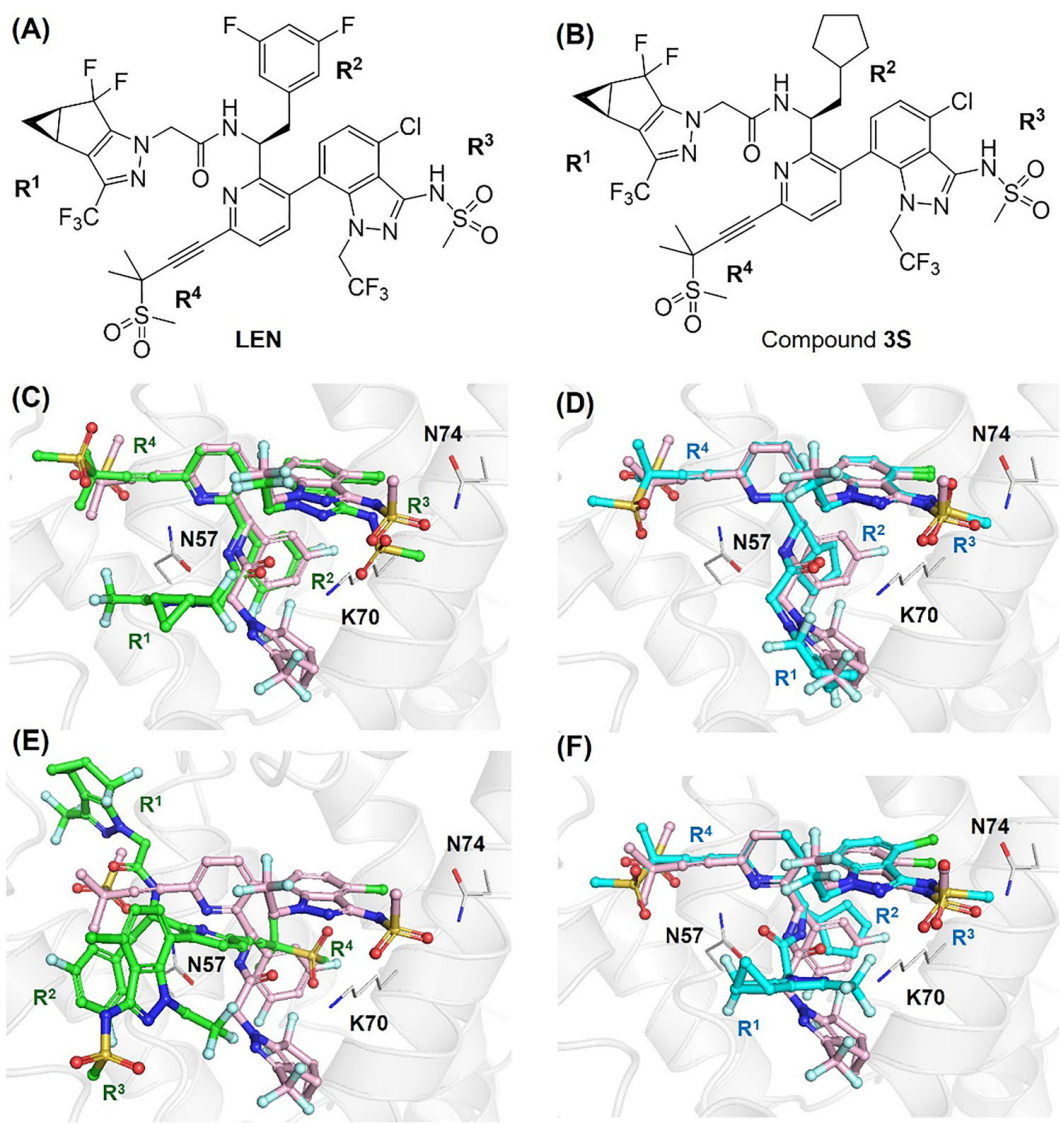
Induced‐fit docking (IFD) poses of LEN and compound **3S** to a WT and M66I CA (PDB 4XFZ) structural model. IFD poses of LEN are shown in green stick‐and‐ball format; IFD poses of compound **3S** are shown in cyan; the reference experimental bound structure of LEN (PDB 6V2F) is shown in pink, and key residues are shown in gray line. The proteins are shown in transparent gray cartoons. (A) Structure of LEN. (B) Structure of compound **3S**. (C) IFD pose of LEN on WT CA. (D) IFD pose of analog **3S** on WT CA. (E) IFD pose of LEN on M66I CA. (F) IFD pose of compound **3S** on M66I CA.

### 2.2 Antiviral Activity Against HIV‐1

New *R* ^2^ analogs were tested for antiviral activity against both WT and mutant M66I HIV‐1 viruses, and also for cytotoxicity, in TZM‐GFP reporter cells. LEN was used as both the positive control and the benchmark. Interestingly, of the eight analogs synthesized, the four analogs (**1S**, **2S**, **3S**, **4S**) conforming to the stereochemistry of LEN, all conferred sub to very low nM potencies against WT HIV‐1 (Table 1), suggesting that a cycloalkyl group at *R* ^2^ is tolerated. Specifically, in our assay analog **1S** (EC_50_ = 0.9 nM), **2S** (EC_50_ = 1.0 nM), and **4S** (EC_50_ = 0.70 nM) were moderately less potent than LEN (EC_50_ = 0.19 nM), whereas analog **3S** (EC_50_ = 0.073 nM) produced a potency 2.6‐fold higher than LEN. Against the M66I mutant, LEN did not confer significant potency at concentrations up to 15 µM in our assay (Table 1), whereas our analog **4S** (EC_50_ = 203 nM) was active in the sub‐µM range comparable to the reported potency for KFA‐027 (EC_50_ = 444 nM, Figure 1E). Most strikingly, our analogs **1S** (EC_50_ = 15.7 nM), **2S** (EC_50_ = 40 nM), and **3S** (EC_50_ = 5.8 nM) conferred unprecedented low nM EC_50_ values against M66I (Table 1), decisively more potent than KFA‐027^22^ (EC_50_ = 444 nM). The overall superior antiviral profile of our new analogs suggests that the increased *sp* ^3^ character [25] and conformational flexibility [40] with cycloalkyls largely mitigate the steric clashes while still maintaining excellent target interactions. Our work so far has also revealed two important structure–activity relationship (SAR) trends: (1) the stereochemistry conforming to LEN is required as the *R* stereoisomers (**1R**, **2R**, **3R**, **4R**) produced markedly weaker potencies against WT HIV and marginal activities against M66I; (2) analogs with a smaller cyclopropyl (**1S**), cyclobutyl (**2S**), or bigger cyclohexyl (**4S**) are weaker than our lead cyclopentyl analog **3S**, suggesting that a 5‐membered carbocycle may be the optimal size for *R* ^2^. Collectively, these results strongly validate our analog design.

**TABLE 1:** Antiviral potency against WT HIV‐1 and the M66I mutant.

Compound	EC_50_ ^a^		FR^d^	CC_50_ (µM) ^a^
	WT HIV‐1 (nM)	M66I HIV‐1 (nM)		
1S	0.9 ± 0.4	15.7 ± 12.0	18	>100
1R	3.8 ± 0.5	2300 ± 200	605	>100
2S	1.0 ± 0.2	40 ± 7	40	>100
2R	63 ± 9	2300 ± 200	37	>100
3S	0.073 ± 0.03	5.8 ± 4.2	79	>100
3R	12.3 ± 3.5	4000 ± 90	325	>100
4S	0.70 ± 0.07	203 ± 49	290	>100
4R	70 ± 9	>40 000	570	>100
LEN	0.19 ± 0.05^b^	>15 000 ^c^	>78 000	>100

^a^ In TZM‐GFP cells.

^b^ Published EC_50_ = 0.13 nM.

^c^ Published EC_50_ >10 µM.

^d^ Fold Resistance, defined as EC_50 (M66I)_ /EC_50 (WT)._

### 2.3 Thermal Shift Assay

To further characterize these new analogs, we assessed their effect on the stability of the target protein in a thermal shift assay (TSA) [41]. Capsid‐targeting inhibitors are known to bind to CA hexamers but not CA monomers. In this assay, WT and M66I crosslinked CA hexamers were used, and a larger ΔTm indicates higher target stabilization, which correlates with binding. The thermal shift data as shown in Table 2 largely corroborate the observed antiviral potency and SAR trends: 1) in the TSA the four *R* stereoisomers **1R**, **2R**, **3R**, and **4R** did not affect at all either of the tested CA hexamers (Table 2); 2) analogs **1S**, **2S**, **3S**, and to a lesser degree **4S**, showed high stabilization of M66I CA hexamer whereas LEN did not; 3) compound **3S** produced an overall stabilization profile superior to that of **1S**, **2S**, and **4S**.

**TABLE 2:** Thermal shift assay (TSA) measuring the effect of compound on the stability of WT and M66I HIV‐1 cross‐linked CA hexamers.

Compound	WT ΔTm (°C)	M66I ΔTm (°C)
1S	12.3 ± 0.1	9.5 ± 0.1
1R	0.07 ± 0.09	−0.04 ± 0.07
2S	12.7 ± 0.2	9.4 ± 0.0
2R	−0.07 ± 0.1	0.0 ± 0.0
3S	14.1 ± 0.1	10.0 ± 0.2
3R	0.0 ± 0.09	0.04 ± 0.07
4S	12.2 ± 0.1	7.0 ± 0.1
4R	0.07 ± 0.1	0.07 ± 0.1
LEN	16.1 ± 0.4	−1.1 ± 0.4

### 2.4 Molecular Modeling

To provide computational insights into the difference in activity between LEN and the new analog **3S**, we performed induced‐fit docking (IFD) calculations [42], generating WT and M66I HIV‐1 CA structural models. The starting model for both was 4XFZ, a crystal structure of the WT native full‐length CA hexamer in complex with PF74 [11]. The existing WT 4XFZ structure was prepared for molecular docking by performing the protein preparation workflow in Schrödinger as described in the Materials and Methods section in the Supporting Information. The M66I CA structural model was obtained by taking the aforementioned prepared 4XFZ structure, performing the M66I mutation on the CA, and performing another round of the protein preparation workflow. 4XFZ was used as the reference structure, opposed to 6V2F [21], of which the cognate ligand is LEN, as the 6V2F structure, when used as a receptor for molecular docking, could not distinguish and explain the difference in activity between LEN and compound **3S**.

IFD poses of LEN and compound **3S** on the WT CA model show little deviation from the 6V2F crystal structure, maintaining the key protein‐ligand interactions as shown in Figure 4C,D. The only difference in pose occurs in the orientation of subunit A (*R* ^1^). The conformational flexibility of subunit A is observed in both rigid docking, induced‐fit docking, and molecular dynamics simulation (results not shown), resulting in various poses with different orientations for subunit A but not for other subunits. At other orientations, ring flipped or in the opposite dihedral direction, subunit A either maintains pi–cation interaction with K70 or forms interactions with the adjacent R173 (not shown).

IFD on the M66I CA model, however, produces drastically different results. LEN does not dock in the generated M66I CA model with the protein–ligand pose and interactions observed in 6V2F, but rather in a completely misaligned pose, resulting in the loss of key interactions (Figure 4E). On the other hand, compound **3S** docks to M66I CA similarly to the observed position in 6V2F, as shown in Figure 4F. Despite the slight difference in how subunit B (*R* ^2^) sits in the binding site, compound **3S** maintains the canonical protein–ligand interaction observed in 6V2F. In the absence of the LEN‐bound structure of CA M66I, the IFD calculations using the 4XFZ structure provide a qualitative explanation for the difference in activity between LEN and compound **3S**.

### 2.5 ADME Properties

The ADME properties of our analogs were evaluated in parallel with LEN. As summarized in Table 3, all analogs exhibited profiles closely aligned with LEN across aqueous solubility, plasma stability, microsomal stability, and PAMPA permeability. Each analog showed excellent stability in both human and rat plasma, with greater than 90% of the parent compound remaining after 24 h of incubation, consistent with the high plasma stability observed for LEN. Similarly, metabolic stability in human and rat liver microsomes revealed prolonged half‐lives exceeding 120 min, indicative of low intrinsic hepatic clearance. As expected, all analogs displayed extremely low aqueous solubility at pH 7.2 and poor permeability in PAMPA. Additionally, plasma protein binding for analog **3S** and LEN was compared in both species (human and rats), and each showed very high binding (>99.9%). These properties, particularly the low solubility and exceptional metabolic stability, mirror the physicochemical features underlying LEN's long‐acting PK profile, in which the compound precipitates to form a slow‑release depot upon SC administration, and therefore represent desirable attributes for further development.

**TABLE 3:** Solubility, permeability, and in vitro metabolic stabilities of selected compounds.

Compd	Aqueous solubility (µmol/L)^a^, *n = 3*	Plasma stability *t*_1/2_ (h), *n = 3*		Microsomal stability^b^ *t*_1/2_ (min), *n = 3*		PAMPA permeability Pe^c^ (10^−6^ cm/s), *n* = 4
		Human	Rat	Human	Rat	
**LEN**	<0.009^d^	> 24^e^	> 24^e^	>120^f^	>120^f^	<0.02^g^
**1S**	0.019 ± 0.005	> 24^e^	> 24^e^	>120^f^	>120^f^	<0.02^g^
**2S**	<0.009^d^	> 24^e^	> 24^e^	>120^f^	>120^f^	<0.02^g^
**3S**	<0.009^d^	> 24^e^	> 24^e^	>120^f^	>120^f^	<0.02^g^
**4S**	<0.009^d^	> 24^e^	> 24^e^	>120^f^	>120^f^	<0.02^g^
**Verapamil**				7.5 ± 0.3	4.34 ± 0.03	

*Note*: Data is presented as mean ± SD.

^a^ Aqueous solubility was determined in Dulbecco's Phosphate‐Buffered Saline (DPBS, pH 7.2).

^b^ CYP enzyme cofactor: NADPH.

^c^ Pe: The apparent permeability coefficient. High Permeability: >1.5 × 10^−6^ cm/s; Low Permeability: < 1.5× 10^−6^ cm/s.

^d^ The measured solubility of the compound was below the lower limit of quantitation (LLOQ); therefore, values are denoted as “< lower limit estimate”.

^e^ Remaining percentage was observed to be greater than 90% at the end of incubation (24 h).

^f^ Remaining percentage was observed to be greater than 90% at the end of incubation (60 min).

^g^ The measured concentration of the compound in receiver was below the lower limit of quantitation (LLOQ); therefore, permeability is determined by replacing receiver value with LLOQ and denoted as “< lower limit estimate”.

### 2.6 PK in Rats

In a subsequent head‐to‐head PK study in rats (Figure 5 and Table 4), analog **3S** exhibited a markedly improved systemic exposure compared with LEN, as evidenced by persistently higher plasma concentrations over 5 days and a 4.6‐fold higher AUC_0‐t_ following the same single intravenous dosing at 1 mg/kg. Correspondingly, analog **3S** showed a substantially lower volume of distribution (*V*_d_) than LEN (0.5 L/kg vs. 4 L/kg), suggesting that it remains predominantly within the vascular compartment and supports a more controlled and plasma focused disposition. Reduced tissue sequestration may enhance target engagement while limiting off‐target accumulation. Furthermore, analog **3S** displayed slower clearance than LEN (0.016 L/h/kg vs. 0.06 L/h/kg). This extremely low clearance (less than 0.5% of hepatic blood flow in rats) indicates high in vivo stability and contributes to a long terminal half‑life of 22 h, consistent with a long‑acting profile. Taken together, the superior plasma exposure of analog **3S** and its favorable clearance to *V*_d_ relationship suggest more predictable pharmacokinetics and the potential for improved safety and dose efficiency.

**FIGURE 5:**
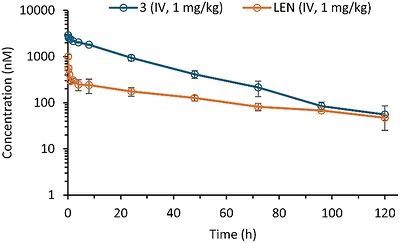
Mean plasma concentration–time profile of LEN and compound **3S** in male Wistar Hannover rats following a single IV dose (1 mg/kg).

**TABLE 4:** PK parameters of LEN and compound **3S** in male Wistar Hannover rats following a single IV dose (1 mg/kg).

PK parameters	LEN (IV, 1 mg/kg)	3S (IV, 1 mg/kg)
*t*_1/2_ (h)	51 ± 8	22 ± 3
AUC_0‐t_ (h.nM)	14968 ± 2343	68463 ± 8655
AUC_0‐∞_ (h.nM)	18452 ± 1935	70357 ± 9925
MRT_0‐t_ (h)	42 ± 3	26 ± 3
*V*_d_ (L/kg)	4.2 ± 0.9	0.49 ± 0.03
Cl (L/h/kg)	0.06 ± 0.01	0.016 ± 0.002

*Note*: Data are presented as mean ± SD (*n* = 4). *t*_1/2_, terminal half‐life. AUC_0‐t_, area under the concentration–time curve from the time of dosing to the last measurable time point. AUC_0‐∞_, area under the concentration–time curve from the time of dosing to infinity. MRT_0‐t_, mean residence time from time zero to the last measurable concentration. *V*_d_, volume of distribution. Cl, clearance.

## 3 Conclusion

In summary, to mitigate the high‐level resistance to LEN conferred by the M66I mutation, we have designed and synthesized LEN R^2^ analogs showing unprecedented low nM potency against the M66I mutant, as exemplified by analog **3S** (EC_50_ = 5.8 nM). In the thermal shift assay, our analogs produced large shifts against the M66I CA hexamer, whereas the shift with LEN was marginal, indicating high binding affinity of our analogs and corroborating the exceptional antiviral potency observed. Our analog design features cycloalkyl *R* ^2^ groups distinctive from LEN or its known analogs. Lead **3S** also demonstrated a substantially improved plasma exposure profile over LEN in the rat PK studies. Overall, the results presented here validate our design and represent a major advancement in LEN‐based HIV therapy and prophylaxis.

## Author Contributions

**Nicolas Jamey**: writing – review and editing, writing – original draft, methodology, investigation, data curation, validation, formal analysis. **D. M. Nirosh Udayanga**: writing – review and editing – original draft, methodology, investigation, data curation, validation, formal analysis. **Roshan Katekar**: methodology, data curation, investigation, validation, writing – original draft, formal analysis. **Won Hee Ryu**: methodology, software, data curation, investigation, validation, visualization, writing – original draft. **Huanchun Zhang**: validation, methodology, data curation, investigation. **Wei Xie**: data curation, methodology, investigation. **Jiashu Xie**: methodology, data curation, investigation, supervision, formal analysis, writing – original draft, validation, resources. **Zhengqiang Wang**: conceptualization, investigation, formal analysis, supervision, funding acquisition, project administration, resources, writing – original draft, writing – review and editing, methodology. **Karen A. Kirby**: methodology, data curation, investigation, validation, formal analysis, supervision, writing – review and editing. **Shreya M. Ravichandran**: data curation, investigation. **Zachary C. Lorson**: data curation, investigation. **William M. Mcfadden**: data curation, investigation. **Xinyong Cai**: data curation, investigation. **Stefan G. Sarafianos**: conceptualization, formal analysis, supervision, funding acquisition, project administration, resources, writing – review and editing, investigation.

## Conflicts of Interest

The authors declare no conflicts of interest.

## Supporting information




**Supporting File 1**: anie74253‐sup‐0001‐SuppMat.pdf.

**Supporting File 2**: anie74253‐sup‐0002‐SuppMat.zip.

## Data Availability

All data associated with this study are available in the published article and its Supporting Information: materials and methods, experimental details; X‐ray crystallography data (raw data accessible from CCDC: Deposition Number 2537357); ^1^H NMR, ^13^C NMR and ^19^F NMR spectra, HPLC traces; PDB coordinates for induced fit docking: 6V2F_reference, IFD_4XFZ_WT_LEN, IFD_4XFZ_WT_Compound **3S**, IFD_4XFZ_M66I_LEN, IFD_4XFZ_M66I_Compound **3S**.
