# Supplementary material for: Breakthrough Design of Highly Potent Antivirals Against Wild‐Type HIV‐1 and Lenacapavir‐Resistant M66I Variant
Source: Angew Chem Int Ed Engl. 2026 Aug 20;65(46):e2059566. doi: 10.1002/anie.2059566 (PMC13749331; doi:10.1002/anie.2059566)
Supplement: Supplementary file 1 [file ANIE-65-e2059566-s002.pdf]

# Supporting Information

## Breakthrough design of highly potent antivirals against wildtype HIV-1 and lenacapavir-resistant M66I variant

Nicolas Jamey[1Δ], D.M. Nirosh Udayanga[1Δ], Huanchun Zhang[2], Roshan Katekar[1], Wei Xie[1], Shreya M. Ravichandran[2], Xinyong Cai[2], Zachary C. Lorson[2], William M. McFadden[2], Won Hee Ryu[1], Jiashu Xie[1], Karen A. Kirby[2], Stefan G. Sarafianos[2*], Zhengqiang Wang[1*]

[1] Center for Drug Design, College of Pharmacy, University of Minnesota, Minneapolis, MN 55455, USA

[2] Center for ViroScience and Cure, Laboratory of Biochemical Pharmacology, Department of Pediatrics, Emory University School of Medicine, Children's Healthcare of Atlanta, Atlanta, GA 30322, USA

[Δ]These authors contributed equally

*Correspondence: Stefan Sarafianos, stefanos.sarafianos@emory.edu; Zhengqiang Wang, wangx472@umn.edu.

# Table of contents


| | |
|---|---|
| Experimental Details | Pages S3–S58 |
| References | Pages S59–S61 |
| X-ray Crystallography Data | Pages S62–S68 |
| $^{1}$H, $^{13}$C, $^{19}$F NMR Spectra and HPLC Traces | Pages S69–S141 |

# Experimental details

## Chemistry general

**Experimental:** All reactions were set up under argon atmosphere utilizing glassware that was flame-dried and cooled under a vacuum. All non-aqueous manipulations were using standard Schlenk techniques. Reactions were monitored using thin-layer chromatography (TLC) on silica gel plates. Visualization of the developed plates was performed under UV light (254 nm) or $KMnO_4$ stain. Flash column chromatography was performed on SYNTHWARE 40-63 μm silica gel.

**Materials:** Unless otherwise indicated, catalysts and materials were obtained from Sigma Aldrich, Oakwood, Ambeed, Strem, or Acros Co. Ltd. Moreover, commercially available reagents were used without additional purification.

**Instrumentation:** NMR spectra were recorded at 400 MHz ($^1$H NMR) and 100 MHz ($^{13}$C NMR) using TMS as an internal standard. Chemical shifts are given relative to TMS or $CDCl_3$ (0 ppm for $^1$H NMR, 77.16 ppm for $^{13}$C NMR). Data are represented as follows: chemical shift (multiplicity, coupling constant (s) in Hz, integration). Multiplicities are denoted as follows: br = broad, s = singlet, d = doublet, t = triplet, q = quartet, m = multiplet. Mass spectroscopy data of the products were collected on an HRMS-TOF instrument using APCI and ESI ionization.

# 1. Synthesis of LEN Analogs

## 1.1 Synthesis of subunit A

This synthesis was mainly inspired by an already described procedure, especially for the preparation of **8**.[1]

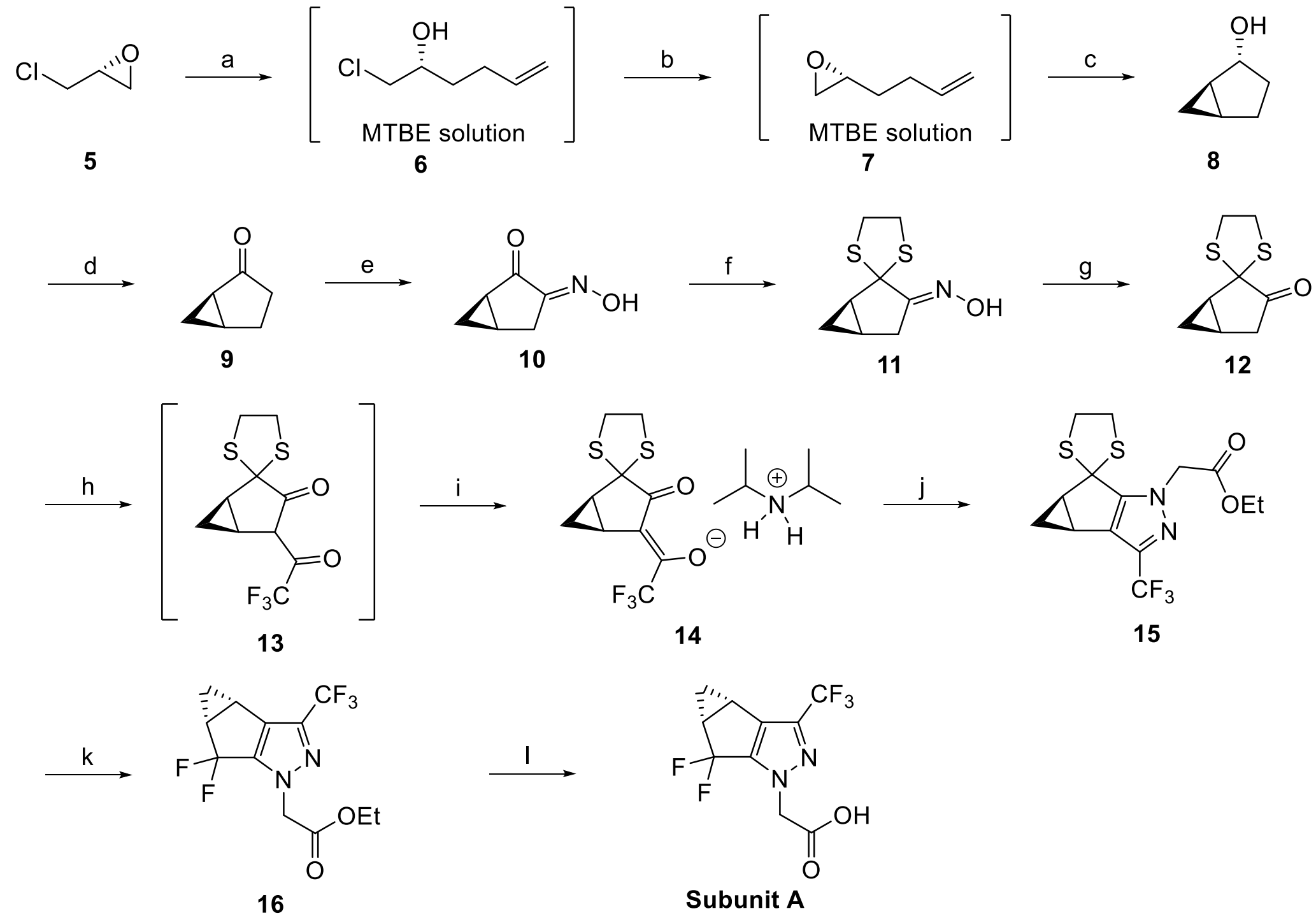


$^{a}$Reagents and conditions: (a) Allylmagnesium chloride, THF, -30 °C, 3h; (b) KOH, MTBE, 12h, 47% over 2 steps; (c) *n*-BuLi, 2,2,6,6-TMP, MTBE, 0 °C, 4h, 96%; (d) DMP, DCM, 0 °C to rt, 12h, 90%; (e) isoamyl nitrite, TMSCl, DCM, -30 °C, 4h, 27%; (f) 1,2-Ethanedithiol, pTSOH, HOAc, rt, overnight, 82%; (g) , pTSOH, 2-butanone, water, 75°C, overnight, 89%; (h) LiHMDS, ETFA, THF, -10 °C, 4h; (i) DIPA, rt, 66% over 2 steps; (j) Ethyl hydrazinoacetate hydrochloride, LiCl, $CH_3COCl$, EtOH, 55 °C, 18h, 78%; (k) DBDMH, HF-Pyridine, DCM, -15 °C, 80%; (l) LiOH, THF/MeOH, rt, 1h, 95%.

**(R)-2-(but-3-en-1-yl)oxirane (7)**

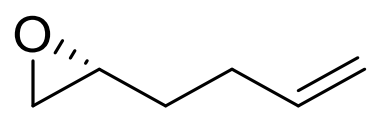

42.0 g (428 mmol)of (R)-epichlorohydrin and 150 mL (3.5 L/kg) of anhydrous THF were charged into a 1L round-bottom flask under a $N_2$ atmosphere and then the contents were adjusted to −30 °C. 235 mL (471 mmol, 1.1 eq) of 2M allyl magnesium chloride was added slowly, maintaining temperature at −30 °C. After 3 hours, the reaction mixture was quenched with a 1M HCl solution (1.5 eq). The cooling bath was removed, and the reaction mixture was stirred for 30 min reaching room temperature. The organic layer was separated and washed with a $NaHCO_3$ solution. The THF was then evaporated, and 400 mL of MTBE was added, with 200 mL of water. The MTBE layer was separated, and this MTBE solution of **6** was cooled down to 0°C and was charged with a solution of KOH (38.4 g, 684 mmol, 1.6 eq). The ice bath was removed to let it warm up to room temperature, and the reaction was stirred overnight. The MTBE organic layer was separated and concentrated, before being distilled under atmospheric pression, to obtain 21 g of the epoxide **7** (47% over 2 steps). **$^1$H-NMR** (400 MHz, $CDCl_3$) δ ppm: 5.83 (ddt, *J* = 16.9, 10.1, 6.6 Hz, 1H), 5.05 (dq, *J* = 17.1, 1.7 Hz, 1H), 4.98 (dq, *J* = 10.2, 1.5 Hz, 1H), 2.95 – 2.90 (m, 1H), 2.74 (td, *J* = 4.6, 1.2 Hz, 1H), 2.47 (dd, *J* = 5.0, 2.7 Hz, 1H), 2.21 (dhept, *J* = 14.6, 7.8 Hz, 1H), 1.63 (tdd, *J* = 7.8, 5.6, 2.4 Hz, 2H).

**(1R,2R,5S)-bicyclo[3.1.0]hexan-2-ol (8)**

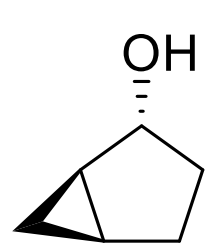


18.1 mL (107 mmol, 0.5 eq) of TMP was charged into a round bottom flask containing 21 g (214 mmol, 1 eq) of **7** in MTBE solution under $N_2$ atmosphere, and the contents were adjusted to 0°C, and stirred for 1 hour. Then 120 mL (300 mmol, 1.4 eq) of *n*-BuLi (2.5 M in hexane) was slowly transferred to this solution over 4 hours at 0°C. The reaction was quenched with aq $NH_4Cl$, and stirred at room temperature overnight. The aq layer was separated and back extracted with MTBE two times. All organic layers were combined, dried ($Na_2SO_4$) and concentrated to give **8** as a pale yellow oil (20 g, 204 mmol, 95%). **$^1$H-NMR** (400 MHz, $CDCl_3$) δ ppm: 4.20 (t, *J* = 4.9 Hz, 1H), 1.92 (tdd, *J* = 12.3, 8.3, 4.3 Hz, 1H), 1.80 (d, *J* = 4.9 Hz, 1H), 1.64 (dd, *J* = 12.5, 8.1 Hz, 1H), 1.52 (dd, *J* = 14.5, 8.3 Hz, 1H), 1.39 (ddt, *J* = 8.0, 5.6, 4.1 Hz, 1H), 1.36 – 1.27 (m, 2H), 0.41 (td, *J* = 8.2, 5.3 Hz, 1H), -0.01 (dt, *J* = 5.4, 3.7 Hz, 1H).

**(1R,5S)-bicyclo[3.1.0]hexan-2-one (9)**

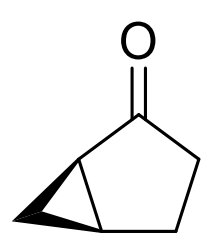

To a solution of 6.54 g (68 mmol) of **8** in DCM (300 mL) at 0°C was added portionwise DMP (45.7 g, 108 mmol, 1.6 eq). The reaction mixture was stirred at rt overnight, then quenched with 1M NaOH. The precipitate formed was filtered and the biphasic mixture was separated. The DCM layer was washed 4 times with 1M NaOH, the combined aq layers were extracted 2 times with DCM. All organic layers were combined, dried (Na2$SO_4$) filtered through a pad of celite eluting with DCM only. The solution was concentrated on rotavapor with no vacuum at 50°C, to obtain 18.2 g of a concentrated solution in DCM with a 1:3 ratio product:DCM (NMR), so 5.1 g of **9** (53.2 mmol, 79%). No other solvents than DCM were used in this step, because the boiling point of **9** is low enough to be partially evaporated with solvent with higher boiling point. **$^1$H-NMR** (400 MHz, $CDCl_3$) δ ppm: 2.19 – 1.94 (m, 5H), 1.78 – 1.72 (m, 1H), 1.22 – 1.15 (m, 1H), 0.92 (td, *J* = 4.6, 3.2 Hz, 1H); **$^{13}$C-NMR** (100 MHz, $CDCl_3$) δ ppm: 215.42, 53.56, 31.54, 27.58, 22.77, 21.74, 13.64.

**(1R,5R,E)-3-(hydroxyimino)bicyclo[3.1.0]hexan-2-one (10)**

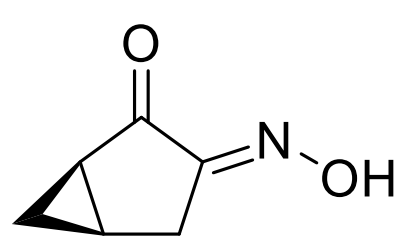

A solution of 9 (5.1 g, 53 mmol) in dry DCM (150 mL) cooled to -60°C was charged with TMSCl (7.4 mL, 58 mmol, 1.1 eq). Then was added dropwise isoamyl nitrite (8.6 mL, 64 mmol, 1.2 eq), and the cooling bath was slowly warm up to -30°C. The reaction was quenched with water, the aq layer was separated and extracted with DCM 3 times. All organic layers were combined, dried over $Na_2SO_4$, filtered and concentrated. The residue was purified by Si gel flash chromatography (60:40 Hexane:Ethyl Acetate), to give 1.84 g of product **10** (14.7 mmol, 28%). The enantiopurity of the product was evaluated on chiral HPLC, and the ratio measured was ≥99:1 (Data available at the end after crystallography data, Figure S1). **$^1$H-NMR** (400 MHz, $CDCl_3$) δ ppm: 2.95 (d, *J* = 19.0 Hz, 1H), 2.76 (ddd, *J* = 19.0, 4.8, 2.1 Hz, 1H), 2.20 – 2.12 (m, 2H), 1.44 – 1.37 (m, 1H), 0.87 (dt, *J* = 5.4, 4.0 Hz, 1H); **$^{13}$C-NMR** (100 MHz, $CDCl_3$) δ ppm: 198.97, 154.90, 28.35, 27.74, 16.27, 16.16; **HRMS** (APCI+) (m/z): $[M+H]^+$ Calculated 126.055, found 126.0540.

**(1R,5R,E)-spiro[bicyclo[3.1.0]hexane-2,2'-[1,3]dithiolan]-3-one oxime (11)**

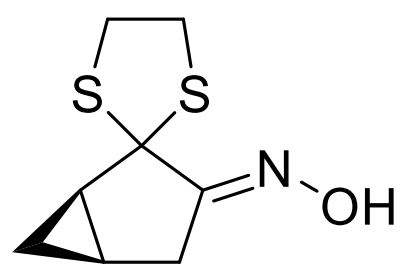

To a solution of **10** (1.12 g, 8.95 mmol) in THF (80 mL) was added AcOH (5.2 mL, 89.5 mmol, 10 eq), pTsOH·$H_2O$ (0.852 g, 4.48 mmol, 0.5 eq), and ethane dithiol (1.35 mL, 16.11 mmol, 1.8 eq). The reaction mixture was stirred at rt for 2 days at 35°C. Purified water and DCM were added to the reaction mixture, the DCM layer was extracted and washed two times with water, then was dried over $Na_2SO_4$, filtered and concentrated, before being purified by Si gel flash chromatography (80:20 Hexane:Ethyl Acetate) to give 1.47 g of product **11** (7.30 mmol, 82%). **$^1$H-NMR** (400 MHz, $CDCl_3$) δ ppm: 9.31 (s, 1H), 3.50 (ddd, *J* = 10.3, 5.5, 3.0 Hz, 1H), 3.43 (dd, *J* = 10.0, 5.0 Hz, 1H), 3.41 – 3.25 (m, 2H), 2.95 (d, *J* = 17.4 Hz, 1H), 2.65 (ddd, *J* = 17.4, 5.3, 1.7 Hz, 1H), 1.82 (ddd, *J* = 8.0, 6.2, 3.7 Hz, 1H), 1.56 (dddd, *J* = 7.7, 6.2, 5.3, 4.0 Hz, 1H), 0.85 (tdd, *J* = 7.7, 6.0, 1.7 Hz, 1H), 0.15 (dt, *J* = 6.0, 3.9 Hz, 1H); **$^{13}$C-NMR** (100 MHz, $CDCl_3$) δ ppm: 164.30, 72.32, 40.15, 38.90, 28.20, 24.62, 13.46, 12.30; **HRMS** (ESI+) (m/z): $[M+H]^+$ calculated 202.0355, found 202.0350.

**(1R,5R)-spiro[bicyclo[3.1.0]hexane-2,2'-[1,3]dithiolan]-3-one (12)**

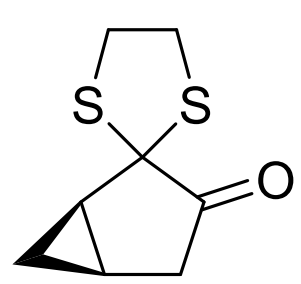

To a solution of 11 (1.46 g, 7.25 mmol) in 40 mL of 2-butanone and 20 mL of water was added pTsOH·$H_2O$ (2.76 g, 14.5 mmol, 2 eq). The reaction mixture was stirred and warmed up to reflux for 2 days. The reaction mixture was concentrated, portioned between DCM and purified water. The DCM layer was separated, dried over $Na_2SO_4$, filtered, and concentrated, to give **12** (1.2 g, 6.5 mmol, 89%), as a light brown crystal pure enough to be used next step without further purification. **$^1$H-NMR** (400 MHz, $CDCl_3$) δ ppm: 3.57 (ddd, *J* = 10.6, 5.6, 4.3 Hz, 1H), 3.44 (ddd, *J* = 10.6, 8.3, 5.5 Hz, 1H), 3.36 – 3.31 (m, 2H), 3.09 (ddd, *J* = 18.3, 5.6, 2.1 Hz, 1H), 2.28 (d, *J* = 18.3 Hz, 1H), 1.88 (dddd, *J* = 8.1, 7.0, 3.9, 0.5 Hz, 1H), 1.63 (tdd, *J* = 7.0, 5.6, 4.1 Hz, 1H), 1.05 (tdd, *J* = 8.0, 6.3, 2.1 Hz, 1H), 0.20 (dt, *J* = 6.3, 4.0 Hz, 1H). **$^{13}$C-NMR** (101 MHz, $CDCl_3$) δ ppm: 209.05, 71.64, 39.51, 38.83, 37.62, 21.32, 14.10, 11.32.

**Diisopropylammonium (Z)-2,2,2-trifluoro-1-((1R,5S)-3-oxospiro[bicyclo[3.1.0]hexane-2,2'-[1,3]dithiolan]-4-ylidene)ethan-1-olate (14)**

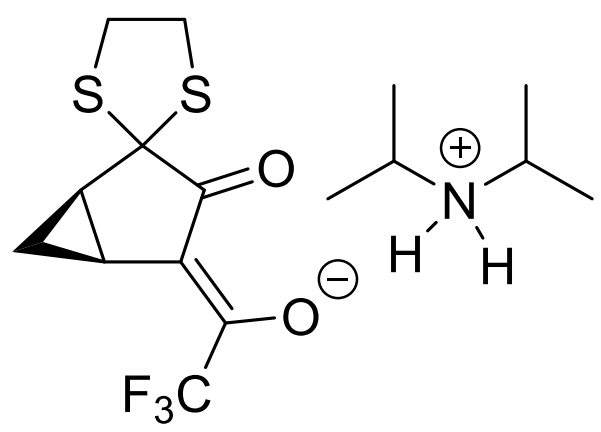

To a solution of 1.04 g (5.58 mmol) of **12** in 100 mL of dry THF was added ethyl trifluoroacetate (0.86 mL, 7.25 mmol,1.3 eq). After everything was dissolved, the reaction mixture was cooled to 0°C, and 1M LiHMDS (7.25 mL, 7.25 mmol, 1.3 eq) was added dropwise over 1h. After 3h of reaction at 0°C, the reaction mixture was warmed up to room temperature and quenched by adding slowly aqueous sulfuric acid solution made with 2.5 mL of $H_2SO_4$ (5 eq) in 10 mL of water. After 1 hour of stirring, the separated organic layer was washed 2 times with water, dried with $Na_2SO_4$, filtered and concentrated under reduced pressure. The concentrated solution obtained was agitated and charged dropwise with 0.94 mL (6.70 mmol, 1.2 equiv) of DIPA, and after 30 min, hexane (20 mL) was slowly added. The resulting precipitate formed was cooled to 0°C over 1 hour, then filtered through a fritted glass, and washed with hexane 2 times. The wet cake obtained was dried under vacuum. As a result, 1.89g (4.93 mmol, 88%) of **14** was obtained as a light-brown powder, pure enough to be used next step without any further purification. **$^1$H-NMR** (400 MHz, $CD_3CN$) δ ppm: 3.65 – 3.52 (m, 2H), 3.45 – 3.31 (m, 4H), 2.27 – 2.12 (m, 3H), 1.74 (td, *J* = 7.8, 4.1 Hz, 1H), 1.25 (s, 6H), 1.23 (s, 6H), 0.99 (ddd, *J* = 8.3, 6.7, 4.6 Hz, 1H), 0.13 (q, *J* = 4.1 Hz, 1H).

**Ethyl 2-((3bS,4aR)-3-(trifluoromethyl)-4,4a-dihydrospiro[cyclopropa[3,4]cyclopenta[1,2-c]pyrazole-5,2'-[1,3]dithiolane]-1(3bH)-yl)acetate (15)**

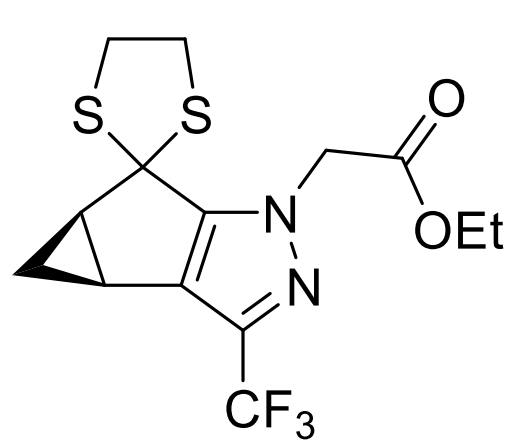

A 1.53 mL (21.47 mmol, 4 eq) portion of acetyl chloride was charged to precooled ethanol (30mL) over 30 min at 0°C. The solution was warmed up to room temperature before the addition of EHA hydrochloride (1.00 g, 6.48 mmol, 1.2 equiv), anhydrous LiCl (2.263 g, 53.4mmol, 10 eq), and **14** (2.07 g, 5.398 mmol) in 10 mL of ethanol. The reaction mixture was agitated for 1 day at 55°C. Then the reaction mixture was concentrated and charged with DCM and water, stirred for 15 min at room temperature, washed with a saturated $NaHCO_3$ solution. The DCM layer was then washed with an aqueous NaCl solution. The organic solution was separated, dried ($Na_2SO_4$), filtered through a pad of celite on fritted glass, and concentrated under reduced pressure. The residue was

purified by Si gel flash chromatography (70:30 Hexane:Ethyl Acetate) to give the compound **15** (1.54 g, 4.23 mmol, 78%). The absence of the undesired enantiomer was verified on this product by using Eu(hfc)$_3$ as chiral NMR shift reagent. The analysis was done with 10 mg of product, by addition of portions of 5 mg Eu(hfc)$_3$ before each analysis. ($^1$H-NMR with Eu(hfc)$_3$ available at the end after crystallography data, Figure S2). **$^1$H-NMR** (400 MHz, $CDCl_3$) δ ppm: 4.96 (d, *J* = 17.3 Hz, 1H), 4.88 (d, *J* = 17.3 Hz, 1H), 4.21 (qd, *J* = 7.2, 2.8 Hz, 2H), 3.53 – 3.44 (m, 4H), 2.61 (dt, *J* = 8.4, 5.1 Hz, 1H), 2.44 – 2.38 (m, 1H), 1.30 (td, *J* = 7.8, 5.7 Hz, 1H), 1.25 (t, *J* = 7.1 Hz, 3H), 0.64 (dt, *J* = 5.8, 4.0 Hz, 1H); **$^{13}$C-NMR** (100 MHz, $CDCl_3$) δ ppm: 167.04, 147.48, 129.33, 119.82, 65.66, 62.09, 51.27, 41.12, 40.88, 40.69, 23.48, 16.08, 14.18; **HRMS** (APCI+) (m/z): $[M+H]^+$ Calculated 365.0600, found 365.0669.

**Ethyl 2-((3bS,4aR)-5,5-difluoro-3-(trifluoromethyl)-3b,4,4a,5-tetrahydro-1H-cyclopropa[3,4]cyclopenta[1,2-c]pyrazol-1-yl)acetate (16)**

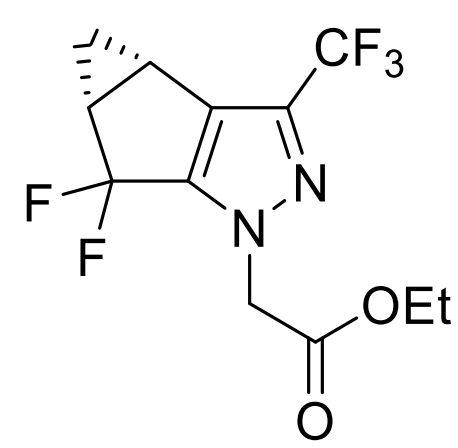


A portion of DBDMH (532 mg, 1.86 mmol, 3.2 eq) was introduced in a 25 mL PTFE sealed flask, with 6 mL of dry DCM. The mixture was stirred at room temperature within an ultrasound bath for 15 min. The flask was cooled to -20°C under Ar atmosphere, and HF·Pyr 70% (226 µL, 8.7 mmol, 20 eq) was added dropwise to the mixture. After 30 min stirring, a solution of **15** (212 mg, 0.58 mmol) in 4 mL of dry DCM was added dropwise over 10 min to the reaction mixture. After 3h of agitation at -15°C, the reaction mixture was warmed up to -10°C, and quenched with 10 mL of a saturated aq $NaHCO_3$ solution. The aqueous phase was further extracted with ethyl acetate 2 times, and all combined organic layers were dried over $Na_2SO_4$, filtered and concentrated under reduced pressure. The residue was purified by Si gel flash chromatography (95:5 to 90:10 Hexane:Ethyl Acetate), to give the difluorinated compound **16** (144 mg, 0.465 mmol, 80%). **$^1$H-NMR** (400 MHz, $CDCl_3$) δ ppm: 4.86 (s, 1H), 4.24 (q, *J* = 7.2 Hz, 2H), 2.52 – 2.41 (m, 2H), 1.40 (dddd, *J* = 8.8, 7.3, 5.8, 1.4 Hz, 1H), 1.27 (t, *J* = 7.1 Hz, 3H), 1.14 (dtd, *J* = 6.3, 4.0, 2.4 Hz, 1H); **$^{13}$C-NMR** (100 MHz, $CDCl_3$) δ ppm: 166.02, 143.40 (t, *J* = 29.3 Hz), 136.47 (q, *J* = 39.8 Hz), 132.84 (m), 120.69 (q, *J* = 268.8 Hz), 120.17 (t, *J* = 244.3 Hz), 62.47, 52.19, 28.11 (dd, *J* = 34.7, 29.3 Hz), 23.43 (d, *J* = 2.6 Hz), 14.10, 12.43 (dd, *J* = 4.0, 2.7 Hz); **HRMS** (ESI+) (m/z): $[M+H]^+$ calculated 311.0814, found 311.0810.

**Subunit A**

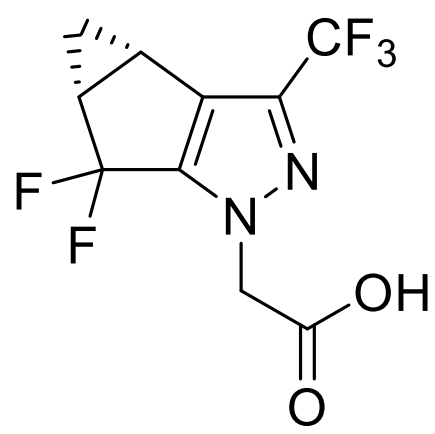


To a solution of **16** (110 mg, 0.353 mmol) in a 2:1 mixture of THF/MeOH (6.0 mL) contained in a 25 mL round-bottom flask was added 2N LiOH (0.35 mL, 0.70 mmol, 2 eq). The mixture was stirred for 2 h at ambient temperature and then concentrated to remove most of the THF and MeOH. The obtained aqueous phase was acidified with 1N HCl to adjust the pH to 2-3, and then extracted with EtOAc (15 mL x 2). The organic phase was dried over $Na_2SO_4$, filtered and concentrated in vacuo to provide **subunit A** (95 mg, 0.335 mmol, 95%) as a white/pale yellow solid. **$^1$H-NMR** (400 MHz, $CDCl_3$) δ ppm: 7.59 (br s, 1H), 4.93 (s, 2H), 2.54 – 2.41 (m, 2H), 1.41 (q, *J* = 7.3 Hz, 1H), 1.18 – 1.13 (m, 1H); **$^{13}$C-NMR** (100 MHz, $CDCl_3$) δ ppm: 170.8, 143.6 (t, *J* = 29.4 Hz), 136.8 (q, *J* = 39.6 Hz), 133.0, 120.6 (dd, *J* = 538.5, 268.5 Hz), 120.1 (t, *J* = 244.4 Hz), 51.5, 28.1 (dd, *J* = 34.6, 29.3 Hz), 23.5 (d, *J* = 2.6 Hz), 12.5 (t, *J* = 3.3 Hz); **HRMS** (ESI+) (m/z): $[M+H]^+$ calculated 283.0501, found 283.0500.

## 1.2 Synthesis of subunit B

a) Synthesis of cyclopentyl diastereomers

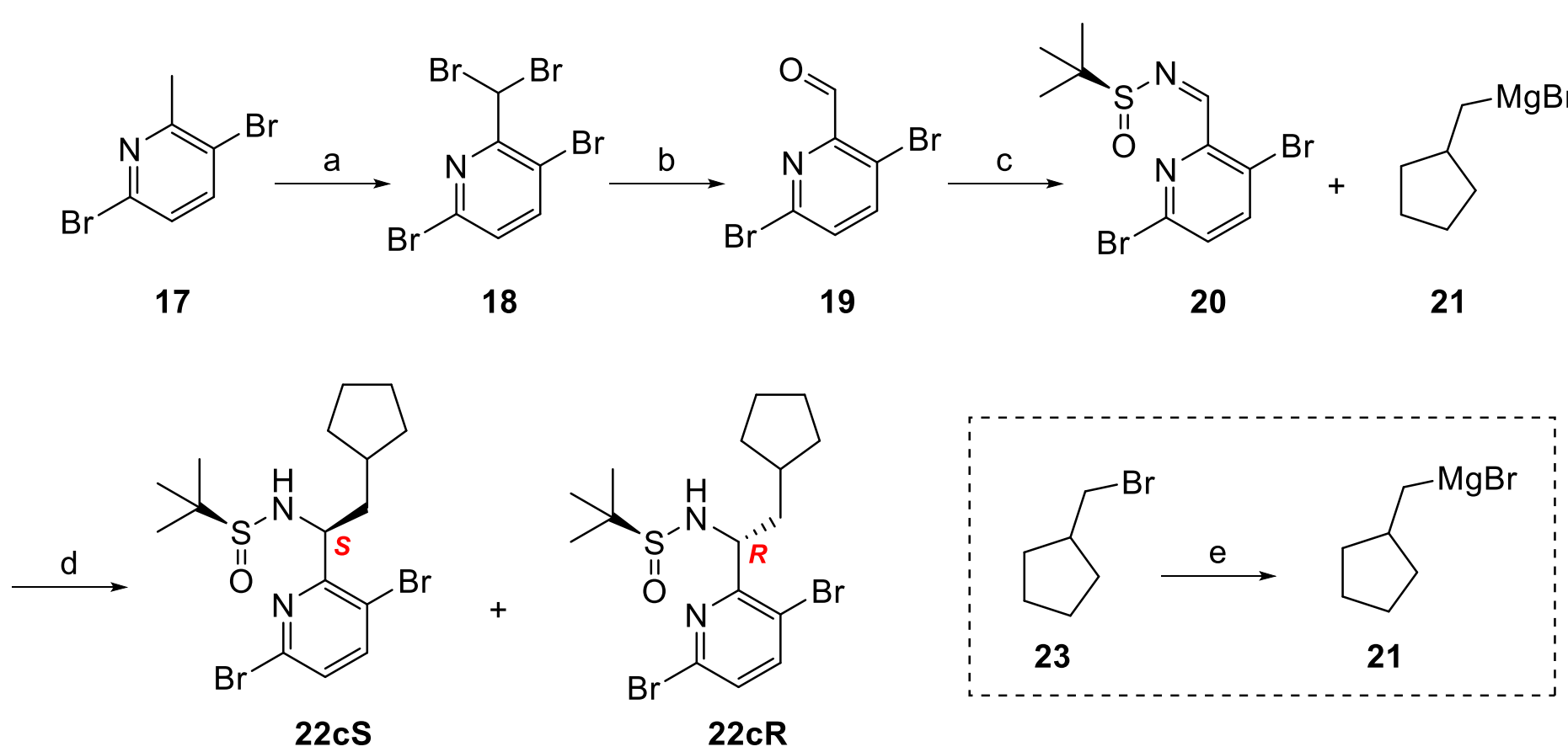

b) New general synthesis of cycloalkyl diastereomers

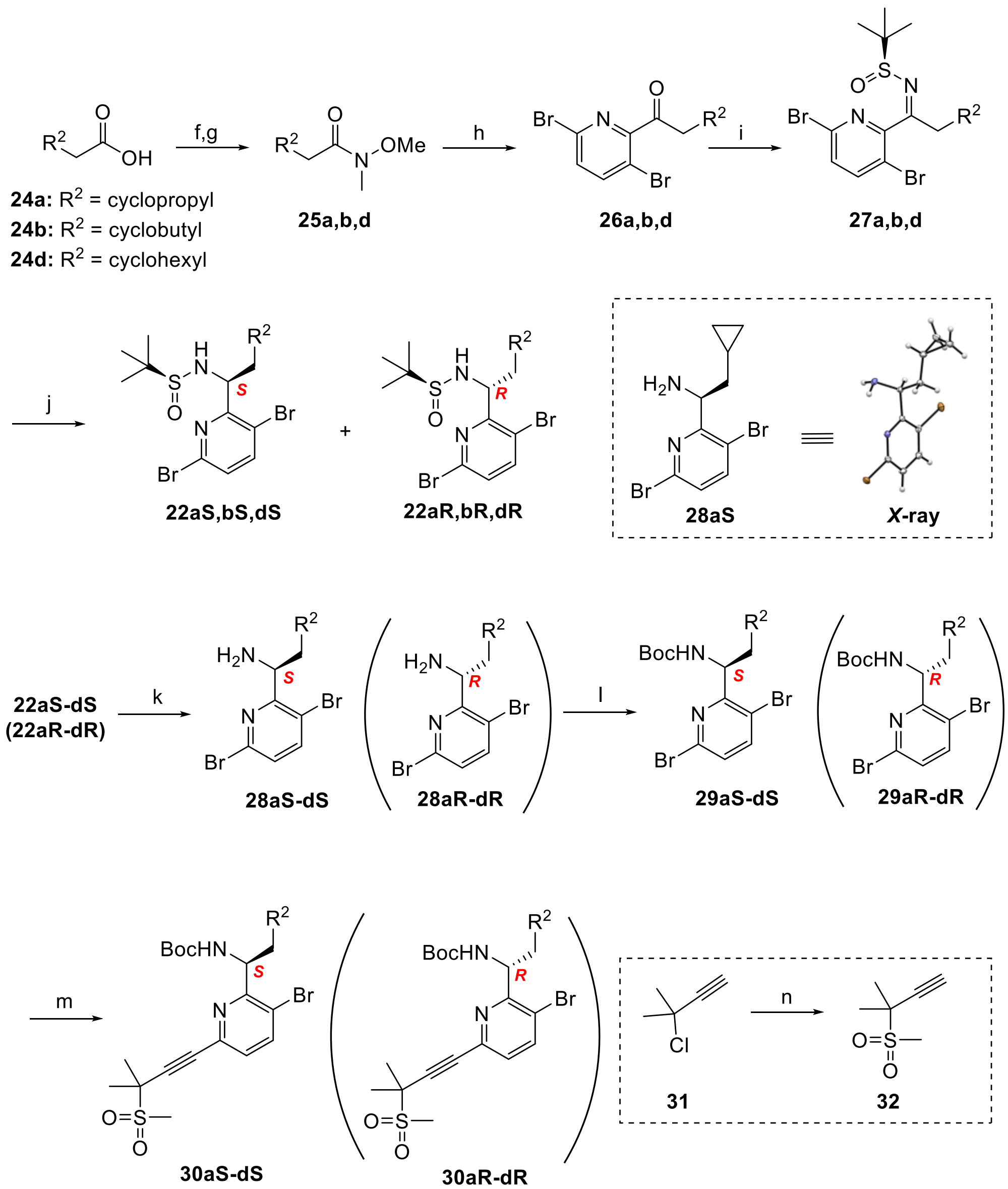


[a]Reagents and conditions: (a) NBS, AIBN, $CCl_4$, 80 ºC, 12 h; (b) $AgNO_3$, EtOH, $H_2O$, reflux, 5 h, 49% (two steps); (c) (*S*)-(-)-tert-Butanesulfinamide, $Cs_2CO_3$, DCM, rt, 1 h, 77%; (d) THF, -78 ºC, 2 h; (e) Mg, $I_2$, $Et_2O$, rt to 35 ºC, 1.5 h; (f) $SOCl_2$, DMF, DCM, rt, 2 h; (g) MeNHOMe.HCl, $Et_3N$, DCM, 0 ºC to rt, 1 h, 70-85% (two steps); (h) 2,5-dibromopyridine, TMPMgCl.LiCl, THF, -20 ºC, 2 h, 70-81%; (i) (*S*)-(-)-tert-butanesulfinamide, $Ti(OEt)_4$, THF, 70 ºC, 24 h, 47-50%; (j) $NaBH_4$, THF, 0 ºC to rt, 12 h; (k) 4 M HCl (in dioxane), MeOH, rt, 3 h; (l) $Boc_2O$, $Et_3N$, DCM, rt, 2 h, 93-

96% (two steps); (m) **32**, $Pd(PPh_3)_2Cl_2$, CuI, $Et_3N$, DMF, rt, 12 h, 50-82%; (n) sodium methanesulfinate, $Cu(OAc)_2$, TMEDA, iPrOAc, 20 ºC to 40 ºC, 12 h, 58%.

**Synthesis of 3,6-dibromo-2-(dibromomethyl)pyridine (18)**

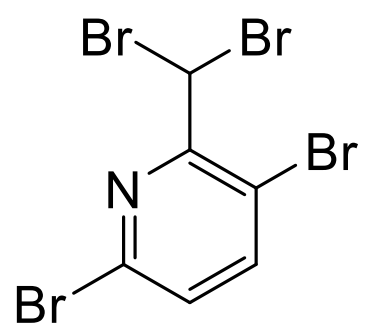


Compound **18** was prepared according to the reported literature procedure.[2] A solution of 3,6-dibromo-2-methylpyridine **17** (50.0 g, 201.6 mmol, 1.0 equiv.) in $CCl_4$ (250 mL) was placed in a round-bottom flask, followed by the addition of *N*-bromosuccinimide (107.0 g, 604.8 mmol, 3.0 equiv.) and Azobisisobutyronitrile (6.6 g, 40.3 mmol, 20 mol%). The reaction mixture was stirred at 80 ºC for 12 hours and then allowed to cool to room temperature. The resulting solid was removed by filtration through a Celite pad, and the filtrate was concentrated under reduced pressure. The crude product was used directly in the next step without further purification.

**Synthesis of 3,6-dibromopicolinaldehyde (19)**

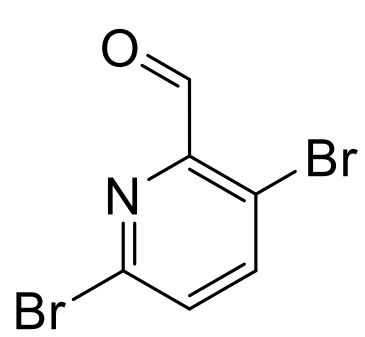


To a solution of **18** (20.0 g, 49.5 mmol, 1 equiv.) in EtOH (200 mL) was added a solution of silver nitrate (9.20 g, 54.4 mmol, 1.1 equiv.) in water (25 mL) dropwise. The reaction mixture was stirred at 80 ºC for 5 hours, then allowed to cool to room temperature. The resulting solid was filtered off, and the filtrate was concentrated and diluted with water. The mixture was extracted with ethyl acetate (3 x 30 mL). The combined organic layers were washed with brine (30 mL), dried over anhydrous $Na_2SO_4$, and concentrated under reduced pressure. The crude residue was purified by CombiFlash on silica gel (0-10% Hexane/EtOAc) to afford titled compound **19** (6.44 g, 49% yield over two steps, light yellow solid). $^1$H NMR matched with the reported literature.[2]

**Synthesis of (*S*)-*N*-((3,6-dibromopyridin-2-yl)methylene)-2-methylpropane-2-sulfinamide (20)**

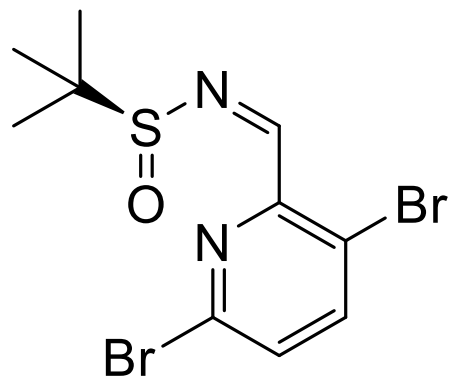


A 100-mL round-bottom flask was charged with compound **19** (6.44 g, 24.6 mmol, 1.0 equiv.), (*S*)-(-)-tert-Butanesulfinamide (3.57 g, 29.5 mmol, 1.2 equiv.), $Cs_2CO_3$ (9.59 g, 29.5 mmol, 1.2 equiv.), and DCM (30 mL). The mixture was stirred at room temperature for 1 hour. Water was then added, and the mixture was extracted with DCM (3 x 30 mL). The combined organic layers were washed with brine (30 mL), dried over anhydrous $Na_2SO_4$, and concentrated under reduced pressure. The crude residue was purified by CombiFlash on silica gel (0-20% Hexane/EtOAc) to afford titled compound **20** (2.70 g, 77%, light yellow powder). $^1$H NMR matched with the reported literature.[2]

**Synthesis of (cyclopentylmethyl)magnesium bromide (21)**

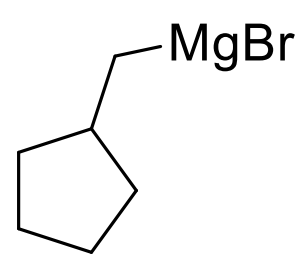


Compound **21** was prepared following the reported literature procedure. An oven-dried, two-neck round-bottom flask equipped with a magnetic stirrer bar was charged with freshly activated magnesium turnings (934.4 mg, 38.9 mmol, 1.5 equiv.) and iodine (326.9 mg, 2.6 mmol, 10 mol%). Anhydrous diethyl ether (10 mL) was added, and the flask was charged with argon. A solution of (Bromomethyl)cyclopentane **23** (3.3 mL, 25.9 mmol, 1.0 equiv.) was added dropwise over 10 minutes at room temperature. The resulting suspension was stirred at room temperature for 30 minutes, then stirred at 35 ºC for an additional 1 hour. The reaction was then cooled to room temperature and used directly in the next step. Note: The magnesium turnings were stirred under vacuum for 2 days at room temperature to remove surface oxide. After the addition of (Bromomethyl)cyclopentane, 5-10 minutes were required to initiate the Grignard formation.

**Synthesis of (S)-*N*-((S)-2-cyclopentyl-1-(3,6-dibromopyridin-2-yl)ethyl)-2-methylpropane-2-sulfinamide (22cS) and (S)-*N*-((R)-2-cyclopentyl-1-(3,6-dibromopyridin-2-yl)ethyl)-2-methylpropane-2-sulfinamide (22cR)**

To a round-bottom flask containing **20** (2.70 g, 7.4 mmol, 1.0 equiv.) in anhydrous THF at -78 ºC under an argon atmosphere was added **21** (25.9 mmol, 3.5 equiv.) dropwise. The resulting mixture was stirred for 2 hours while maintaining the temperature at -78 ºC. After 2 hours, the reaction mixture was quenched with saturated $NH_4Cl$, and the mixture was extracted with ethyl acetate (3 x 30 mL). The combined organic layers were washed with brine (30 mL), dried over anhydrous $Na_2SO_4$, and concentrated under reduced pressure. The crude residue was purified by flash chromatography on silica gel (Hexane/diethyl ether 1:1) by collecting small portions, to afford two separated diastereomers **22cS** and **22cR**. The yield was not calculated because a portion of a mixture of two diastereomers always remained. Both diastereomers are light yellow oils.

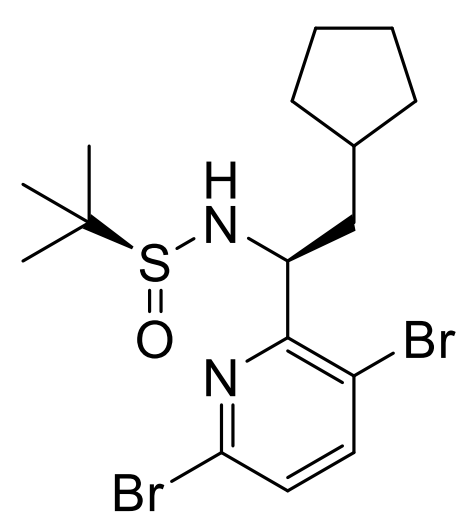


**$^1$H NMR** (400 MHz, $CDCl_3$) δ 7.63 (d, *J* = 8.3 Hz, 1H), 7.21 (d, *J* = 8.3 Hz, 1H), 4.80 – 4.75 (m, 1H), 4.74 (s, 1H), 2.07 – 1.97 (m, 1H), 1.89 – 1.80 (m, 1H), 1.79 – 1.70 (m, 2H), 1.67 – 1.57 (m, 3H), 1.56 – 1.48 (m, 2H), 1.26 (s, 9H), 1.24 – 1.18 (m, 1H), 1.16 – 1.07 (m, 1H); **$^{13}$C NMR** (100 MHz, $CDCl_3$) δ 161.9, 142.7, 140.0, 127.9, 118.2, 58.5, 56.1, 43.2, 36.6, 33.1, 31.8, 25.1, 22.8; **HRMS** ($ESI^+$) Calculated for $C_{16}H_{24}Br_2N_2OS$ $[M+H]^+$ 453.0034; found 453.0034.

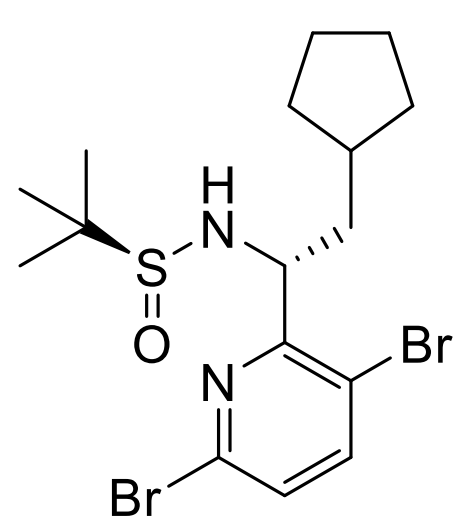


**$^1$H NMR** (400 MHz, $CDCl_3$) δ 7.69 (d, *J* = 8.3 Hz, 1H), 7.26 (d, *J* = 8.3 Hz, 1H), 4.88 (dt, *J* = 9.0, 6.9 Hz, 1H), 4.15 (d, *J* = 9.0 Hz, 1H), 2.00 – 1.85 (m, 3H), 1.84 – 1.76 (m, 2H), 1.66 – 1.55 (m, 2H), 1.54 – 1.45 (m, 2H), 1.20 – 1.10 (m, 2H), 1.16 (s, 9H); **$^{13}$C NMR** (100 MHz, $CDCl_3$) δ 161.1, 142.7, 140.2, 127.9, 118.7, 58.3, 56.3, 43.2, 36.4, 33.0, 32.2, 25.1, 25.1, 22.4; **HRMS** ($ESI^+$) Calculated for $C_{16}H_{24}Br_2N_2OS$ $[M+H]^+$ 453.0034; found 453.0028.

**New general synthesis of cycloalkyl diastereomers:**

**General Procedure A**

The corresponding Weinreb amides were prepared according to the literature procedure.[3] To a solution of the corresponding carboxylic acid (1.0 equiv.), and DMF (a few drops) in DCM (30 mL) was added thionyl chloride (2.0 equiv.) dropwise at room temperature. The reaction mixture was stirred for 2 hours. Then, the solvent was removed under reduced pressure to afford the corresponding crude acid chloride.

To a solution of crude acid chloride in DCM (25 mL) was added N,O-dimethyl hydroxylamine hydrochloride (1.5 equiv.). The reaction mixture was cooled to 0 ºC, and triethylamine (1.6 equiv.) was added dropwise. The mixture was then stirred at room temperature for 1 hour. Upon completion of the reaction, sat. aq. $NaHCO_3$ was added, and the mixture was extracted with DCM (3 x 30 mL). The combined organic layers were washed with brine (20 mL), dried over anhydrous $Na_2SO_4$, and concentrated under reduced pressure to afford the corresponding Weinreb amide.

**General Procedure B**

The corresponding ketones were prepared following a literature procedure.[4] To a 100 mL round-bottom flask containing 2.5-dibromopyridine (1.0 equiv.) in anhydrous THF (40 mL) was added TMPMgCl.LiCl (1.2 equiv.) *via* syringe pump over 30 minutes at -20 ºC under argon atmosphere. The reaction mixture was stirred for 40 minutes, followed by the dropwise addition of the corresponding Weinreb amide (1.5 equiv.) in THF. The resulting mixture was then stirred at -20 ºC for an additional 2 hours. The mixture was acidified with 2 M HCl to adjust the pH = 1-2, then basified with 5% NaOH to bring pH = 9-10. The mixture was then extracted with ethyl acetate (3 x 30 mL), and the combined organic layers were washed with brine (30 mL), dried over anhydrous $Na_2SO_4$, and concentrated under reduced pressure. The crude residue was purified by CombiFlash on silica gel (0-10% Hexane/EtOAc) to afford the desired ketone.

**General Procedure C**

The corresponding *N*-sulfinyl imines were prepared according to the literature procedure.[5] The corresponding ketone (1.0 equiv.) and (*S*)-(-)-tert-Butanesulfinamide (2.0 equiv.) were dissolved in THF (30 mL), followed by the addition of $Ti(OEt)_4$ (4.0 equiv.). The reaction mixture was stirred at 70 °C for 24 hours. After completion, water was added, and the resulting solid was removed by filtration through a Celite pad. The filtrate was extracted with ethyl acetate (3 x 30 mL). The combined organic layers were washed with brine (30 mL), dried over anhydrous $Na_2SO_4$, and concentrated under reduced pressure. The crude residue was purified by CombiFlash on silica gel (0-20% Hexane/EtOAc) to afford the desired *N*-sulfinyl imine.

**General Procedure D**

The corresponding diastereomers were synthesized following a modified literature procedure.[5] $NaBH_4$ (3.0 equiv.) was added portion-wise to a cooled (0 °C) solution of corresponding *N*-sulfinyl imine (1.0 equiv.) in anhydrous THF (10 mL). The resulting reaction mixture was stirred at 0 °C for approximately 3 hours and then stirred at room temperature for another 9 hours. The reaction was quenched by the slow addition of water, followed by extraction with ethyl acetate (3 x 20 mL). The combined organic layers were washed with brine (30 mL), dried over anhydrous $Na_2SO_4$, and concentrated under reduced pressure. The crude residue was purified by CombiFlash on silica gel (0-30% Hexane/EtOAc) or preparative HPLC to afford the desired diastereomers.

**Synthesis of 2-cyclopropyl-N-methoxy-N-methylacetamide (25a)**

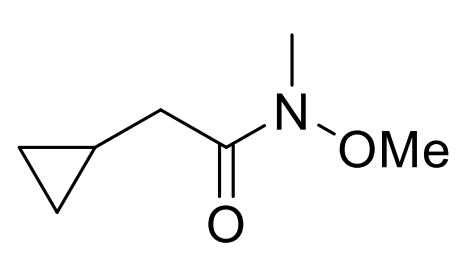


The title compound was prepared according to general procedure A using Cyclopropylacetic acid **24a** (5.0 g, 50.0 mmol, 1.0 equiv.). Yield is 85% (6.08 g, colorless oil). **$^1$H NMR** (400 MHz, $CDCl_3$) δ 3.49 (s, 3H), 3.02 (s, 3H), 2.18 (d, *J* = 6.9 Hz, 2H), 1.00 – 0.85 (m, 1H), 0.45 – 0.32 (m, 2H), 0.02 – -0.04 (m, 2H). $^1$H NMR matches with previously reported literature.

**Synthesis of 2-cyclobutyl-N-methoxy-N-methylacetamide (25b)**

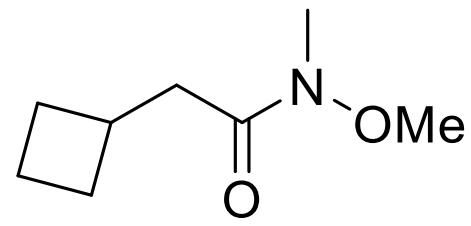


The title compound was prepared according to general procedure A using Cyclobutylacetic acid **24b** (3.0 g, 26.3 mmol, 1.0 equiv.). Yield is 91% (3.77 g, colorless oil). **$^1$H NMR** (400 MHz, $CDCl_3$) δ 3.68 (d, *J* = 1.9 Hz, 3H), 3.16 (s, 3H), 2.81 – 2.67 (m, 1H), 2.54 (d, *J* = 8.8 Hz, 2H), 2.20 – 2.09 (m, 2H), 1.98 – 1.80 (m, 2H), 1.75 – 1.64 (m, 2H). $^1$H NMR matches with previously reported literature.

**Synthesis of 2-cyclohexyl-N-methoxy-N-methylacetamide (25d)**

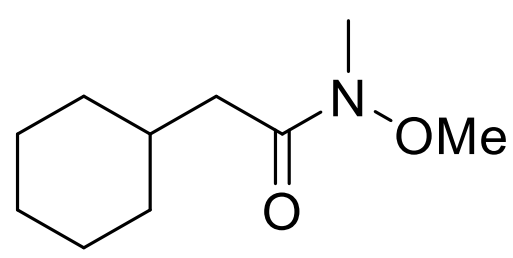


The title compound was prepared according to general procedure A using Cyclohexylacetic acid **24d** (8.3 g, 58.4 mmol, 1.0 equiv.). Yield is 70% (7.57 g). $^1$H NMR match with reported data. **$^1$H NMR** (400 MHz, $CDCl_3$) δ 3.67 (s, 3H), 3.18 (s, 3H), 2.30 (d, *J* = 7.0 Hz, 2H), 1.85 (ttt, *J* = 10.6, 7.0, 3.4 Hz, 1H), 1.78 – 1.74 (m, 1H), 1.74 – 1.69 (m, 2H), 1.68 – 1.61 (m, 2H), 1.35 – 1.21 (m, 2H), 1.21 – 1.08 (m, 2H), 0.97 (qd, *J* = 12.4, 12.0, 2.9 Hz, 1H).

**Synthesis of 2-cyclobutyl-1-(3,6-dibromopyridin-2-yl)ethan-1-one (26b)**

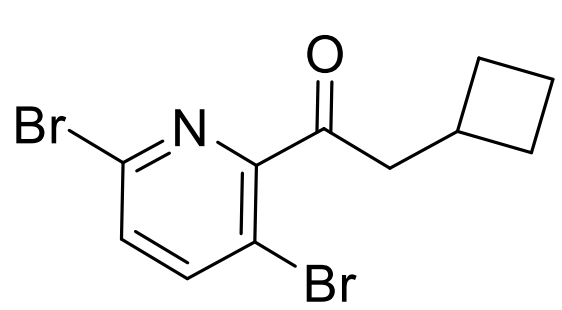


The title compound was prepared according to general procedure B using Weinreb amide **25b** (3.77 g, 24.0 mmol, 1.5 equiv.). Product was purified by CombiFlash on silica gel (0-10% Hexane/EtOAc) (4.67 g, 77% yield, light yellow oil). **$^1$H NMR** (400 MHz, $CDCl_3$) δ 7.78 (d, *J* = 8.4 Hz, 1H), 7.41 (d, *J* = 8.3 Hz, 1H), 3.16 (d, *J* = 7.3 Hz, 2H), 2.77 (hept, *J* = 8.1 Hz, 1H), 2.23 – 2.08 (m, 2H), 1.95 – 1.82 (m, 2H), 1.79 – 1.66 (m, 2H); **$^{13}$C NMR** (100 MHz, $CDCl_3$) δ 199.7, 153.7, 144.4, 139.3, 131.0, 116.5, 46.9, 31.4, 28.5, 18.9; **HRMS** (APCI$^+$) Calculated for $C_{11}H_{11}Br_2NO$ $[M+H]^+$ 333.9265; found 333.9225.

**Synthesis of 2-cyclohexyl-1-(3,6-dibromopyridin-2-yl)ethan-1-one (26d)**

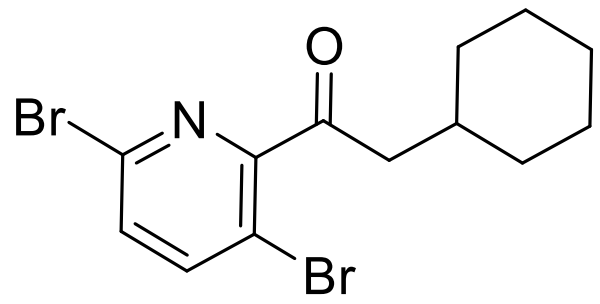

The title compound was prepared according to general procedure B using Weinreb amide **25d** (7.6 g, 40.9 mmol, 1.5 equiv.). Product was purified by CombiFlash on silica gel (0-10% Hexane/EtOAc) (7.98 g, 81% yield, light yellow solid). **$^1$H NMR** (400 MHz, $CDCl_3$) δ 7.79 (d, *J* = 8.4 Hz, 1H), 7.42 (d, *J* = 8.4 Hz, 1H), 2.92 (d, *J* = 6.8 Hz, 2H), 2.0 – 1.86 (m, 1H), 1.81 – 1.73 (m, 2H), 1.72 – 1.58 (m, 3H), 1.35 – 1.22 (m, 2H), 1.20 – 1.11 (m, 1H), 1.01 (qd, *J* = 12.5, 3.1 Hz, 2H); **$^{13}$C NMR** (100 MHz, $CDCl_3$) δ 200.1, 154.1, 144.4, 139.4, 131.0, 116.5, 47.4, 33.8, 33.2, 26.2, 26.1; **HRMS** (APCI$^+$) Calculated for $C_{13}H_{15}Br_2NO$ $[M+H]^+$ 361.9578; found 361.9578.

**Synthesis of (*S*)-*N*-(2-cyclopropyl-1-(3,6-dibromopyridin-2-yl)ethylidene)-2-methylpropane-2-sulfinamide (27a)**

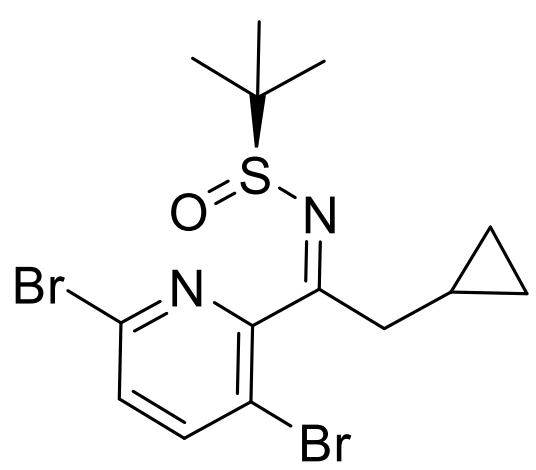

The title compound was prepared according to general procedure C using ketone **26a** (10.3 g, 32.9 mmol, 1.0 equiv.). Product was purified by CombiFlash on silica gel (0-20% Hexane/EtOAc) (6.57 g, 47% yield, light yellow oil). **$^1$H NMR** (400 MHz, $CDCl_3$) δ 7.61 (d, *J* = 8.4 Hz, 1H), 7.28 (d, *J* = 8.5 Hz, 1H), 2.63 (d, *J* = 7.2 Hz, 2H), 1.26 (s, 9H), 1,10 – 0.97 (m, 1H), 0.60 – 0.45 (m, 2H), 0.22 – 0.12 (m, 1H), 0.11 – 0.03 (m, 1H); **$^{13}$C NMR** (100 MHz, $CDCl_3$) δ 158.1, 143.6, 141.2, 139.4, 128.8, 116.5, 58.4, 45.9, 22.9 (3C), 7.2, 4.8, 4.6; **HRMS** (APCI$^+$) Calculated for $C_{14}H_{18}Br_2N_2OS$ $[M+H]^+$ 420.9585; found 420.9546.

**Synthesis of (*S,Z*)-*N*-(2-cyclobutyl-1-(3,6-dibromopyridin-2-yl)ethylidene)-2-methylpropane-2-sulfinamide (27b)**

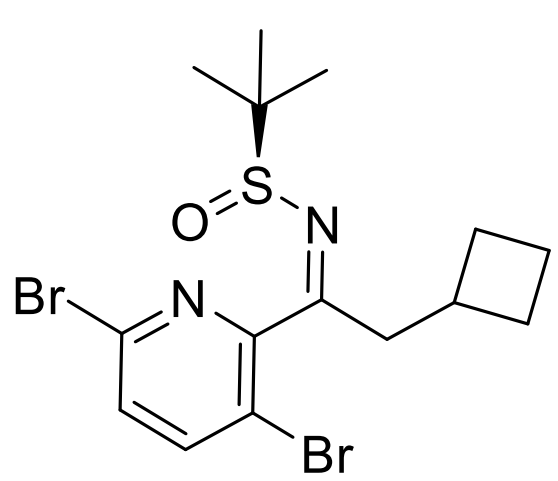

The title compound was prepared according to general procedure C using ketone **26b** (4.67 g, 14.1 mmol, 1.0 equiv.). Product was purified by CombiFlash on silica gel (0-20% Hexane/EtOAc) (2.87 g, 47% yield, light yellow oil). **$^1$H NMR** (400 MHz, $CDCl_3$) δ 7.62 (d, *J* = 8.4 Hz, 1H), 7.29 (d, *J* = 8.4 Hz, 1H), 2.99 – 2.86 (m, 1H), 2.86 – 2.71 (m, 2H), 2.25 – 2.07

(m, 2H), 2.01 – 1.84 (m, 2H), 1.81 – 1.67 (m, 2H), 1.25 (s, 9H); **$^{13}$C NMR** (100 MHz, $CDCl_3$) δ 158.2, 141.2, 139.4, 128.7 (2C), 116.2, 58.2, 47.8, 32.1, 28.5 (2C), 22.9 (3C), 18.8; **HRMS** ($ESI^+$) Calculated for $C_{15}H_{20}Br_2N_2OS$ $[M+H]^+$ 434.9741; found 434.9654.

**Synthesis of (*S*)-N-((*S*)-2-cyclopropyl-1-(3,6-dibromopyridin-2-yl)ethyl)-2-methylpropane-2-sulfinamide (22aS), and (*S*)-N-((*R*)-2-cyclopropyl-1-(3,6-dibromopyridin-2-yl)ethyl)-2-methylpropane-2-sulfinamide (22aR)**

The title compounds were prepared according to general procedure D using *N*-sulfinyl imine **27a** (6.57 g, 15.7 mmol, 1.0 equiv.). The product was purified by preparative HPLC using Water/ACN as the mobile phase.

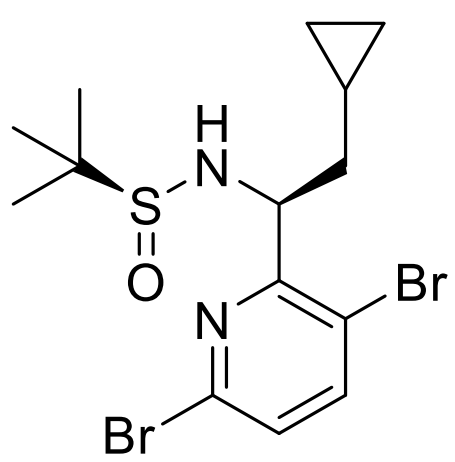


**$^{1}$H NMR** (400 MHz, $CDCl_3$) δ 7.64 (d, $J$ = 8.3 Hz, 1H), 7.22 (d, $J$ = 8.3 Hz, 1H), 4.93 – 4.83 (m, 1H), 4.79 (d, $J$ = 9.3 Hz, 1H), 1.82 (ddd, $J$ = 13.9, 7.9, 5.9 Hz, 1H), 1.44 (ddd, $J$ = 13.7, 7.9, 5.4 Hz, 1H), 1.27 (s, 9H), 0.85 – 0.73 (m, 1H), 0.52 – 0.35 (m, 2H), 0.10 – -0.01 (m, 2H); **$^{13}$C NMR** (100 MHz, $CDCl_3$) δ 161.4, 142.65, 139.9, 127.9, 118.6, 59.5, 55.9, 42.1, 22.8, 7.8, 5.1, 4.2; **HRMS** ($ESI^+$) Calculated for $C_{14}H_{20}Br_2N_2OS$ $[M+H]^+$ 424.9721; found 424.9723.

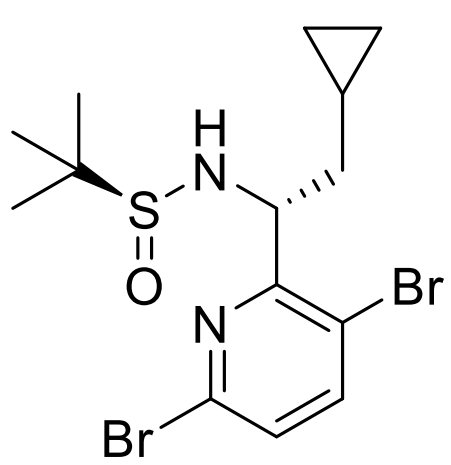


**$^{1}$H NMR** (400 MHz, $CDCl_3$) δ 7.64 (d, $J$ = 8.2 Hz, 1H), 7.22 (d, $J$ = 8.3 Hz, 1H), 5.02 – 4.91 (m, 1H), 4.27 (d, $J$ = 9.1 Hz, 1H), 1.91 (dt, $J$ = 13.3, 6.6 Hz, 1H), 1.68 (dt, $J$ = 13.9, 7.3 Hz, 1H), 1.15 (s, 9H), 0.80 – 0.66 (m, 1H), 0.48 – 0.39 (m, 1H), 0.38 – 0.31 (m, 1H), 0.09 – 0.01 (m, 1H), -0.02 – -0.10 (m, 1H); **$^{13}$C NMR** (100 MHz, $CDCl_3$) δ 160.9, 142.5, 140.1, 127.9, 119.1, 58.7, 56.4, 41.9, 22.5, 7.6, 4.9, 4.6; **HRMS** ($APCI^+$) Calculated for $C_{14}H_{20}Br_2N_2OS$ $[M+H]^+$ 424.9721; found 424.9720.

**Synthesis of (*R*)-*N*-((*S*)-2-cyclobutyl-1-(3,6-dibromopyridin-2-yl)ethyl)-2-methylpropane-2-sulfinamide (22bS) and (*R*)-*N*-((*R*)-2-cyclobutyl-1-(3,6-dibromopyridin-2-yl)ethyl)-2-methylpropane-2-sulfinamide (22bR)**

The title compounds were prepared according to general procedure D using *N*-sulfinyl imine **27b** (2.87 g, 6.63 mmol, 1.0 equiv.). The product was purified by preparative HPLC using Water/ACN as the mobile phase.

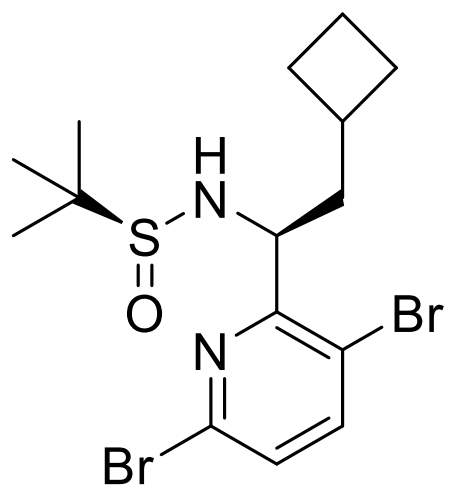


**$^{1}$H NMR** (400 MHz, $CDCl_3$) δ 7.63 (d, *J* = 8.3 Hz, 1H), 7.21 (d, *J* = 8.3 Hz, 1H), 4.76 – 4.63 (m, 2H), 2.47 (hept, *J* = 7.6 Hz, 1H), 2.08 – 1.96 (m, 2H), 1.92 – 1.76 (m, 4H), 1.73 – 1.55 (m, 2H), 1.26 (s, 9H); $^{13}$C NMR (100 MHz, $CDCl_3$) δ 161.5, 142.6, 139.98, 127.9, 118.3, 57.5, 55.9, 43.9, 32.7, 28.6, 27.9, 22.8 (3C), 18.5; **HRMS** ($ESI^+$) Calculated for $C_{15}H_{22}Br_2N_2OS$ $[M+H]^+$ 436.9898; found 436.9895.

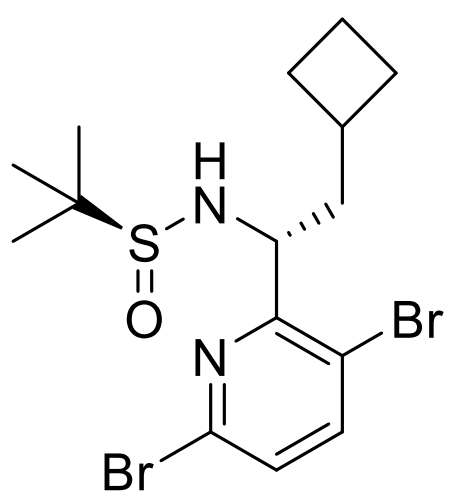


**$^{1}$H NMR** (400 MHz, $CDCl_3$) δ 7.65 (d, *J* = 8.3 Hz, 1H), 7.23 (d, *J* = 8.3 Hz, 1H), 4.80 (dt, *J* = 9.1, 6.9 Hz, 1H), 4.14 (d, *J* = 9.0 Hz, 1H), 2.41 (hept, *J* = 7.6 Hz, 1H), 2.11 – 1.92 (m, 4H), 1.90 – 1.71 (m, 2H), 1.70 – 1.54 (m, 2H), 1.15 (s, 9H); **$^{13}$C NMR** (100 MHz, $CDCl_3$) δ 160.9, 142.6, 140.2, 127.9, 118.8, 57.1, 56.4, 44.0, 32.5, 28.6, 28.2, 22.5 (3C), 18.5; **HRMS** ($ESI^+$) Calculated for $C_{15}H_{22}Br_2N_2OS$ $[M+H]^+$ 436.9898; found 436.9898.

**Synthesis of (*S*)-*N*-((*S*)-2-cyclohexyl-1-(3,6-dibromopyridin-2-yl)ethyl)-2-methylpropane-2-sulfinamide (22dS) and *S*)-*N*-((*R*)-2-cyclohexyl-1-(3,6-dibromopyridin-2-yl)ethyl)-2-methylpropane-2-sulfinamide (22dR)**

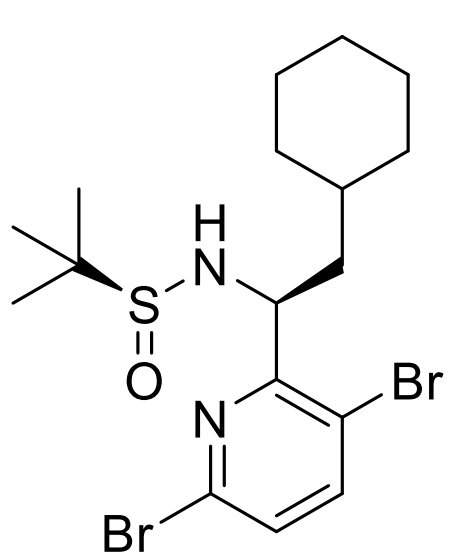


The title compounds were prepared according to general procedure D using *N*-sulfinyl imine **27d** (4.86 g, 10.5 mmol, 1.0 equiv.). Product was purified by CombiFlash on silica gel (0-30% Hexane/EtOAc).

**$^{1}$H NMR** (400 MHz, $CDCl_3$) δ 7.63 (d, *J* = 8.3 Hz, 1H), 7.21 (d, *J* = 8.3 Hz, 1H), 4.89 – 4.80 (m, 1H), 4.72 (d, *J* = 9.6 Hz, 1H), 1.97 – 1.86 (m, 1H), 1.82 – 1.60 (m, 5H), 1.58 – 1.48 (m, 2H), 1.27 (s, 9H), 1.24 – 1.13 (m, 3H), 1.03 –

0.90 (m, 2H); **$^{13}$C NMR** (100 MHz, $CDCl_3$) δ 162.0, 142.7, 140.0, 127.9, 118.0, 56.6, 56.1, 44.3, 34.1, 34.0, 31.7, 26.5, 26.4, 26.0, 22.8; **HRMS** (ESI$^+$) Calculated for $C_{17}H_{26}Br_2N_2OS$ $[M+H]^+$ 467.0190; found 467.0189.

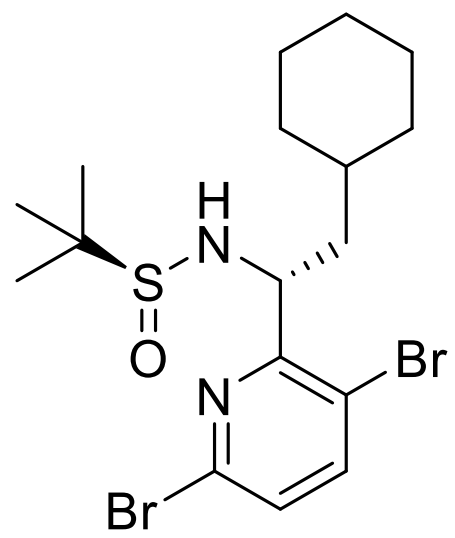


**$^1$H NMR** (400 MHz, $CDCl_3$) δ 7.66 (d, $J$ = 8.3 Hz, 1H), 7.24 (d, $J$ = 8.3 Hz, 1H), 5.01 – 4.91 (m,1H), 4.09 (d, $J$ = 8.9 Hz, 1H), 1.90 – 1.79 (m, 1H), 1.77 – 1.59 (m, 6H), 1.54 – 1.39 (m, 1H), 1.33 – 1.94 (m, 3H), 1.15 (s, 9H), 1.05 – 0.89 (m, 2H); **$^{13}$C NMR** (100 MHz, $CDCl_3$) δ 161.4, 142.7, 140.3, 127.9, 118.6, 56.4, 44.4, 33.8, 33.80 32.6, 26.45, 26.1, 25.9, 22.4; **HRMS** (ESI$^+$) Calculated for $C_{17}H_{26}Br_2N_2OS$ $[M+H]^+$ 467.0190; found 467.0192.

**General Procedure E**

To the solution of the corresponding diastereomer (1.0 equiv.) in MeOH was added 4M HCl in dioxane. The resulting mixture was stirred at room temperature for 3 hours, then concentrated under reduced pressure to afford a crude white solid, which was used directly in the next step.

The crude white solid was dissolved in DCM (5 mL), and triethylamine (2.0 equiv.) was added dropwise at room temperature. Di-*tert*-butyl decarbonate (1.2 equiv.) was then added portion-wise, and the mixture was stirred for 2 hours. The resulting reaction mixture was diluted with water and extracted with DCM (3 x 20 mL). The combined organic layers were washed with brine (20 mL), dried over anhydrous $Na_2SO_4$, and concentrated under reduced pressure. The crude residue was purified by CombiFlash on silica gel (0-10% Hexane/EtOAc) to afford the corresponding *N*-Boc-protected bis-bromopyridine.

**General Procedure F**

To the solution of corresponding *N*-Boc-protected bis-bromopyridine (1.0 equiv.), **32** (1.2 equiv.), and triethylamine (3.0 equiv.) in DMF (5 mL), were added CuI (5 mol%) and $Pd(PPh_3)_2Cl_2$ (5 mol%). The reaction was stirred under an argon atmosphere at room temperature for 12 hours. It was then diluted with water and ethyl acetate (30 mL). The organic layer was washed with water (5 x 20 mL). The organic layer was collected and washed with brine (20 mL), dried over anhydrous

$Na_2SO_4$, and concentrated under reduced pressure. The crude residue was purified by CombiFlash on silica gel (0-30% Hexane/EtOAc) to afford the corresponding subunit B.

**Synthesis of tert-butyl (*S*)-(2-cyclopropyl-1-(3,6-dibromopyridin-2-yl)ethyl)carbamate (29aS)**

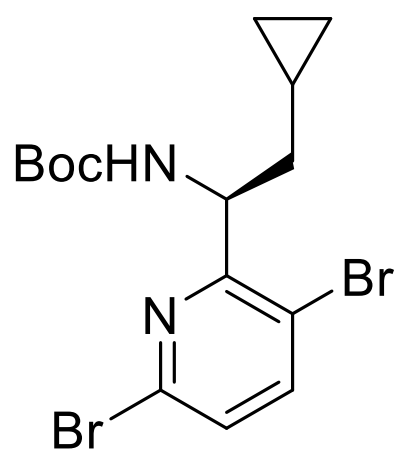


The title compound was prepared according to general procedure E using diastereomer **22aS** (1.53 g, 3.6 mmol, 1.0 equiv.). Product was purified by CombiFlash on silica gel (0-10% Hexane/EtOAc) (1.40 g, 92% yield, light yellow oil). **$^1$H NMR** (400 MHz, $CDCl_3$) δ 7.65 (d, *J* = 8.3 Hz, 1H), 7.23 (d, *J* = 8.3 Hz, 1H), 5.60 (d, *J* = 9.2 Hz, 1H), 5.38 – 5.28 (m, 1H), 1.71 – 1.56 (m, 2H), 1.45 (s, 9H), 0.79 – 0.65 (m, 1H), 0.49 – 0.34 (m, 2H), 0.07 – 0.00 (m, 1H), -0.02 – -0.09 (m, 1H); **$^{13}$C NMR** (100 MHz, $CDCl_3$) δ 160.7, 155.2, 142.5, 140.0, 127.8, 118.9, 79.4, 53.8, 40.7, 28.4, 7.4, 4.8, 4.3; **HRMS** ($APCI^+$) Calculated for $C_{15}H_{20}Br_2N_2O_2$ $[M+H]^+$ 420.9949; found 420.9950.

**Synthesis of tert-butyl (*R*)-(2-cyclopropyl-1-(3,6-dibromopyridin-2-yl)ethyl)carbamate (29aR)**

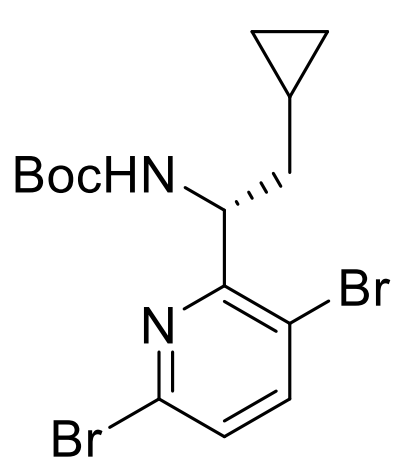


The title compound was prepared according to general procedure E using diastereomer **22aR** (2.00 g, 4.76 mmol, 1.0 equiv.). Product was purified by CombiFlash on silica gel (0-10% Hexane/EtOAc) (1.74 g, 87% yield, light yellow oil). **$^1$H NMR** (400 MHz, $CDCl_3$) δ 7.64 (d, *J* = 8.3 Hz, 1H), 7.22 (d, *J* = 8.3 Hz, 1H), 5.58 (d, *J* = 9.6 Hz, 1H), 5.31 (q, *J* = 6.8 Hz, 1H), 1.68 – 1.54 (m, 2H), 1.43 (s, 9H), 0.78 – 0.64 (m, 1H), 0.47 – 0.32 (m, 2H), 0.05 – -0.01 (m, 1H), -0.03 – -0.12 (m, 1H); $^{13}$C NMR (100 MHz, $CDCl_3$) δ 160.8, 155.2, 142.6, 140.0, 127.8, 119.0, 79.4, 53.8, 40.7, 28.4 (3C), 7.4, 4.8, 4.3; **HRMS** ($ESI^+$) Calculated for $C_{15}H_{20}Br_2N_2O_2$ $[M+Na]^+$ 442.9769; found 442.9720.

**Synthesis of tert-butyl (*S*)-(2-cyclobutyl-1-(3,6-dibromopyridin-2-yl)ethyl)carbamate (29bS)**

The title compound was prepared according to general procedure E using diastereomer **22bS** (300.8 mg, 0.69 mmol, 1.0 equiv.). Product was purified by CombiFlash on silica gel (0-10%

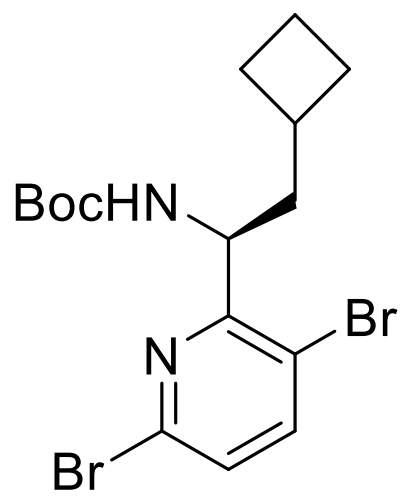

Hexane/EtOAc) (278.4 mg, 93% yield, light yellow oil). **$^{1}$H NMR** (400 MHz, $CDCl_3$) δ 7.64 (d, *J* = 8.2 Hz, 1H), 7.22 (d, *J* = 8.3 Hz, 1H), 5.49 (d, *J* = 9.1 Hz, 1H), 5.22 – 5.10 (m, 1H), 2.40 (hept, *J* = 7.7 Hz, 1H), 2.10 – 1.95 (m, 2H), 1.91 – 1.70 (m, 4H), 1.69 – 1.56 (m, 2H), 1.44 (s, 9H); **$^{13}$C NMR** (100 MHz, $CDCl_3$) δ 160.9, 155.2, 142.6, 140.1, 127.8, 118.6, 79.34, 52.3, 42.8, 32.8, 28.7, 28.4 (3C), 28.4, 18.6; **HRMS** (ESI$^+$) Calculated for $C_{16}H_{22}Br_2N_2O_2$ $[M+H]^+$ 435.0106; found 435.0106.

**Synthesis of tert-butyl (*R*)-(2-cyclobutyl-1-(3,6-dibromopyridin-2-yl)ethyl)carbamate (29bR)**

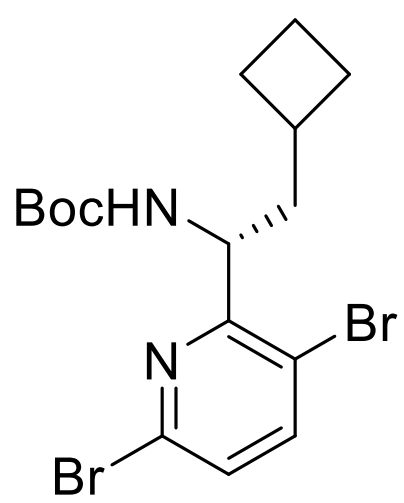

The title compound was prepared according to general procedure E using diastereomer **22bR** (450.0 mg, 1.03 mmol, 1.0 equiv.). Product was purified by CombiFlash on silica gel (0-10% Hexane/EtOAc) (437.6 mg, 98% yield, light yellow oil). **$^{1}$H NMR** (400 MHz, $CDCl_3$) δ 7.64 (d, *J* = 8.3 Hz, 1H), 7.22 (d, *J* = 8.2 Hz, 1H), 5.47 (d, *J* = 8.7 Hz, 1H), 5.20 – 5.09 (m, 1H), 2.40 (hept, *J* = 7.7 Hz, 1H), 2.10 – 1.96 (m, 2H), 1.90 – 1.71 (m, 4H), 1.68 – 1.57 (m, 2H), 1.44 (s, 9H); **$^{13}$C NMR** (100 MHz, $CDCl_3$) δ 160.9, 155.2, 142.6, 140.1, 127.8, 118.6, 79.4, 52.3, 42.8, 32.8, 28.8, 28.5, 28.4 (3C), 18.6; **HRMS** (ESI$^+$) Calculated for $C_{16}H_{22}Br_2N_2O_2$ $[M+H]^+$ 435.0106; found 435.0104.

**Synthesis of *tert*-butyl (*S*)-(2-cyclopentyl-1-(3,6-dibromopyridin-2-yl)ethyl)carbamate (29cS)**

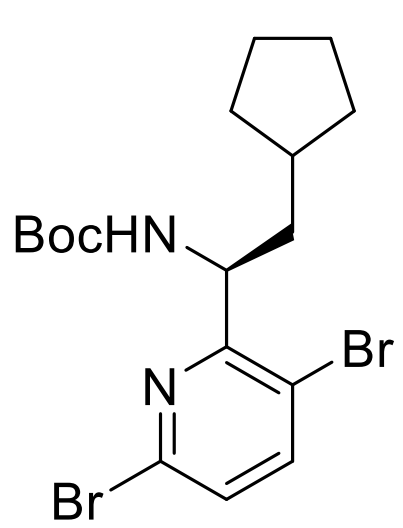

The title compound was prepared according to general procedure E using diastereomer **22cS** (1.59 g, 3.57 mmol, 1.0 equiv.). Product was purified by CombiFlash on silica gel (0-10% Hexane/EtOAc) (1.48 g, 93% yield, light yellow oil). **$^{1}$H NMR** (400 MHz, $CDCl_3$) δ 7.64 (d, *J* = 8.3 Hz, 1H), 7.22 (d, *J* = 8.3 Hz, 1H), 5.45 (d, *J* = 8.4 Hz, 1H), 5.22 (q, *J* = 7.2 Hz, 1H), 1.96 – 1.84 (m, 2H), 1.82 – 1.71 (m, 1H), 1.68 – 1.62 (m, 2H), 1.62 – 1.56 (m, 2H), 1.56 – 1.48 (m, 2H), 1.44 (s, 9H), 1.29 – 1.18 (m, 1H), 1.18 – 1.05 (m, 1H); **$^{13}$C NMR** (100 MHz, $CDCl_3$) δ 161.4, 155.4, 142.6, 140.1, 127.8, 118.6, 79.5, 53.01, 42.2, 36.8, 33.2, 32.4, 28.4, 25.2, 25.1; **HRMS** (APCI$^+$) Calculated for $C_{17}H_{24}Br_2N_2O_2$ $[M+H]^+$ 449.0262; found 449.0265.

**Synthesis of *tert*-butyl (*R*)-(2-cyclopentyl-1-(3,6-dibromopyridin-2-yl)ethyl)carbamate (29cR)**

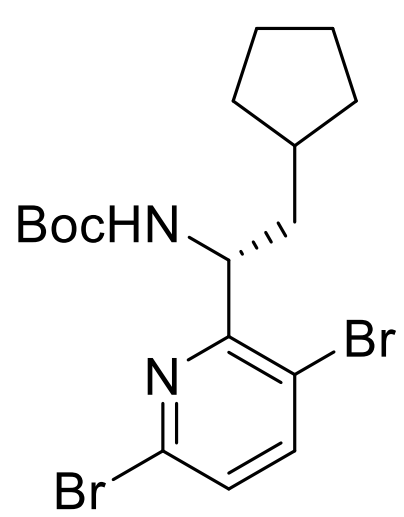


The title compound was prepared according to general procedure E using diastereomer **22cR** (810.0 mg, 1.80 mmol, 1.0 equiv.). Product was purified by CombiFlash on silica gel (0-10% Hexane/EtOAc) (1.14 g, 96% yield, light yellow oil). **$^1$H NMR** (400 MHz, $CDCl_3$) δ 7.64 (d, *J* = 8.2 Hz, 1H), 7.22 (d, *J* = 8.3 Hz, 1H), 5.47 (d, *J* = 9.3 Hz, 1H), 5.22 (q, *J* = 7.0 Hz, 1H), 1.99 – 1.84 (m, 2H), 1.84 – 1.72 (m, 1H), 1.69 – 1.62 (m, 2H), 1.61 – 1.56 (m, 2H), 1.56 – 1.48 (m, 2H), 1.44 (s, 9H), 1.28 – 1.18 (m, 1H), 1.17 – 1.07 (m, 1H); **$^{13}$C NMR** (100 MHz, $CDCl_3$) δ 161.4, 155.3, 142.6, 140.1, 127.8, 118.6, 79.4, 53.0, 42.2, 36.7, 33.2, 32.3, 28.4, 25.2, 25.1; **HRMS** (APCI$^+$) Calculated for $C_{17}H_{24}Br_2N_2O_2$ $[M+H]^+$ 449.0262; found 449.0262.

**Synthesis of tert-butyl (*S*)-(2-cyclohexyl-1-(3,6-dibromopyridin-2-yl)ethyl)carbamate (29dS)**

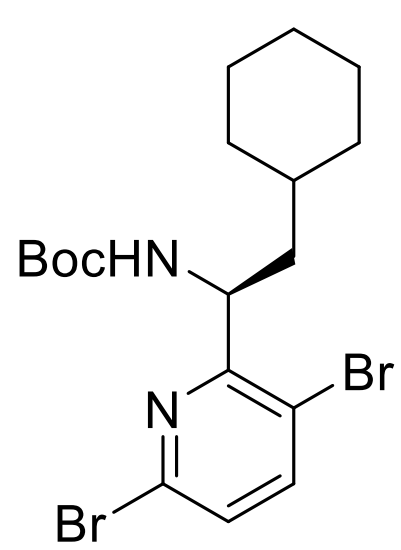


The title compound was prepared according to general procedure E using diastereomer **22dS** (487.1 mg, 1.04 mmol, 1.0 equiv.). Product was purified by CombiFlash on silica gel (0-10% Hexane/EtOAc) (300 mg, 63%, light yellow solid). **$^1$H NMR** (400 MHz, $CDCl_3$) δ 7.62 (d, *J* = 8.3 Hz, 1H), 7.21 (d, *J* = 8.3 Hz, 1H), 5.40 (d, *J* = 9.3 Hz, 1H), 5.32 – 5.22 (m, 1H), 2.03 (d, *J* = 12.6 Hz, 1H), 1.74 – 1.59 (m, 4H), 1.50 – 1.46 (m, 3H), 1.43 (s, 9H), 1.29 – 1.12 (m, 3H), 1.03 – 0.88 (m, 2H); **$^{13}$C NMR** (100 MHz, $CDCl_3$) δ 161.7, 155.5, 142.6, 140.1, 127.7, 118.4, 79.5, 51.3, 43.6, 34.3, 34.2, 32.3, 28.4, 26.5, 26.4, 26.1; **HRMS** (APCI$^+$) Calculated for $C_{18}H_{26}Br_2N_2O_2$ $[M+H]^+$ 463.0419; found 463.0336.

**Synthesis of tert-butyl (*R*)-(2-cyclohexyl-1-(3,6-dibromopyridin-2-yl)ethyl)carbamate (29dR)**

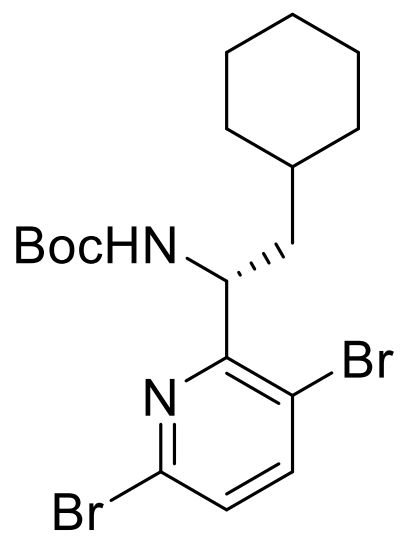


The title compound was prepared according to general procedure E using diastereomer **22dR** (1.05 g, 2.26 mmol, 1.0 equiv.). Product was purified by CombiFlash on silica gel (0-10% Hexane/EtOAc), light yellow solid. **$^1$H NMR** (400 MHz, $CDCl_3$) δ 7.63 (d, *J* = 8.2 Hz, 1H), 7.22 (d, *J* = 8.3 Hz, 1H), 5.41 (d, *J* = 9.3 Hz, 1H), 5.33 – 5.23 (m, 1H), 2.07 – 1.98 (m, 1H), 1.77 – 1.61 (m, 4H), 1.56 – 1.44 (m, 3H), 1.44 (s, 9H), 1.31 – 1.07 (m, 3H), 1.03 – 0.86 (m, 2H); **$^{13}$C NMR** (100 MHz, $CDCl_3$) δ 161.7, 155.5, 142.6, 140.1, 127.7, 118.4, 79.4, 51.3, 43.6, 34.3, 34.2, 32.3, 28.4, 26.5, 26.3, 26.1; **HRMS** (ESI$^+$) Calculated for $C_{18}H_{26}Br_2N_2O_2$ $[M+H]^+$ 463.0419; found 463.0347.

**Synthesis of tert-butyl (S)-(1-(3-bromo-6-(3-methyl-3-(methylsulfonyl)but-1-yn-1-yl)pyridin-2-yl)-2-cyclopropylethyl)carbamate (30aS)**

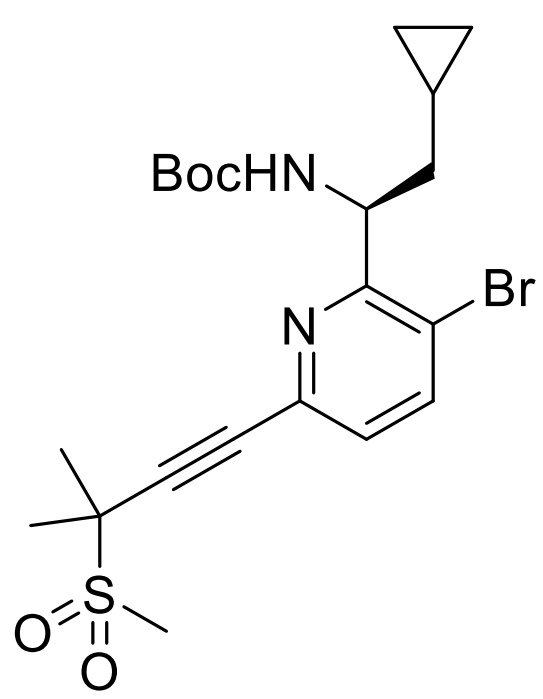


The title compound was prepared according to general procedure F using *N*-Boc-protected bis-bromopyridine **29aS** (1.40 g, 3.35 mmol, 1.0 equiv.). Product was purified by CombiFlash on silica gel (0-30% Hexane/EtOAc) (1.08 g, 67% yield, light yellow solid). **$^1$H NMR** (400 MHz, $CDCl_3$) δ 7.80 (d, *J* = 8.2 Hz, 1H), 7.20 (d, *J* = 8.2 Hz, 1H), 5.71 (d, *J* = 9.0 Hz, 1H), 5.43 – 5.32 (m, 1H), 3.11 (s, 3H), 1.78 (s, 6H), 1.67 – 1.58 (m, 2H), 1.46 (s, 9H), 0.78 – 0.66 (m, 1H), 0.46 – 0.34 (m, 2H), 0.08 – 0.00 (m, 1H), -0.01 – -0.09 (m, 1H); **$^{13}$C NMR** (100 MHz, $CDCl_3$) δ 159.9, 155.2, 140.6, 140.1, 127.1, 120.2, 87.5, 85.1, 79.4, 57.9, 53.9, 41.0, 35.2, 28.5, 22.7, 22.7, 7.6, 4.8, 4.3; **HRMS** (ESI$^+$) Calculated for $C_{21}H_{29}BrN_2O_4S$ $[M+H]^+$ 485.1110; found 485.1114.

**Synthesis of tert-butyl (R)-(1-(3-bromo-6-(3-methyl-3-(methylsulfonyl)but-1-yn-1-yl)pyridin-2-yl)-2-cyclopropylethyl)carbamate (30aR)**

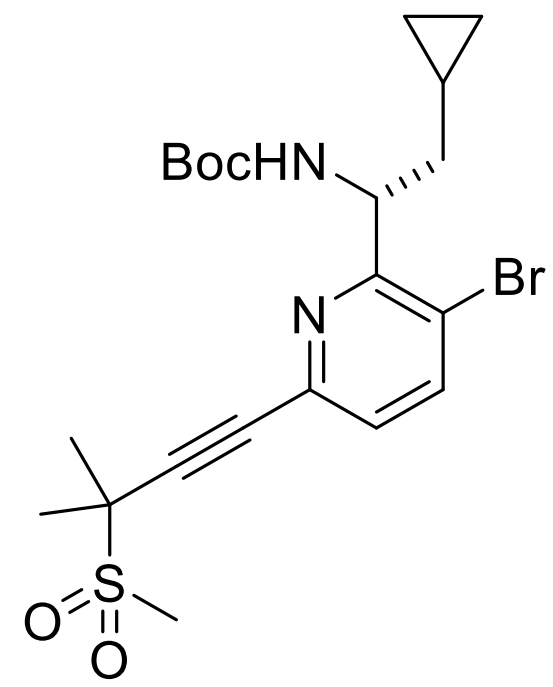


The title compound was prepared according to general procedure F using *N*-Boc-protected bis-bromopyridine **29aR** (1.74 g, 4.19 mmol, 1.0 equiv.). Product was purified by CombiFlash on silica gel (0-30% Hexane/EtOAc) (1.17 g, 84% yield, light yellow solid). **$^{1}$H NMR** (400 MHz, $CDCl_3$) δ 7.80 (d, *J* = 8.2 Hz, 1H), 7.20 (d, *J* = 8.2 Hz, 1H), 5.71 (d, *J* = 9.5 Hz, 1H), 5.43 – 5.33 (m, 1H), 3.11 (s, 3H), 1.78 (s, 6H), 1.64 (t, *J* = 6.8 Hz, 2H), 1.46 (s, 9H), 0.76 – 0.68 (m, 1H), 0.45 – 0.33 (m, 2H), 0.08 – 0.00 (m, 1H), -0.02 – -0.10 (m, 1H); **$^{13}$C NMR** (100 MHz, $CDCl_3$) δ 159.9, 155.2, 140.6, 140.1, 127.1, 120.2, 87.5, 85.1, 79.4, 57.9, 53.9, 41.0, 35.2, 28.5, 22.7, 22.7, 7.6, 4.8, 4.3; **HRMS** ($ESI^+$) Calculated for $C_{21}H_{29}BrN_2O_4S$ $[M+H]^+$ 485.1110; found 485.1102.

**Synthesis of tert-butyl (*S*)-(1-(3-bromo-6-(3-methyl-3-(methylsulfonyl)but-1-yn-1-yl)pyridin-2-yl)-2-cyclobutylethyl)carbamate (30bS)**

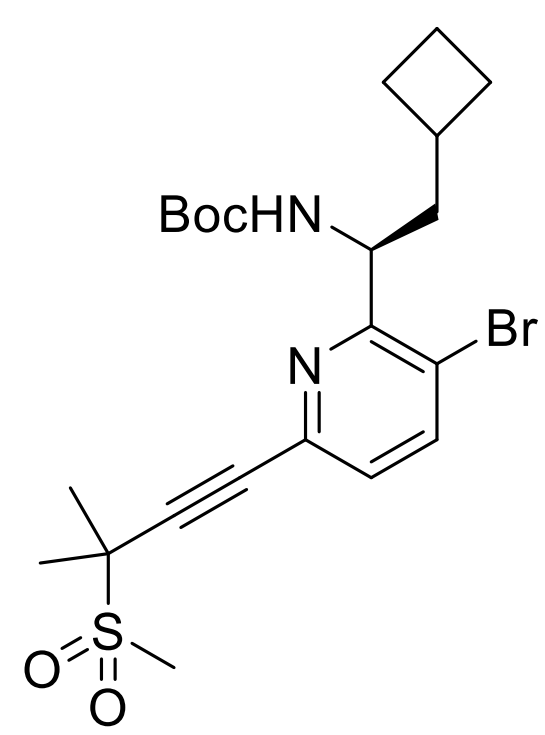


The title compound was prepared according to general procedure F using *N*-Boc-protected bis-bromopyridine **29bS** (278.4 mg, 0.64 mmol, 1.0 equiv.). Product was purified by CombiFlash on silica gel (0-30% Hexane/EtOAc) (111.8 mg, 35% yield, light yellow solid). **$^{1}$H NMR** (400 MHz, $CDCl_3$) δ 7.79 (d, *J* = 8.2 Hz, 1H), 7.19 (d, *J* = 8.2 Hz, 1H), 5.60 (d, *J* = 8.9 Hz, 1H), 5.25 – 5.15 (m, 1H), 3.11 (s, 3H), 2.40 (hept, *J* = 7.7 Hz, 1H), 2.14 – 1.93 (m, 2H), 1.93 – 1.70 (m, 4H), 1.78 (s, 6H), 1.69 – 1.56 (m, 2H), 1.45 (s, 9H); **$^{13}$C NMR** (100 MHz, $CDCl_3$) δ 160.1, 155.2, 140.6, 140.1, 127.1, 119.9, 87.5, 85.1, 79.3, 57.9, 52.4, 43.1, 35.2, 32.9, 28.7, 28.4 (3C), 28.4, 22.7 (2C), 18.6; **HRMS** ($ESI^+$) Calculated for $C_{22}H_{31}BrN_2O_4S$ $[M+H]^+$ 499.1266; found 499.1264.

**Synthesis of tert-butyl (*R*)-(1-(3-bromo-6-(3-methyl-3-(methylsulfonyl)but-1-yn-1-yl)pyridin-2-yl)-2-cyclobutylethyl)carbamate (30bR)**

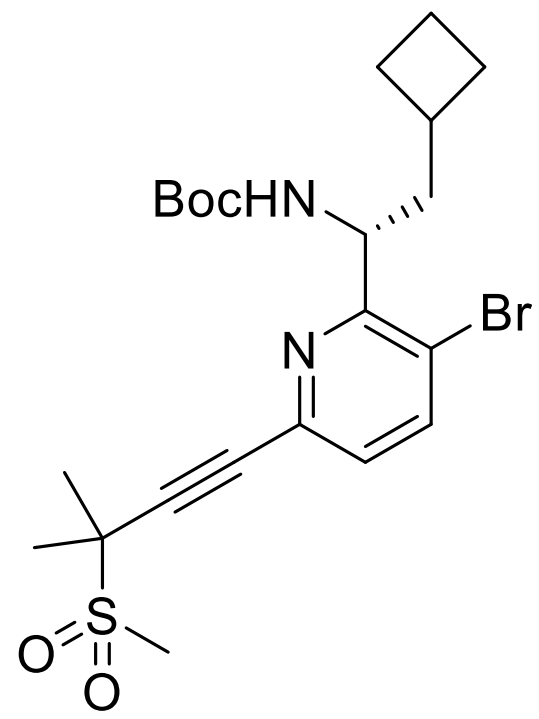


The title compound was prepared according to general procedure F using *N*-Boc-protected bis-bromopyridine **29bR** (437.6 mg, 1.01 mmol, 1.0 equiv.). Product was purified by CombiFlash on silica gel (0-30% Hexane/EtOAc) (334.9 mg, 66% yield, light yellow solid). **$^1$H NMR** (400 MHz, $CDCl_3$) δ 7.79 (d, *J* = 8.2 Hz, 1H), 7.19 (d, *J* = 8.2 Hz, 1H), 5.60 (d, *J* = 8.9 Hz, 1H), 5.25 – 5.15 (m, 1H), 3.11 (s, 3H), 2.40 (hept, *J* = 7.9 Hz, 1H), 2.12 – 1.95 (m, 2H), 1.91 – 1.70 (m, 4H), 1.78 (s, 6H), 1.68 – 1.56 (m, 2H), 1.45 (s, 9H); **$^{13}$C NMR** (100 MHz, $CDCl_3$) δ 160.1, 155.2, 140.6, 140.1, 127.1, 119.9, 87.5, 85.1, 79.3, 57.9, 52.4, 43.1, 35.2, 32.9, 28.7, 28.4 (3C), 28.4, 22.7 (2C), 18.6; **HRMS** (ESI$^+$) Calculated for $C_{22}H_{31}BrN_2O_4S$ $[M+H]^+$ 499.1266; found 499.1263.

**Synthesis of *tert*-butyl (*S*)-(1-(3-bromo-6-(3-methyl-3-(methylsulfonyl)but-1-yn-1-yl)pyridin-2-yl)-2-cyclopentylethyl)carbamate (30cS)**

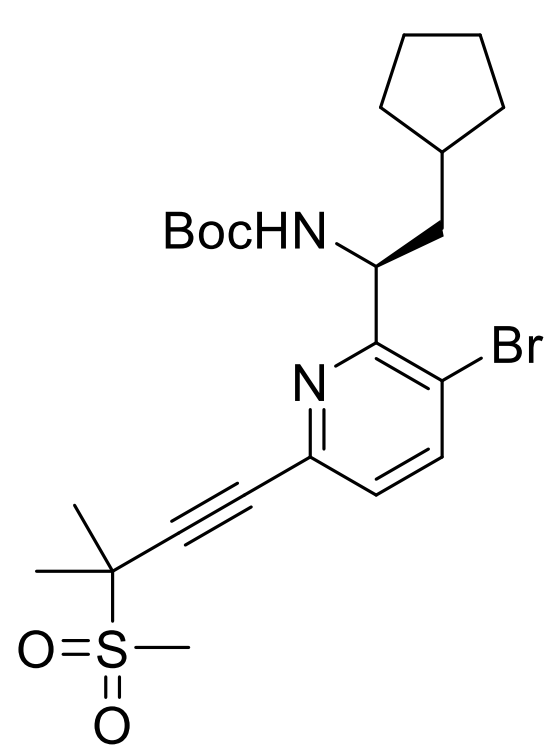


The title compound was prepared according to general procedure F using *N*-Boc-protected bis-bromopyridine **29cS** (0.50 g, 1.12 mmol, 1.0 equiv.). Product was purified by CombiFlash on silica gel (0-30% Hexane/EtOAc) (471.2 mg, 82% yield, light yellow solid). **$^1$H NMR** (400 MHz, $CDCl_3$) δ 7.79 (d, *J* = 8.2 Hz, 1H), 7.18 (d, *J* = 8.2 Hz, 1H), 5.57 (d, *J* = 8.7 Hz, 1H), 5.32 – 5.22 (m, 1H), 3.11 (s, 3H), 1.98 – 1.85 (m, 2H), 1.82 – 1.72 (m, 1H), 1.78 (s, 6H), 1.68 – 1.57 (m, 4H), 1.56 – 1.48 (m, 2H), 1.44 (s, 9H), 1.30 – 1.19 (m, 1H), 1.17 – 1.06 (m, 1H); **$^{13}$C NMR** (100 MHz, $CDCl_3$) δ 160.6, 155.4, 140.6, 140.12, 127.0, 119.8, 87.4, 85.1, 79.3, 57.9, 532 42.5, 36.8, 35.2, 33.2, 32.4, 28.4, 25.2, 25.1, 22.7; **HRMS** (ESI$^+$) Calculated for $C_{23}H_{33}BrN_2O_4S$ $[M+H]^+$ 513.1423; found 513.1417.

**Synthesis of *tert*-butyl (*R*)-(1-(3-bromo-6-(3-methyl-3-(methylsulfonyl)but-1-yn-1-yl)pyridin-2-yl)-2-cyclopentylethyl)carbamate (30cR)**

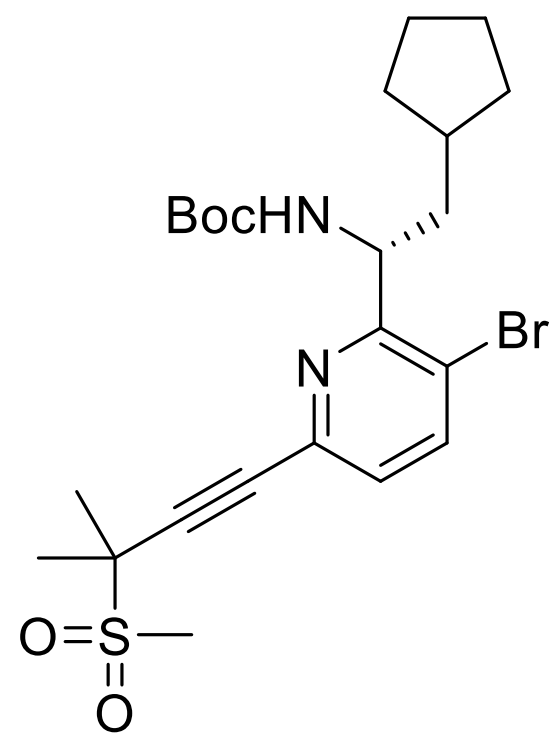


The title compound was prepared according to general procedure F using *N*-Boc-protected bis-bromopyridine **29cR** (1.13 g, 2.54 mmol, 1.0 equiv.). Product was purified by CombiFlash on silica gel (0-30% Hexane/EtOAc) (650.2 mg, 50% yield, light yellow solid). **$^1$H NMR** (400 MHz, $CDCl_3$) δ 7.79 (d, $J$ = 8.2 Hz, 1H), 7.19 (d, $J$ = 8.2 Hz, 1H), 5.58 (d, $J$ = 9.3 Hz, 1H), 5.32 – 5.22 (m, 1H), 3.11 (s, 3H), 1.97 – 1.85 (m, 2H), 1.83 – 1.73 (m, 1H), 1.78 (s, 6H), 1.68 – 1.57 (m, 4H), 1.56 – 1.48 (m, 2H), 1.44 (s, 9H), 1.30 – 1.19 (m, 1H), 1.17 – 1.06 (m, 1H); **$^{13}$C NMR** (100 MHz, $CDCl_3$) δ 160.6, 155.4, 140.6, 140.2, 127.0, 119.8, 87.4, 85.1, 79.3, 57.9, 53.2, 42.5, 36.8, 35.2, 33.2, 32.4, 28.4, 25.2, 25.1, 22.7, 22.7; **HRMS** ($ESI^+$) Calculated for $C_{23}H_{33}BrN_2O_4S$ $[M+H]^+$ 513.1423 ; found 513.1424.

**Synthesis of tert-butyl (*S*)-(1-(3-bromo-6-(3-methyl-3-(methylsulfonyl)but-1-yn-1-yl)pyridin-2-yl)-2-cyclohexylethyl)carbamate (30dS)**

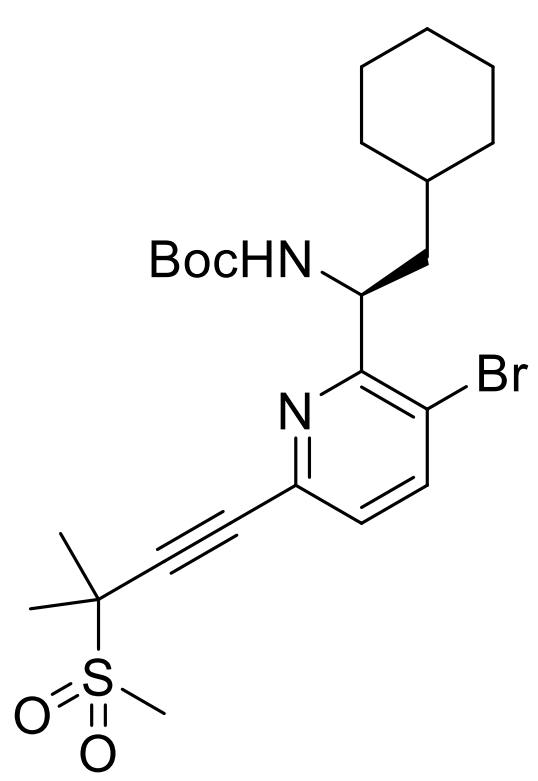


The title compound was prepared according to general procedure F using *N*-Boc-protected bis-bromopyridine **29dS** (300.0 mg, 0.65 mmol, 1.0 equiv.). Product was purified by CombiFlash on silica gel (0-30% Hexane/EtOAc) (229.7 mg, 67%, light yellow solid). **$^1$H NMR** (400 MHz, $CDCl_3$) δ 7.71 (d, $J$ = 8.2 Hz, 1H), 7.11 (d, $J$ = 8.2 Hz, 1H), 5.46 (d, $J$ = 9.4 Hz, 1H), 5.33 – 5.24 (m, 1H), 3.03 (s, 3H), 2.04 – 1.96 (m, 1H), 1.71 (s, 6H), 1.66 – 1.51 (m, 3H), 1.49 – 1.29 (m, 4H), 1.37 (s, 9H), 1.24 – 1.03 (m, 3H), 0.97 – 0.83 (m, 2H); **$^{13}$C NMR** (100 MHz, $CDCl_3$) δ 160.9, 155.4, 140.6, 140.2, 127.0, 119.7, 87.4, 85.1, 79.3, 57.9, 51.4, 44.0, 35.2, 34.4, 34.2, 32.4, 28.4, 26.5, 26.4, 26.2, 22.7; **HRMS** ($APCI^+$) Calculated for $C_{24}H_{35}BrN_2O_4S$ $[M+H]^+$ 527.1579; found 527.1579.

**Synthesis of tert-butyl (*R*)-(1-(3-bromo-6-(3-methyl-3-(methylsulfonyl)but-1-yn-1-yl)pyridin-2-yl)-2-cyclohexylethyl)carbamate (30dR)**

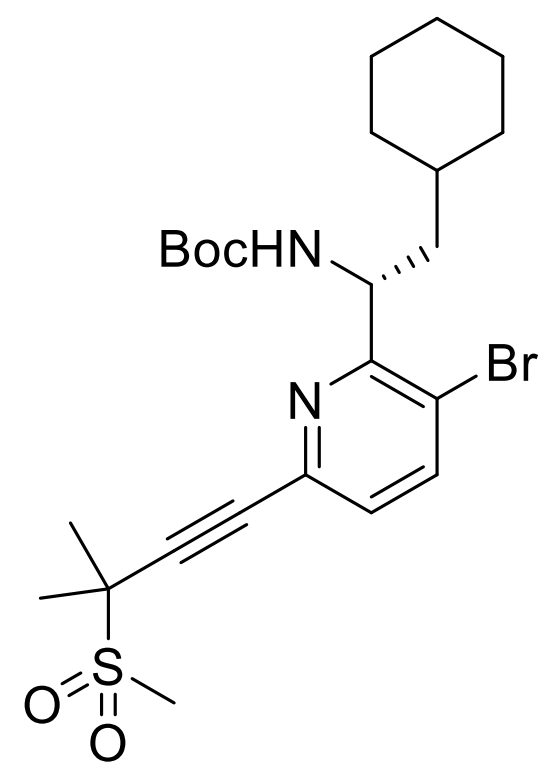


The title compound was prepared according to general procedure F using *N*-Boc-protected bis-bromopyridine **29dR** (1.04 g, 2.268 mmol). Product was purified by CombiFlash on silica gel (0-30% Hexane/EtOAc) (754.4 mg, 63% over two steps, light yellow solid). **$^1$H NMR** (400 MHz, $CDCl_3$) δ 7.78 (d, *J* = 8.2 Hz, 1H), 7.18 (d, *J* = 8.2 Hz, 1H), 5.53 (d, *J* = 9.4 Hz, 1H), 5.40 – 5.30 (m, 1H), 3.11 (s, 3H), 2.11 – 2.04 (m, 1H), 1.78 (s, 6H), 1.73 – 1.59 (m, 3H), 1.56 – 1.35 (m, 4H), 1.45 (s, 9H), 1.31 – 1.10 (m, 3H), 1.04 – 0.89 (m, 2H); **$^{13}$C NMR** (100 MHz, $CDCl_3$) δ 160.9, 155.4, 140.6, 140.2, 127.0, 119.6, 87.4, 85.1, 79.3, 57.9, 51.4, 44.0, 35.2, 34.4, 34.2, 32.3, 28.4, 26.5, 26.4, 26.2, 22.7; **HRMS** (APCI$^+$) Calculated for $C_{24}H_{35}BrN_2O_4S$ $[M+H]^+$ 527.1579; found 527.1579.

**Synthesis of 3-methyl-3-(methylsulfonyl)but-1-yne (32)**

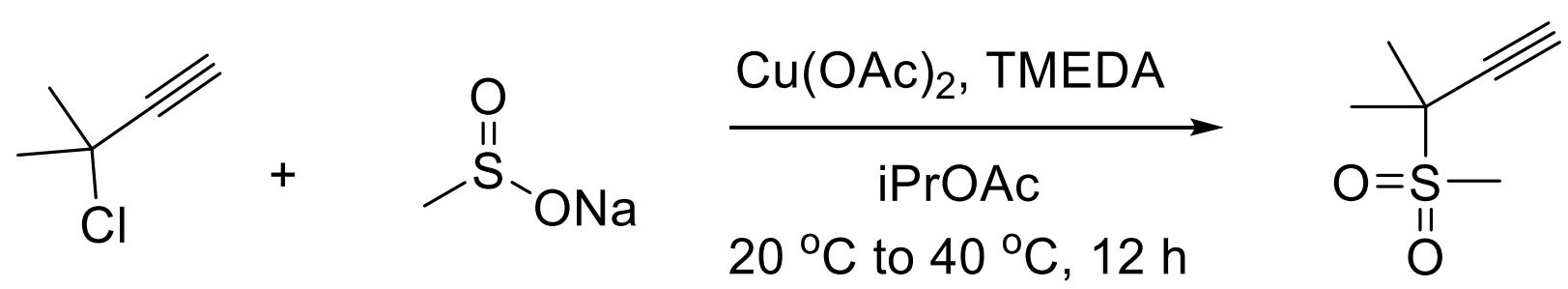


A round-bottom flask was charged with sodium methansulfinate (6.99 g, 68.6 mmol, 1.4 equiv.), $Cu(OAc)_2$ (443.4 mg, 2.45 mmol, 5 mol%), TMEDA (0.732 mL, 4.9 mmol, 10 mol%), and isopropyl acetate (20 mL). The reaction mixture was cooled to 20 ºC, and 3-Chloro-3-methyl-1-butyne (5.0 g, 49.0 mmol, 1.0 equiv.) was added slowly. The resulting mixture was stirred at 40 ºC for 12 hours. Upon completion of the reaction, the mixture was cooled to 20 ºC and treated with 5% aqueous $H_2SO_4$ (20 mL). The mixture was extracted with ethyl acetate (3 x 30 mL), and the combined organic layers were dried over anhydrous $Na_2SO_4$ and concentrated under reduced pressure. The mixture was recrystallized with ethyl acetate/pentane at 0 ºC to afford the desired product **32** as a white powder (4.28 g, 58% yield). The $^1$H NMR is matched with previously reported literature. **$^1$H NMR** (400 MHz, $CDCl_3$) δ 3.04 (s, 3H), 2.58 (s, 1H), 1.67 (s, 6H).

## 1.3 Synthesis of subunit C

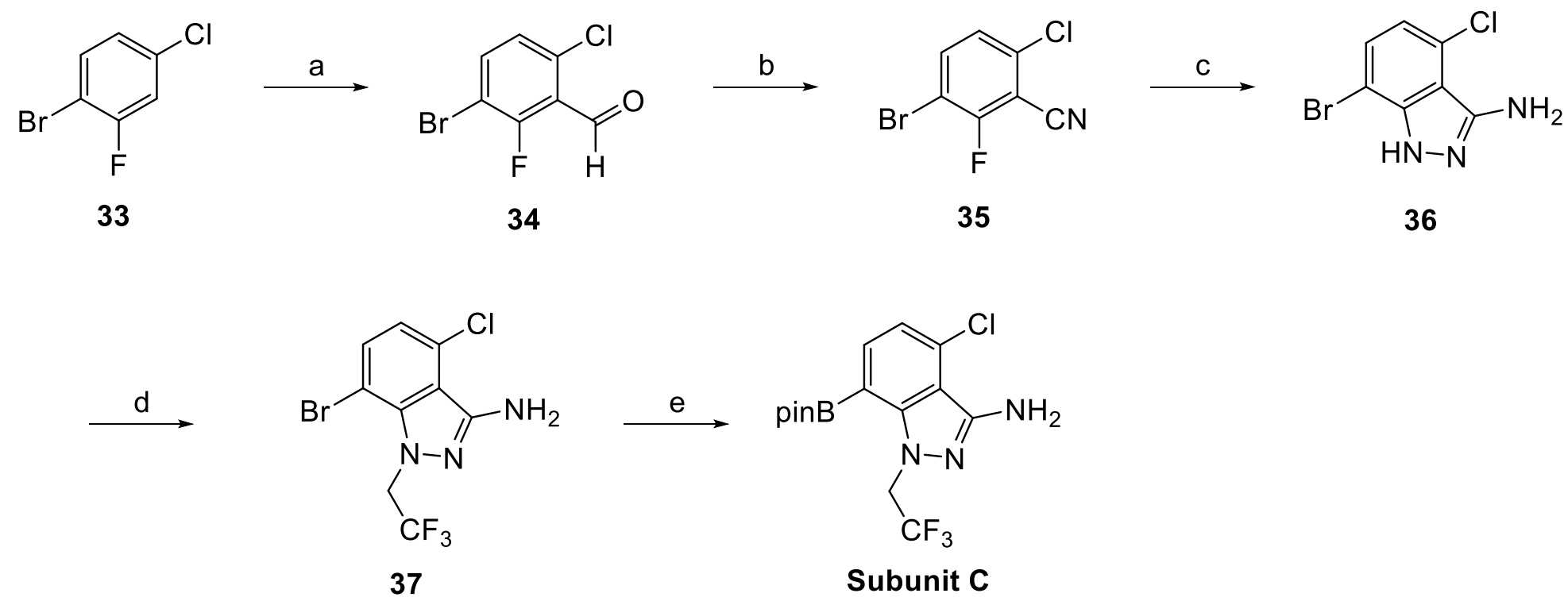

Reagents and conditions: (a) LDA, DMF, THF, -78 ℃, 99%; (b) $NH_2OSO_3H$, $H_2O$, 50°C, overnight, 80%; (c) $H_2NNH_2{\bullet}H_2O$, EtOH, 80 ℃, overnight, 27%; (d) 2,2,2-Trifluoroethyl trifluoromethanesulfonate, $Cs_2CO_3$, DMF, 65°C, overnight, 56%; (e) $Bpin_2$, $Pd(PPh_3)Cl_2$, KOAc, 1,4-dioxane, 110 ℃, 46%.

This synthesis follow an already described procedure.[2]

**3-bromo-6-chloro-2-fluorobenzaldehyde (34)**

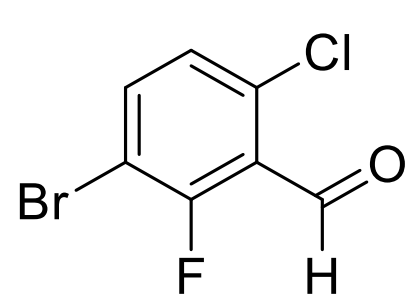

To a solution of 1-bromo-4-chloro-2-fluorobenzene **33** (5.27 g, 25.2 mmol) in 60 mL of dry THF at -78°C was added over 15 min a 2M solution of LDA in THF (16.4 mL, 32.7 mmol, 1.3 eq). The reaction mixture was allowed to warm to -20°C and was stirred for 1 hour. The reaction was cooled again to -78°C and was slowly charged with DMF (5.0 mL, 65 mmol, 2.6 eq). After 10 min at -78°C, the reaction mixture was allowed to slowly warm up to 0°C and was stirred for 45 min. The reaction was quenched via slow addition of ice-cold water (60 mL), diluted with EtOAc (60 mL) and stirred for 15 more min at room temperature. The organic layer was washed with water (2 times 30 mL), 1N HCl (40 mL), then a 15% NaCl solution (40 mL). The separated organic phase was dried ($Na_2SO_4$), filtered and concentrated in vacuo. The resultant crude of **34** (5.95 g, 25.1 mmol, 99%) was obtained as a white-orange solid, directly used in the next step without any further purification. **$^1$H-NMR** (400 MHz, $CDCl_3$) δ ppm: 10.41 (d, $J$ = 1.2 Hz, 1H), 7.69 (dd, $J$ = 8.6, 7.0

Hz, 1H), 7.20 (dd, $J$ = 8.6, 1.6 Hz, 1H); **$^{13}$C-NMR** (100 MHz, $CDCl_3$) δ ppm: 186.10 (d, $J$ = 2.3 Hz), 159.67 (d, $J$ = 265.0 Hz), 138.18 (d, $J$ = 2.4 Hz), 136.12 (d, $J$ = 2.8 Hz), 127.63 (d, $J$ = 4.6 Hz), 122.80 (d, $J$ = 10.7 Hz), 109.33 (d, $J$ = 21.6 Hz).

**3-bromo-6-chloro-2-fluorobenzonitrile (35)**

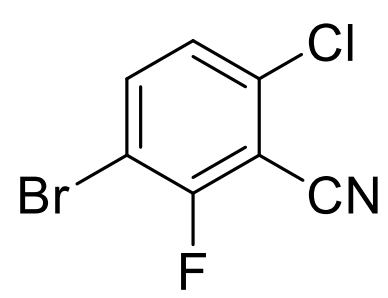


To a suspension of the aldehyde **34** (5.95 g, 25.1 mmol) in 100 mL of water was added at room temperature O-sulfinic acid hydroxylamine (4.96 g, 43.9 mmol, 1.75 eq). The reaction mixture was heated to 50°C and stirred overnight (18h). The mixture was cooled to room temperature and stirred for 1 hour. The solid obtained was isolated by filtration and washed with water. The wet solid was taken up with EtOAc. This organic phase was washed with water, and the aq layer was extracted two times with EtOAc. All the organic layers were combined, dried over $Na_2SO_4$, filtered and concentrated in vacuo to yield as a white-beige solid the compound **35** (4.69 g, 20.0 mmol, 80%). **$^{1}$H-NMR** (400 MHz, $CDCl_3$) δ ppm: 7.76 (ddd, $J$ = 8.8, 7.2, 1.7 Hz, 1H), 7.29 – 7.24 (m, 1H); **$^{13}$C-NMR** (100 MHz, $CDCl_3$) δ ppm: 160.51 (d, $J$ = 261.9 Hz), 138.20 (d, $J$ = 1.8 Hz), 137.07 (d, $J$ = 1.2 Hz), 126.66 (d, $J$ = 4.3 Hz), 110.53, 108.30 (d, $J$ = 20.1 Hz), 104.80 (d, $J$ = 18.9 Hz).

**7-bromo-4-chloro-1H-indazol-3-amine (36)**

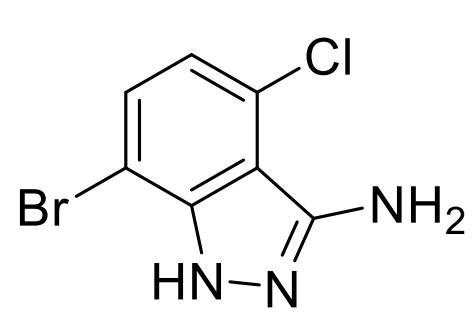


To a solution of **35** (4.68 g, 20.0 mmol, 1 eq) in anhydrous ethanol (70 mL) was added dropwise $NH_2NH_2 \cdot H_2O$ (3.9 mL, 80.0 mmol, 4 eq), while stirring at room temperature. The reaction mixture was heated to 90°C and stirred overnight (16 h), then cooled to room temperature and concentrated in vacuo to remove most of the ethanol. The crude was dissolved in EtOAc, forming a precipitate. The white solid was removed by filtration, and the organic phase was washed with water, then brine, before being dried over $Na_2SO_4$, filtered and concentrated in vacuo to give a white-orange solid, purified by Si gel flash chromatography (98:2 to 96:4 DCM:MeOH). 1.35 g (5.48 mmol,

28%) of product **36** was obtained. **$^{1}$H-NMR** (400 MHz, DMSO-*d6*) δ ppm: 12.22 (s, 1H), 7.43 (d, *J* = 7.9 Hz, 1H), 6.86 (d, *J* = 7.9 Hz, 1H), 5.32 (br s, 2H); **$^{13}$C-NMR** (100 MHz, DMSO-*d6*) δ ppm: 149.1, 141.1, 129.5, 125.2, 119.1, 111.9, 101.0; **HRMS** (APCI$^{+}$) (m/z): $[M+H]^+$ calculated 245.9429 (and 247.9408), found 245.9428 (and 247.9407).

**7-bromo-4-chloro-1-(2,2,2-trifluoroethyl)-1H-indazol-3-amine (37)**

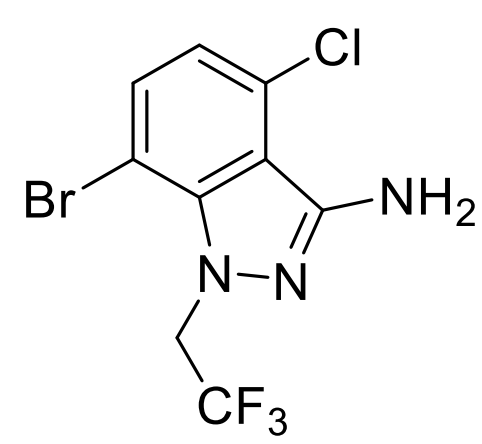


In 30 mL of DMF was added **36** (1.34 g, 5.44 mmol) with $Cs_2CO_3$ (3.54 g, 10.9 mmol, 2 eq). The resulting mixture was stirred for 30 min prior to the addition of 2,2,2-Trifluoroethyl trifluoromethanesulfonate (1.41 mL, 9.79 mmol, 1.8 eq). The reaction mixture was stirred overnight at 65°C, then cooled to room temperature and quenched with 300 mL of ice-cold water. The aq phase was extracted 3 times with EtOAc. All organic layers were combined, washed with water then brine, dried over $Na_2SO_4$, filtered and concentrated in vacuo. The crude obtained was purified by Si gel flash chromatography (90:10 Hexane:EtOAc) to give 1.01 g (3.07 mmol, 56%) of product **37**, directly used for the next step. **$^{1}$H-NMR** (400 MHz, $CDCl_3$) δ ppm: 7.44 (d, *J* = 8.0 Hz, 1H), 6.89 (d, *J* = 8.0 Hz, 1H), 5.21 (q, *J* = 8.0 Hz, 2H), 4.65 (br s, 3H)

**4-chloro-1-(2,2,2-trifluoroethyl)-7-(4,4,5,5-tetramethyl-1,3,2-dioxaborolan-2-yl)-1H-indazol-3-amine (subunit C)**

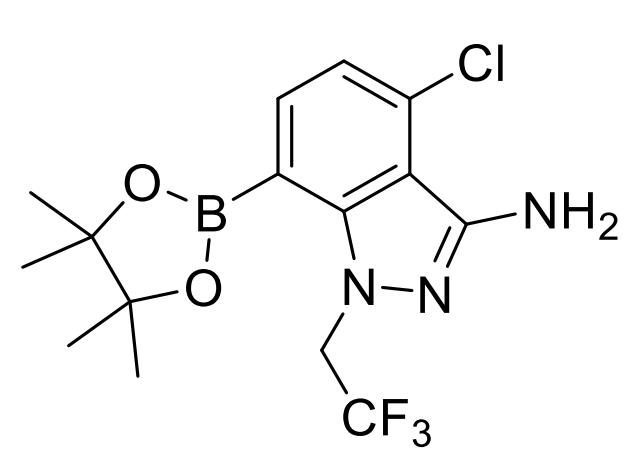


To a solution of **37** (1.00 g, 3.04 mmol, 1 eq) in 1,4-dioxane (30 mL) was added KOAc (1.193 g, 12.16 mmol, 4 eq), bis(pinacolato)diboron (2.316 g, 9.12 mmol, 3 eq) and $Pd(PPh_3)_2Cl_2$ (0.213 g, 0.304 mmol, 0.1 eq). The reaction mixture was stirred overnight at 110°C, then was cooled down

to room temperature and quenched by slow addition of ice-cold water. 2 extraction with EtOAc were done, all the organic layers were combined, washed with water then brine, dried over $Na_2SO_4$, filtered and concentrated in vacuo. The crude was purified by Si gel flash chromatography (90:10 to 80:20 Hexane:EtOAc) to give 0.53 g (1.41 mmol, 46%) of **subunit C**. **$^1$H-NMR** (400 MHz, $CDCl_3$) δ ppm: 7.83 (dd, $J$ = 7.7, 1.9 Hz, 1H), 7.00 (dd, $J$ = 7.6, 1.9 Hz, 1H), 5.41 (qd, $J$ = 8.6, 1.9 Hz, 2H), 4.59 (s, 2H), 1.38 (s, 12H); **$^{13}$C-NMR** (100 MHz, $CDCl_3$) δ ppm: 148.6, 145.9, 138.7, 131.0, 124.48 (q, $J$ = 282.0 Hz), 119.6, 113.0, 84.7, 83.6, 50.74 (q, $J$ = 33.8 Hz), 25.1, 24.8; **HRMS** (ESI$^+$) (m/z): $[M+H]^+$ calculated 376.1206, found 376.1150. Data match with previous literature.

## 1.4 Coupling of subunits A, B, and C

Synthesis of the *(S)*-isomers :

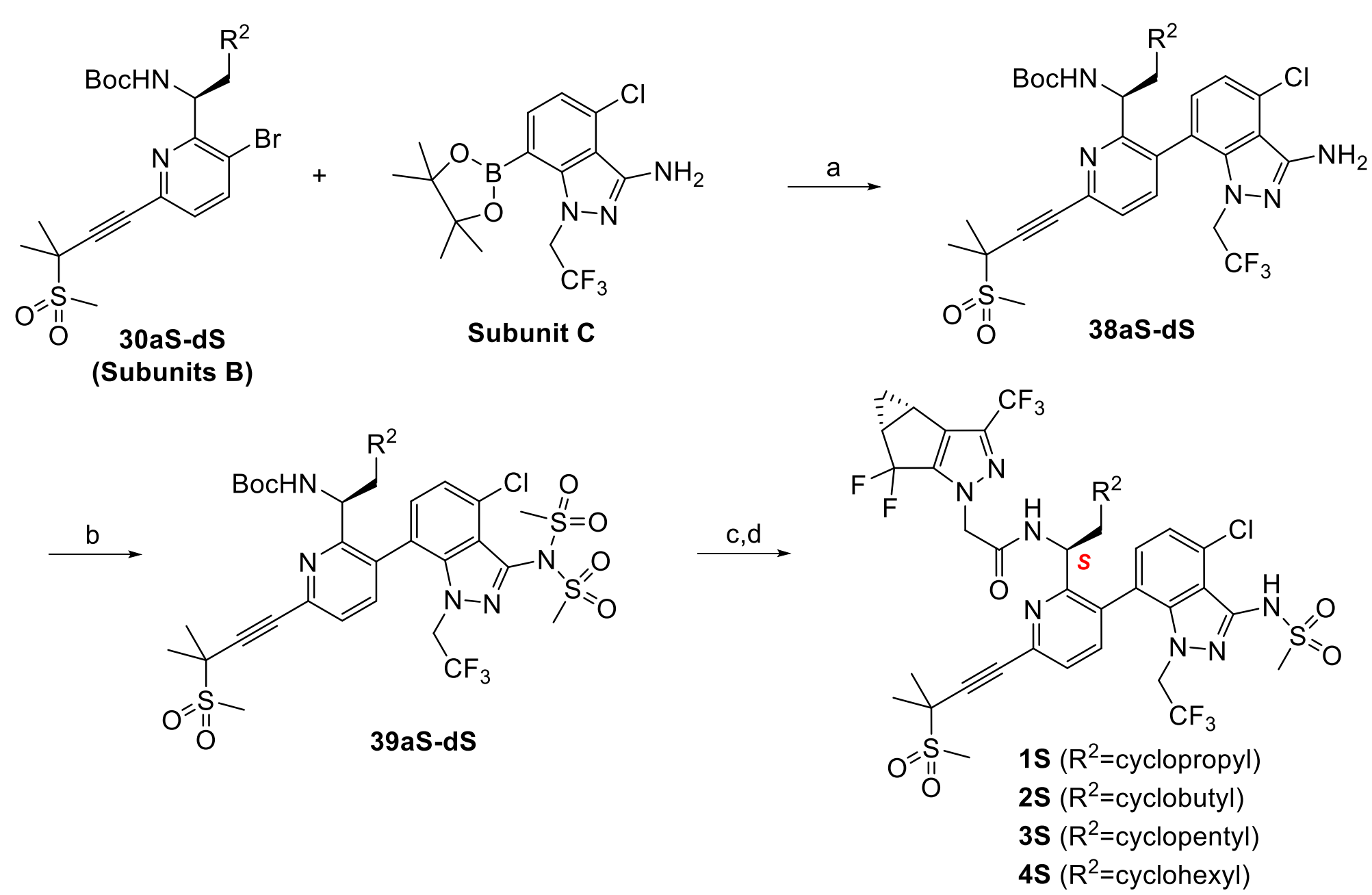


Synthesis of the *(R)*-isomers :

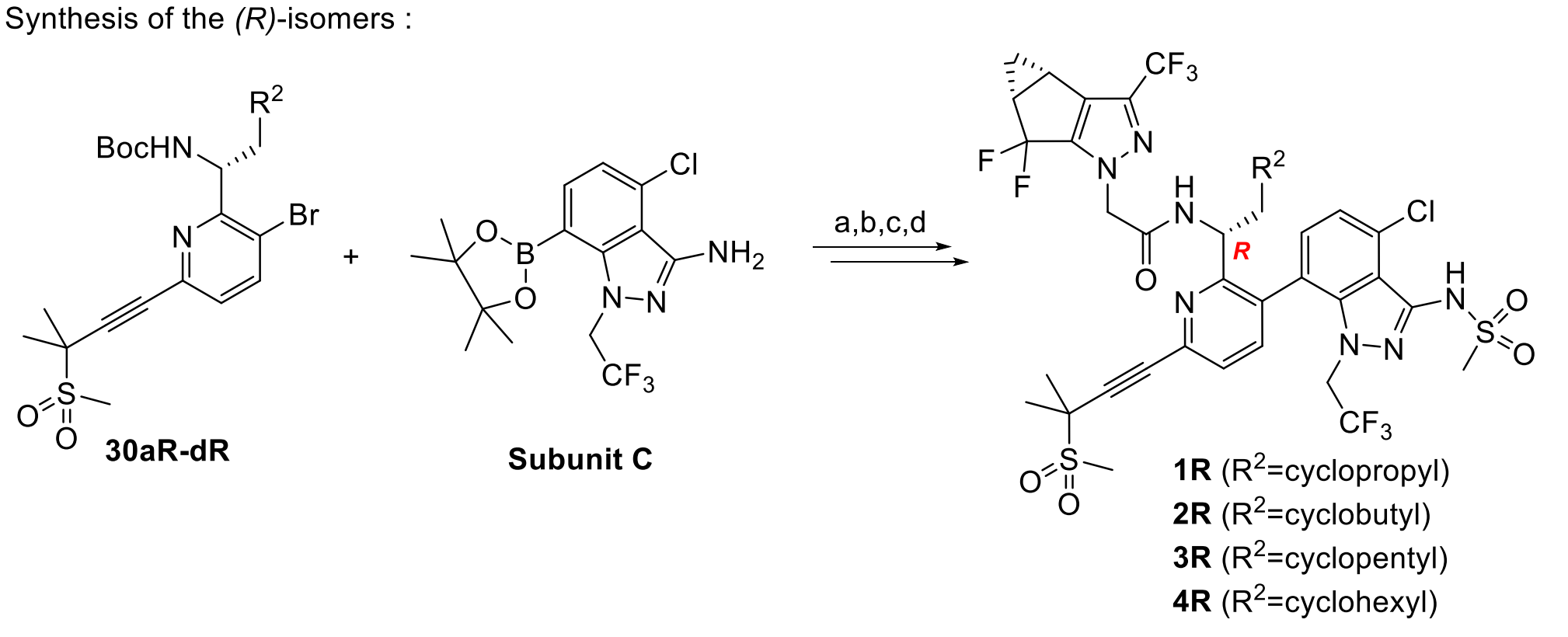

Reagents and conditions, all yields with R as cyclopentyl: (a) Pd(dppf)$Cl_2$, $K_2CO_3$, 1,4-dioxane/$H_2O$, 80 °C, 16h, 67%; (b) methane sulfonylchloride, $Et_3N$, DCM, rt, 2h, 58%; (c) TFA, DCM, rt, 2h, 99%; (d) Subunit A, HATU, DIPEA, DMF, rt, overnight, 37%.

**General procedure G**

**Subunit B** (1 eq), **subunit C** (1.3 eq), [1,1'bis(diphenylphosphino)-ferrocene]-dichloropalladium(II) (0.1 eq), and potassium carbonate (6 eq) were charged in a round-bottom flask and placed under argon. 1,4 Dioxane (10 mL/mmol) and water (1.5 mL/mmol) were added. The mixture was heated to 110 °C and allowed to stir overnight. Upon completion (TLC analysis), the reaction mixture was cooled to room temperature and partitioned between EtOAc and water. The aqueous layer was removed, and the organic layer was concentrated under vacuum. The resulting residue was purified by flash column chromatography to provide the title compound **38aS-dS, or 38aR-dR.**

**General procedure H**

**38aS-dS** (or **38aR-dR**) (1 eq) was dissolved in $CH_2Cl_2$ (15 mL/mmol) and triethylamine (0.35 eq) was then added. To the resulting mixture was added methanesulfonyl chloride (2.5 eq) with stirring at room temperature. The reaction mixture was stirred at room temperature for 1 h until the reaction was complete. Upon completion (TLC analysis), the reaction mixture was diluted with EtOAc (100 mL/mmol) and washed successively with saturated aqueous $NaHCO_3$, water, and brine. The organic layer was dried over $Na_2SO_4$, filtered, and concentrated. The residue was purified by flash column chromatography (30% EtOAc in hexanes) to afford the title compound **39aS-dS** (or **39aR-dR**).

**General procedure I**

**39aS-dS** (or **39aR-dR**) (1 eq) was charged in 10-mL round-bottom flask and a mixture of $CH_2Cl_2$/TFA (2:1, 15 mL/mmol) was added and stirred for 1 h. The mixture was then concentrated to afford the title compound as trifluoroacetic acid salt, which was used without purification. This crude was dissolved in DMF (10 mL/mmol) and charged with **subunit A** (1.2 eq),

diisopropylethylamine (2 eq) and HATU (1.2 eq). The reaction mixture was stirred at room temperature overnight (18 h), then concentrated in vacuo, diluted with water and EtOAc. The aqueous phase was extracted 2 times with EtOAc. All organic layers were combined, washed with water, brine, dried ($Na_2SO_4$), concentrated to obtain a residue that was diluted in EtOAc:DCM (1:1)

**Synthesis of tert-butyl (*S*)-(1-(3-(3-amino-4-chloro-1-(2,2,2-trifluoroethyl)-1H-indazol-7-yl)-6-(3-methyl-3-(methylsulfonyl)but-1-yn-1-yl)pyridin-2-yl)-2-cyclopropylethyl)carbamate (38aS)**

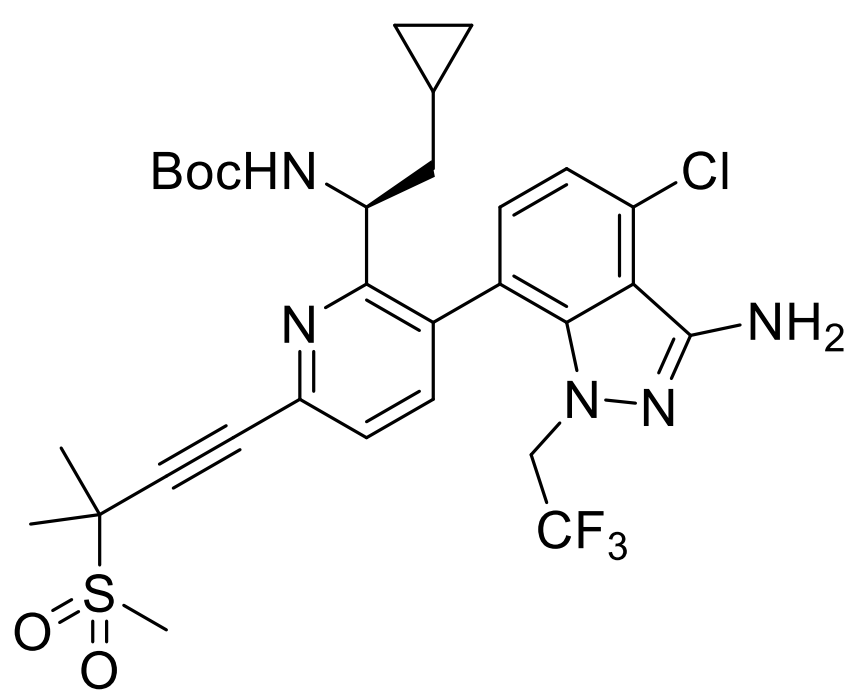


The title compound was prepared according to general procedure G using cyclopropyl subunit B **30aS** (316.0 mg, 0.65 mmol, 1.0 equiv.). Product was purified by CombiFlash on silica gel (0-40% Hexane/EtOAc) (190.8 mg, 44%, white solid). **$^1$H NMR** (400 MHz, $CDCl_3$) δ 7.52 (d, *J* = 8.2 Hz, 1H), 7.41 (d, *J* = 7.9 Hz, 1H), 6.99 (d, *J* = 7.7 Hz, 1H), 6.86 (d, *J* = 7.7 Hz, 1H), 5.55 (d, *J* = 9.4 Hz, 1H), 4.77 (s, 2H), 4.56 – 4.46 (m, 1H), 4.28 (dq, *J* = 16.3, 8.2 Hz, 1H), 3.95 (dq, *J* = 16.4, 8.2 Hz, 1H), 3.10 (s, 3H), 1.76 (s, 6H), 1.55 – 1.43 (m, 1H), 1.39 – 1.27 (m, 1H), 1.33 (s, 9H), 0.42 – 0.31 (m, 1H), 0.24 – 0.10 (m, 2H), -0.13 – -0.22 (m, 1H), -0.46 – -0.55 (m, 1H); **$^{13}$C NMR** (100 MHz, $CDCl_3$) δ 160.6, 155.0, 148.8, 142.1, 140.2, 138.8, 130.9, 130.7, 127.8, 125.9, 123.1 (q, *J* = 280.0 Hz), 119.7, 118.8, 113.9, 87.4, 85.4, 79.3, 57.9, 52.8, 50.2 (q, *J* = 34.0 Hz), 41.6, 35.2, 28.3, 22.7, 7.7, 4.7, 4.2; **$^{19}$F NMR** (376 MHz, $CDCl_3$) δ -70.26 (minor atropisomer not included); **HRMS** (APCI$^+$) Calculated for $C_{30}H_{35}ClF_3N_5O_4S$ $[M+H]^+$ 654.2124; found 654.2127.

**tert-butyl (R)-(1-(3-(3-amino-4-chloro-1-(2,2,2-trifluoroethyl)-1H-indazol-7-yl)-6-(3-methyl-3-(methylsulfonyl)but-1-yn-1-yl)pyridin-2-yl)-2-cyclopropylethyl)carbamate (38aR)**

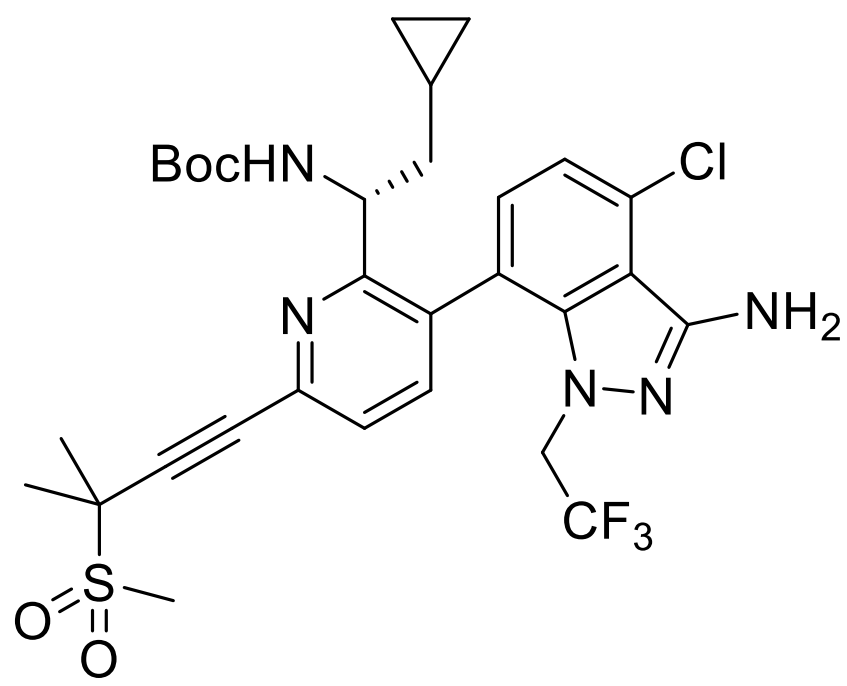


The title compound was prepared according to general procedure G using **30aR** (358 mg, 0.74 mmol, 1.0 equiv.). Product was purified by CombiFlash on silica gel (0-40% Hexane/EtOAc) (138 mg, 29%, white solid).

**$^1$H-NMR** (400 MHz, $CDCl_3$) δ 7.56 (d, *J* = 8.5 Hz, 1H), 7.44 (d, *J* = 8.0 Hz, 1H), 7.15 – 7.02 (m, 1H), 6.90 (d, *J* = 7.6 Hz, 1H), 5.51 (d, *J* = 9.5 Hz, 1H), 4.84 – 4.62 (m, 2H), 4.62 – 4.46 (m, 1H), 4.32 (dq, *J* = 16.2, 8.1 Hz, 1H), 4.05 – 3.83 (m, 1H), 3.15 (br s, 3H), 1.80 (s, 6H), 1.57 – 1.20 (m, 12H), 0.51 – 0.12 (m, 2H), -0.04 – -0.23 (m, 1H), -0.35 – -0.53 (m, 1H). **$^{13}$C NMR** (100 MHz, CDCl3) δ 160.7, 155.2, 148.9, 142.3, 140.4, 138.9, 130.9, 128.0, 127.8, 126.1, 119.9, 118.9, 114.2, 87.6, 85.7, 79.6, 58.1, 52.9, 50.35 (q, *J* = 34.5 Hz), 41.9, 35.4, 28.5, 22.9, 7.8, 4.8, 4.3.

**Synthesis of tert-butyl (*S*)-(1-(3-(4-chloro-3-(*N*-(methylsulfonyl)methylsulfonamido)-1-(2,2,2-trifluoroethyl)-1H-indazol-7-yl)-6-(3-methyl-3-(methylsulfonyl)but-1-yn-1-yl)pyridin-2-yl)-2-cyclopropylethyl)carbamate (39aS)**

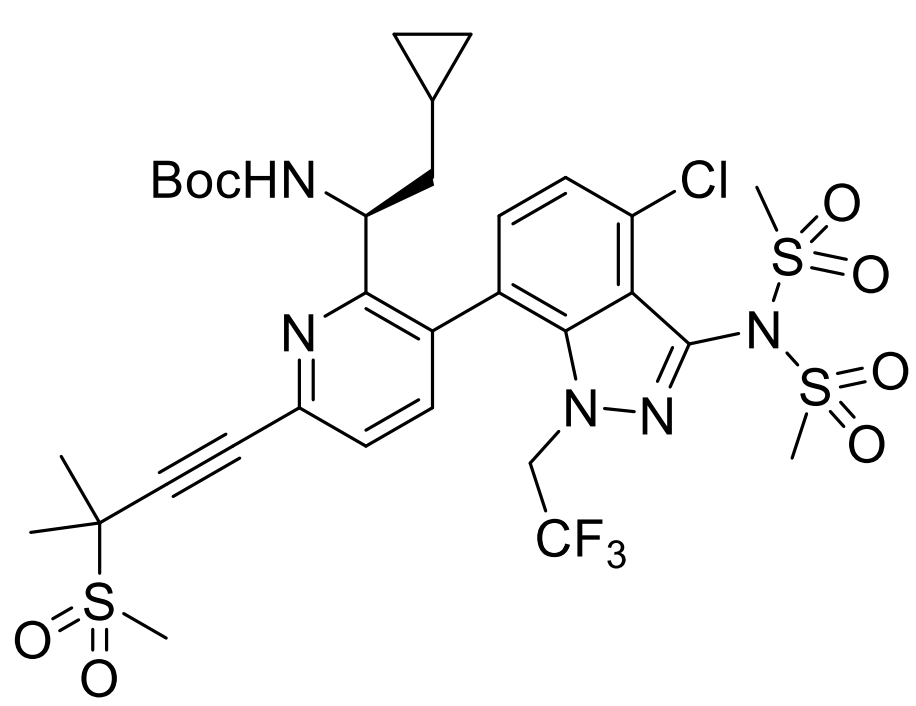


The title compound was prepared according to general procedure H using compound **38aS** (190.8 mg, 0.29 mmol, 1.0 equiv.). Product was purified by CombiFlash on silica gel (0-35% Hexane/EtOAc) (174.9 mg, 84%, white solid). **$^1$H NMR** (400 MHz, $CDCl_3$) δ 7.61 (d, *J* = 8.9 Hz, 1H), 7.50 (d, *J* = 7.9 Hz, 1H), 7.35 (d, *J* = 7.9 Hz, 1H), 7.10 (d, *J* = 7.6 Hz, 1H), 5.37 (d, *J* = 9.2 Hz, 1H), 4.64 (dq, *J* = 16.2, 8.2 Hz, 1H), 4.54 – 4.40 (m, 1H), 4.34 (dq, *J* = 15.9, 8.0 Hz, 1H), 3.65 (s, 3H), 3.58 (s, 3H), 3.17 (s, 3H), 1.83 (s, 6H), 1.65 – 1.50 (m, 1H), 1.44 – 1.24 (m, 1H), 1.37 (s, 9H), 0.46 – 0.35 (m, 1H), 0.34 – 0.26 (m, 1H), 0.24 – 0.14 (m, 1H), -0.05 – -0.16 (m, 1H), -0.45 – -0.55 (m, 1H); **$^{13}$C NMR** (100 MHz, $CDCl_3$) δ 161.0, 155.2, 142.6, 140.6, 138.9, 135.5, 131.4, 129.9, 126.7, 126.0, 123.7, 122.7 (q, *J* = 280.0 Hz), 120.2, 120.1, 88.1, 85.3, 79.6, 58.1, 53.0, 50.2 (q, *J* = 36.0 Hz), 42.9, 42.4, 41.1,

35.4, 28.4, 22.8, 7.8, 4.8, 4.3; **$^{19}$F NMR** (376 MHz, $CDCl_3$) δ -70.09 (minor atropisomer not included); **HRMS** ($ESI^+$) Calculated for $C_{32}H_{39}ClF_3N_5O_8S_3$ $[M+H]^+$ 810.1680; found 810.1680.

**tert-butyl (R)-(1-(3-(4-chloro-3-(N-(methylsulfonyl)methylsulfonamido)-1-(2,2,2-trifluoroethyl)-1H-indazol-7-yl)-6-(3-methyl-3-(methylsulfonyl)but-1-yn-1-yl)pyridin-2-yl)-2-cyclopropylethyl)carbamate (39aR)**

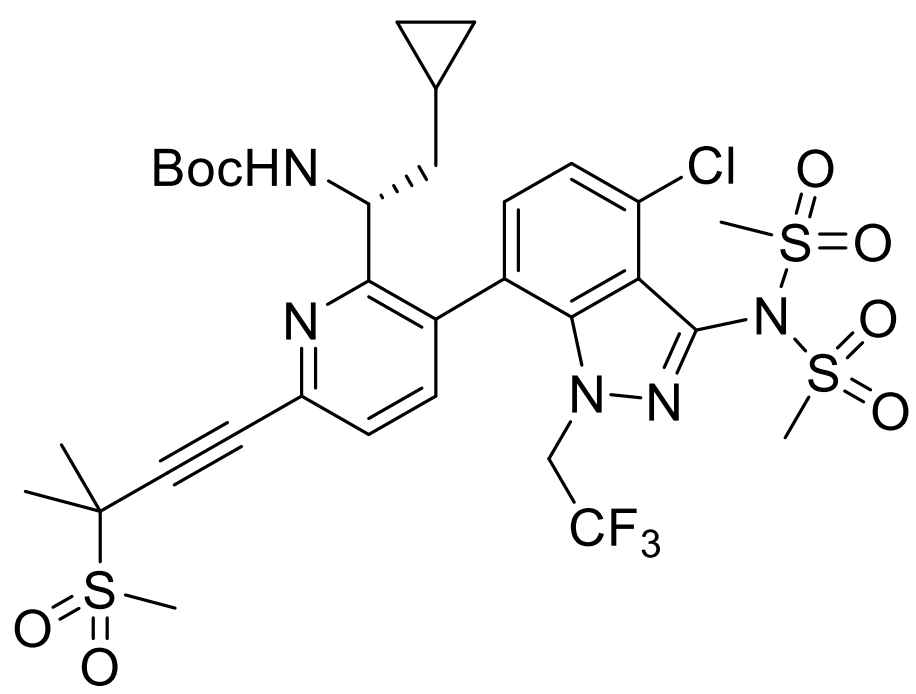


The title compound was prepared according to general procedure H using compound **38aR** (741 mg, 113 mmol, 1.0 equiv.). Product was purified by CombiFlash on silica gel (0-35% Hexane/EtOAc) (699 mg, 76%, white solid). Observation of the two atropoisomer on NMR in a 9:1 ratio

**$^1$H-NMR** (400 MHz, $CDCl_3$) δ ppm: 7.58 (d, *J* = 7.9 Hz, 1H), 7.47 (d, *J* = 7.9 Hz, 1H), 7.33 (d, *J* = 7.5 Hz, 1H), 7.08 (d, *J* = 7.7 Hz, 1H), 5.30 (d, *J* = 9.2 Hz, 0.9H), 4.77 – 4.69 (m, 0.1H), 4.62 (dq, *J* = 16.2, 8.1 Hz, 0.9H), 4.54 – 4.39 (m, 1H), 4.32 (dq, *J* = 15.9, 7.9 Hz, 0.9H), 4.25 – 4.16 (m, 0.1H), 3.64 (s, 3H), 3.57 (s, 4H), 3.15 (s, 3H), 1.82 (s, 6H), 1.61 – 1.48 (m, 1H), 1.44 – 1.23 (m, 10H), 0.48 – 0.34 (m, 1H), 0.33 – 0.14 (m, 2H), -0.07 – -0.22 (m, 1H), -0.39 (d, *J* = 4.8 Hz, 0.1H), -0.50 (d, *J* = 8.1 Hz, 0.9H); **$^{13}$C-NMR** (100 MHz, $CDCl_3$) δ ppm: 161.1, 155.2, 142.8, 140.7, 138.8, 135.6, 131.4, 129.9, 126.8, 126.0, 123.7, 120.3, 120.2, 87.8, 85.5, 79.6, 53.1, 43.1, 42.5, 41.2, 35.4, 28.5, 22.9, 7.8, 4.9, 4.4 ; **$^{19}$F NMR** (376 MHz, $CDCl_3$) δ ppm: -69.45 (t, *J* = 7.7 Hz), -70.10 (t, *J* = 8.1 Hz, minor atropoisomer)

**Synthesis of N-((S)-1-(3-(4-chloro-3-(methylsulfonamido)-1-(2,2,2-trifluoroethyl)-1H-indazol-7-yl)-6-(3-methyl-3-(methylsulfonyl)but-1-yn-1-yl)pyridin-2-yl)-2-cyclopropylethyl)-2-((3bS,4aR)-5,5-difluoro-3-(trifluoromethyl)-3b,4,4a,5-tetrahydro-1H-cyclopropa[3,4]cyclopenta[1,2-c]pyrazol-1-yl)acetamide (1S)**

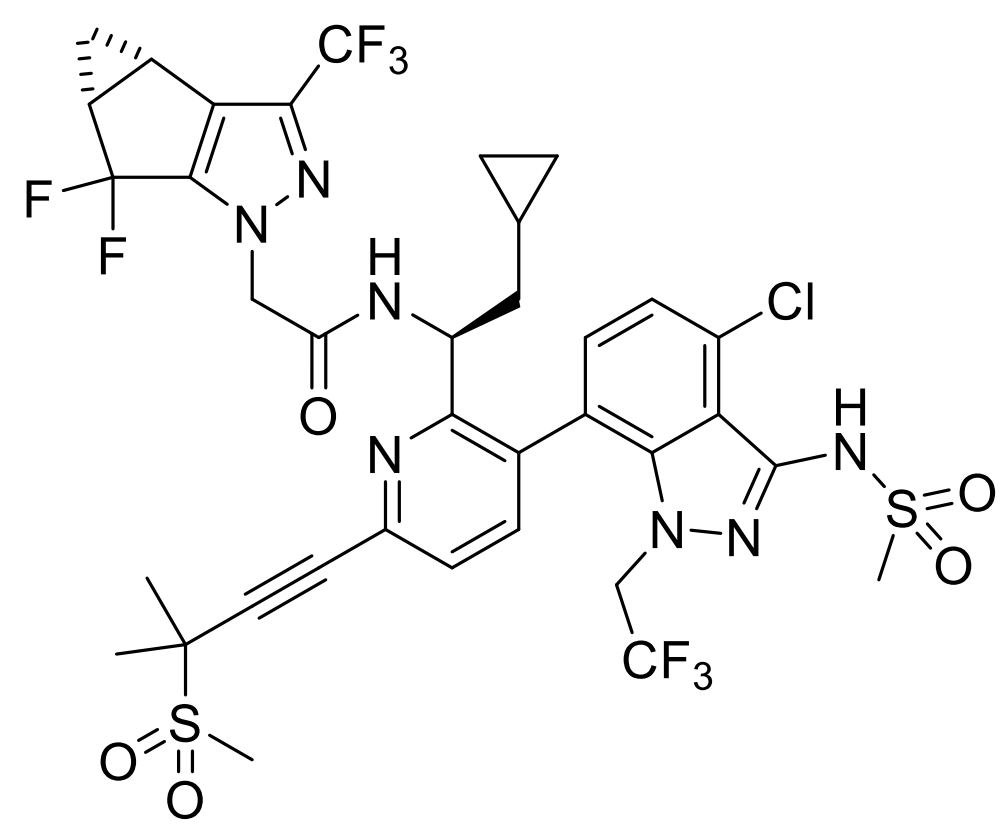

The title compound was prepared according to general procedure I, using compound **39aS** (174.9 mg, 0.24 mmol, 1.0 equiv.). Product was purified by CombiFlash on silica gel (0-55% Hexane/EtOAc) (126.1 mg, 59% over three steps, white solid, 99% pure, evaluated on HPLC-UV sig=250 nm). **$^{1}$H NMR** (400 MHz, $CDCl_3$) δ 7.60 (d, *J* = 7.9 Hz, 1H), 7.53 (d, *J* = 11.1 Hz, 1H), 7.51 (d, *J* = 8.0 Hz, 1H), 7.21 (d, *J* = 7.6 Hz, 1H), 7.03 (d, *J* = 7.7 Hz, 1H), 6.86 (d, *J* = 8.4 Hz, 1H), 4.77 (s, 2H), 4.76 – 4.71 (m, 1H), 4.52 (dq, *J* = 16.2, 8.1 Hz, 1H), 3.99 (dq, *J* = 16.1, 8.1 Hz, 1H), 3.41 (s, 3H), 3.16 (s, 3H), 2.57 – 2.42 (m, 2H), 1.83 (s, 6H), 1.59 – 1.48 (m, 1H), 1.47 – 1.37 (m, 2H), 1.17 – 1.09 (m, 1H), 0.42 – 0.32 (m, 1H), 0.31 – 0.25 (m, 1H), 0.26 – 0.15 (m, 1H), -0.12 – -0.21 (m, 1H), -0.39 – -0.47 (m, 1H); **$^{13}$C NMR** (100 MHz, $CDCl_3$) δ 164.0, 159.4, 143.6 (t, *J* = 29.3 Hz), 142.7, 139.9, 139.6, 138.9, 136.7 (q, *J* = 39.9 Hz), 132.89 – 132.53 (m), 131.4, 130.0, 126.9, 126.4, 122.8 (q, *J* = 279.6 Hz), 121.7, 120.5 (q, *J* = 267.3 Hz), 119.9 (t, *J* = 243.0 Hz), 119.1, 115.2, 88.1, 85.1, 58.0, 53.7, 51.9, 50.8 (q, *J* = 35.1 Hz), 41.5, 40.9, 35.2, 28.1 (dd, *J* = 34.6, 29.3 Hz), 23.4, 22.7, 12.2, 7.4, 4.7, 4.1; **$^{19}$F NMR** (376 MHz, $CDCl_3$) δ -61.57, -70.30, -80.94 (d, *J* = 256.0 Hz), -103.48 (d, *J* = 256.0 Hz) (minor atropisomer not included); **HRMS** ($ESI^+$) Calculated for $C_{36}H_{35}ClF_8N_7O_5S_2$ $[M+H]^+$ 896.1702; found 896.1695.

**N-((R)-1-(3-(4-chloro-3-(methylsulfonamido)-1-(2,2,2-trifluoroethyl)-1H-indazol-7-yl)-6-(3-methyl-3-(methylsulfonyl)but-1-yn-1-yl)pyridin-2-yl)-2-cyclopropylethyl)-2-((3bS,4aR)-5,5-difluoro-3-(trifluoromethyl)-3b,4,4a,5-tetrahydro-1H-cyclopropa[3,4]cyclopenta[1,2-c]pyrazol-1-yl)acetamide (1R)**

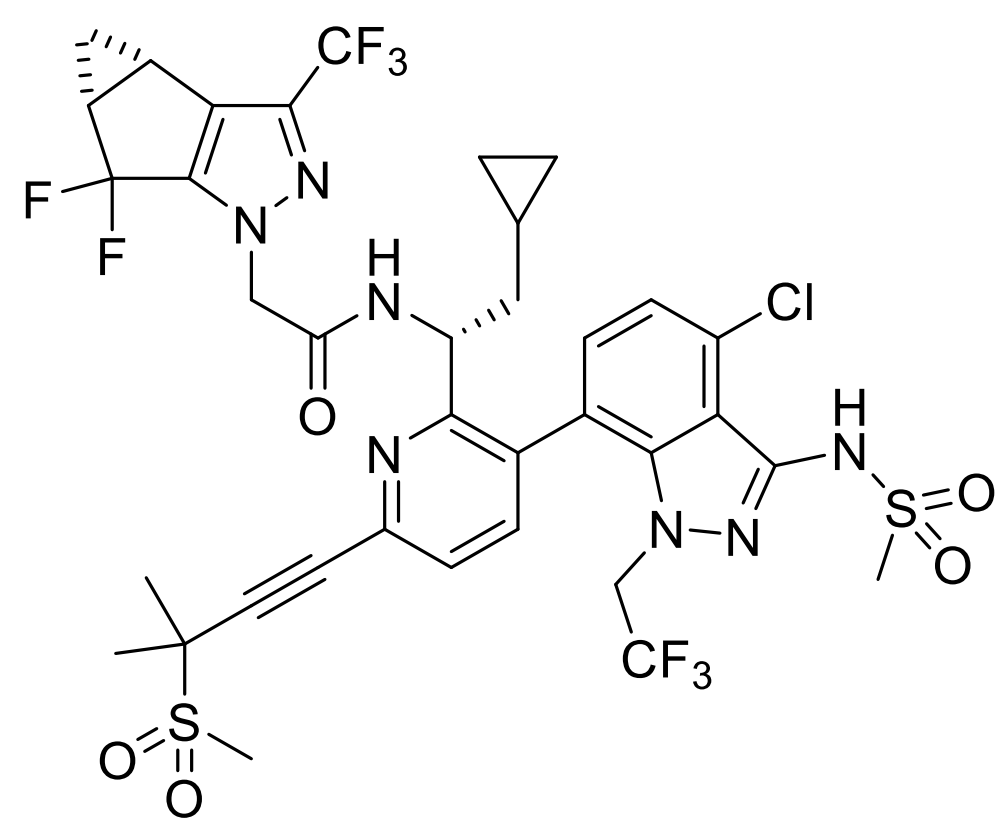

The title compound was prepared according to general procedure I, using compound **39aR** (532 mg, 0.666 mmol, 1.0 equiv.). Product was purified by CombiFlash on silica gel (0-55% Hexane/EtOAc) (235 mg, 39% over two steps, white solid, 98% pure, evaluated on HPLC-UV sig=250 nm).

**$^1$H-NMR** (400 MHz, CDCl3) δ ppm: 7.63 (s, 0.15H, minor atropoisomer), 7.58 (d, *J* = 7.9 Hz, 1H), 7.51 – 7.45 (m, 2H), 7.20 (d, *J* = 7.7 Hz, 1H), 7.02 (d, *J* = 7.7 Hz, 1H), 6.72 (d, *J* = 8.5 Hz, 0.85H, major atropoisomer), 6.58 (d, *J* = 8.7 Hz, 0.15H, minor atropoisomer), 5.07 (td, *J* = 8.8, 4.8 Hz, 0.15H), 4.81 – 4.68 (m, 2.9H, major atropoisomer), 4.52 (dq, *J* = 16.2, 8.1 Hz, 0.85H, major atropoisomer), 4.39 (dq, *J* = 15.5, 7.7 Hz, 0.15H, minor atropoisomer), 4.08 (dq, *J* = 16.1, 7.8 Hz, 0.15H, minor atropoisomer), 3.97 (dq, *J* = 16.0, 8.0 Hz, 0.85H, major atropoisomer), 3.41 (2 s, 3H), 3.15 (s, 3H), 2.53 – 2.43 (m, 2H), 1.82 (s, 6H), 1.50 (dq, J = 14.1, 7.0 Hz, 1H), 1.45 – 1.32 (m, 2H), 1.17 – 1.08 (m, 1H), 0.39 – 0.15 (m, 3H), -0.18 (dq, *J* = 9.2, 4.7 Hz, 1H), -0.31 – -0.38 (m, 0.15H, minor atropoisomer), -0.43 (dq, *J* = 9.6, 4.8 Hz, 0.85H, major atropoisomer); **$^{13}$C NMR** (100 MHz, CDCl3) δ 164.1, 159.5, 142.8, 140.1, 139.7, 139.1, 131.5, 130.1, 127.1, 126.5, 121.7, 119.2, 115.2, 88.3, 85.2, 58.1, 53.8, 51.9, 51.9, 41.7, 41.1, 35.3, 28.2 (dd, *J* = 34.5, 29.4 Hz), 23.5, 22.8, 22.8, 22.7, 12.4, 7.4, 4.8, 4.2; **$^{19}$F NMR** (376 MHz, $CDCl_3$) δ -61.53, -70.27, -81.15 (dd, *J* = 255.6, 13.4 Hz), -103.55 (dd, *J* = 256.1, 8.4 Hz); **HRMS** (ESI$^+$) Calculated for $C_{36}H_{35}ClF_8N_7O_5S_2$ $[M+H]^+$ 896.1702; found 896.1685.

**Synthesis of tert-butyl (S)-(1-(3-(3-amino-4-chloro-1-(2,2,2-trifluoroethyl)-1H-indazol-7-yl)-6-(3-methyl-3-(methylsulfonyl)but-1-yn-1-yl)pyridin-2-yl)-2-cyclobutylethyl)carbamate (38bS)**

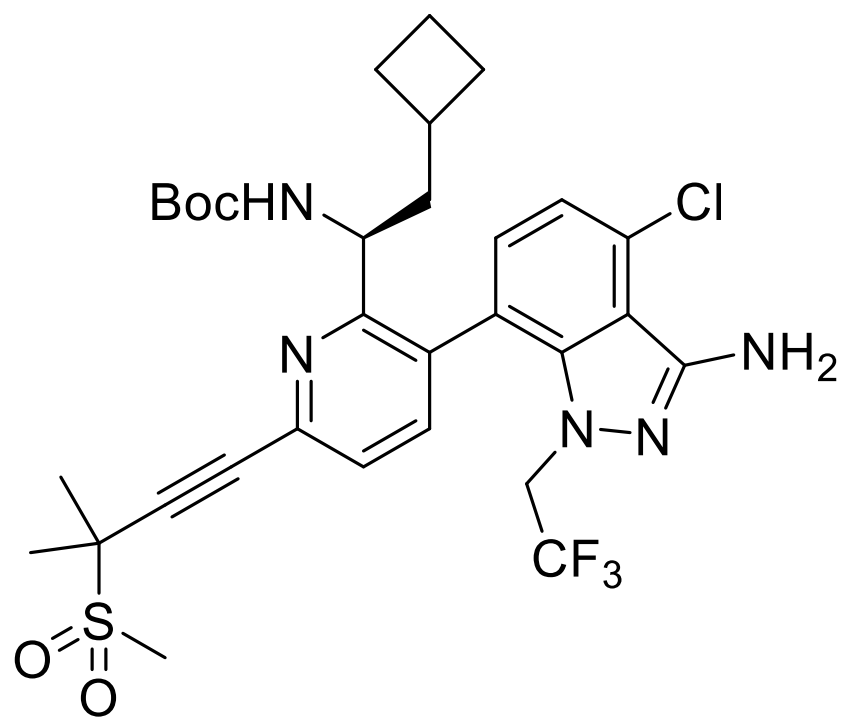


The title compound was prepared according to general procedure G using cyclobutyl subunit B **30bS** (111.8 mg, 0.22 mmol, 1.0 equiv.). Product was purified by CombiFlash on silica gel (0-40% Hexane/EtOAc) (59.8 mg, 41%, white solid). **$^{1}$H NMR** (400 MHz, $CDCl_3$) δ 7.62 – 7.52 (m, 1H), 7.44 (d, *J* = 7.9 Hz, 1H), 7.19 – 7.06 (m, 1H), 6.95 (d, *J* = 7.7 Hz, 1H), 5.43 (d, *J* = 9.5 Hz, 1H), 4.68 (s, 2H), 4.43 – 4.35 (m, 1H), 4.34 – 4.23 (m, 1H), 4.01 – 3.84 (m, 1H), 3.16 (s, 3H), 2.16 – 2.03 (m, 1H), 1.83 (s, 6H), 1.75 – 1.54 (m, 6H), 1.39 (s, 9H), 1.27 – 1.21 (m, 1H), 1.12 – 0.96 (m, 1H); **$^{13}$C NMR** (100 MHz, $CDCl_3$) δ 160.9, 155.1, 148.7, 142.1, 140.2, 138.8, 130.8, 130.4, 127.8, 125.9, 123.1 (q, *J* = 280.4 Hz), 119.8, 118.7, 114.0, 87.4, 85.5, 79.3, 57.9, 51.1, 50.2 (q, *J* = 33.1 Hz), 43.8, 35.3, 32.7, 28.3, 28.3 (3C), 27.9, 22.8, 22.7, 18.5; **$^{19}$F NMR** (376 MHz, $CDCl_3$) δ -70.26; **HRMS** ($ESI^+$) Calculated for $C_{31}H_{37}ClF_3N_5O_4S$ $[M+H]^+$ 668.2285; found 668.2285.

**Synthesis of tert-butyl (*R*)-(1-(3-(3-amino-4-chloro-1-(2,2,2-trifluoroethyl)-1H-indazol-7-yl)-6-(3-methyl-3-(methylsulfonyl)but-1-yn-1-yl)pyridin-2-yl)-2 cyclobutylethyl)carbamate (38bR)**

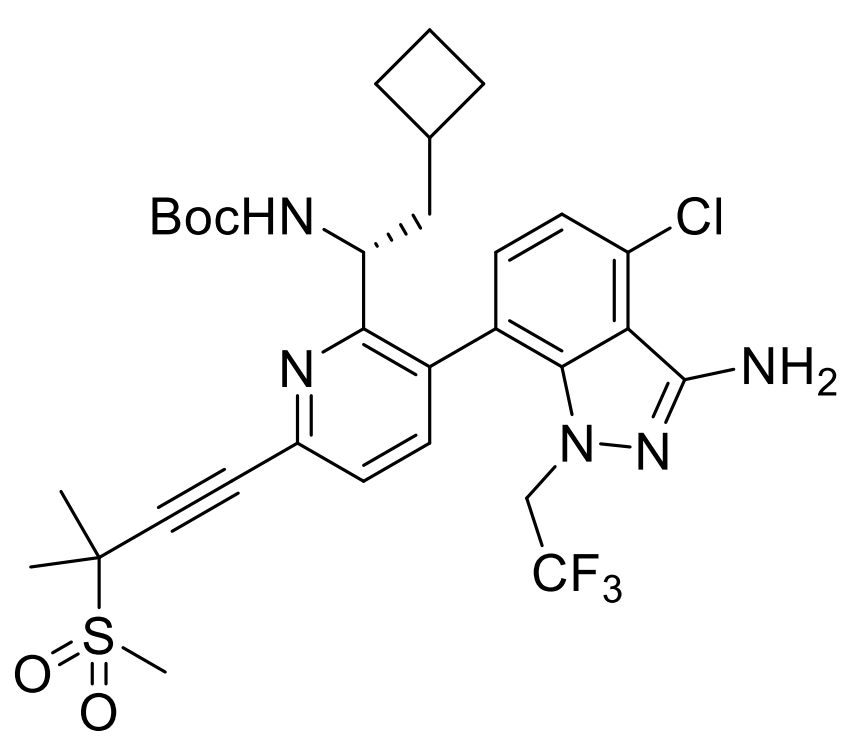


The title compound was prepared according to general procedure G using cyclobutyl subunit B **30bR** (334.9 mg, 0.67 mmol, 1.0 equiv.). Product was purified by CombiFlash on silica gel (0-40% Hexane/EtOAc) (259.0 mg, 58%, white solid). **$^{1}$H NMR** (400 MHz, $CDCl_3$) δ 7.61 – 7.52 (m, 1H), 7.44 (d, *J* = 7.9 Hz, 1H), 7.18 – 7.06 (m, 1H), 6.95 (d, *J* = 7.6 Hz, 1H), 5.42 (d, *J* = 8.9 Hz, 1H), 4.66 (s, 2H), 4.43 – 4.35 (m, 1H), 4.34 – 4.24 (m, 1H), 4.00 – 3.86 (m, 1H), 3.16 (s, 3H), 2.16 – 2.02 (m, 1H), 1.83 (s, 6H), 1.74 – 1.54 (m, 6H), 1.39 (s, 9H), 1.31 – 1.25 (m, 1H), 1.10 – 1.00 (m, 1H); **$^{13}$C NMR** (100 MHz, $CDCl_3$) δ 160.9, 155.0, 148.7, 142.1, 140.2, 138.8, 130.8, 130.4, 127.8, 125.9, 123.1 (q, *J* = 280.7 Hz), 119.8,

118.7, 114.0, 87.4, 85.5, 79.3, 57.9, 51.1, 50.2 (q, *J* = 33.9 Hz), 43.8, 35.3, 32.7, 28.4, 28.3 (3C), 27.9, 22.8, 22.7, 18.5; **$^{19}$F NMR** (376 MHz, $CDCl_3$) δ -70.26; **HRMS** ($ESI^+$) Calculated for $C_{31}H_{37}ClF_3N_5O_4S$ $[M+H]^+$ 668.2285; found 668.2283.

**Synthesis of tert-butyl (*S*)-(1-(3-(4-chloro-3-(*N*-(methylsulfonyl)methylsulfonamido)-1-(2,2,2-trifluoroethyl)-1H-indazol-7-yl)-6-(3-methyl-3-(methylsulfonyl)but-1-yn-1-yl)pyridin-2-yl)-2-cyclobutylethyl)carbamate (39bS)**

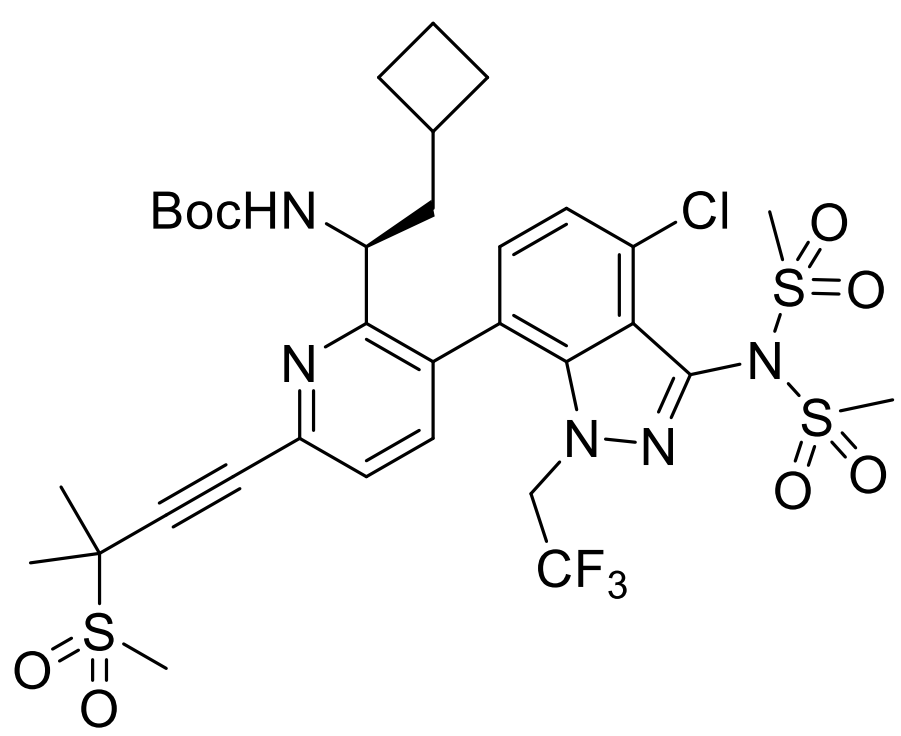


The title compound was prepared according to general procedure H using compound **38bS** (59.8 mg, 0.089 mmol, 1.0 equiv.). Product was purified by CombiFlash on silica gel (0-35% Hexane/EtOAc) (32.6 mg, 45%, white solid). **$^1$H NMR** (400 MHz, $CDCl_3$) δ 7.51 (d, *J* = 7.9 Hz, 1H), 7.40 (d, *J* = 7.9 Hz, 1H), 7.31 (d, *J* = 7.5 Hz, 1H), 7.05 (d, *J* = 7.5 Hz, 1H), 5.15 (d, *J* = 8.9 Hz, 1H), 4.57 (dq, *J* = 15.8, 7.6 Hz, 1H), 4.26 (dq, *J* = 15.6, 7.2 Hz, 1H), 4.20 – 4.11 (m, 1H), 3.56 (s, 3H), 3.51 (s, 3H), 3.09 (s, 3H), 2.08 – 1.94 (m, 1H), 1.76 (s, 6H), 1.68 – 1.42 (m, 6H), 1.30 (s, 9H), 1.26 – 1.17 (m, 1H), 0.86 – 0.77 (m, 1H); **$^{13}$C NMR** (100 MHz, $CDCl_3$) δ 161.2, 155.2, 142.6, 140.5, 138.8, 135.5, 131.3, 129.5, 126.7, 125.9, 123.7, 122.6 (q, *J* = 280.2 Hz), 120.1, 120.1, 87.8, 85.4, 79.5, 58.0, 51.6 (q, *J* = 33.6 Hz), 51.4, 42.8, 42.4 (2C), 35.3, 32.7, 28.3 (4C), 27.8, 22.7 (2C), 18.5; **$^{19}$F NMR** (376 MHz, $CDCl_3$) δ -70.18; **HRMS** ($ESI^+$) Calculated for $C_{33}H_{41}ClF_3N_5O_8S_3$ $[M+H]^+$ 824.1836; found 824.1835.

**Synthesis of tert-butyl (*R*)-(1-(3-(4-chloro-3-(*N*-(methylsulfonyl)methylsulfonamido)-1-(2,2,2-trifluoroethyl)-1H-indazol-7-yl)-6-(3-methyl-3-(methylsulfonyl)but-1-yn-1-yl)pyridin-2-yl)-2-cyclobutylethyl)carbamate (39bR)**

The title compound was prepared according to general procedure H using compound **38bR** (259.0 mg, 0.388 mmol, 1.0 equiv.). Product was purified by CombiFlash on silica gel (0-35%

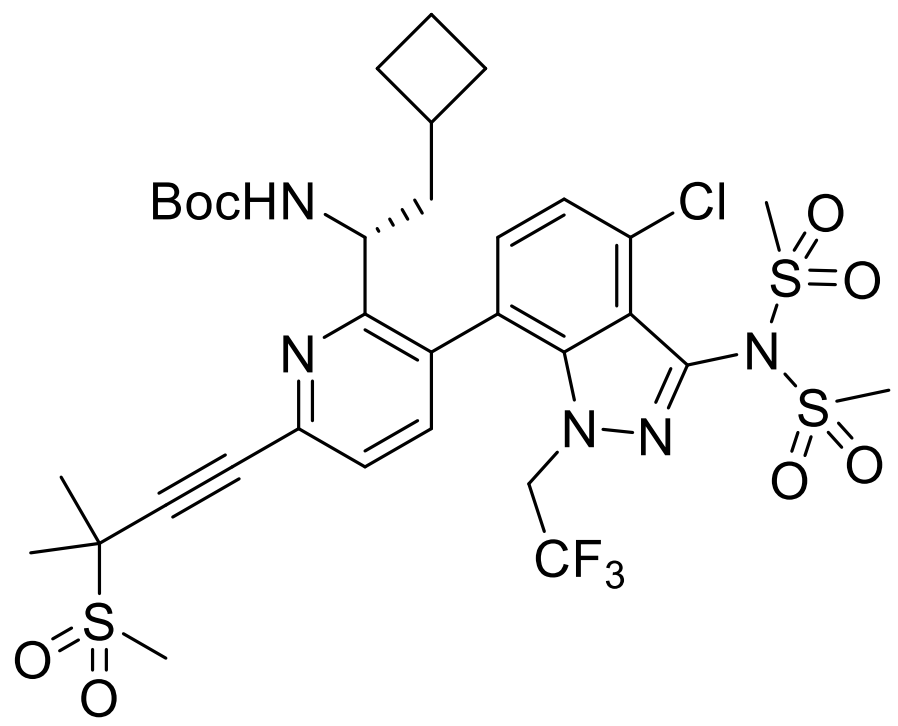


Hexane/EtOAc) (242.0 mg, 76%, white solid). **$^1$H NMR** (400 MHz, $CDCl_3$) δ 7.51 (d, *J* = 6.9 Hz, 1H), 7.40 (d, *J* = 7.9 Hz, 1H), 7.31 (d, *J* = 7.6 Hz, 1H), 7.05 (d, *J* = 8.1 Hz, 1H), 5.14 (d, *J* = 8.7 Hz, 1H), 4.57 (dq, *J* = 16.3, 8.0 Hz, 1H), 4.25 (dq, *J* = 16.4, 8.0 Hz, 1H), 4.20 – 4.12 (m, 1H), 3.56 (s, 3H), 3.51 (s, 3H), 3.09 (s, 3H), 2.06 – 1.97 (m, 1H), 1.76 (s, 6H), 1.68 – 1.43 (m, 6H), 1.30 (s, 9H), 1.26 – 1.20 (m, 1H), 0.84 – 0.75 (m, 1H); **$^{13}$C NMR** (100 MHz, $CDCl_3$) δ 161.2, 155.2, 142.6, 140.5, 138.8, 135.5, 131.3, 129.4, 126.7, 125.8, 123.7, 122.6 (q, *J* = 281.0 Hz), 120.2, 120.0, 87.7, 85.4, 79.4, 58.0, 51.6 (q, *J* = 33.1 Hz), 51.4, 42.8, 42.4 (2C), 35.3, 32.7, 28.3 (4C), 27.8, 22.7 (2C), 18.5; **$^{19}$F NMR** (376 MHz, $CDCl_3$) δ -70.18; **HRMS** ($ESI^+$) Calculated for $C_{33}H_{41}ClF_3N_5O_8S_3$ $[M+H]^+$ 824.1836; found 824.1830.

**Synthesis of *N*-((*S*)-1-(3-(4-chloro-3-(methylsulfonamido)-1-(2,2,2-trifluoroethyl)-1H-indazol-7-yl)-6-(3-methyl-3-(methylsulfonyl)but-1-yn-1-yl)pyridin-2-yl)-2-cyclobutylethyl)-2-((3b*R*,4aS)-5,5-difluoro-3-(trifluoromethyl)-3b,4,4a,5-tetrahydro-1H-cyclopropa[3,4]cyclopenta[1,2-c]pyrazol-1-yl)acetamide (2S)**

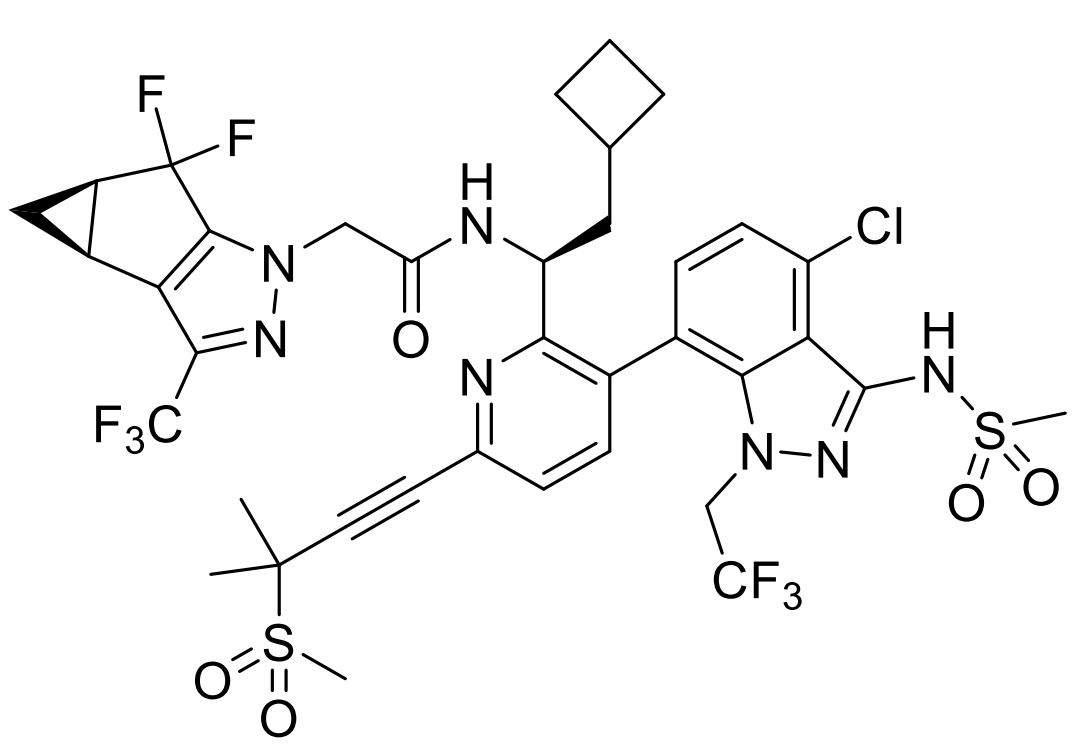


The title compound was prepared according to general procedure I using compound **39bS** (32.6 mg, 0.039 mmol, 1.0 equiv.). Product was purified by CombiFlash on silica gel (0-45% Hexane/EtOAc) (13.4 mg, 37% over three steps, white solid, 98% pure, evaluated on HPLC-UV sig=250 nm). **$^1$H NMR** (400 MHz, $CDCl_3$) δ 7.58 (d, *J* = 8.0 Hz, 1H), 7.48 (d, *J* = 7.9 Hz, 1H), 7.44 (s, 1H), 7.24 (d, *J* = 7.6 Hz, 1H), 7.05 (d, *J* = 7.7 Hz, 1H), 6.65 (d, *J* = 8.7 Hz, 1H), 4.74 (s, 2H), 4.58 – 4.52 (m, 1H), 4.48 (dq, *J* = 16.4, 8.0 Hz, 1H), 3.95 (dq, *J* = 16.0, 8.1 Hz, 1H), 3.40 (s, 3H), 3.15 (s, 3H), 2.56 – 2.43 (m, 2H), 2.03 – 1.93 (m, 1H), 1.83 (s, 6H), 1.79 – 1.60 (m, 5H), 1.58 – 1.50 (m, 1H), 1.47 – 1.39 (m, 1H), 1.37 – 1.29 (m, 1H), 1.17 – 1.08 (m, 1H), 1.06 – 0.94 (m, 1H); **$^{13}$C NMR** (100 MHz, $CDCl_3$) δ 163.9, 159.5, 143.5 (t, *J* = 29.0 Hz), 142.6, 139.9, 139.6, 139.0, 136.8 (q, *J* = 39.8 Hz), 132.9 – 132.6 (m), 131.3, 129.6, 127.0, 126.4, 122.7 (q, *J* =

279.8 Hz), 121.7, 120.5 (q, $J$ = 267.5 Hz), 119.9 (t, $J$ = 242.5 Hz), 118.9, 115.0, 88.2, 85.0, 58.0, 53.7, 50.8 (q, $J$ = 34.2 Hz), 50.2, 43.0, 41.6, 35.2, 32.5, 28.3, 28.0 (dd, $J$ = 34.3, 29.0 Hz), 27.8, 23.4, 22.7, 22.7, 18.4, 12.3; **$^{19}$F NMR** (376 MHz, $CDCl_3$) δ -61.60, -70.34, -81.03 (d, $J$ = 255.9 Hz), -103.50 (d, $J$ = 256.0 Hz); (minor atropisomer not included); **HRMS** (ESI$^+$) Calculated for $C_{37}H_{37}ClF_8N_7O_5S_2$ $[M+H]^+$ 910.1858; found 910.1846.

**Synthesis of *N*-((R)-1-(3-(4-chloro-3-(methylsulfonamido)-1-(2,2,2-trifluoroethyl)-1H-indazol-7-yl)-6-(3-methyl-3-(methylsulfonyl)but-1-yn-1-yl)pyridin-2-yl)-2-cyclobutylethyl)-2-((3b*R*,4aS)-5,5-difluoro-3-(trifluoromethyl)-3b,4,4a,5-tetrahydro-1H-cyclopropa[3,4]cyclopenta[1,2-c]pyrazol-1-yl)acetamide (2R)**

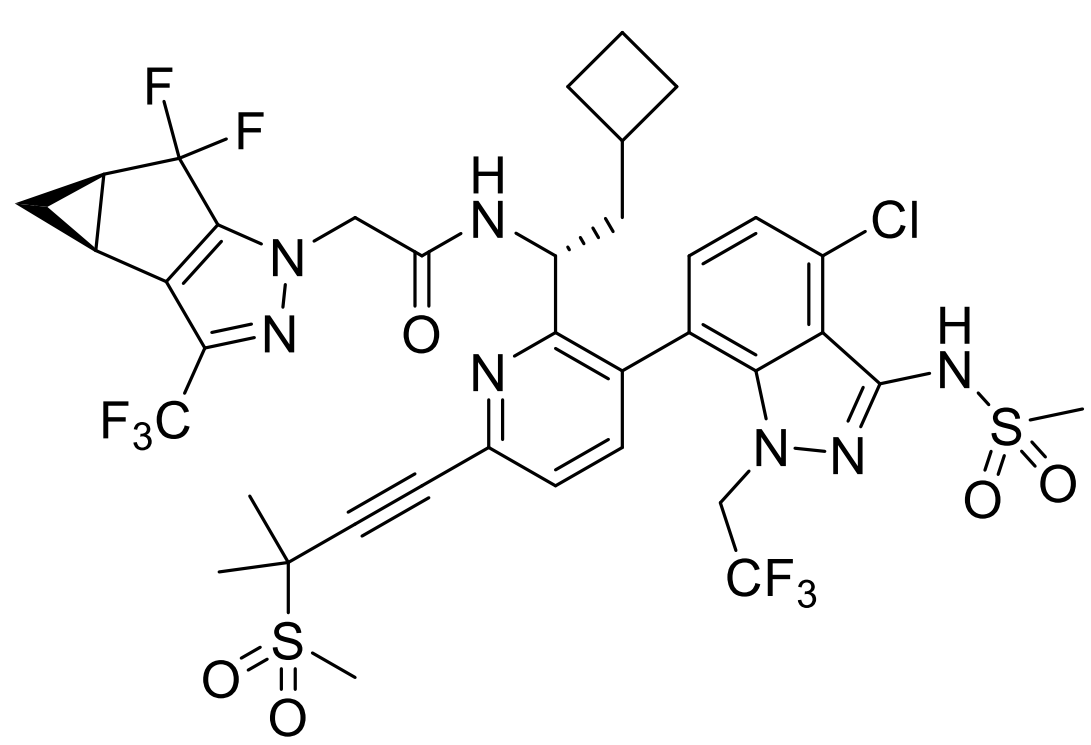


The title compound was prepared according to general procedure I using compound **39bR** (242.0 mg, 0.29 mmol, 1.0 equiv.). Product was purified by CombiFlash on silica gel (0-45% Hexane/EtOAc) (139.8 mg, 53% over three steps, white solid, 97% pure, evaluated on HPLC-UV sig=250 nm). **$^1$H NMR** (400 MHz, $CDCl_3$) δ 7.57 (d, $J$ = 7.9 Hz, 1H), 7.50 (d, $J$ = 5.5 Hz, 1H), 7.48 (s, 1H), 7.23 (d, $J$ = 7.6 Hz, 1H), 7.05 (d, $J$ = 7.7 Hz, 1H), 6.65 (d, $J$ = 8.8 Hz, 1H), 4.74 (s, 2H), 4.62 – 4.55 (m, 1H), 4.49 (dq, $J$ = 16.2, 8.1 Hz, 1H), 3.92 (dq, $J$ = 16.0, 8.0 Hz, 1H), 3.39 (s, 3H), 3.14 (s, 3H), 2.52 – 2.41 (m, 2H), 2.06 – 1.94 (m, 1H), 1.81 (s, 6H), 1.75 – 1.58 (m, 5H), 1.57 – 1.48 (m, 1H), 1.45 – 1.38 (m, 1H), 1.36 – 1.29 (m, 1H), 1.17 – 1.06 (m, 1H), 1.04 – 0.88 (m, 1H); **$^{13}$C NMR** (100 MHz, $CDCl_3$) δ 164.01, 159.5, 143.5 (t, $J$ = 29.3 Hz), 142.6, 139.9, 139.6, 139.0, 136.7 (q, $J$ = 39.8 Hz), 132.9 – 132.6 (m), 131.3, 129.7, 127.0, 126.4, 122.7 (q, $J$ = 279.9 Hz), 121.7, 120.5 (q, $J$ = 267.3 Hz), 119.9 (t, $J$ = 243.0 Hz), 119.0, 115.2, 88.2, 85.1, 58.0, 53.7, 50.8 (q, $J$ = 34.9 Hz), 49.9, 43.1, 41.5, 35.2, 32.4, 28.3, 28.0 (dd, $J$ = 34.3, 29.1 Hz), 27.8, 23.4, 22.7, 22.6, 18.4, 12.3; **$^{19}$F NMR** (376 MHz, $CDCl_3$) δ -61.55, -70.33, -81.25 (d, $J$ = 256.2 Hz), -103.52 (d, $J$ = 255.7 Hz) (minor atropisomer not included); **HRMS** (ESI$^+$) Calculated for $C_{37}H_{37}ClF_8N_7O_5S_2$ $[M+H]^+$ 910.1858; found 910.1858.

**tert-butyl (S)-(1-(3-(3-amino-4-chloro-1-(2,2,2-trifluoroethyl)-1H-indazol-7-yl)-6-(3-methyl-3-(methylsulfonyl)but-1-yn-1-yl)pyridin-2-yl)-2-cyclopentylethyl)carbamate (38cS)**

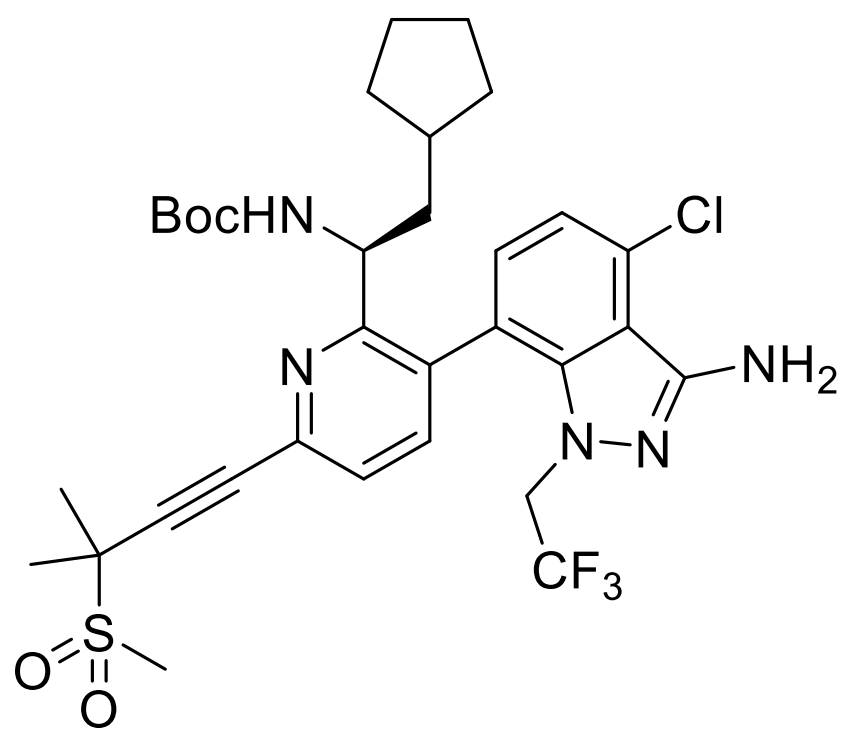


The title compound was prepared according to general procedure G using cyclopropyl subunit B **30cS** (689 mg, 1.34 mmol, 1.0 equiv.). Product was purified by CombiFlash on silica gel (0-40% Hexane/EtOAc) (406 mg, 44%, white solid). Observation of 2 atropoisomers on NMR in a 0.4:0.6 ratio. **$^{1}$H-NMR** (400 MHz, $CDCl_3$) δ ppm: 7.59 (d, *J* = 8.2 Hz, 0.4H, atropoisomer), 7.56 (d, *J* = 8.2 Hz, 0.6H atropoisomer), 7.44 (d, *J* = 7.9 Hz, 1H), 7.18 – 7.09 (m, 0.4H, atropoisomer), 7.06 (d, *J* = 7.6 Hz, 0.6H, atropoisomer), 7.01 – 6.92 (m, 0.4H, atropoisomer), 6.91 (d, *J* = 7.7 Hz, 0.6H, atropoisomer), 5.53 – 5.39 (m, 0.6H, atropoisomer), 4.69 (br s, 1.6H, atropoisomer), 4.49 – 4.42 (m, 0.4H, atropoisomer), 4.34 (dq, *J* = 16.1, 8.0 Hz, 1H), 3.98 (dq, *J* = 16.6, 8.2 Hz, 0.6H, atropoisomer), 3.93 – 3.80 (m, 0.4H, atropoisomer), 3.16 (s, 1.8H, atropoisomer), 3.15 (s, 1.2H, atropoisomer), 1.82 (s, 6H), 1.79 (d, *J* = 5.8 Hz, 1H), 1.70 – 1.52 (m, 4H), 1.51 – 1.22 (m, 15H), 0.96 – 0.76 (m, 1H), 0.42 (s, 1H); **$^{13}$C-NMR** (100 MHz, $CDCl_3$) δ ppm: 161.5, 155.4, 148.8, 142.2, 140.3, 138.9, 131.0, 130.5, 127.9, 127.7, 126.0, 119.8, 118.9, 114.1, 87.5, 85.7, 79.4, 58.1, 52.1, 43.3, 36.6, 35.4, 33.0, 31.9, 31.7, 28.4, 25.0, 24.8, 24.8, 22.9, 22.8, 22.8, 22.8, 14.3, 14.2; **HRMS** ($ESI^+$) (m/z): $C_{32}H_{39}ClF_3N_5O_4S$ $[M+H]^+$ calculated 682.2437, found 682.2414.

**Synthesis of tert-butyl (*R*)-(1-(3-(3-amino-4-chloro-1-(2,2,2-trifluoroethyl)-1H-indazol-7-yl)-6-(3-methyl-3-(methylsulfonyl)but-1-yn-1-yl)pyridin-2-yl)-2-cyclopentylethyl)carbamate (38cR)**

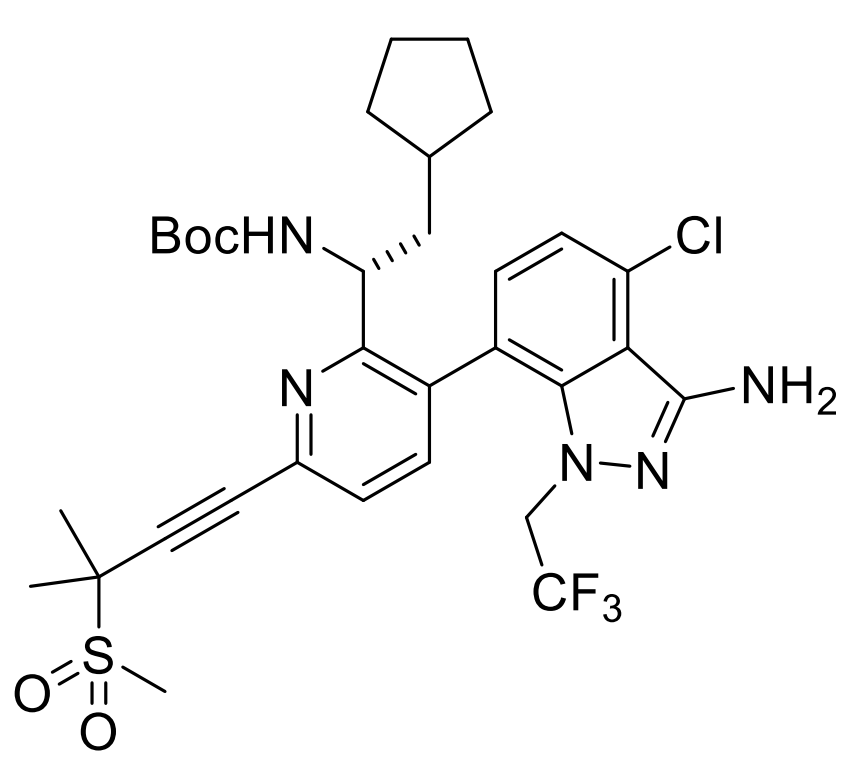


The title compound was prepared according to general procedure G using cyclopropyl subunit B **30cR** (402.2 mg, 0.78 mmol, 1.0 equiv.). Product was purified by CombiFlash on silica gel (0-40% Hexane/EtOAc) (256.1 mg, 48%, white solid). **$^{1}$H NMR** (400 MHz, $CDCl_3$) δ 7.50 (d, *J* = 8.5 Hz, 1H), 7.40 (d, *J* = 7.8 Hz, 1H), 7.00 (d, *J* = 7.7 Hz, 1H), 6.86 (d, *J* = 7.7 Hz, 1H), 5.48 (d, *J* = 9.5 Hz, 1H), 4.77 (s, 2H), 4.45 – 4.36 (m,

1H), 4.35 – 4.24 (m, 1H), 4.08 – 3.88 (m, 1H), 3.10 (s, 3H), 1.76 (s, 6H), 1.63 – 1.49 (m, 3H), 1.45 – 1.20 (m, 6H), 1.32 (s, 9H), 0.90 – 0.74 (m, 1H), 0.41 – 0.30 (m, 1H); **$^{13}$C NMR** (100 MHz, $CDCl_3$) δ 161.4, 155.3, 148.8, 142.1, 140.2, 138.7, 130.9, 130.3, 127.7, 125.9, 123.2 (q, *J* = 280.0 Hz), 119.7, 118.8, 113.9, 87.4, 85.5, 79.2, 57.9, 51.9, 50.3 (q, *J* = 34.3 Hz), 43.1, 36.5, 35.2, 32.9, 31.6, 28.3, 24.9, 24.7, 22.7; **$^{19}$F NMR** (376 MHz, $CDCl_3$) δ -70.22; **HRMS** ($ESI^+$) Calculated for $C_{32}H_{39}ClF_3N_5O_4S$ $[M+H]^+$ 682.2437; found 682.2442.

**tert-butyl (S)-(1-(3-(4-chloro-3-(N-(methylsulfonyl)methylsulfonamido)-1-(2,2,2-trifluoroethyl)-1H-indazol-7-yl)-6-(3-methyl-3-(methylsulfonyl)but-1-yn-1-yl)pyridin-2-yl)-2-cyclopentylethyl)carbamate (39cS)**

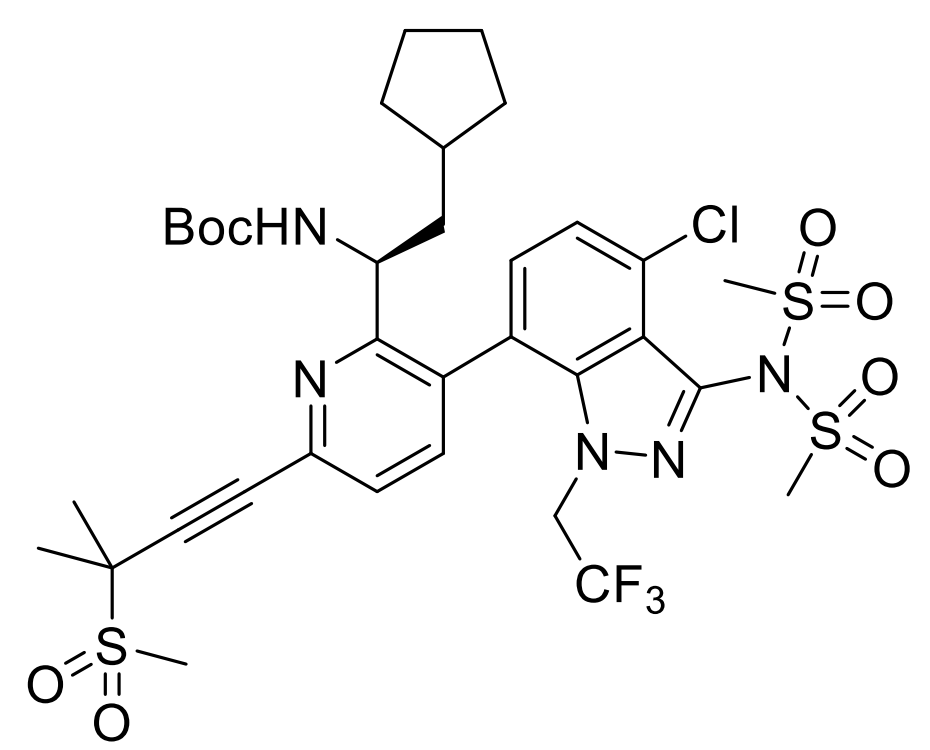


The title compound was prepared according to general procedure H using compound **38cS** (406 mg, 0.595 mmol, 1.0 equiv.). Product was purified by CombiFlash on silica gel (0-30% Hexane/EtOAc) (392 mg, 79%, white solid). **$^1$H-NMR** (400 MHz, $CDCl_3$) δ ppm: 7.58 (d, *J* = 7.9 Hz, 1H), 7.47 (d, *J* = 8.1 Hz, 1H), 7.36 (d, *J* = 7.5 Hz, 1H), 7.08 (d, *J* = 7.8 Hz, 1H), 5.22 (d, *J* = 8.7 Hz, 1H), 4.76 – 4.59 (m, 1H), 4.47 – 4.25 (m, 1H), 3.61 (d, *J* = 19.4 Hz, 6H), 3.16 (s, 3H), 1.82 (s, 6H), 1.69 – 1.22 (m, 18H), 0.94 – 0.78 (m, 1H), 0.30 – 0.12 (m, 2H); **$^{13}$C-NMR** (100 MHz, $CDCl_3$) δ ppm: 161.7, 155.6, 142.6, 141.5, 140.5, 138.8, 135.6, 131.6, 129.5, 126.7, 125.9, 123.8, 120.3, 120.2, 87.7, 85.6, 79.5, 60.4, 58.1, 52.6, 42.9, 42.5, 36.5, 35.4, 31.6, 28.4, 25.0, 24.8, 24.8, 22.8; **HRMS** ($ESI^+$) (m/z): Calculated for $C_{34}H_{43}ClF_3N_5O_8S_3$ $[M+H]^+$ 838.1988, found 838.1983. And $[M+Na]^+$ calculated 860.1807, found 860.1802.

**Synthesis of tert-butyl (*R*)-(1-(3-(4-chloro-3-(*N*-(methylsulfonyl)methylsulfonamido)-1-(2,2,2-trifluoroethyl)-1H-indazol-7-yl)-6-(3-methyl-3-(methylsulfonyl)but-1-yn-1-yl)pyridin-2-yl)-2-cyclopentylethyl)carbamate (39cR)**

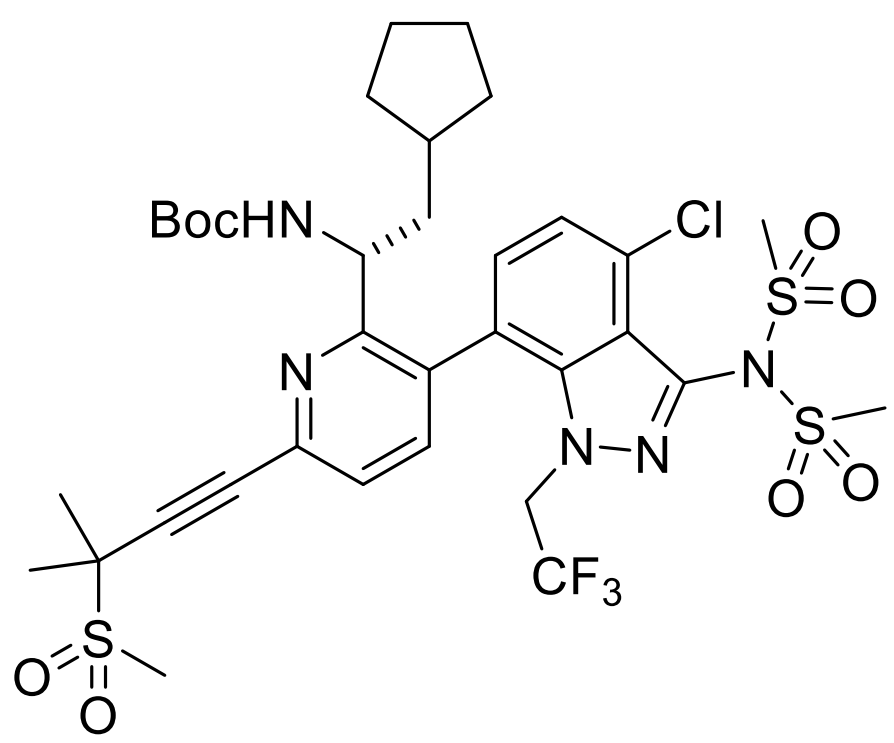


The title compound was prepared according to general procedure H using compound **38cR** (256.1 mg, 0.37 mmol, 1.0 equiv.). Product was purified by CombiFlash on silica gel (0-30% Hexane/EtOAc) (225.3 mg, 72%, white solid). **$^{1}$H NMR** (400 MHz, $CDCl_3$) δ 7.58 (d, *J* = 8.0 Hz, 1H), 7.47 (d, *J* = 8.0 Hz, 1H), 7.36 (d, *J* = 7.6 Hz, 1H), 7.08 (d, *J* = 7.7 Hz, 1H), 5.22 (d, *J* = 8.8 Hz, 1H), 4.78 – 4.58 (m, 1H), 4.48 – 4.35 (m, 1H), 4.34 – 4.23 (m, 1H), 3.64 (s, 3H), 3.59 (s, 3H), 3.16 (s, 3H), 1.83 (s, 6H), 1.62 – 1.50 (m, 3H), 1.38 (s, 9H), 1.46 – 1.22 (m, 6H), 0.92 – 0.80 (m, 1H), 0.25 – 0.10 (m, 1H); **$^{13}$C NMR** (100 MHz, $CDCl_3$) δ 161.6, 155.5, 142.5, 140.4, 138.7, 135.5, 131.5, 129.4, 126.6, 125.8, 123.7, 122.2 (q, *J* = 280.0 Hz), 120.2, 120.1, 87.6, 85.5, 79.4, 60.3, 57.9, 52.5, 50.33 (q, *J* = 35.1 Hz), 42.8, 42.4, 36.4, 35.2, 32.9, 31.4, 28.3, 24.9, 24.7, 22.7; **$^{19}$F NMR** (376 MHz, $CDCl_3$) δ -70.23; **HRMS** ($APCI^+$) Calculated for $C_{34}H_{43}ClF_3N_5O_8S_3$ $[M+H]^+$ 838.1988; found 838.1985.

**N-((S)-1-(3-(4-chloro-3-(methylsulfonamido)-1-(2,2,2-trifluoroethyl)-1H-indazol-7-yl)-6-(3-methyl-3-(methylsulfonyl)but-1-yn-1-yl)pyridin-2-yl)-2-cyclopentylethyl)-2-((3bS,4aR)-5,5-difluoro-3-(trifluoromethyl)-3b,4,4a,5-tetrahydro-1H-cyclopropa[3,4]cyclopenta[1,2-c]pyrazol-1-yl)acetamide (3S)**

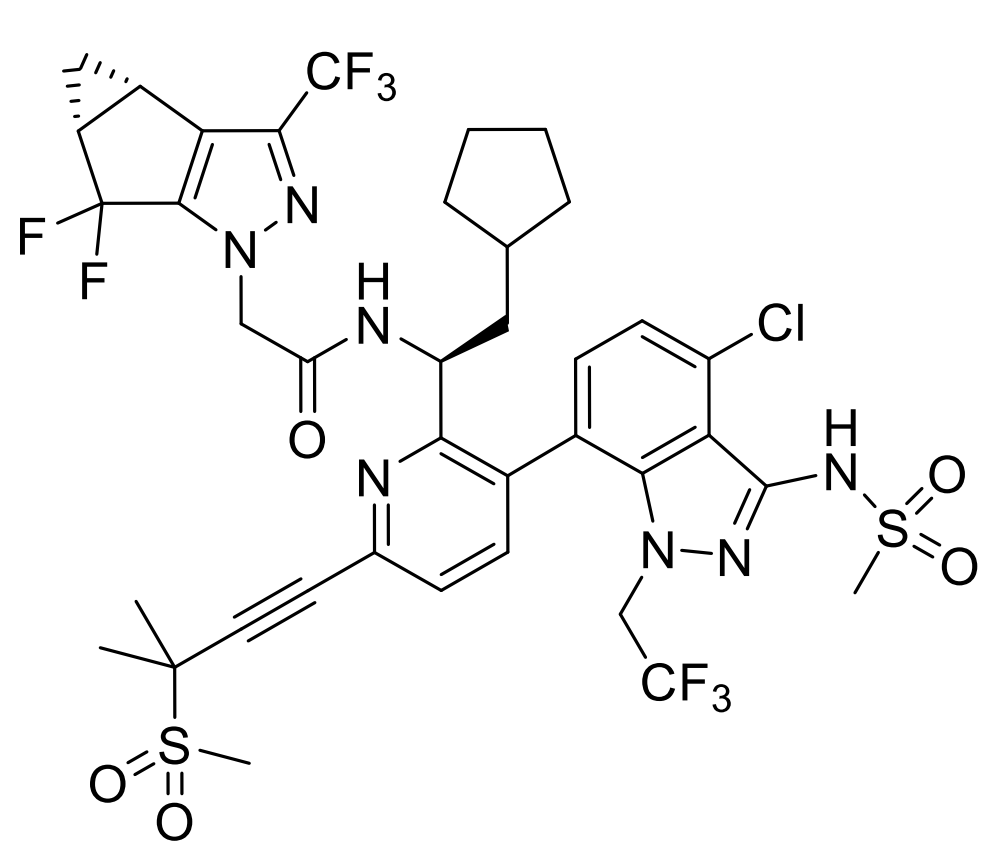


The title compound was prepared according to general procedure I, using compound **39cS** (390 mg, 0.466 mmol, 1.0 equiv.). Product was purified by CombiFlash on silica gel (0-50% Hexane/EtOAc) (163 mg, 37% over two steps, white solid, 97% pure, evaluated on HPLC-UV sig=250 nm). The two atropoisomers of **3S** were separated. The NMR of the isolated major atropoisomer is described below, showing a 10% conversion into the minor atropoisomer within hours. **$^{1}$H NMR** (400 MHz, CDCl3) δ ppm: 7.57 (d, J = 7.9 Hz, 1H), 7.48 (d,

J = 7.9 Hz, 1H), 7.43 (s, 1H), 7.21 (d, J = 7.7 Hz, 1H), 7.01 (d, J = 7.7 Hz, 1H), 6.62 (d, J = 8.6 Hz, 0.9H, major atropoisomer), 6.47 (d, J = 8.9 Hz, 0.1H, minor atropoisomer), 4.75 (d, J = 1.6 Hz, 1.8H, major atropoisomer), 4.72 (d, J = 4.5 Hz, 0.2H, minor atropoisomer), 4.62 (ddd, J = 9.9, 8.5, 3.7 Hz, 1H), 4.53 (dq, J = 16.3, 8.1 Hz, 0.9H, major atropoisomer), 4.41 (dq, J = 15.5, 7.6 Hz, 0.1H, minor atropoisomer), 3.99 (dq, J = 16.1, 8.2 Hz, 1H), 3.41 (s, 3H), 3.15 (s, 3H), 2.54 – 2.43 (m, 2H), 1.82 (s, 6H), 1.63 (ddd, J = 14.1, 9.7, 3.9 Hz, 1H), 1.53 – 1.23 (m, 10H), 1.15 – 1.08 (m, 1H), 0.92 – 0.78 (m, 1H), 0.39 – 0.24 (m, 1H). **$^{13}$C NMR** (100 MHz, CDCl3) δ 164.2, 160.2, 143.7, 142.8, 140.0, 139.7, 139.0, 131.5, 129.7, 127.1, 126.4, 121.7, 119.2, 115.2, 88.1, 85.3, 58.1, 53.9, 51.1, 42.7, 41.7, 36.6, 35.4, 33.0, 31.8, 28.2 (dd, J = 34.4, 29.6 Hz), 25.0, 24.8, 23.5, 22.8, 12.4; **$^{19}$F NMR** (376 MHz, $CDCl_3$) δ -61.59, -70.30, -81.03 (d, *J* = 256.6 Hz), -103.50 (d, *J* = 256.0 Hz). **HRMS** (ESI$^+$) Calculated for $C_{38}H_{39}ClF_8N_7O_5S_2$ $[M+H]^+$ 924.2009; found 924.2024.

**Synthesis of *N*-((*R*)-1-(3-(4-chloro-3-(methylsulfonamido)-1-(2,2,2-trifluoroethyl)-1H-indazol-7-yl)-6-(3-methyl-3-(methylsulfonyl)but-1-yn-1-yl)pyridin-2-yl)-2-cyclopentylethyl)-2-((3bR,4aS)-5,5-difluoro-3-(trifluoromethyl)-3b,4,4a,5-tetrahydro-1H-cyclopropa[3,4]cyclopenta[1,2-c]pyrazol-1-yl)acetamide (3R)**

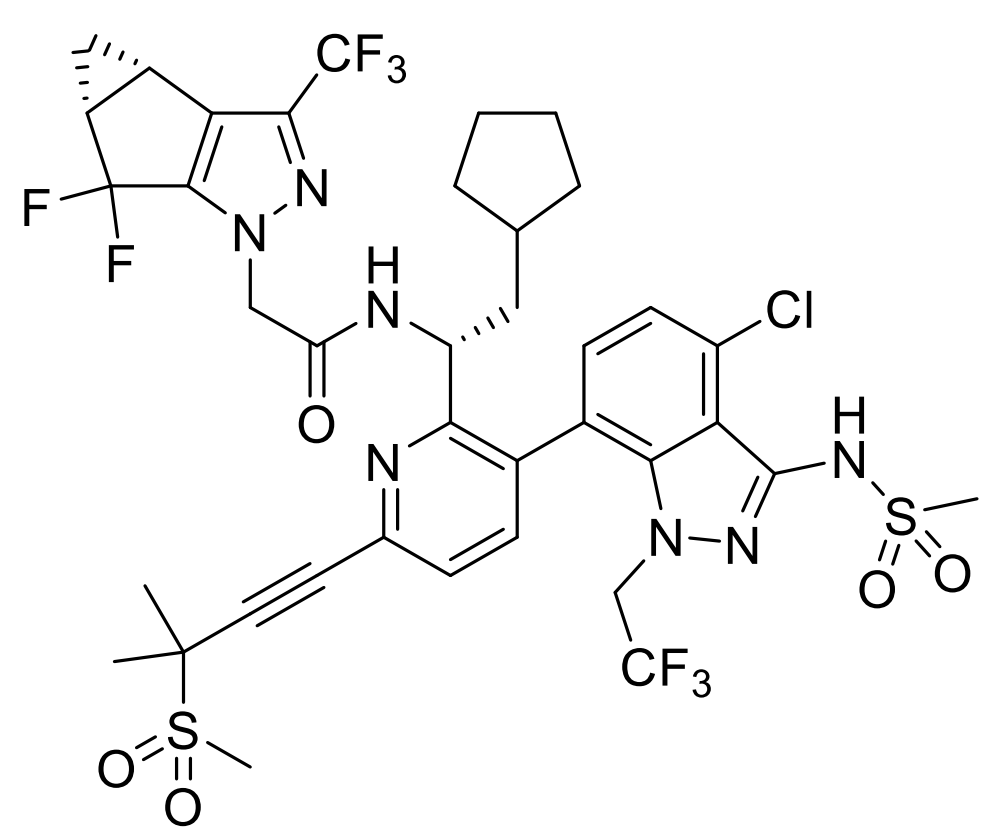

The title compound was prepared according to general procedure I, using compound **39cR** (225.3 mg, 0.27 mmol, 1.0 equiv.). Product was purified by CombiFlash on silica gel (0-50% Hexane/EtOAc) (113.6 mg, 47% over three steps, white solid, 98% pure, evaluated on HPLC-UV sig=250 nm). **$^1$H NMR** (400 MHz, $CDCl_3$) δ 7.60 (d, *J* = 6.9 Hz, 1H), 7.51 (d, *J* = 2.0 Hz, 1H), 7.29 (d, *J* = 1.0 Hz, 1H), 7.22 (d, *J* = 7.6 Hz, 1H), 7.02 (d, *J* = 7.6 Hz, 1H), 6.71 (d, *J* = 8.7 Hz, 1H), 4.77 (d, *J* = 3.3 Hz, 2H), 4.71 – 4.63 (m, 1H), 4.55 (dq, *J* = 16.2, 8.2 Hz, 1H), 3.98 (dq, *J* = 16.0, 8.1 Hz, 1H), 3.41 (s, 3H), 3.16 (s, 3H), 2.53 – 2.41 (m, 2H), 1.82 (s, 6H), 1.69 – 1.58 (m, 1H), 1.55 – 1.47 (m, 2H), 1.46 – 1.39 (m, 3H), 1.37 – 1.29 (m, 4H), 1.17 – 1.09 (m, 1H), 0.92 – 0.78 (m, 1H), 0.36 – 0.24 (m, 1H); **$^{13}$C NMR** (100 MHz, $CDCl_3$) δ 164.2, 160.1, 143.5 (t, *J* = 29.4 Hz), 142.6, 139.8, 139.6, 138.9, 136.7 (q, *J* = 39.6 Hz), 132.9 – 132.7 (m), 131.4, 129.7, 127.0, 126.4, 122.8 (q, *J* = 279.6 Hz), 121.6, 120.5 (q, *J* = 267.3 Hz), 119.9 (t, *J* =

243.4 Hz), 119.1, 115.2, 88.1, 85.1, 58.0, 53.7, 50.9 (q, *J* = 34.9 Hz), 50.9, 42.6, 41.6, 36.4, 35.2, 32.9, 31.7, 28.0 (dd, *J* = 34.7, 29.4 Hz), 24.8, 24.7, 23.3, 22.7, 22.6, 12.3; **$^{19}$F NMR** (376 MHz, $CDCl_3$) δ -61.576, -70.30, -81.27 (d, *J* = 256.3 Hz), -103.56 (d, *J* = 256.1 Hz) (minor atropisomer not included); **HRMS** (ESI$^+$) Calculated for $C_{38}H_{39}ClF_8N_7O_5S_2$ $[M+H]^+$ 924.2009; found 924.2011.

**tert-butyl (S)-(1-(3-(3-amino-4-chloro-1-(2,2,2-trifluoroethyl)-1H-indazol-7-yl)-6-(3-methyl-3-(methylsulfonyl)but-1-yn-1-yl)pyridin-2-yl)-2-cyclohexylethyl)carbamate (38dS)**

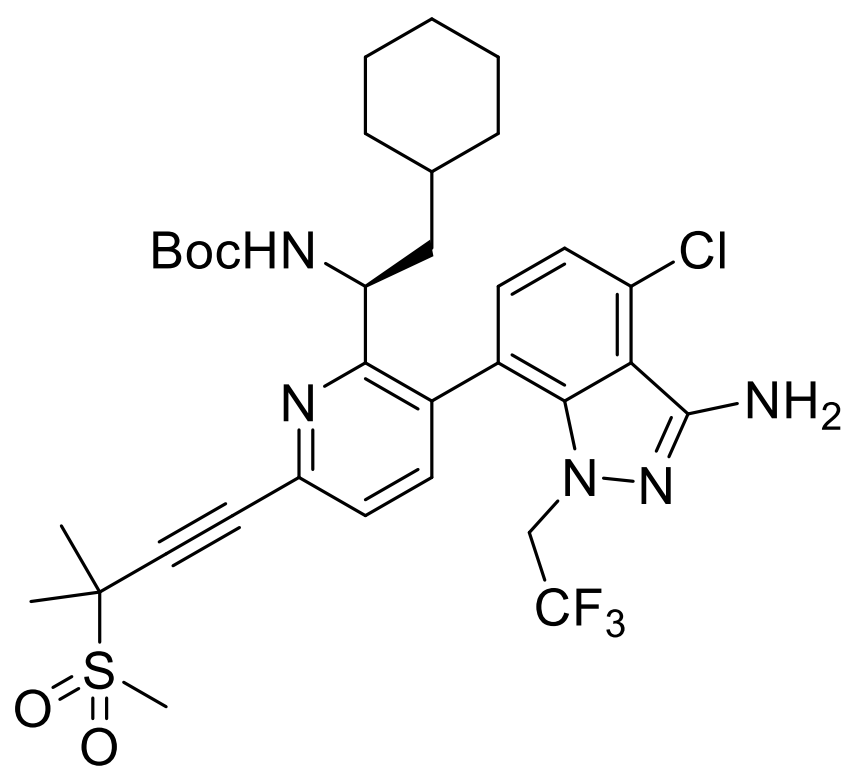


The title compound was prepared according to general procedure G using cyclopropyl subunit B **30dS** (229 mg, 0.435 mmol, 1.0 equiv.). Product was purified by CombiFlash on silica gel (0-40% Hexane/EtOAc) (112 mg, 37%, white solid). Observation of 2 atropoisomers on NMR. **$^1$H NMR** (400 MHz, $CDCl_3$) δ 7.63 – 7.51 (m, 1H), 7.43 (d, *J* = 7.9 Hz, 1H), 7.21 – 7.09 (m, ~0.4H), 7.06 (d, *J* = 7.6 Hz, ~0.6H), 6.97 (d, *J* = 7.4 Hz, ~0.4H), 6.90 (d, *J* = 7.6 Hz, ~0.6H), 5.50 (d, *J* = 9.8 Hz, ~0.3H), 5.38 (d, *J* = 9.5 Hz, ~0.7H), 4.82 – 4.61 (m, 2H), 4.57 – 4.44 (m, 1H), 4.39 – 4.18 (m, 1H), 4.06 – 3.79 (m, 1H), 3.15 (s, 3H), 1.82 (s, 6H), 1.60 – 0.94 (m, 20H), 0.81 – 0.64 (m, 1H), 0.39 – 0.14 (m, 1H); **$^{13}$C NMR** (100 MHz, $CDCl_3$) δ 161.7, 155.4, 149.3, 148.8, 142.3, 141.6, 140.3, 138.8, 138.6, 131.0, 130.4, 127.9, 126.0, 119.9, 118.9, 114.1, 87.5, 85.7, 58.1, 50.2, 44.9, 35.4, 34.1, 34.0, 33.8, 32.1, 28.5, 28.1, 26.5, 26.4, 26.3, 26.1, 25.0, 22.9, 14.3.

**tert-butyl (R)-(1-(3-(3-amino-4-chloro-1-(2,2,2-trifluoroethyl)-1H-indazol-7-yl)-6-(3-methyl-3-(methylsulfonyl)but-1-yn-1-yl)pyridin-2-yl)-2-cyclohexylethyl)carbamate (38dR)**

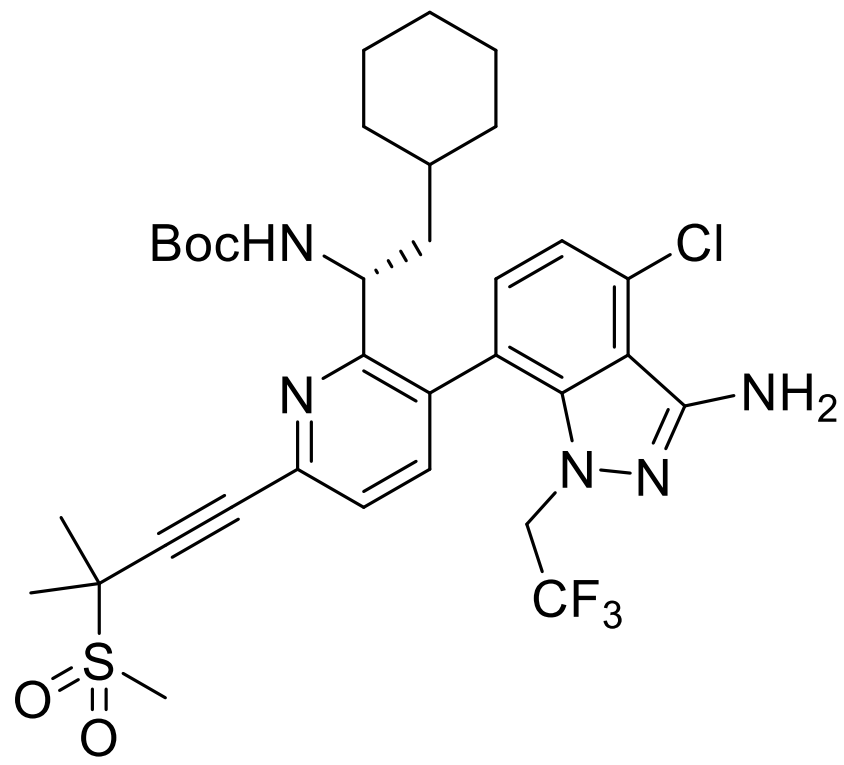


The title compound was prepared according to general procedure G using the enantiomer cyclopropyl subunit B **30dR** (292 mg, 0.554 mmol, 1.0 equiv.). Product was purified by CombiFlash on silica gel (0-40% Hexane/EtOAc) (201 mg, 52%, white solid). Observation of 2 atropoisomers on NMR. **$^1$H NMR** (400 MHz, $CDCl_3$) δ 7.61 – 7.52 (m, 1H), 7.43 (d, *J* = 7.9 Hz, 1H), 7.21 – 7.09 (m, ~0.4H), 7.06 (d, *J* = 7.6 Hz, ~0.6H), 6.97 (d, *J* = 7.4 Hz, ~0.4H), 6.90 (d, *J* = 7.6 Hz, ~0.6H), 5.50 (d, *J* = 9.8 Hz, ~0.3H), 5.38 (d, *J* = 9.5 Hz, ~0.7H), 4.84 – 4.61 (m, 2H), 4.59 – 4.42 (m, 1H), 4.40 – 4.17 (m, 1H), 3.99 (dq, *J* = 16.5, 8.3 Hz, ~0.6H), 3.92 – 3.81 (m, ~0.4H), 3.15 (s, 3H), 1.82 (s, 6H), 1.56 – 0.96 (m, 20H), 0.80 – 0.64 (m, 1H), 0.41 – 0.14 (m, 1H); **$^{13}$C NMR** (100 MHz, $CDCl_3$) δ 161.7, 155.4, 148.8, 142.3, 140.4, 138.8, 131.0, 130.4, 127.9, 126.0, 119.9, 118.9, 79.5, 75.2, 58.1, 50.2, 44.9, 35.4, 34.0, 32.1, 28.5, 28.1, 26.5, 26.4, 26.3, 26.1, 25.0, 22.9.

**tert-butyl (S)-(1-(3-(4-chloro-3-(N-(methylsulfonyl)methylsulfonamido)-1-(2,2,2-trifluoroethyl)-1H-indazol-7-yl)-6-(3-methyl-3-(methylsulfonyl)but-1-yn-1-yl)pyridin-2-yl)-2-cyclohexylethyl)carbamate (39dS)**

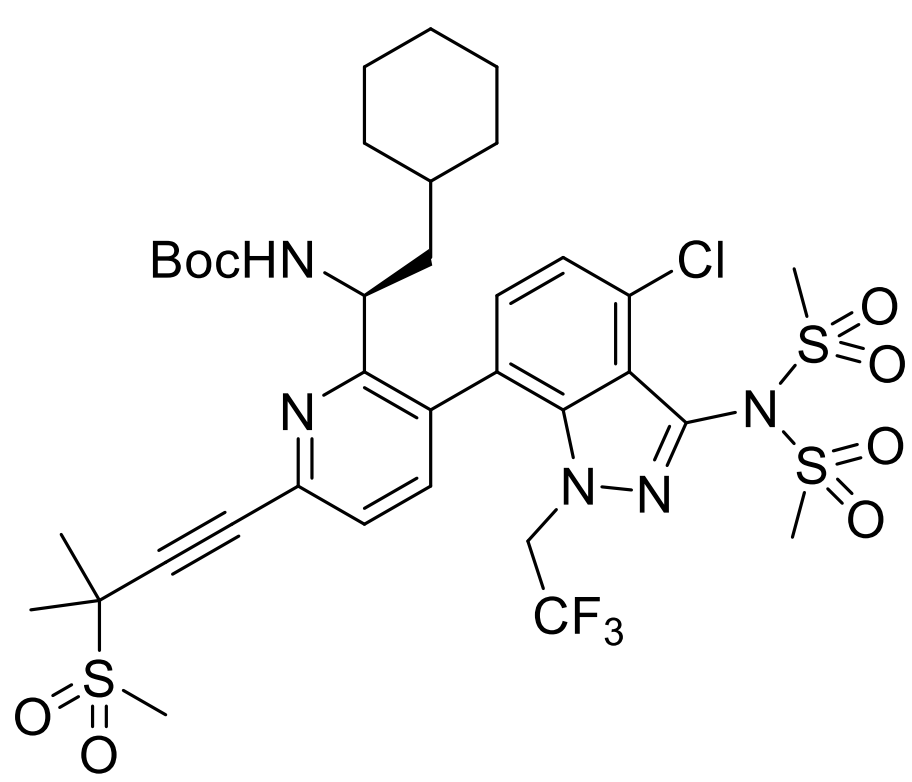


The title compound was prepared according to general procedure H using compound **38dS** (110 mg, 0.158 mmol, 1.0 equiv.). Product was purified by CombiFlash on silica gel (0-30% Hexane/EtOAc) (103 mg, 77%, white solid). **$^1$H NMR** (400 MHz, $CDCl_3$) δ 7.57 (d, *J* = 7.9 Hz, 1H), 7.46 (d, *J* = 8.3 Hz, 1H), 7.36 (d, *J* = 7.8 Hz, 1H), 7.07 (d, *J* = 7.6 Hz, 1H), 5.67 (br s, ~0.1H), 5.28 – 5.13 (m, ~0.8H), 4.76 – 4.28 (m, 3H), 3.63 (s, 3H), 3.59 (s, 3H), 3.15 (d, *J* = 1.0 Hz, 3H), 1.82 (s, 6H), 1.59 – 1.49 (m, 2H), 1.47 – 1.26 (m, 12H), 1.22 – 0.87 (m, 6H), 0.71 (br q, *J* = 11.7 Hz, 1H), 0.18 – -0.05 (m, 1H); **$^{13}$C NMR** (100 MHz, $CDCl_3$) δ 162.0, 155.7, 142.6, 141.5, 140.6, 138.8, 131.7, 126.8, 125.9, 123.8, 120.3, 120.2, 87.8, 85.6, 79.6, 58.1, 50.8, 44.0, 43.2, 42.9, 42.6, 42.3, 35.4, 34.1, 31.7, 28.5, 26.4,

26.3, 26.0, 25.8, 24.8, 23.5, 22.9; **HRMS** (ESI$^+$) calculated with loss of the Boc protection $C_{30}H_{38}ClF_3N_5O_6S_3$ [M-Boc+H]$^+$: 752.1620, found 752.1659.

**tert-butyl (R)-(1-(3-(4-chloro-3-(N-(methylsulfonyl)methylsulfonamido)-1-(2,2,2-trifluoroethyl)-1H-indazol-7-yl)-6-(3-methyl-3-(methylsulfonyl)but-1-yn-1-yl)pyridin-2-yl)-2-cyclohexylethyl)carbamate (39dR)**

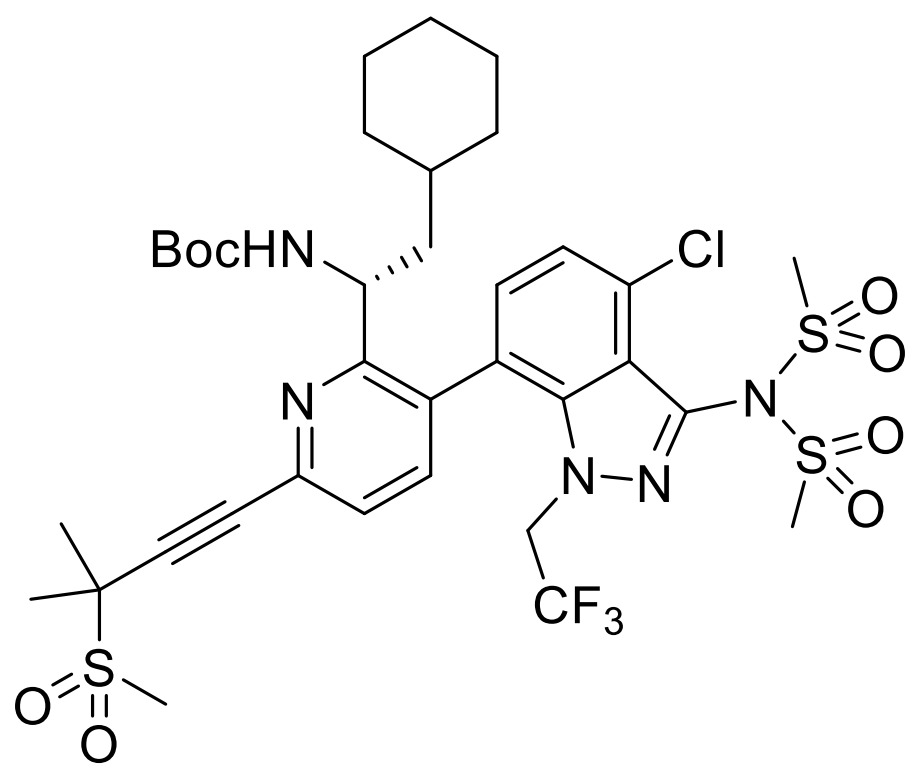


The title compound was prepared according to general procedure H using compound **38dR** (201 mg, 0.289 mmol, 1.0 equiv.). Product was purified by CombiFlash on silica gel (0-30% Hexane/EtOAc) (164 mg, 33%, white solid). **$^1$H NMR** (400 MHz, $CDCl_3$) identical to **39d**; **$^{13}$C NMR** (100 MHz, $CDCl_3$) : identical to **39d**.

**N-((S)-1-(3-(4-chloro-3-(methylsulfonamido)-1-(2,2,2-trifluoroethyl)-1H-indazol-7-yl)-6-(3-methyl-3-(methylsulfonyl)but-1-yn-1-yl)pyridin-2-yl)-2-cyclohexylethyl)-2-((3bR,4aS)-5,5-difluoro-3-(trifluoromethyl)-3b,4,4a,5-tetrahydro-1H-cyclopropa[3,4]cyclopenta[1,2-c]pyrazol-1-yl)acetamide (4S)**

The title compound was prepared according to general procedure I, using compound **39dS** (102 mg, 0.120 mmol, 1.0 equiv.). Product was purified by CombiFlash on silica gel (0-50% Hexane/EtOAc) (60 mg, 53% over two steps, white solid. 98% pure, evaluated on HPLC-UV sig=250 nm). The major atropoisomer was separated on preparative HPLC, but it partially converted into the minor atropoisomer, reaching a ratio of ≈90:10 as observed on NMR.

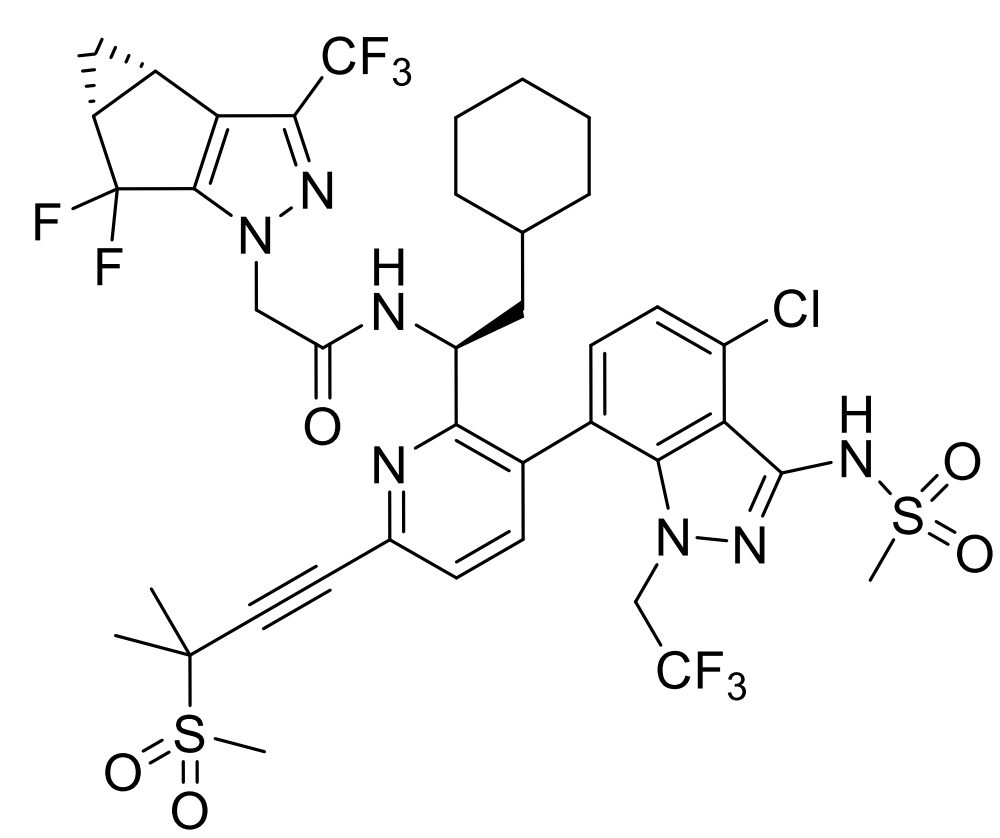


**$^1$H NMR** (400 MHz, CDCl3) δ ppm: 7.60 (d, $J$ = 6.3 Hz, 0.1H), 7.57 (d, $J$ = 7.9 Hz, 0.9H), 7.48 (d, $J$ = 7.9 Hz, 1H), 7.44 (s, 1H), 7.20 (d, $J$ = 7.6 Hz, 1H), 7.00 (d, $J$ = 7.6 Hz, 1H), 6.59 (d, $J$ = 8.4 Hz, 0.9H, major atropoisomer), 6.45 (d, $J$ = 9.0 Hz, 0.1H, minor

atropoisomer), 4.80 – 4.63 (m, 3H), 4.52 (dq, *J* = 16.2, 8.1 Hz, 0.9H, major atropoisomer), 4.47 – 4.37 (m, 0.1H, minor atropoisomer), 4.00 (dq, *J* = 16.1, 8.0 Hz, 1H), 3.41 (s, 3H), 3.15 (s, 3H), 2.53 – 2.43 (m, 2H), 1.82 (s, 6H), 1.60 – 1.38 (m, 6H), 1.35 – 1.18 (m, 4H), 1.16 – 0.82 (m, 8H), 0.71 (qd, *J* = 12.0, 3.3 Hz, 1H), 0.24 (qd, *J* = 11.8, 3.6 Hz, 1H); **$^{13}$C NMR** (100 MHz, CDCl3) δ 164.3, 160.4, 143.9, 143.6 (t, *J* = 29.4 Hz), 143.4, 142.8, 140.9, 140.0, 139.7, 139.0, 137.03, 136.6, 132.9, 131.5, 129.7, 127.1, 126.4, 124.3, 121.7, 120.0, 119.2, 115.1, 88.1, 85.2, 58.1, 53.8, 51.6, 51.2, 50.9, 49.3, 44.1, 41.7, 35.4, 34.1, 34.0, 31.8, 31.7, 28.2 (dd, *J* = 34.6, 29.2 Hz), 26.3, 26.1, 25.8, 24.8, 23.6, 23.5, 22.8, 22.8, 12.4; **$^{19}$F NMR** (376 MHz, $CDCl_3$) δ -61.52 (minor atropoisomer), -61.58 (major atropoisomer), -69.39 (t, *J* = 7.8 Hz, minor atropoisomer), -70.27 (t, *J* = 8.0 Hz, major atropoisomer), -81.05 (dd, *J* = 256.0, 13.4 Hz, major atropoisomer), -81.40 (dd, *J* = 255.5, 13.5 Hz, minor atropoisomer), -103.28 (dd, *J* = 255.9, 9.0 Hz, minor atropoisomer), -103.44 (dd, *J* = 256.1, 8.7 Hz, major atropoisomer); **HRMS** ($ESI^+$) Calculated for $C_{39}H_{41}ClF_8N_7O_5S_2$ $[M+H]^+$ 938.2166; found 938.2157.

**N-((R)-1-(3-(4-chloro-3-(methylsulfonamido)-1-(2,2,2-trifluoroethyl)-1H-indazol-7-yl)-6-(3-methyl-3-(methylsulfonyl)but-1-yn-1-yl)pyridin-2-yl)-2-cyclohexylethyl)-2-((3bR,4aS)-5,5-difluoro-3-(trifluoromethyl)-3b,4,4a,5-tetrahydro-1H-cyclopropa[3,4]cyclopenta[1,2-c]pyrazol-1-yl)acetamide (4R).**

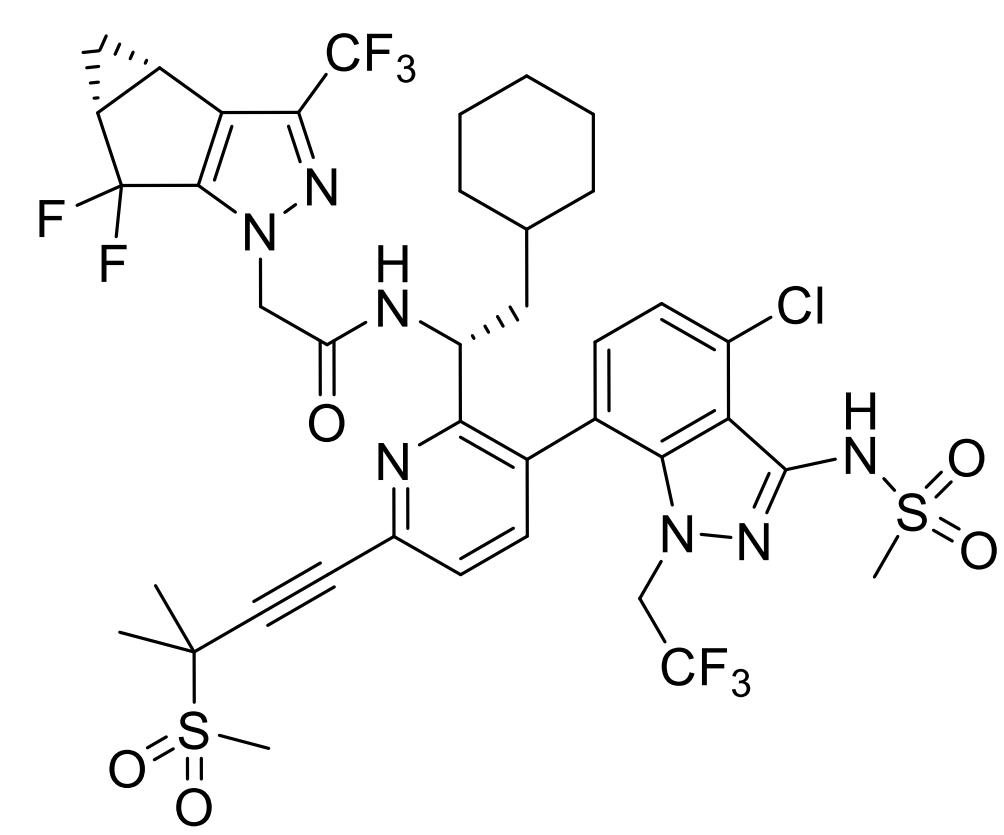


The title compound was prepared according to general procedure I, using compound **39dR** (163 mg, 0.190 mmol, 1.0 equiv.). Product was purified by CombiFlash on silica gel (0-50% Hexane/EtOAc) (96 mg, 54% over two steps, white solid, 98% pure, evaluated on HPLC-UV sig=250 nm). The major atropoisomer was separated, but it partially converted into the minor atropoisomer, reaching a ratio of ~90:10 as observed on NMR.

**$^1$H-NMR** (400 MHz, CDCl3) δ ppm: 7.57 (d, *J* = 7.9 Hz, 1H), 7.48 (d, *J* = 7.9 Hz, 1H), 7.44 (s, 0.9H, major atropoisomer), 7.29 (d, *J* = 1.4 Hz, 0.1H, minor atropoisomer), 7.21 (d, *J* = 7.7 Hz, 1H), 7.00 (d, *J* = 7.7 Hz, 1H), 6.58 (d, *J* = 8.6 Hz, 1H), 4.84 – 4.66 (m, 3H), 4.54 (dq, *J* = 16.3, 8.1 Hz, 0.9H), 4.43 (dq, *J* = 15.0, 7.2 Hz, 0.1H), 3.98 (dq, *J* = 16.0, 8.0 Hz, 1H), 3.41 (s, 3H), 3.15 (s,

3H), 2.53 – 2.42 (m, 2H), 1.82 (s, 6H), 1.60 – 1.38 (m, 4H), 1.35 – 0.82 (m, 9H), 0.71 (qd, *J* = 12.2, 3.3 Hz, 1H), 0.24 (q, *J* = 12.0 Hz, 0.9H), 0.17 – 0.05 (m, 0.1H); **$^{13}$C NMR** (100 MHz, CDCl3) δ 164.2, 160.4, 143.7 (t, *J* = 29.8 Hz), 142.8, 140.0, 139.7, 139.1, 132.9, 131.5, 129.7, 127.1, 126.4, 121.7, 119.1, 115.1, 88.3, 85.2, 58.1, 53.8, 51.2, 50.9, 49.2, 44.2, 41.7, 35.4, 34.0, 34.0, 31.8, 28.2 (dd, *J* = 34.7, 29.4 Hz), 26.3, 26.1, 25.8, 23.5, 22.8, 22.8, 14.3, 12.4; **$^{19}$F NMR** (376 MHz, $CDCl_3$) δ -61.51 (minor atropoisomer), -61.56 (major atropoisomer), -81.11 (dd, *J* = 255.8, 14.0 Hz, minor atropoisomer), -81.17 (dd, *J* = 256.2, 13.5 Hz, major atropoisomer), -103.58 (dd, *J* = 256.4, 8.6 Hz, major atropoisomer), -103.80 (dd, *J* = 256.1, 8.7 Hz, minor atropoisomer); **HRMS** ($ESI^+$) Calculated for $C_{39}H_{41}ClF_8N_7O_5S_2$ $[M+H]^+$ 938.2166; found 938.8979.

## 2. Thermal shift assays (TSAs)

TSAs used purified covalently-crosslinked hexameric CA containing A14C/E45C/W184A/M185A (CA121) either as wild-type (WT) or containing an M66I mutation. CA121 cloned in a pET11a expression plasmid kindly provided by Dr. Owen Pornillos (University of Virginia, Charlottesville, VA, USA). CA121 was expressed in BL21(DE3)RIL *E. coli* and purified according to reported protocols.[6] The TSAs were performed as previously described,[7-16] with each reaction containing 7.5 µM WT or M66I CA121 in 50 mM sodium phosphate buffer (pH 8.0), 1× Sypro Orange Protein Gel Stain (Life Technologies, Carlsbad, CA, USA), and either 1% DMSO (control) or 20 µM compound (1% DMSO final). The plate was heated from 25 to 95 °C with a heating rate of 0.2 °C every 10 s in the QuantStudio 3 Real-Time PCR system (Thermo Fisher Scientific, Waltham, MA, USA). The fluorescence intensity was measured with an Ex range of 475–500 nm and Em range of 520–590 nm. The difference in the melting temperature ($\Delta T_m$) of CA121 in DMSO ($T_0$) verses in the presence of compound ($T_m$) were calculated using the following Eq. (1):

$$\Delta T_m\ (°C) = T_m - T_0 \quad (1).$$

## 3. Virus production

The wild-type laboratory HIV-1 strain, HIV-$1_{NL4-3}$,[17] was produced using a pNL4-3 vector (NIH AIDS Reagent Program, Division of AIDS, NIAID, NIH, Bethesda, MD, USA). The M66I variant was produced by site-directed mutagenesis of the pNL4-3 WT vector in the capsid region. WT or M66I HIV-$1_{NL4-3}$ was generated by transfecting HEK 293FT cells with 10 µg of pNL4-3 vector and FuGENE®HD Transfection Reagent (Promega, Madison, WI, USA) in a T75 flask. Supernatant was harvested 48−72 h post-transfection and transferred to MT2 cells for viral propagation. Virus was harvested upon observation of syncytia formation, typically after 3−5 days. The viral supernatant was then concentrated using 8% w/v PEG 8000 overnight at 4 °C, followed by centrifugation for 40 min at 3,500 rpm. The resulting viral-containing pellet was concentrated 10-fold by resuspension in DMEM without FBS and stored at −80 °C.

## 4. Anti-HIV-1 and cytotoxicity assays

Anti-HIV-1 activity of compounds was examined in TZM-GFP cells. The potency of WT and M66I HIV-1 inhibition was determined based on the inhibition of viral LTR-activated GFP expression in the presence of compounds compared to DMSO controls. Briefly, TZM-GFP cells were plated at density of $1\times10^4$ cells per well in a 96-well plate. After 24 h, media was replaced with increasing concentrations of compound. Cells were exposed to WT or M66I HIV-$1_{NL4-3}$ (MOI=1) 24 h post treatment. After 48 h incubation, anti-HIV-1 activity was determined by counting the amount of GFP positive cells on a Cytation™ 5 Imaging Reader (BioTek, Winooski, VT, USA) and 50% effective concentration ($EC_{50}$) values were determined. TZM-GFP cells treated with compounds to assess antiviral activity were also tested in parallel for cytotoxicity. At the end of treatment duration, cell viability was assessed using an XTT assay kit (Roche) according to the manufacturer's instructions. Compound-induced toxicity manifested as a decrease in cell viability, normalized against the cell viability of the DMSO-treated cells. Values were plotted in GraphPad Prism 5 to obtain the $CC_{50}$ values. All cell-based assays were conducted in duplicate and in at least two independent experiments. Final values were calculated for each independent assay and average values for all assays were calculated.

## 5. Molecular modeling

Two experimental structures of CA were used for molecular modeling: 4XFZ[18] and 6V2F.[2] Both structures were prepared using the protein preparation workflow in Schrödinger Maestro (release 2025-3, Schrödinger Inc., New York, USA),[19] which includes filling of missing hydrogens, side chains, and loops. The resulting hydrogen bonding network was then optimized, and the heavy atoms converged to root mean squared deviation of 0.3 Å using the OPLS4 force field.[20]

Induced fit docking was performed using the induced fit docking protocol using both Glide (Schrödinger Inc., New York, USA)[21] and Prime (Schrödinger Inc., New York, USA).[22] The cognate ligand of 4XFZ was selected to be the center of receptor grid in induced fit docking. The ring conformations were sampled using energy window of 2.5 kcal/mol. For docking, receptor (CA) van der Waals scaling was set to be 0.5. Ligand van der Waals scaling was set to be 0.5. The residues within 5 Å of the ligand were refined using Prime.

## 6. ADME assays

Aqueous solubility assay: The aqueous solubilities of compounds were evaluated in Dulbecco's Phosphate Buffered Saline (DPBS, pH 7.2) using kinetic solubility study. A 3 µL from 100 mM dimethyl sulfoxide (DMSO) stock solution was added to 297 µL of DPBS to generate a saturated suspension. The mixture was equilibrated at ambient temperature for 2 h to allow for dissolution. Thereafter, the suspension was filtered through a 0.45 µm polyvinylidene fluoride (PVDF) syringe filter to remove undissolved material. The filtrate was subsequently analyzed by liquid chromatography–tandem mass spectrometry (LC–MS/MS) using an Agilent 1260 Infinity HPLC system (Agilent Technologies, Santa Clara, CA, USA) coupled with AB Sciex QTrap 5500 mass spectrometer (AB Sciex LLC, Toronto, ON, Canada).

Plasma stability assay: The plasma stability of compounds was assessed in Wistar han rat plasma (BioIVT, Hicksville, NY, USA) and human plasma (Innovative Research, Novi, MI, USA). Plasma was diluted to 80% with 0.1 mol/L potassium phosphate buffer (pH 7.4) before incubation. Each compound was incubated at a final concentration of 1 µM and incubation was carried out at

37 °C. At predetermined time points (0, 1, 3, 6, and 24 h), 50 µL aliquots of the plasma were withdrawn and immediately quenched with 200 µL of acetonitrile containing 0.1% formic acid. The samples were vortex-mixed and subsequently centrifuged at 15,000 rpm for 5 min at 4 °C. The resulting supernatants were analyzed by LC–MS/MS to determine the in vitro plasma half-life ($t_{1/2}$) of each compound.

Microsomal stability assay: Microsomal stabilities (Phase I) of compounds were performed using commercially available human and Wistar rat liver microsomes (XenoTech, Kansas City, KS, USA). Briefly, test compounds were added at a final concentration of 1 µM to the reaction mixture containing liver microsomal protein (0.5 mg/mL final concentration) and $MgCl_2$ (1 mM final concentration) in 0.1 M potassium phosphate buffer (pH 7.4). The metabolic reaction was initiated by the addition of nicotinamide adenine dinucleotide phosphate (NADPH, metabolic cofactor) at a final concentration of 1 mM and maintained at 37 °C. Positive control (verapamil) and negative control (without NADPH) were run in parallel to confirm microsomal activity and non-NADPH degradation respectively. At predetermined time points, 50 µL aliquots of the reaction mixture were withdrawn and immediately quenched with 200 µL of acetonitrile containing 0.1% formic acid. Samples were vortex-mixed and centrifuged at 15,000 rpm for 5 min at 4 °C. The resulting supernatants were collected and analyzed by LC–MS/MS to determine the in vitro metabolic half-life ($t_{1/2}$).

Parallel Artificial Membrane Permeability Assay (PAMPA): The passive permeability of selected compounds was evaluated using the Corning BioCoat Precoated PAMPA Plate System (catalog No. 353015, Corning, Glendale, AZ, USA). The precoated plate assembly, stored at −20 °C, was equilibrated to room temperature for 30 min prior to use. The assay was performed according to the manufacturer's protocol. Test compounds were prepared by diluting 10 mM DMSO stock solutions to a final concentration of 10 µM in 10% methanol in Dulbecco's Phosphate Buffered Saline (DPBS, pH 7.2). A 300 µL of each compound solution was added to the wells of the receiver (donor) plate, and 200 µL of 10% methanol in DPBS was added to wells of precoated filter (acceptor) plate. The assembled plates were kept at room temperature without agitation for 5 h to permit passive diffusion across the artificial membrane. After incubation, the plates were separated, and compound concentrations in both donor and acceptor wells were quantified by LC–MS/MS analysis. The apparent permeability of each compound was calculated using standard Eq. (2).

$$Pe = \{-ln\ [1-C_A(t)/C_{eq}]\}/[A\times(1/V_D+1/V_A)\times t] \quad (2)$$

where A = filter area (0.3 $cm^2$), $V_D$ = donor well volume (0.3 mL), $V_A$ = acceptor well volume (0.2 mL), $t$ = incubation time (seconds), $C_A(t)$ = compound concentration in acceptor well at time $t$, $C_D(t)$ = compound concentration in donor well at time $t$, and $C_{eq}$ was calculated using the Eq. (3):

$$C_{eq} = [C_D(t)\times V_D+C_A(t)\times V_A]/(V_D+V_A) \quad (3)$$

A cutoff criteria of *Pe* value at $1.5 \times 10^{-6}$ cm/s was used to classify the compounds into high and low permeability according to the literature report of this PAMPA plate system.[23]

Plasma protein binding: Plasma protein binding was evaluated using a Rapid Equilibrium Dialysis (RED) plate (Thermo Scientific, Research triangle park, NC, USA). Test compounds were added to pooled human (Innovative Research, Novi, MI, USA) and pooled wistar han rat plasma (BioIVT, Hicksville, NY, USA) at a concentration of 1 μM, maintaining a final DMSO concentration of 1%. Aliquot of 200 μL spiked plasma was added into the red chamber of the RED plate in duplicate, while 350 μL of Dulbecco's phosphate-buffered saline (DPBS) was added in the adjacent white chamber. The plate was sealed and incubated at 37 °C for 5 h with shaking at 200 rpm. Following incubation, samples were collected from both plasma and buffer chambers, and compound concentrations were quantified via LC-MS/MS using matrix-matched calibration standards. The fraction of compound bound to plasma proteins was determined according to Eq. (4).

$$Fraction\ bound\ (\%) = (C_p-C_d)/C_p \times 100 \quad (4)$$

Where, $C_p$ is the concentration in plasma chamber and $C_d$ is the concentration in DPBS buffer chamber. In addition, the percentage of recovery was calculated using Eq. (5) to ensure the robustness of the measurements.

$$Recovery\ (\%) = (C_p \times 0.2 + C_d \times 0.35)/(C_0 \times 0.2) \times 100 \quad (5)$$

In Eq. (5), $C_0$ is the plasma concentration before spiking. Acceptable recovery was set arbitrarily between 70% and 130%.

## 7. *In vivo* pharmacokinetics studies

The pharmacokinetic study of LEN and **3S** was performed in male Wistar rats, sourced from Charles River Laboratories (Wilmington, MA, USA). Experimental protocol (IACUC protocol no. 2407-42177A) was approved by the Institutional Animal Care and Use Committee at the University of Minnesota, USA. Rats were randomly divided into two groups (n=4 per group) and administered LEN (1 mg/kg) or **3S** (1 mg/kg) by intravenous route. For dosing, the compound was dissolved at 0.5 mg/mL concentration in 5% ethanol, 20% propylene glycol, 45% polyethylene glycol (PEG 300), and 30% DPBS. Blood samples (~20 µL) were serially collected into EDTA-coated tubes from each animal at designated time points (0.083, 0.25, 0.5, 1, 2, 4, 8, 24, 48, 72, 96, and 120 h post-dose) via saphenous vein puncture. Plasma was separated by centrifugation at 3000 rpm for 15 min and stored at −80 °C until further analysis.

## 8. X-ray crystallography data

Crystallization: Crystals of **28aS** suitable for X-ray diffraction analysis were obtained by slow evaporation from a dichloromethane/hexane solvent system.

Data collection: A crystal (approximate dimensions 0.200 x 0.140 x 0.030 $mm^3$) was placed onto the tip of a 150 µm diameter MiTeGen Dual-Thickness Microloop and mounted on a Bruker D8 PHOTON-III CPAD diffractometer for a data collection at 100(2) K.[24] A preliminary set of cell constants was calculated from reflections harvested from three sets of frames. These initial sets of frames were oriented such that orthogonal wedges of reciprocal space were surveyed. This produced an initial orientation matrix determined from 297 reflections. The data collection was carried out using MoKα radiation (parabolic mirrors) with a frame time of 6 seconds and a detector distance of 5.0 cm. A strategy program was used to assure complete coverage of all unique data to a resolution of 0.72 Å. All major sections of frames were collected with 1.20º steps in ω or ϕ at different detector positions in 2θ. The intensity data were corrected for absorption and decay (SADABS).[25] Final cell constants were calculated from 2889 strong reflections from the actual data collection after integration (SAINT).[26] Please refer to Table S1 for additional crystal and refinement information.

Structure solution and refinement: The structure was solved using SHELXT-2018/2 and refined

using SHELXL-2019/2.[27-30] The space group $P2_1$ was determined based on systematic absences and intensity statistics. A direct-methods solution was calculated which provided most non-hydrogen atoms from the E-map. Full-matrix least squares / difference Fourier cycles were performed which located the remaining non-hydrogen atoms. All non-hydrogen atoms were refined with anisotropic displacement parameters. All hydrogen atoms were placed in ideal positions and refined as riding atoms with relative isotropic displacement parameters. The final full matrix least squares refinement converged to $R1 = 0.0154$ and $wR2 = 0.0401$ ($F^2$, obs. data). Equations (6-10) shown below are for X-ray statistics in Table S1.

$$R_1 = \Sigma||F_o|-|F_c|| / \Sigma|F_o| \quad (6)$$

$$wR2 = [\Sigma[w(F_o^2-F_c^2)^2] / \Sigma[w(F_o^2)^2]]^{1/2} \quad (7)$$

$$\text{where } w = q / [\sigma^2(F_o^2) + (a*P)^2 + b*P + d + e*sin(\theta)] \quad (8)$$

$$R_{int} = \Sigma|F_o^2 - \langle F_o^2 \rangle| / \Sigma|F_o^2| \quad (9)$$

$$Goodness\text{-}of\text{-}Fit\ (GooF) = S = [\Sigma[w(F_o^2-F_c^2)^2] / (n-p)]^{1/2} \quad (10)$$

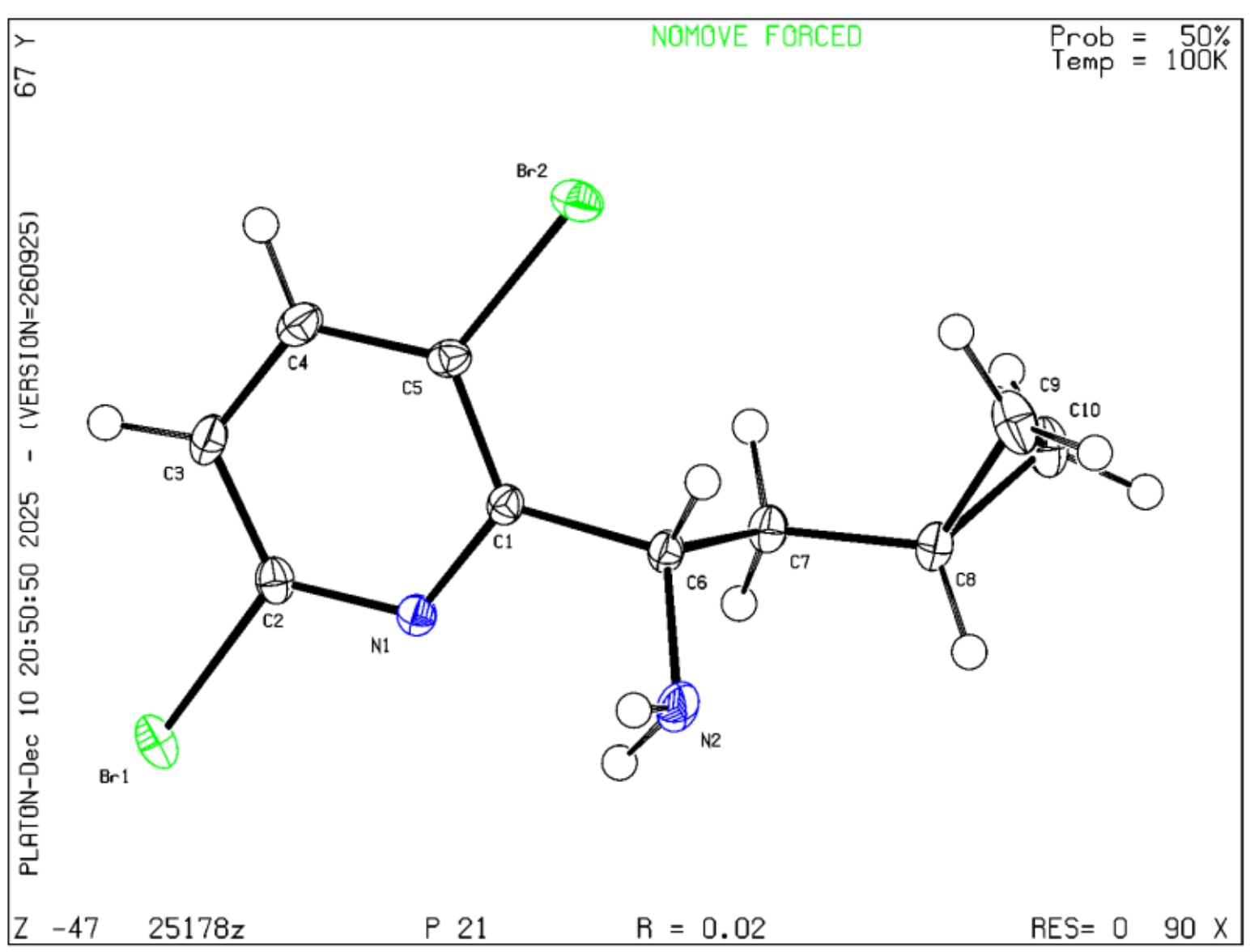


X-ray crystal structure of **28aS** (25178z). Ellipsoid contour is shown at 50% probability level.[31-34]

## References


[1] N. Kadunce, O. St-Jean, A. Cagulada, W.-T. T. Chang, A. F. G. Goldberg, Z. Huang, J. Kang, J. Kim, K. Kwon, O. Lapina, L. Lin, T. J. Rainey, L. Roever, N. P. Shah, S. A. Wolckenhauer, *Org. Process Res. Dev.* **2024**, *28*, 3368-3381.

[2] S. M. Bester, G. Wei, H. Zhao, D. Adu-Ampratwum, N. Iqbal, V. V. Courouble, A. C. Francis, A. S. Annamalai, P. K. Singh, N. Shkriabai, P. Van Blerkom, J. Morrison, E. M. Poeschla, A. N. Engelman, G. B. Melikyan, P. R. Griffin, J. R. Fuchs, F. J. Asturias, M. Kvaratskhelia, *Science* **2020**, *370*, 360-364.

[3] A. D. J. Calow, D. Dailler, J. F. Bower, *J. Am. Chem. Soc.* **2022**, *144*, 11069-11074.

[4] A. H. Shinde, R. Sayini, P. Singh, J. M. Burns, S. Ahmad, G. M. Laidlaw, B. F. Gupton, D. A. Klumpp, L. Jin, *J. Org. Chem.* **2025**, *90*, 471-478.

[5] G. Chelucci, S. Baldino, S. Chessa, G. A. Pinna, F. Soccolini, *Tetrahedron: Asymmetry* **2006**, *17*, 3163-3169.

[6] O. Pornillos, B. K. Ganser-Pornillos, B. N. Kelly, Y. Hua, F. G. Whitby, C. D. Stout, W. I. Sundquist, C. P. Hill, M. Yeager, *Cell* **2009**, *137*, 1282-1292.

[7] W. M. McFadden, M. C. Casey-Moore, G. A. L. Bare, K. A. Kirby, X. Wen, G. Li, H. Wang, R. L. Slack, A. A. Snyder, Z. C. Lorson, I. L. Kaufman, M. E. Cilento, P. R. Tedbury, M. Gembicky, A. J. Olson, B. E. Torbett, K. B. Sharpless, S. G. Sarafianos, *Cell Chem Biol* **2024**, *31*, 477-486 e477.

[8] R. L. Sahani, T. Akther, M. E. Cilento, A. E. Castaner, H. Zhang, K. A. Kirby, J. Xie, S. G. Sarafianos, Z. Wang, *RSC Med Chem* **2021**, *12*, 2031-2044.

[9] R. L. Sahani, R. Diana-Rivero, S. K. V. Vernekar, L. Wang, H. Du, H. Zhang, A. E. Castaner, M. C. Casey, K. A. Kirby, P. R. Tedbury, J. Xie, S. G. Sarafianos, Z. Wang, *Viruses* **2021**, *13*.

[10] L. Wang, M. C. Casey, S. K. V. Vernekar, R. L. Sahani, K. A. Kirby, H. Du, H. Zhang, P. R. Tedbury, J. Xie, S. G. Sarafianos, Z. Wang, *Acta Pharm Sin B* **2021**, *11*, 810-822.

[11] L. Wang, M. C. Casey, S. K. V. Vernekar, R. L. Sahani, J. Kankanala, K. A. Kirby, H. Du, A. Hachiya, H. Zhang, P. R. Tedbury, J. Xie, S. G. Sarafianos, Z. Wang, *Eur J Med Chem* **2020**, *204*, 112626.

[12] L. Wang, M. C. Casey, S. K. V. Vernekar, H. T. Do, R. L. Sahani, K. A. Kirby, H. Du, A. Hachiya, H. Zhang, P. R. Tedbury, J. Xie, S. G. Sarafianos, Z. Wang, *Eur J Med Chem* **2020**, *200*, 112427.

[13] S. K. V. Vernekar, R. L. Sahani, M. C. Casey, J. Kankanala, L. Wang, K. A. Kirby, H. Du, H. Zhang, P. R. Tedbury, J. Xie, S. G. Sarafianos, Z. Wang, *Viruses* **2020**, *12*.

[14] M. C. Lo, A. Aulabaugh, G. Jin, R. Cowling, J. Bard, M. Malamas, G. Ellestad, *Anal Biochem* **2004**, *332*, 153-159.

[15] Y. Miyazaki, N. Doi, T. Koma, A. Adachi, M. Nomaguchi, *Front Microbiol* **2017**, *8*, 1413.

[16] M. W. Pantoliano, E. C. Petrella, J. D. Kwasnoski, V. S. Lobanov, J. Myslik, E. Graf, T. Carver, E. Asel, B. A. Springer, P. Lane, F. R. Salemme, *J Biomol Screen* **2001**, *6*, 429-440.

[17] A. Adachi, H. E. Gendelman, S. Koenig, T. Folks, R. Willey, A. Rabson, M. A. Martin, *J Virol* **1986**, *59*, 284-291.

[18] A. T. Gres, K. A. Kirby, V. N. KewalRamani, J. J. Tanner, O. Pornillos, S. G. Sarafianos, *Science* **2015**, *349*, 99-103.

[19] G. M. Sastry, M. Adzhigirey, T. Day, R. Annabhimoju, W. Sherman, *J Comput Aided Mol Des* **2013**, *27*, 221-234.

[20] C. Lu, C. Wu, D. Ghoreishi, W. Chen, L. Wang, W. Damm, G. A. Ross, M. K. Dahlgren, E. Russell, C. D. Von Bargen, R. Abel, R. A. Friesner, E. D. Harder, *J Chem Theory Comput* **2021**, *17*, 4291-4300.

[21] Y. Yang, K. Yao, M. P. Repasky, K. Leswing, R. Abel, B. K. Shoichet, S. V. Jerome, *J Chem Theory Comput* **2021**, *17*, 7106-7119.

[22] M. P. Jacobson, D. L. Pincus, C. S. Rapp, T. J. Day, B. Honig, D. E. Shaw, R. A. Friesner, *Proteins* **2004**, *55*, 351-367.

[23] X. Chen, A. Murawski, K. Patel, C. L. Crespi, P. V. Balimane, *Pharm Res* **2008**, *25*, 1511-1520.

[24] Bruker (2026). APEX (Version APEX5). Bruker AXS Inc., Madison, Wisconsin, USA.

[25] L. Krause, R. Herbst-Irmer, G. M. Sheldrick, D. Stalke, *J. Appl. Cryst.* **2015**, 48, 3-10.

[26] Bruker (2026). SAINT (Version 8.4). Bruker AXS Inc., Madison, Wisconsin, USA.

[27] G. M. Sheldrick, *Acta Cryst.* **2015**, C71, 3 – 8.

[28] G. M. Sheldrick, *Acta Cryst.* **2008**, A64, 112–122.

[29] G. M. Sheldrick, *Acta Cryst.* **2015**, A71, 3 – 8.

[30] C. B. Hübschle, G. M. Sheldricka, B. Dittricha, *J. Appl. Cryst.* **2011**, 44, 1281-1284.

[31] A. L. Spek, *Acta Cryst.* **2020**, E76, 1 – 11

[32] A. L. Spek, *Inorg. Chim. Acta* **2018**, 470, 232-237.

[33] A. L. Spek, *Acta Cryst.* **2009**, D65, 148-155.

[34] A. L. Spek, *J. Appl. Cryst.* **2003**, 36, 7-11.

Table S1. Crystal data and structure refinement for **28aS** (CCDC Database Code: 25178z).

| | | |
|---|---|---|
| CCDC identification code | 25178z | |
| Empirical formula | $C_{10} H_{12} Br_2$ N2 | |
| Formula weight | 320.04 | |
| Temperature | 100(2) K | |
| Wavelength | 0.71073 Å | |
| Crystal system | Monoclinic | |
| Space group | $P2_1$ | |
| Unit cell dimensions | $a$ = 4.8721(2) Å | a = 90° |
| | $b$ = 14.4560(6) Å | b = 105.101(2)° |
| | $c$ = 8.2498(4) Å | g = 90° |
| Volume | 560.98(4) $Å^3$ | |
| $Z$ | 2 | |
| Density (calculated) | 1.895 $Mg/m^3$ | |
| Absorption coefficient | 7.188 $mm^{-1}$ | |
| $F$(000) | 312 | |
| Crystal color, morphology | colourless, plate | |
| Crystal size | 0.200 x 0.140 x 0.030 $mm^3$ | |
| Theta range for data collection | 2.557 to 29.566° | |
| Index ranges | -6 £ $h$ £ 6, -20 £ $k$ £ 20, -11 £ $l$ £ 11 | |
| Reflections collected | 15791 | |
| Independent reflections | 3124 [$Rint$ = 0.0281] | |
| Observed reflections | 3076 | |
| Completeness to theta = 25.242° | 99.8% | |
| Absorption correction | Multi-scan | |
| Max. and min. transmission | 0.7459 and 0.4933 | |
| Refinement method | Full-matrix least-squares on $F^2$ | |
| Data / restraints / parameters | 3124 / 2 / 133 | |
| Goodness-of-Fit (GooF) on $F^2$ | 0.958 | |
| Final $R$ indices [$I$>2sigma($I$)] | $R1$ = 0.0154, $wR2$ = 0.0401 | |
| $R$ indices (all data) | $R1$ = 0.0159, $wR2$ = 0.0404 | |
| Absolute structure parameter | -0.006(3) | |
| Largest diff. peak and hole | 0.303 and -0.520 e.$Å^{-3}$ | |

Table S2. Atomic coordinates (x $10^4$) and equivalent isotropic displacement parameters ($Å^2$x $10^3$ ) for 25178z. $U_{eq}$ is defined as one third of the trace of the orthogonalized $U_{ij}$ tensor.

| | x | y | z | $U_{eq}$ |
|---|---|---|---|---|
| C1 | 3261(5) | 4620(2) | 3826(3) | 12(1) |
| N1 | 1258(4) | 3959(1) | 3710(2) | 13(1) |
| C2 | 797(5) | 3638(2) | 5121(3) | 14(1) |
| Br1 | -2077(1) | 2711(1) | 4860(1) | 18(1) |
| C3 | 2206(5) | 3921(2) | 6722(3) | 17(1) |
| C4 | 4221(5) | 4616(2) | 6855(3) | 17(1) |
| C5 | 4701(5) | 4969(2) | 5387(3) | 13(1) |
| Br2 | 7354(1) | 5947(1) | 5544(1) | 18(1) |
| C6 | 3747(5) | 4949(2) | 2171(3) | 13(1) |
| N2 | 3389(5) | 4222(2) | 901(3) | 23(1) |
| C7 | 1726(5) | 5760(2) | 1514(3) | 15(1) |
| C8 | 2330(5) | 6250(2) | 35(3) | 16(1) |
| C9 | 4898(6) | 6873(2) | 316(4) | 23(1) |
| C10 | 1939(5) | 7284(2) | -123(3) | 21(1) |

Table S3. Bond lengths [Å] and angles [°] for 25178z.

| | | | |
|---|---|---|---|
| C1-N1 | 1.352(3) | N2-H2A | 0.91(2) |
| C1-C5 | 1.391(3) | N2-H2B | 0.91(2) |
| C1-C6 | 1.522(3) | C7-C8 | 1.505(3) |
| N1-C2 | 1.324(3) | C7-H7A | 0.9900 |
| C2-C3 | 1.382(3) | C7-H7B | 0.9900 |
| C2-Br1 | 1.910(2) | C8-C10 | 1.507(3) |
| C3-C4 | 1.389(3) | C8-C9 | 1.509(3) |
| C3-H3 | 0.9500 | C8-H8 | 1.0000 |
| C4-C5 | 1.390(3) | C9-C10 | 1.513(3) |
| C4-H4 | 0.9500 | C9-H9A | 0.9900 |
| C5-Br2 | 1.897(2) | C9-H9B | 0.9900 |
| C6-N2 | 1.461(3) | C10-H10A | 0.9900 |
| C6-C7 | 1.536(3) | C10-H10B | 0.9900 |
| C6-H6 | 1.0000 | | |
| | | | |
| N1-C1-C5 | 120.19(19) | N2-C6-H6 | 108.0 |
| N1-C1-C6 | 115.86(19) | C1-C6-H6 | 108.0 |
| C5-C1-C6 | 123.9(2) | C7-C6-H6 | 108.0 |
| C2-N1-C1 | 118.01(19) | C6-N2-H2A | 108(3) |
| N1-C2-C3 | 125.5(2) | C6-N2-H2B | 105(2) |
| N1-C2-Br1 | 115.69(17) | H2A-N2-H2B | 108(3) |
| C3-C2-Br1 | 118.79(16) | C8-C7-C6 | 113.76(17) |
| C2-C3-C4 | 117.0(2) | C8-C7-H7A | 108.8 |
| C2-C3-H3 | 121.5 | C6-C7-H7A | 108.8 |
| C4-C3-H3 | 121.5 | C8-C7-H7B | 108.8 |
| C3-C4-C5 | 118.2(2) | C6-C7-H7B | 108.8 |
| C3-C4-H4 | 120.9 | H7A-C7-H7B | 107.7 |
| C5-C4-H4 | 120.9 | C7-C8-C10 | 119.4(2) |
| C4-C5-C1 | 121.0(2) | C7-C8-C9 | 119.1(2) |
| C4-C5-Br2 | 118.74(17) | C10-C8-C9 | 60.23(16) |
| C1-C5-Br2 | 120.26(16) | C7-C8-H8 | 115.6 |
| N2-C6-C1 | 113.66(18) | C10-C8-H8 | 115.6 |
| N2-C6-C7 | 110.47(19) | C9-C8-H8 | 115.6 |
| C1-C6-C7 | 108.63(16) | C8-C9-C10 | 59.84(15) |

| | | | |
|---|---|---|---|
| C8-C9-H9A | 117.8 | C8-C10-H10A | 117.8 |
| C10-C9-H9A | 117.8 | C9-C10-H10A | 117.8 |
| C8-C9-H9B | 117.8 | C8-C10-H10B | 117.8 |
| C10-C9-H9B | 117.8 | C9-C10-H10B | 117.8 |
| H9A-C9-H9B | 114.9 | H10A-C10-H10B | 114.9 |
| C8-C10-C9 | 59.93(16) | | |

Symmetry transformations used to generate equivalent atoms:

Table S4. Anisotropic displacement parameters ($Å^2$x $10^3$) for 25178z. The anisotropic displacement factor exponent takes the form: $-2\pi^2[ h^2 a^{*2}U_{11} + ... + 2 h k a^* b^* U_{12} ]$

| | $U_{11}$ | $U_{22}$ | $U_{33}$ | $U_{23}$ | $U_{13}$ | $U_{12}$ |
|---|---|---|---|---|---|---|
| C1 | 14(1) | 11(1) | 11(1) | 1(1) | 3(1) | 2(1) |
| N1 | 14(1) | 12(1) | 13(1) | 0(1) | 4(1) | 2(1) |
| C2 | 15(1) | 13(1) | 16(1) | 2(1) | 6(1) | 2(1) |
| Br1 | 18(1) | 12(1) | 25(1) | 4(1) | 10(1) | 1(1) |
| C3 | 22(1) | 18(1) | 12(1) | 3(1) | 7(1) | 4(1) |
| C4 | 21(1) | 17(1) | 12(1) | -1(1) | 3(1) | 4(1) |
| C5 | 14(1) | 12(1) | 13(1) | -2(1) | 2(1) | 2(1) |
| Br2 | 15(1) | 14(1) | 22(1) | -2(1) | 0(1) | -1(1) |
| C6 | 17(1) | 12(1) | 11(1) | 1(1) | 6(1) | 2(1) |
| N2 | 44(1) | 15(1) | 14(1) | -2(1) | 14(1) | 2(1) |
| C7 | 15(1) | 16(1) | 14(1) | 2(1) | 6(1) | 2(1) |
| C8 | 18(1) | 16(1) | 13(1) | 4(1) | 5(1) | 2(1) |
| C9 | 17(1) | 21(1) | 32(1) | 9(1) | 8(1) | 0(1) |
| C10 | 22(1) | 18(1) | 24(1) | 7(1) | 9(1) | 5(1) |

Table S5. Hydrogen coordinates (x $10^4$) and isotropic displacement parameters (Å$^2$ x $10^3$) for 25178z.

| | x | y | z | U(eq) |
|---|---|---|---|---|
| H3 | 1813 | 3652 | 7689 | 20 |
| H4 | 5244 | 4844 | 7922 | 20 |
| H6 | 5741 | 5185 | 2399 | 16 |
| H2A | 4870(60) | 3820(20) | 1230(50) | 35 |
| H2B | 1760(60) | 3920(20) | 950(50) | 35 |
| H7A | -250 | 5524 | 1187 | 18 |
| H7B | 1867 | 6211 | 2435 | 18 |
| H8 | 1850 | 5898 | -1045 | 19 |
| H9A | 5960 | 6882 | -556 | 27 |
| H9B | 6082 | 6950 | 1481 | 27 |
| H10A | 1320 | 7612 | 775 | 25 |
| H10B | 1197 | 7545 | -1263 | 25 |

Table S6. Torsion angles [°] for 25178z.

| | |
|---|---|
| C5-C1-N1-C2 | -2.2(3) |
| C6-C1-N1-C2 | 178.79(19) |
| C1-N1-C2-C3 | -0.3(3) |
| C1-N1-C2-Br1 | 179.65(15) |
| N1-C2-C3-C4 | 1.7(3) |
| Br1-C2-C3-C4 | -178.16(16) |
| C2-C3-C4-C5 | -0.7(3) |
| C3-C4-C5-C1 | -1.7(3) |
| C3-C4-C5-Br2 | 177.91(16) |
| N1-C1-C5-C4 | 3.3(3) |
| C6-C1-C5-C4 | -177.86(19) |
| N1-C1-C5-Br2 | -176.35(15) |
| C6-C1-C5-Br2 | 2.5(3) |
| N1-C1-C6-N2 | -34.0(3) |
| C5-C1-C6-N2 | 147.1(2) |
| N1-C1-C6-C7 | 89.4(2) |
| C5-C1-C6-C7 | -89.5(3) |
| N2-C6-C7-C8 | -64.3(2) |
| C1-C6-C7-C8 | 170.35(19) |
| C6-C7-C8-C10 | -143.9(2) |
| C6-C7-C8-C9 | -73.7(3) |
| C7-C8-C9-C10 | -109.2(2) |
| C7-C8-C10-C9 | 108.7(2) |

Enantiopurity verified by chiral HPLC of **(1R,5R,E)-3-(hydroxyimino)bicyclo[3.1.0]hexan-2-one (10)**

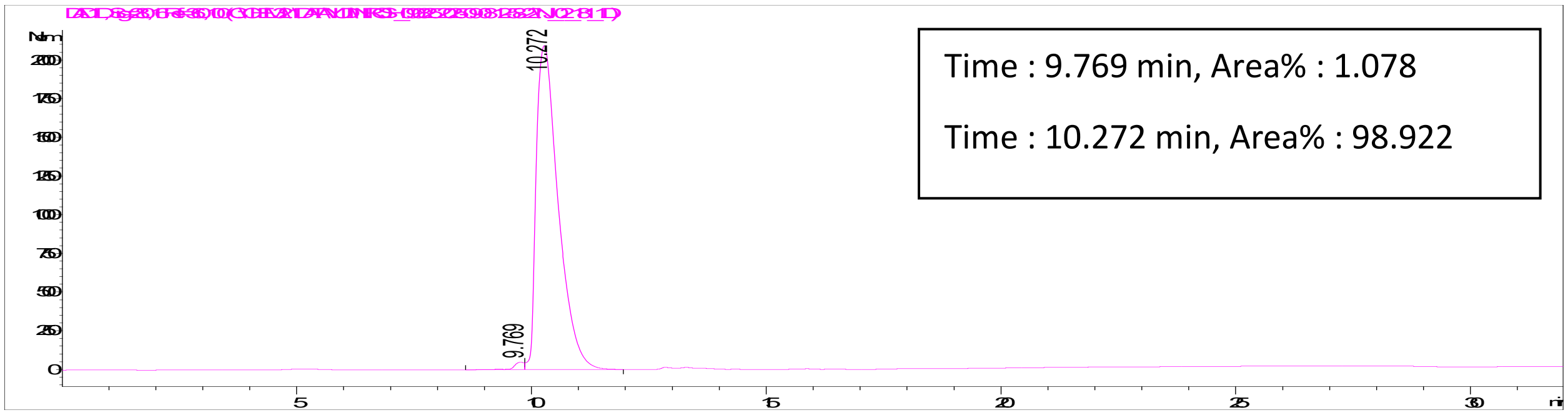


*Figure S1 : Chromatogram (chiral LC) of compound 10 to determine enantiopurity*

Gradient :

| Time (min) | B (%) | Flow (ml/min) |
|---|---|---|
| 0.00 | 5.0 | 1.00 |
| 3.00 | 5.0 | 1.00 |
| 25.00 | 100.0 | 1.00 |
| 30.00 | 100.0 | 1.00 |
| 32.00 | 5.0 | 1.00 |

Column :

P/No. C0G-4491-E0
Lux 5u Cellulose-4
New Column 250 x 4.6 mm
S/Nb. 710804-4
B/No. 5599-34

**Enantiopurity of Ethyl 2-((3bS,4aR)-3-(trifluoromethyl)-4,4a-dihydrospiro[cyclopropa[3,4]cyclopenta[1,2-c]pyrazole-5,2'-[1,3]dithiolane]-1(3bH)-yl)acetate (15) verified by progressive addition of Eu(hfc)$_3$ on proton NMR :**

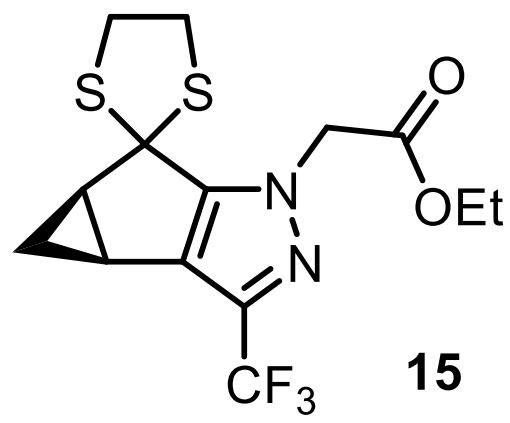


**15**

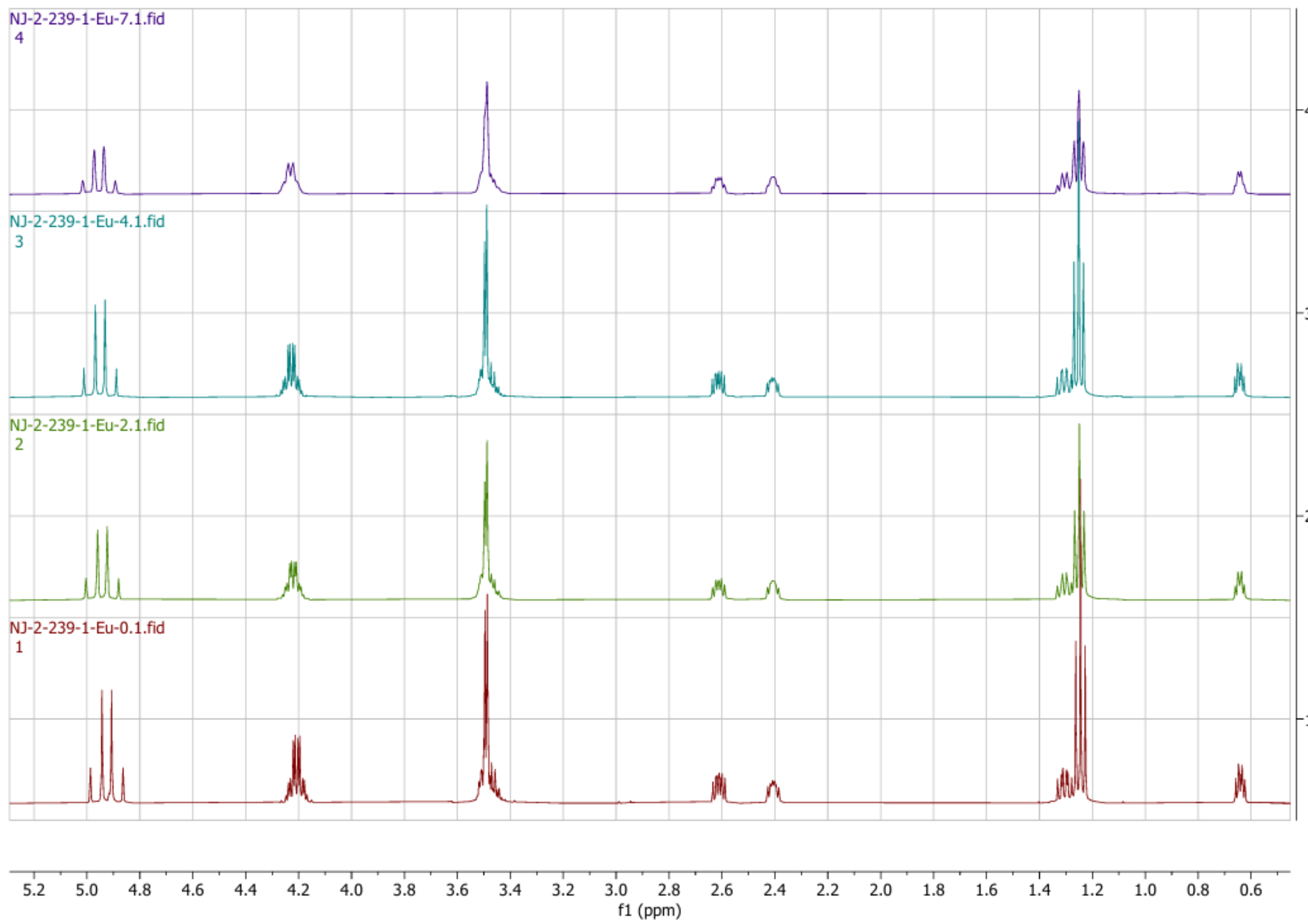


*Figure S2 : Progressive addition of Eu(hfc)3 as NMR shifting reagent to prove enantiopurity of* ***15***

After addition of Eu(hfc)$_3$ no duplicate of any peak was observed, indicating absence of the undesired enantiomer.

$^{1}H$ NMR (400 MHz, $CDCl_3$) of compound **subunit A**

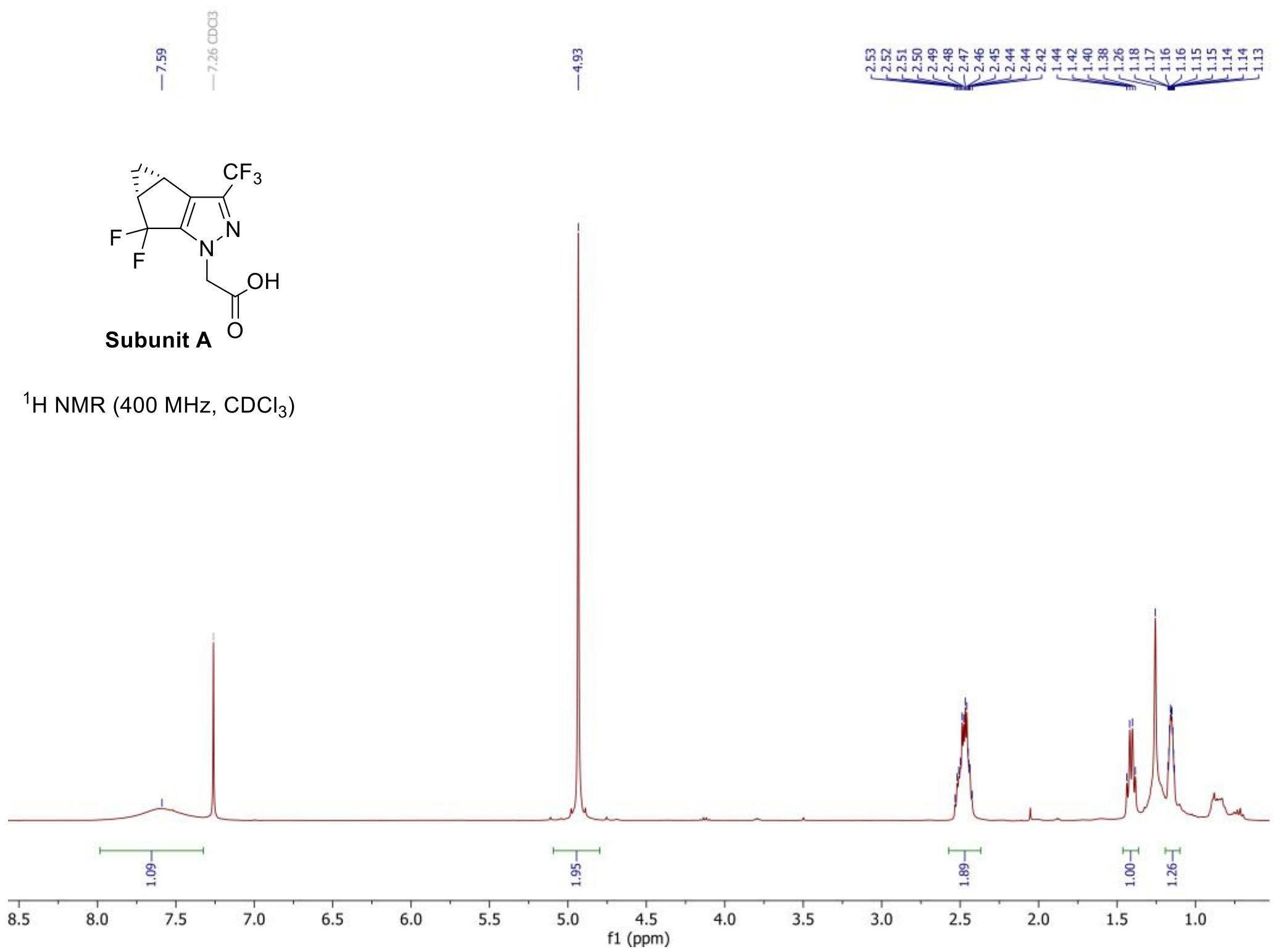


$^{13}C$ NMR (100 MHz, $CDCl_3$) of compound **subunit A**

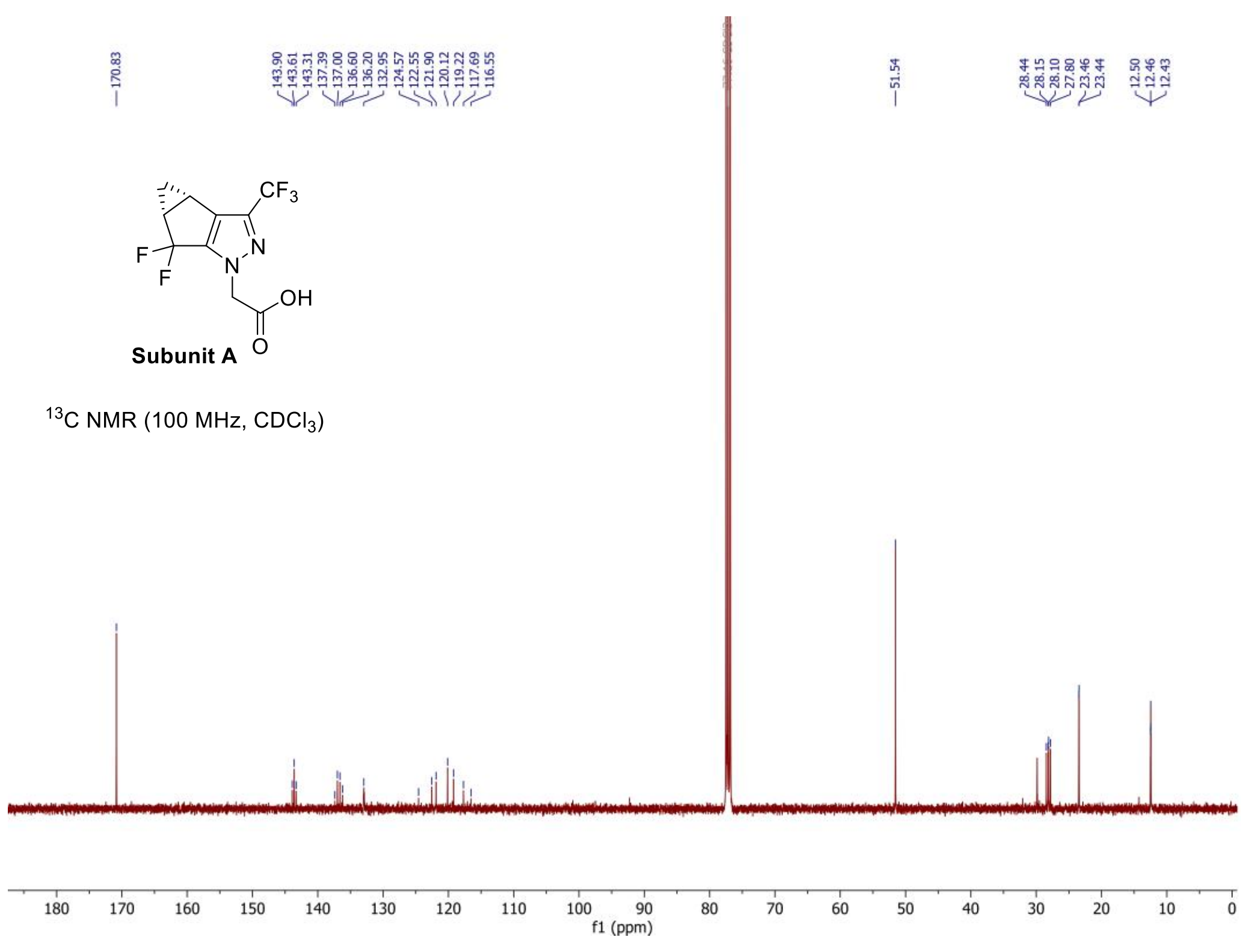

$^{1}H$ NMR (400 MHz, $CDCl_3$) of **subunit C**

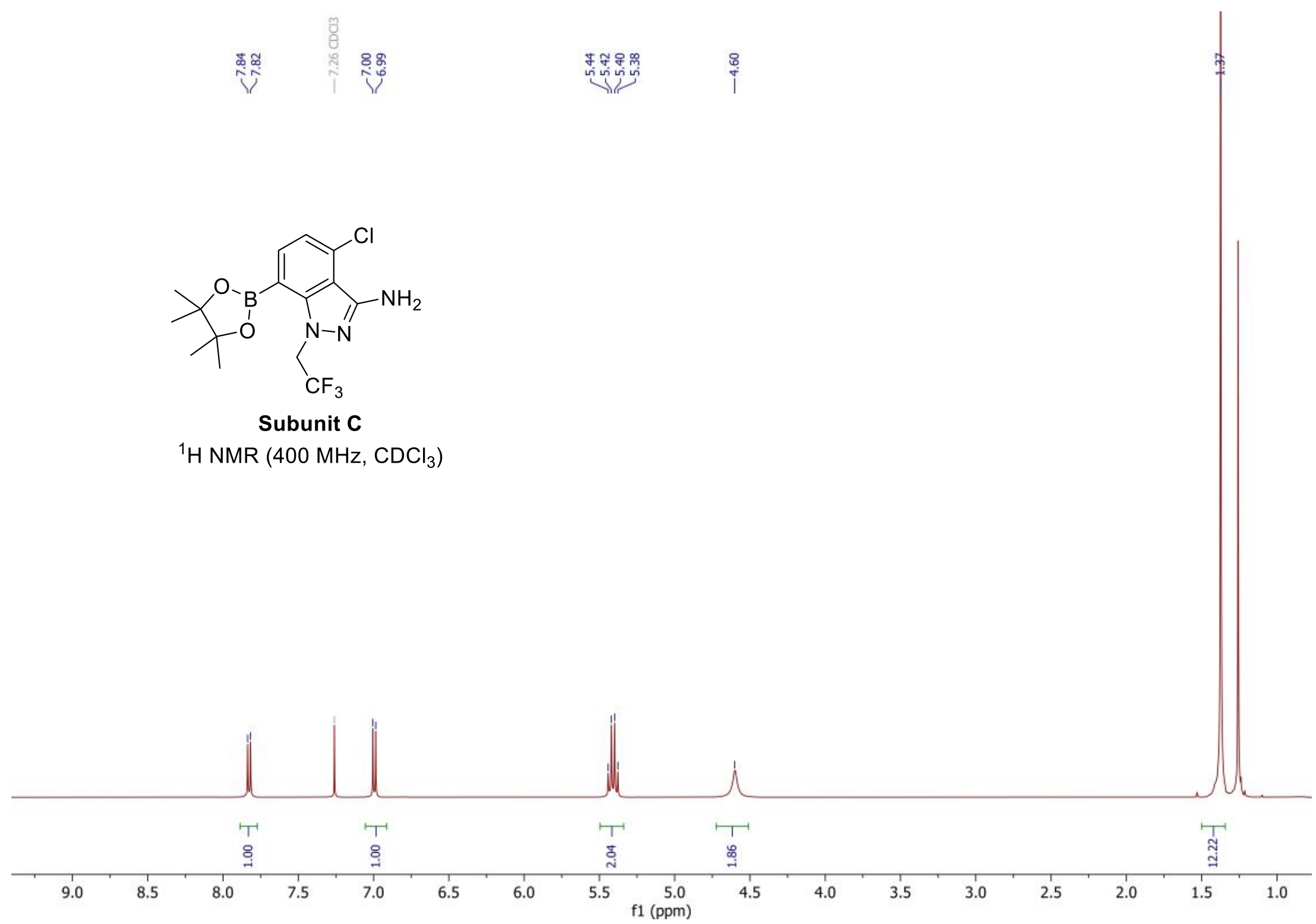


$^{13}C$ NMR (100 MHz, $CDCl_3$) of **subunit C**

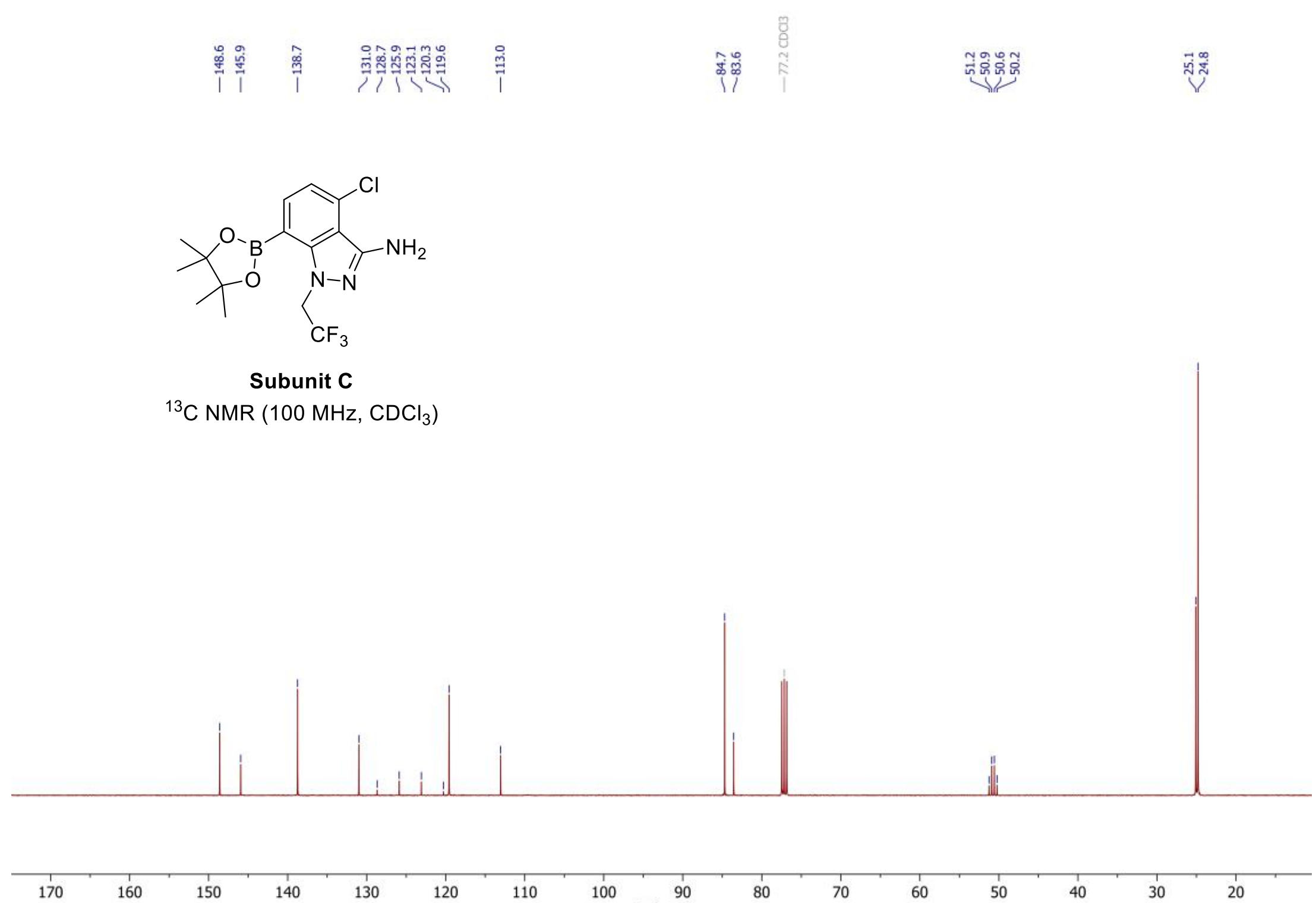

$^{1}H$ NMR (400 MHz, $CDCl_3$) of compound **22aS**

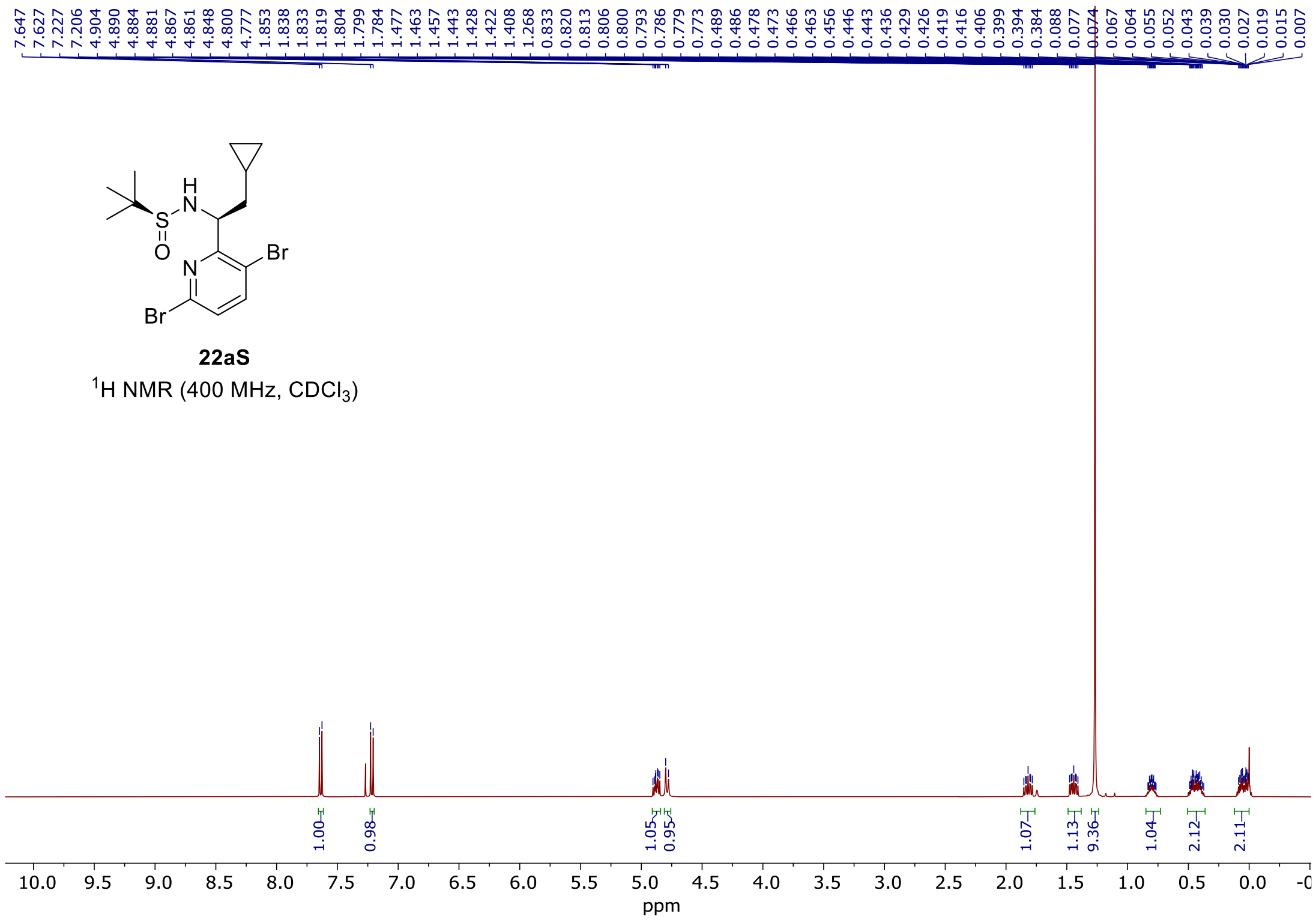


$^{13}C$ NMR (100 MHz, $CDCl_3$) of compound **22aS**

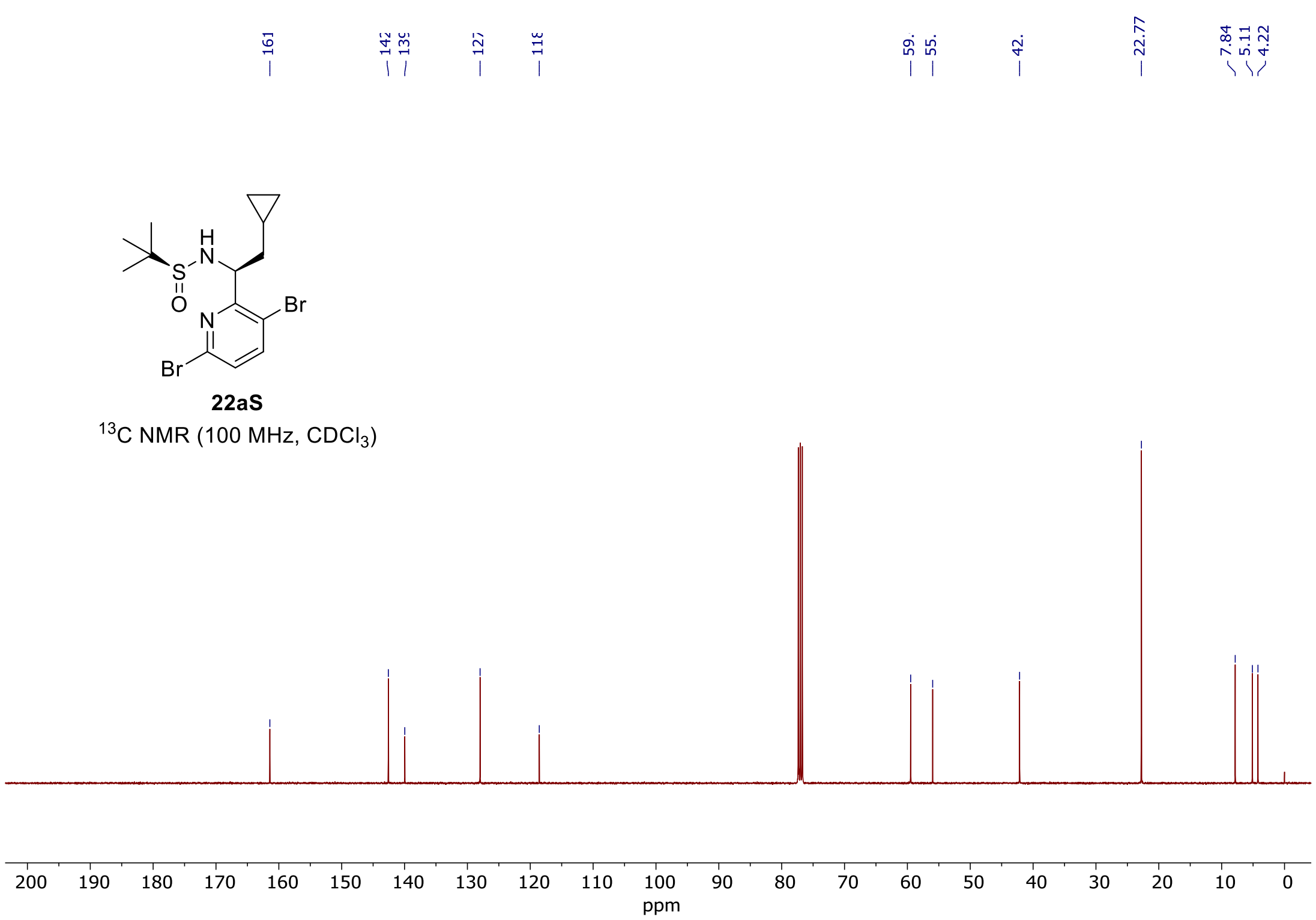

$^{1}H$ NMR (400 MHz, $CDCl_3$) of compound **22aR**

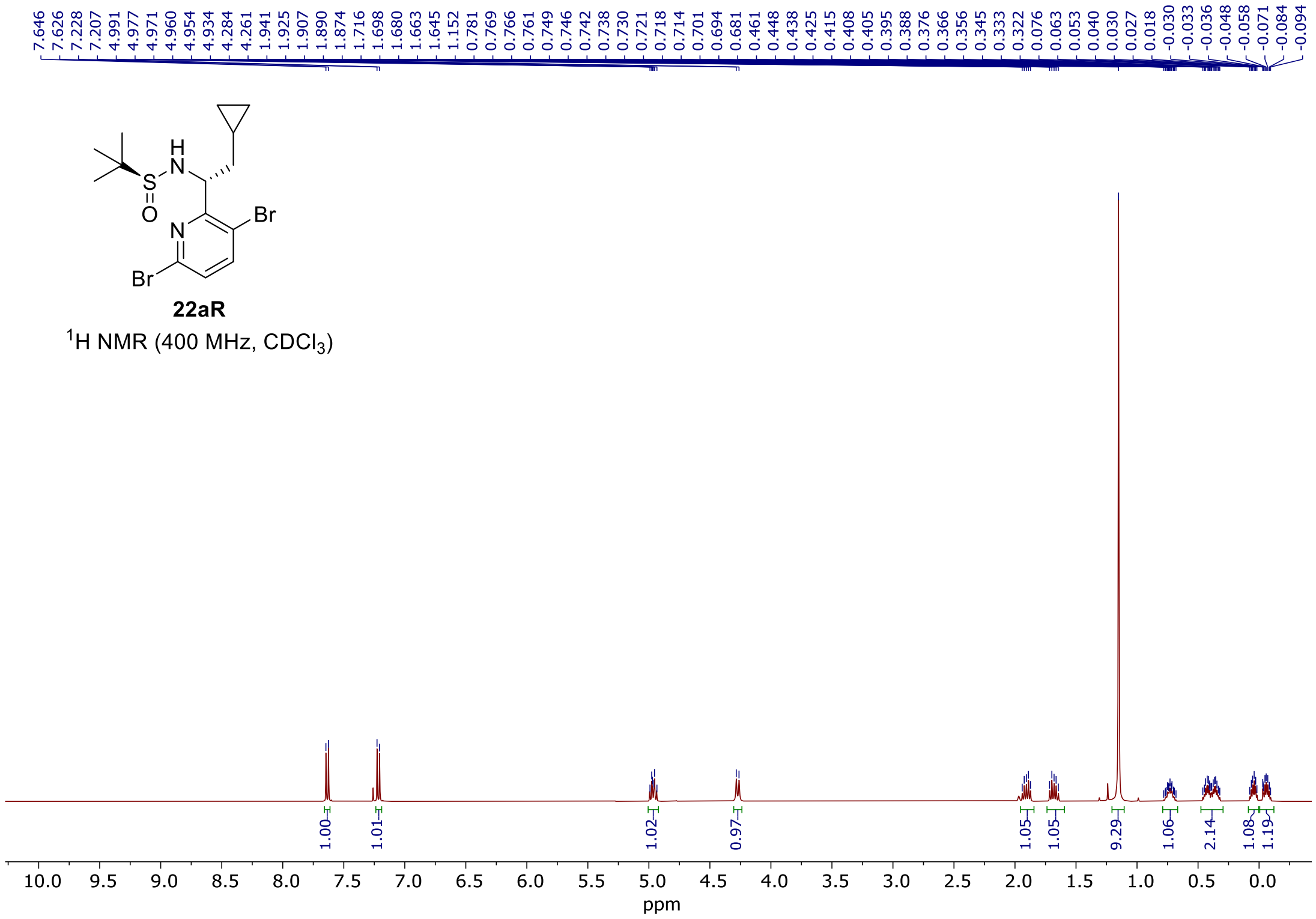


$^{13}C$ NMR (100 MHz, $CDCl_3$) of compound **22aR**

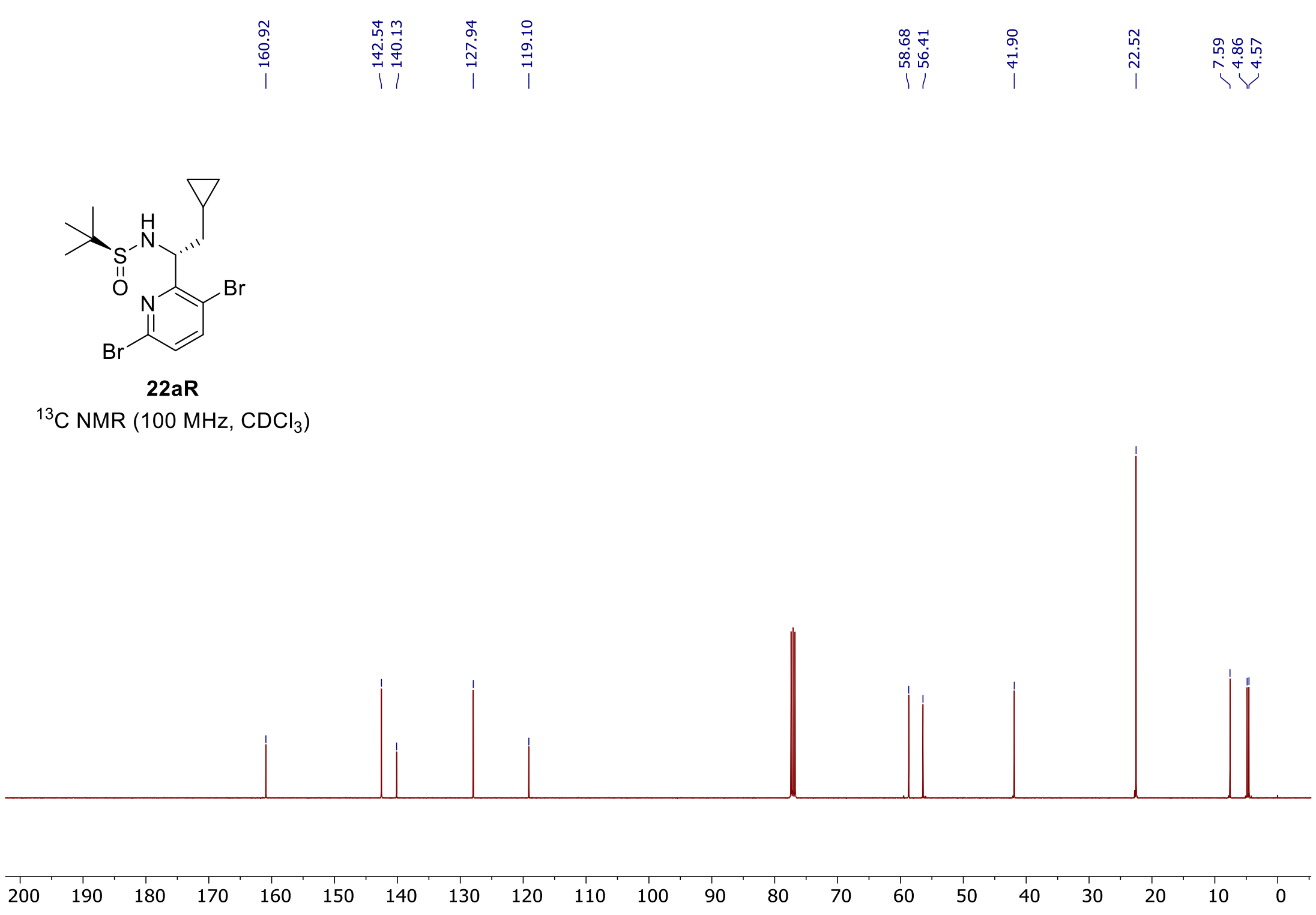

$^{1}$H NMR (400 MHz, $CDCl_3$) of the compound **22bS**

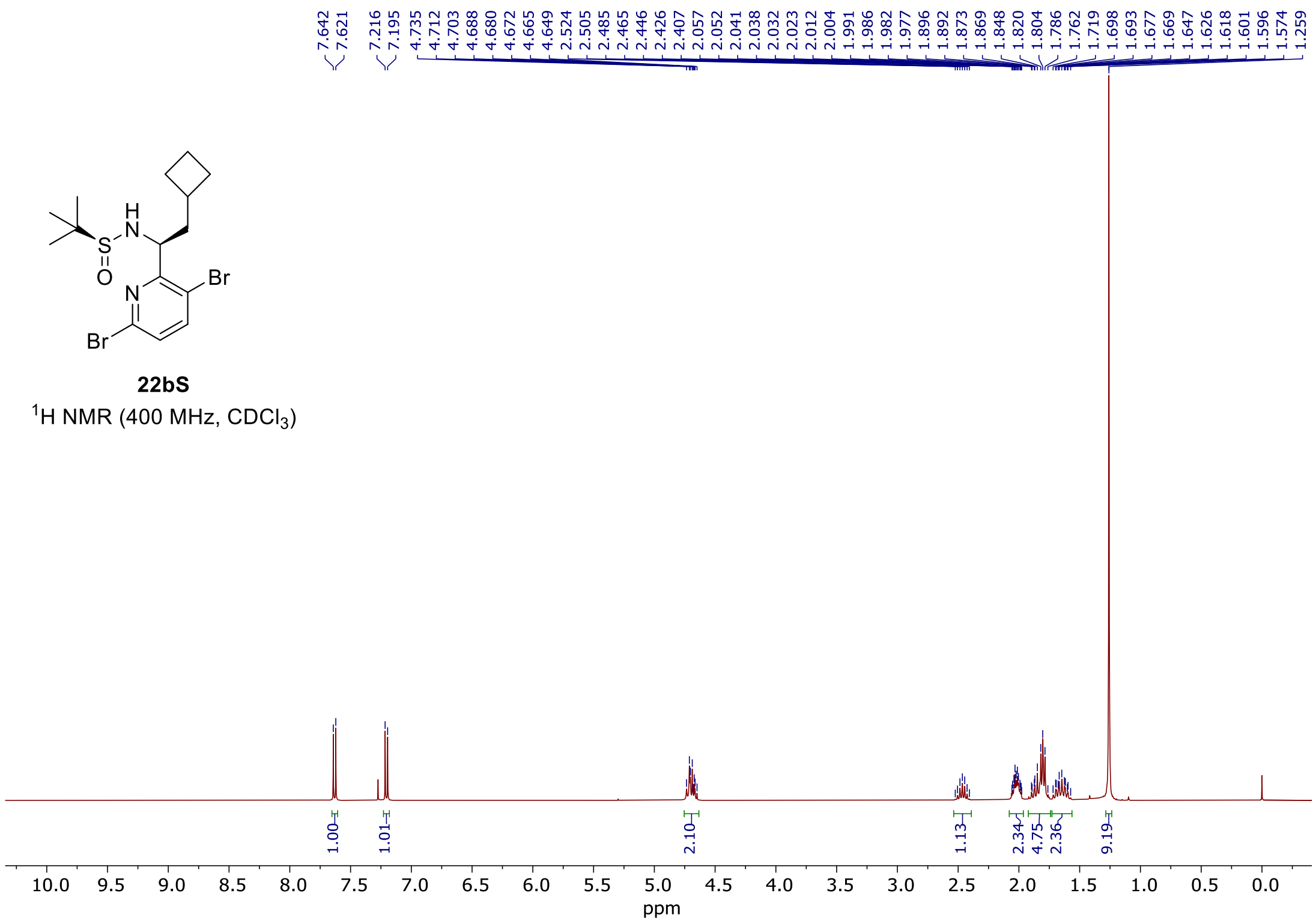


$^{13}$C NMR (100 MHz, $CDCl_3$) of compound **22bS**

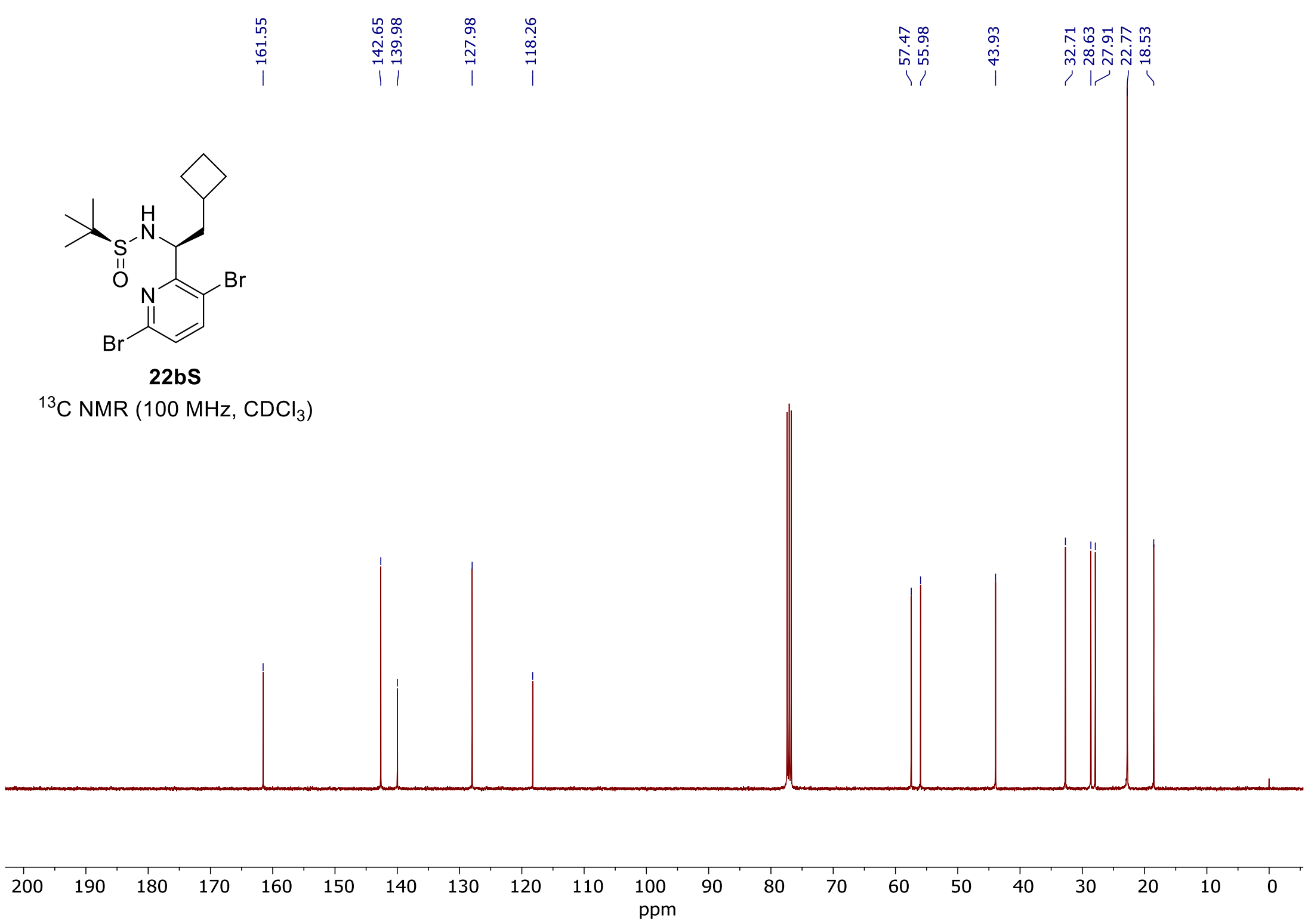

<sup></sup>

$^{1}$H NMR (400 MHz, $CDCl_3$) of the compound **22bR**

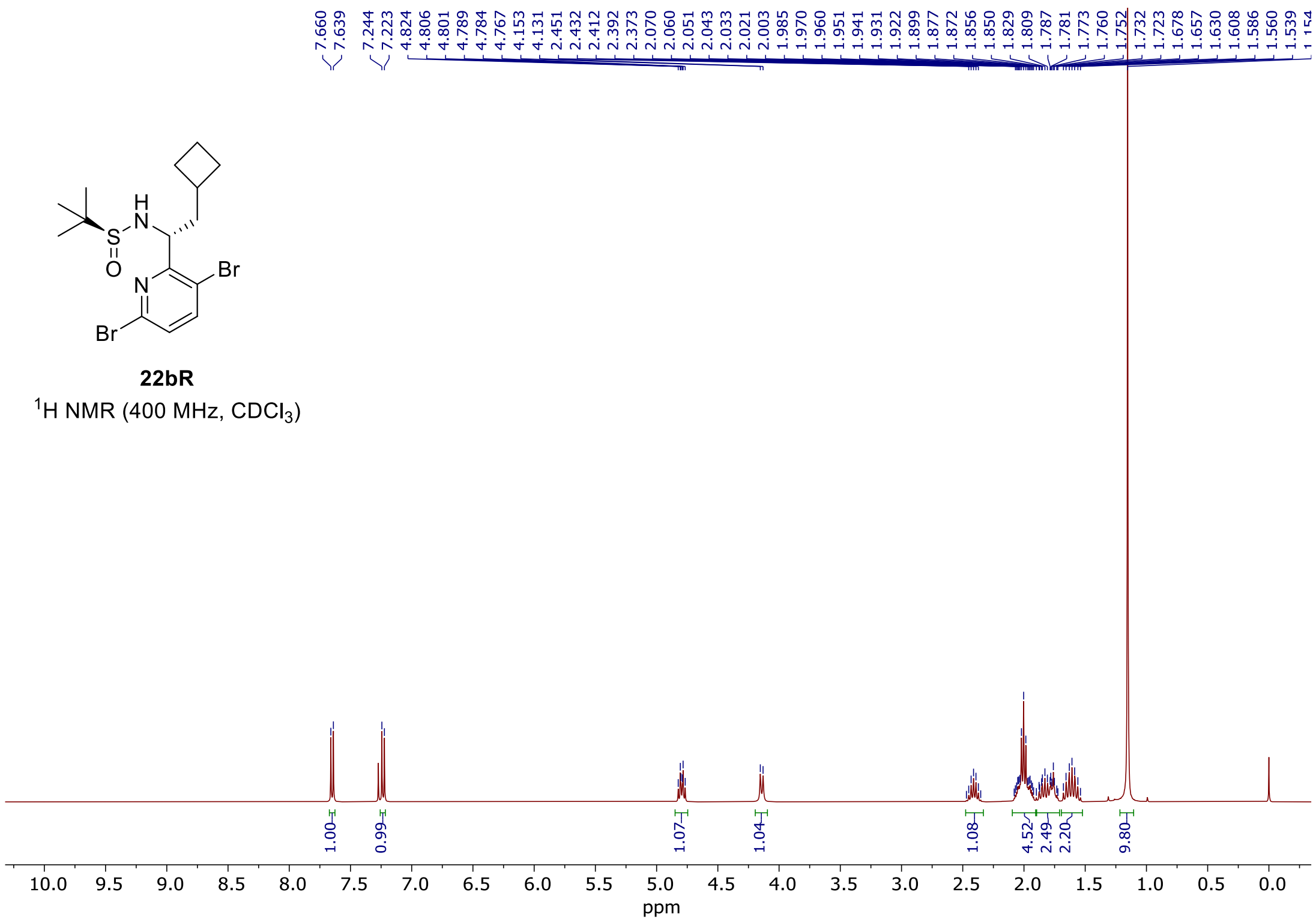


$^{13}$C NMR (100 MHz, $CDCl_3$) of compound **22bR**

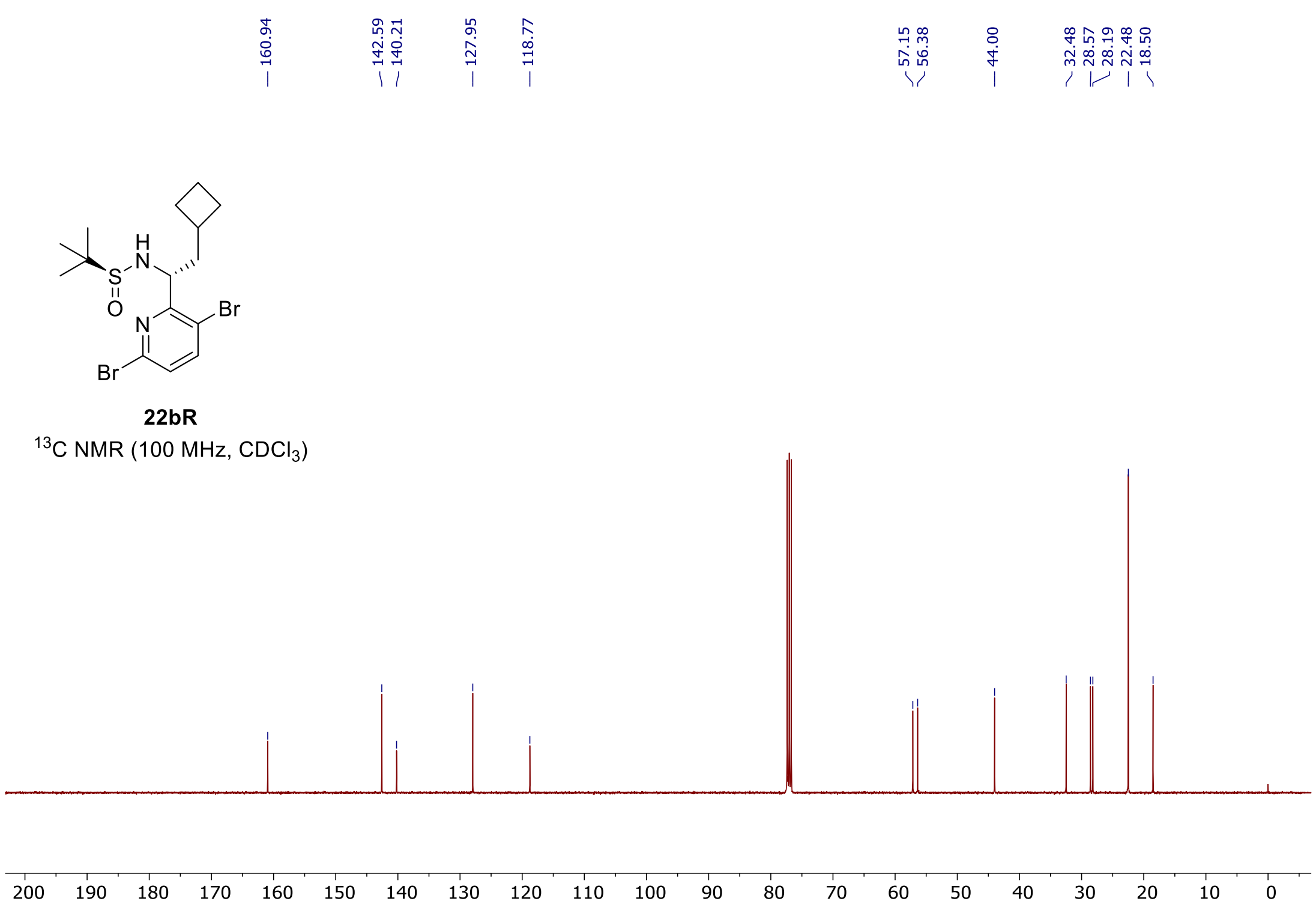

$^{1}H$ NMR (400 MHz, $CDCl_3$) of compound **22cS**

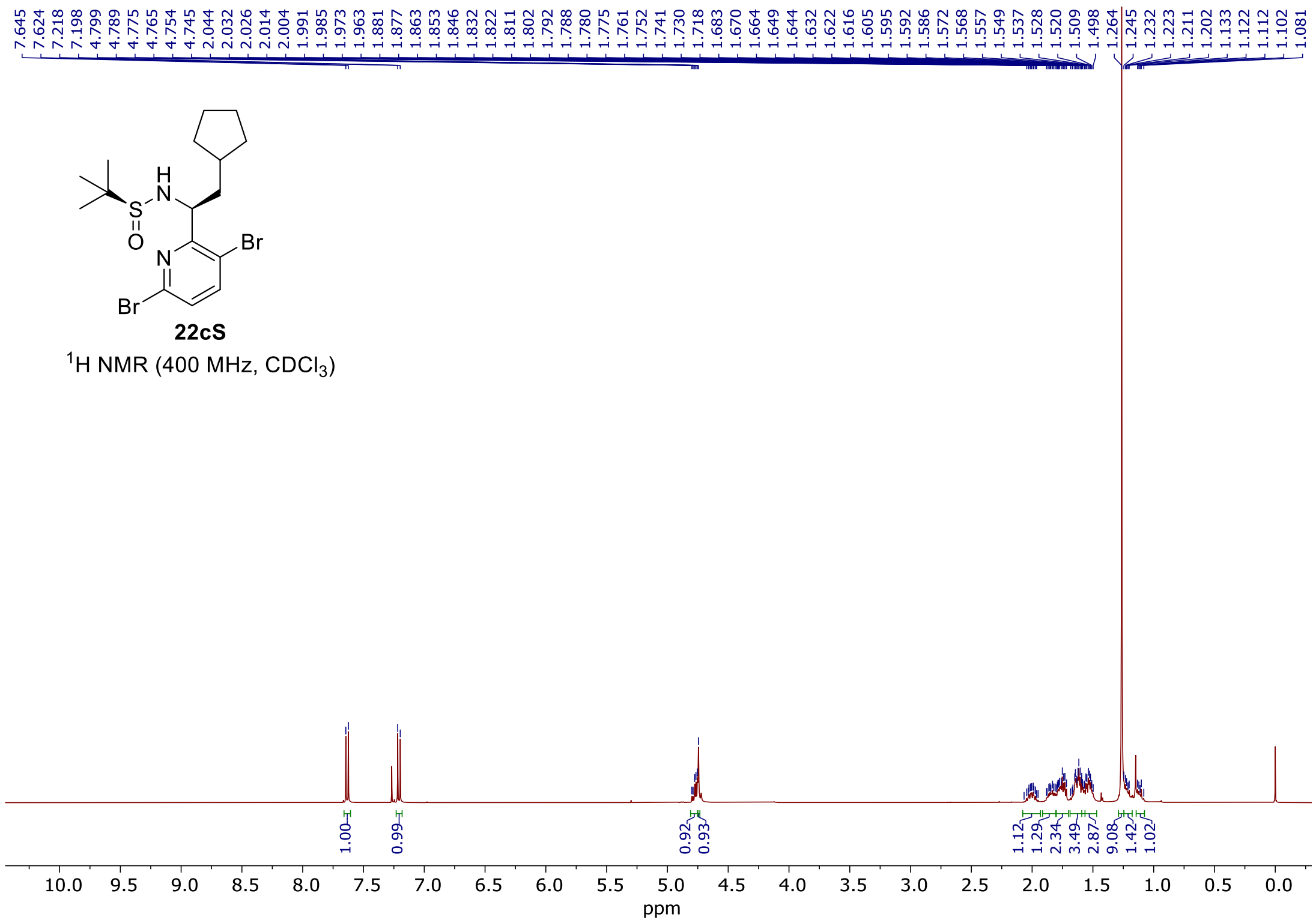


$^{13}C$ NMR (100 MHz, $CDCl_3$) of compound **22cS**

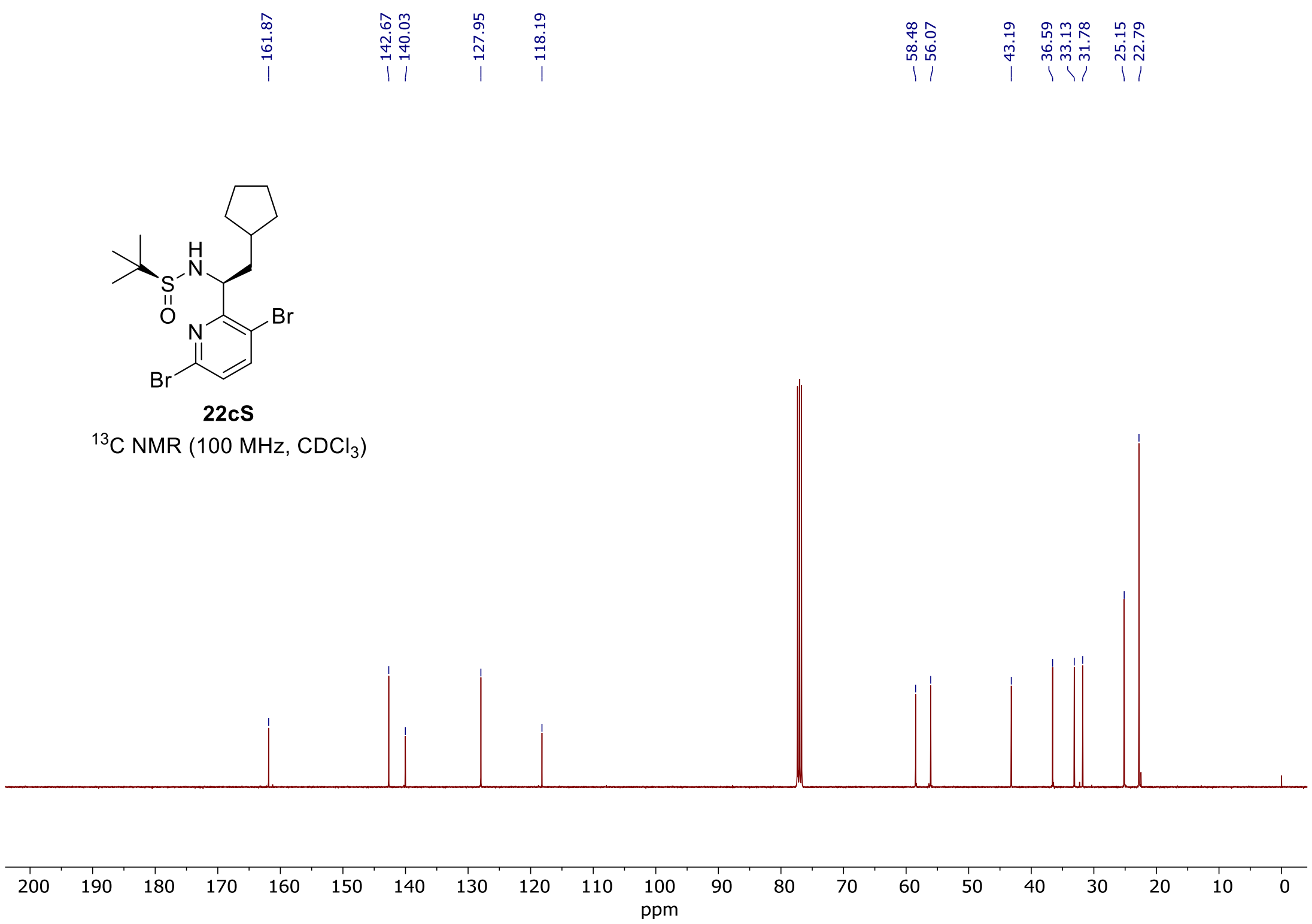

$^{1}$H NMR (400 MHz, $CDCl_3$) of compound **22cR**

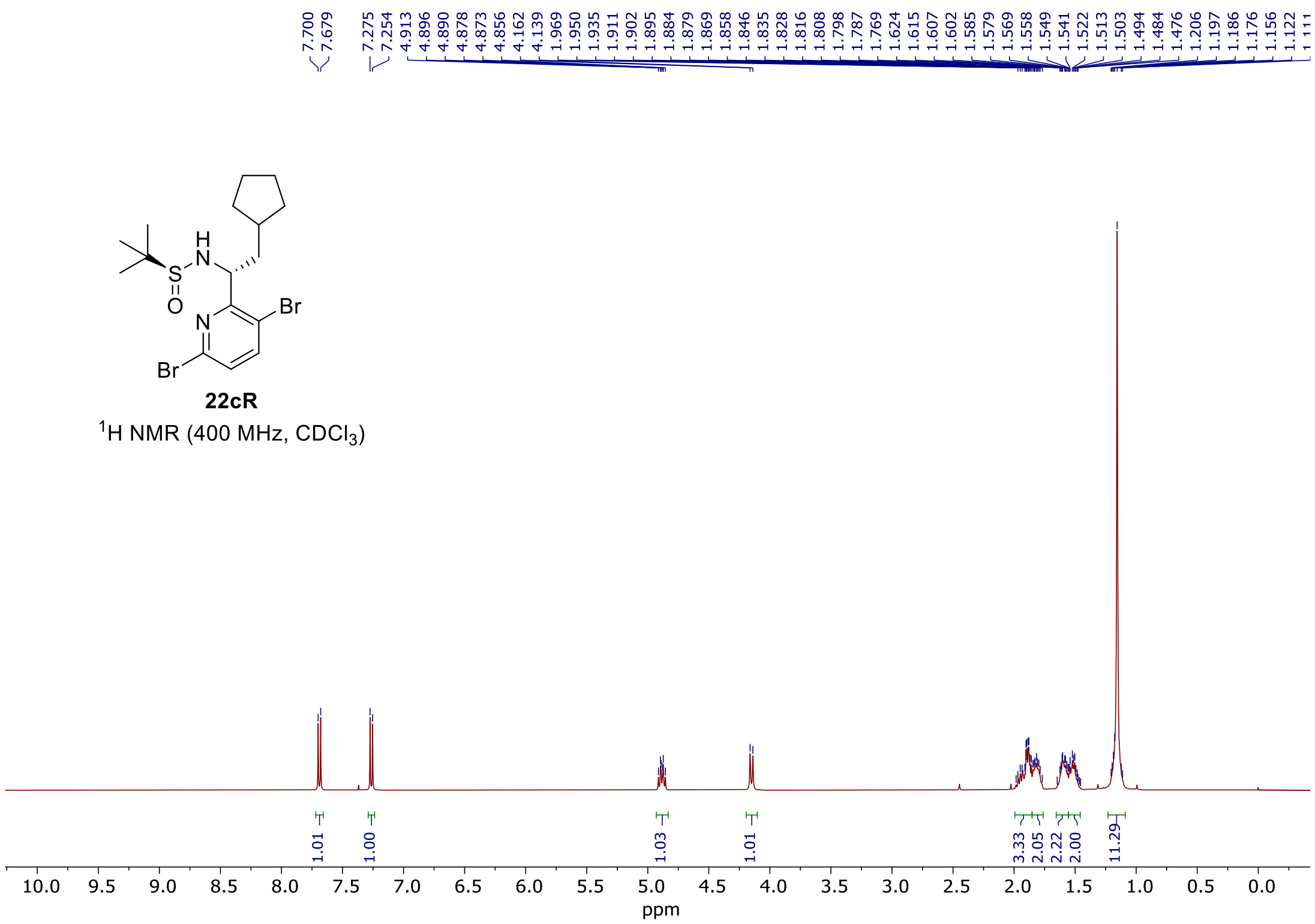


$^{13}$C NMR (100 MHz, $CDCl_3$) of compound **22cR**

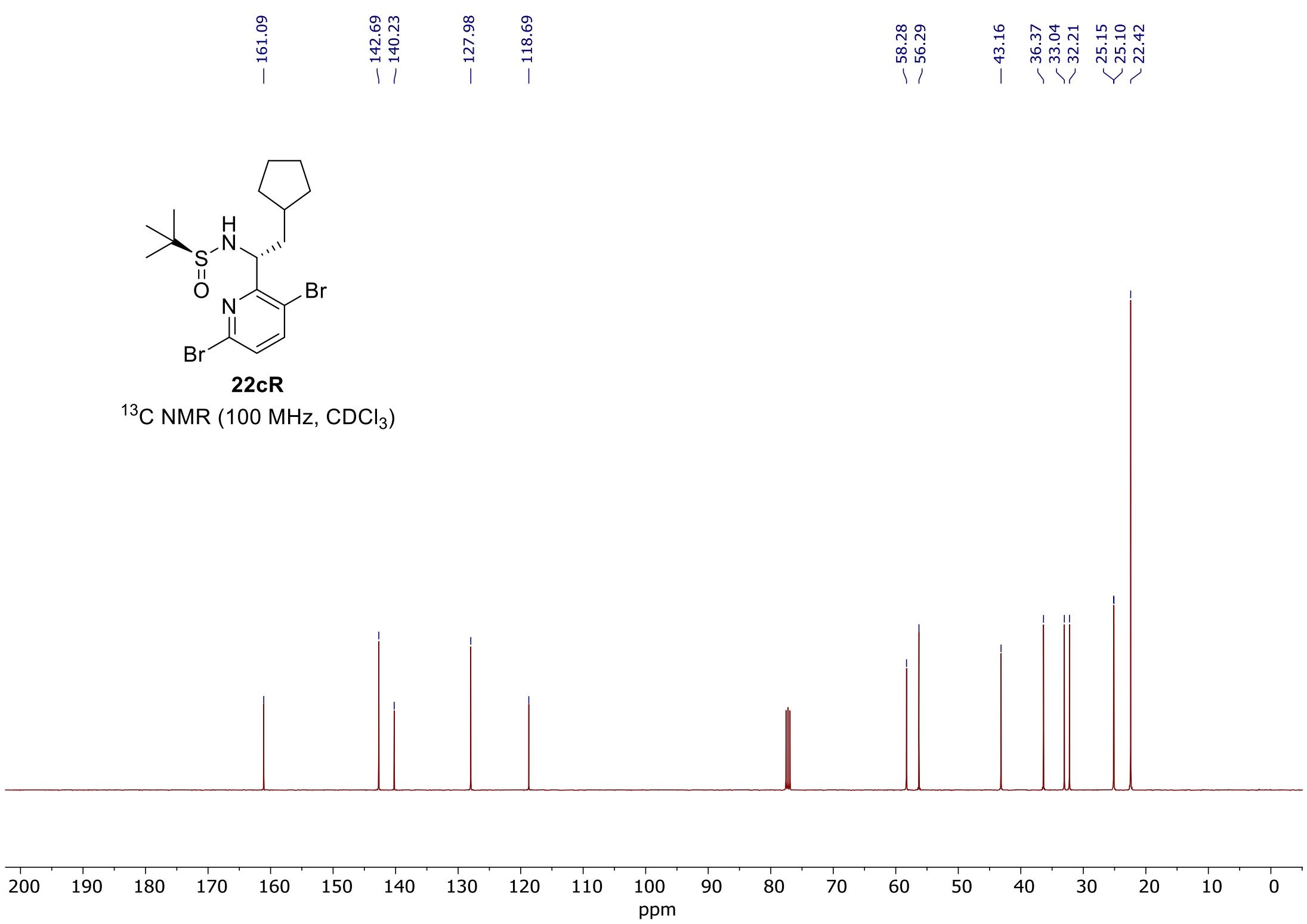

## $^{1}$H NMR (400 MHz, $CDCl_3$) of compound **22dS**

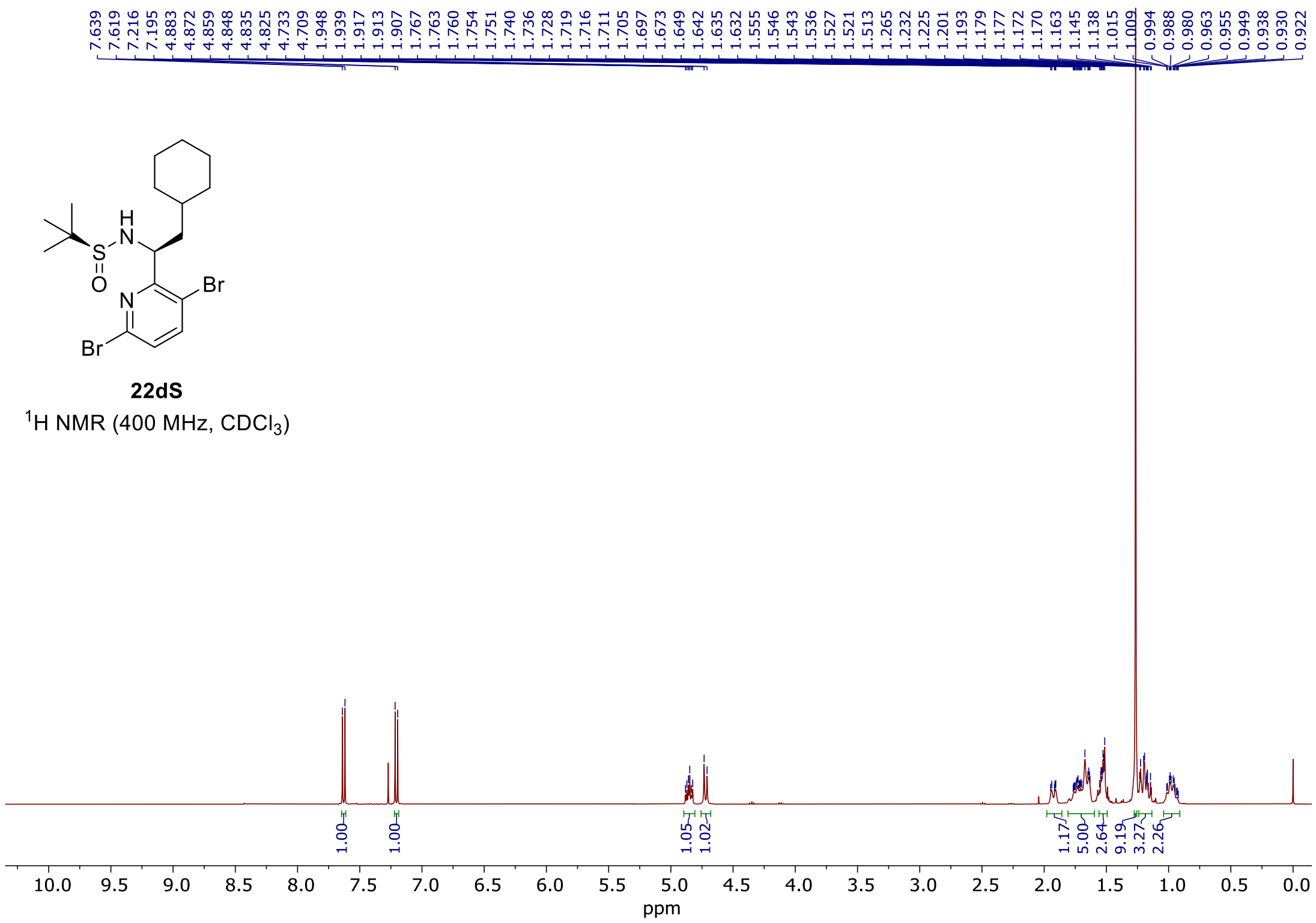


## $^{13}$C NMR (100 MHz, $CDCl_3$) of compound **22dS**

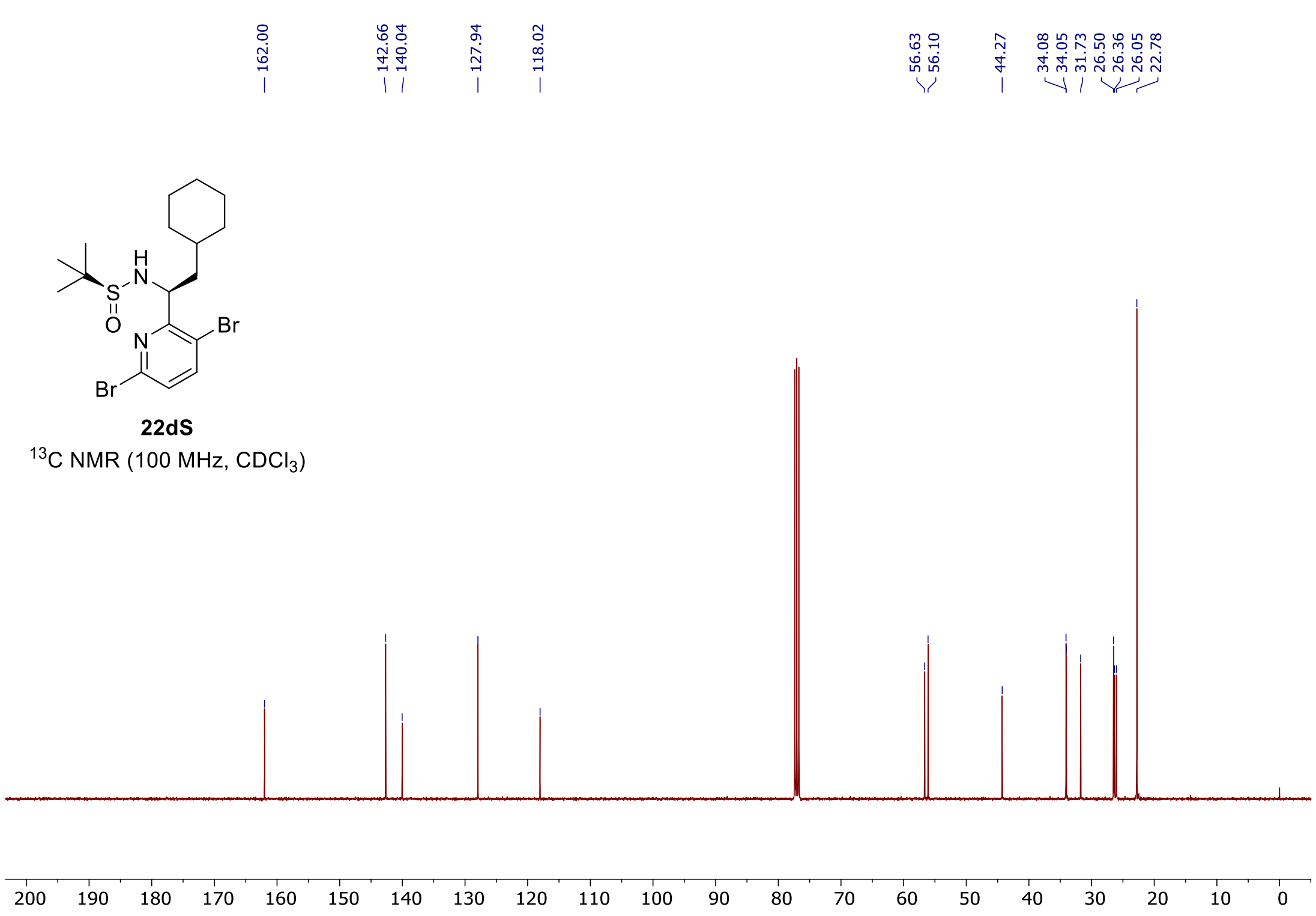

$^{1}H$ NMR (400 MHz, $CDCl_3$) of compound **22dR**

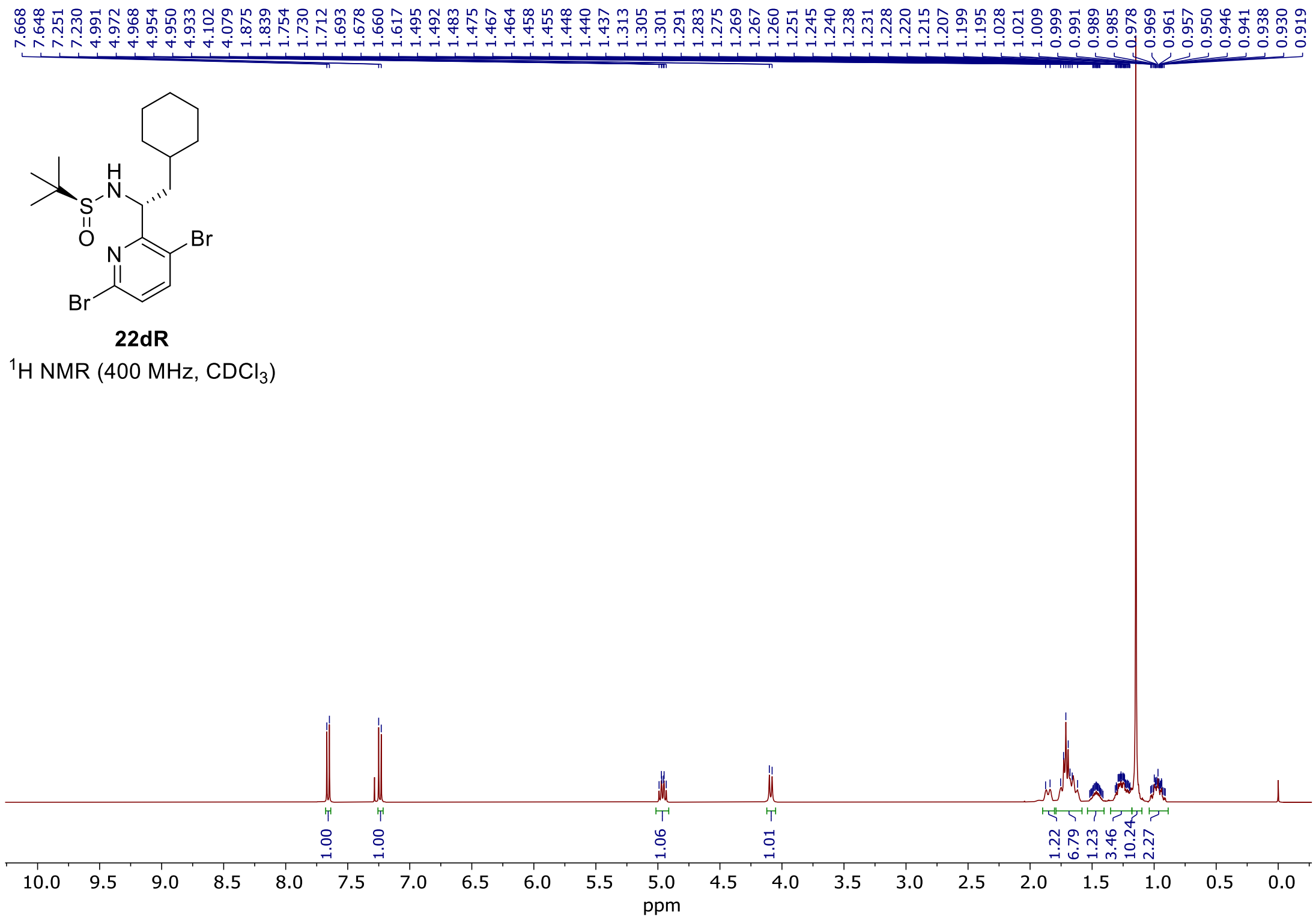


$^{13}C$ NMR (100 MHz, $CDCl_3$) of compound **22dR**

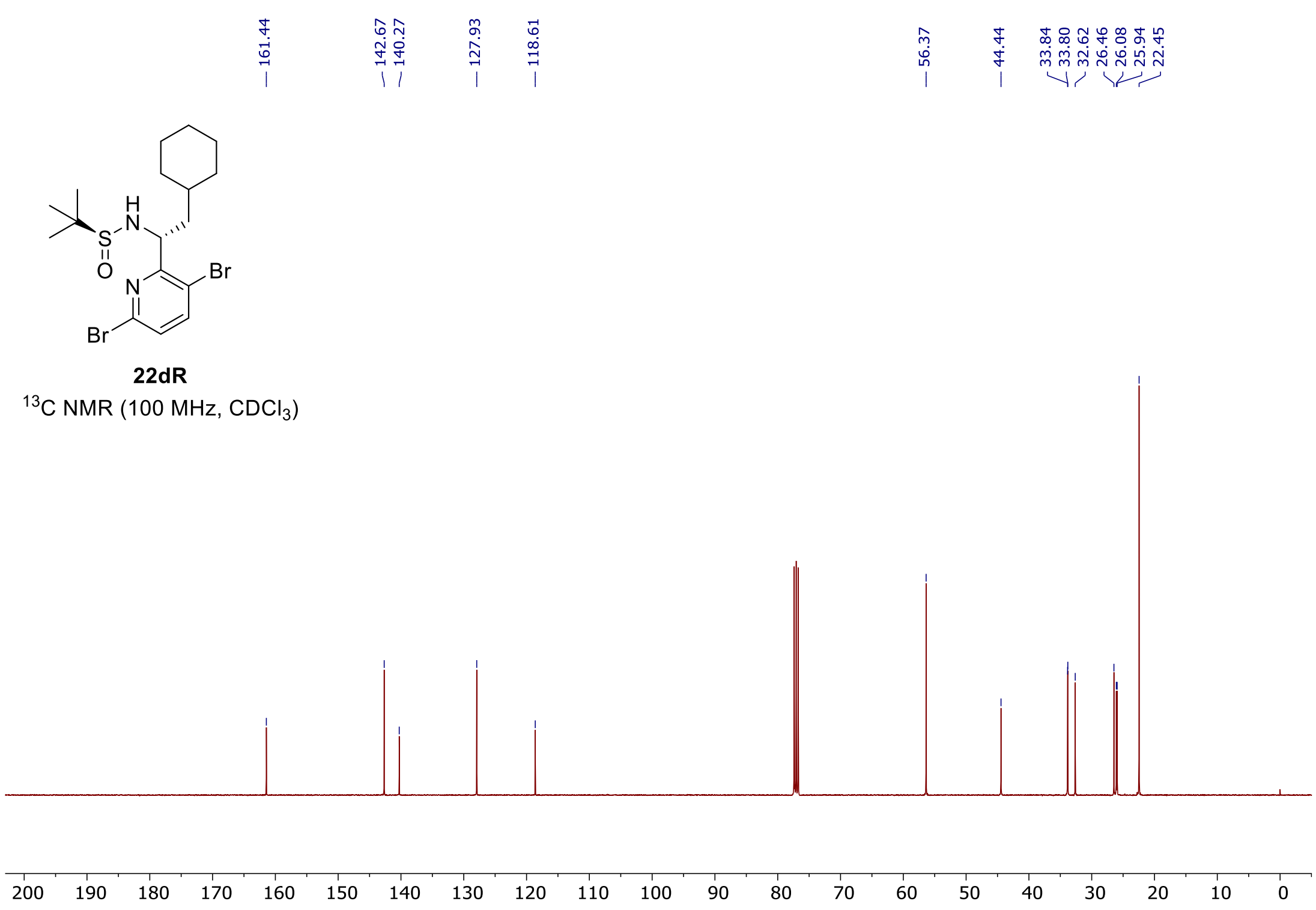

$^{1}H$ NMR (400 MHz, $CDCl_3$) of the compound **26b**

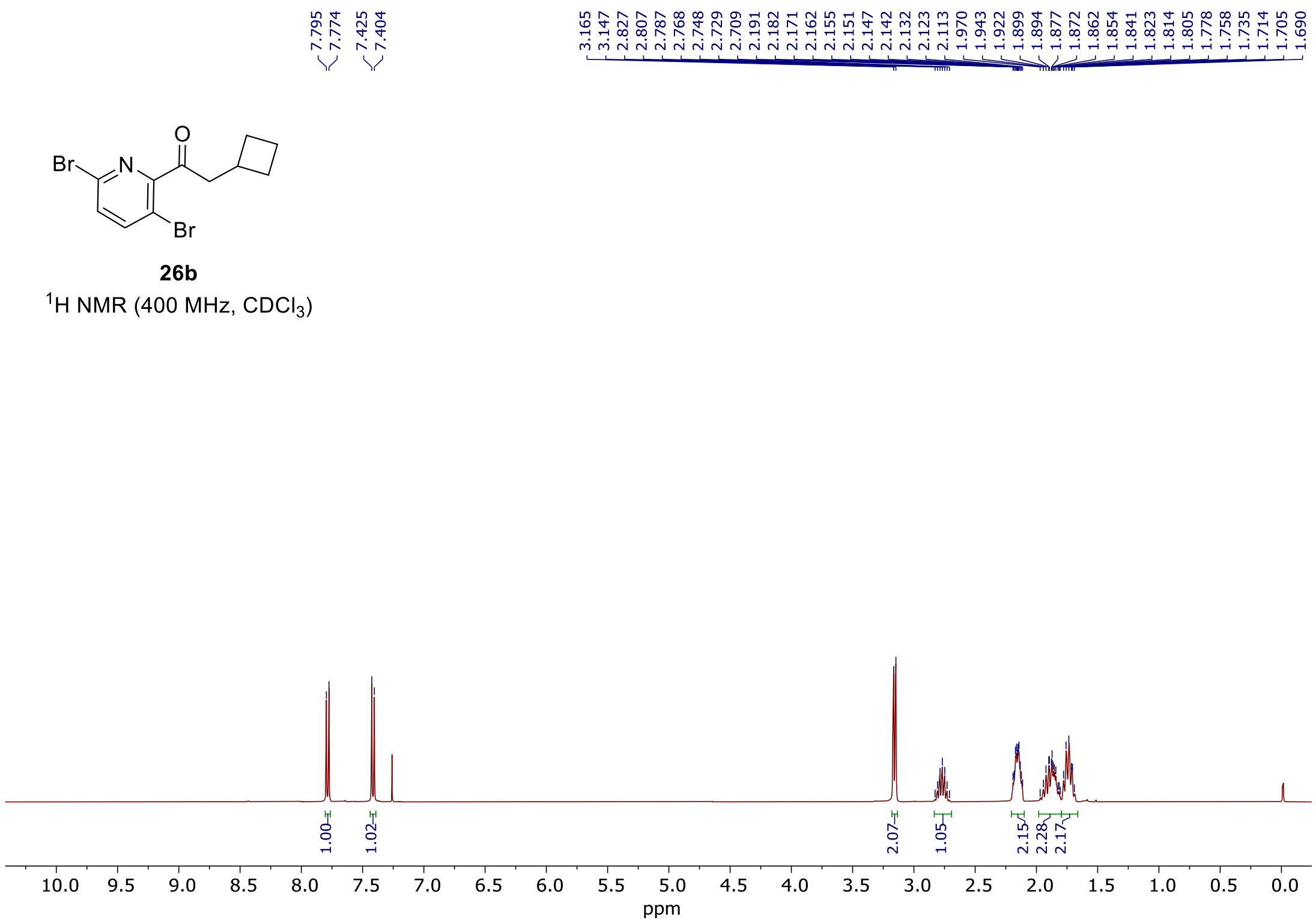


$^{13}C$ NMR (100 MHz, $CDCl_3$) of compound **26b**

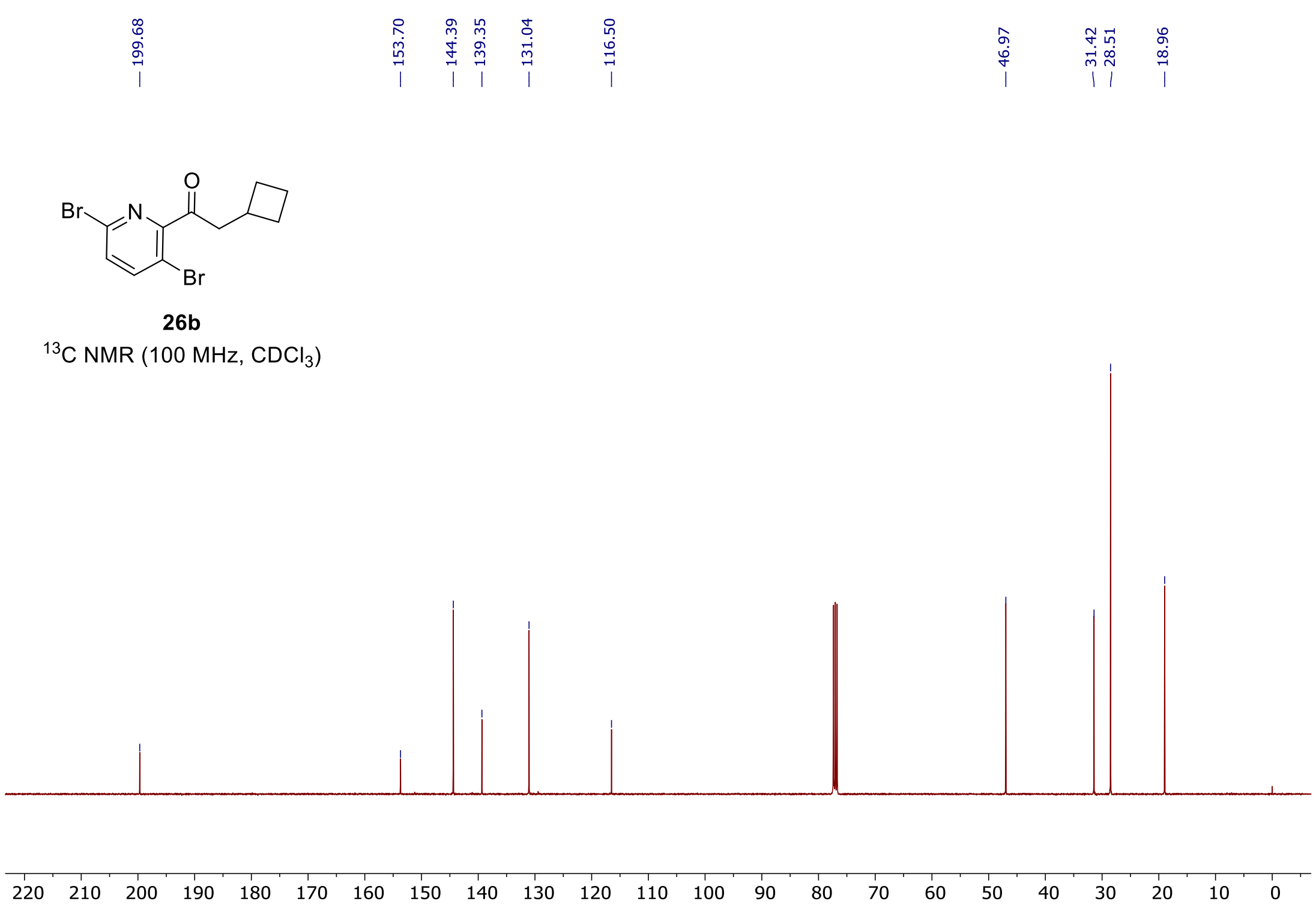

$^{1}$H NMR (400 MHz, $CDCl_3$) of compound **26d**

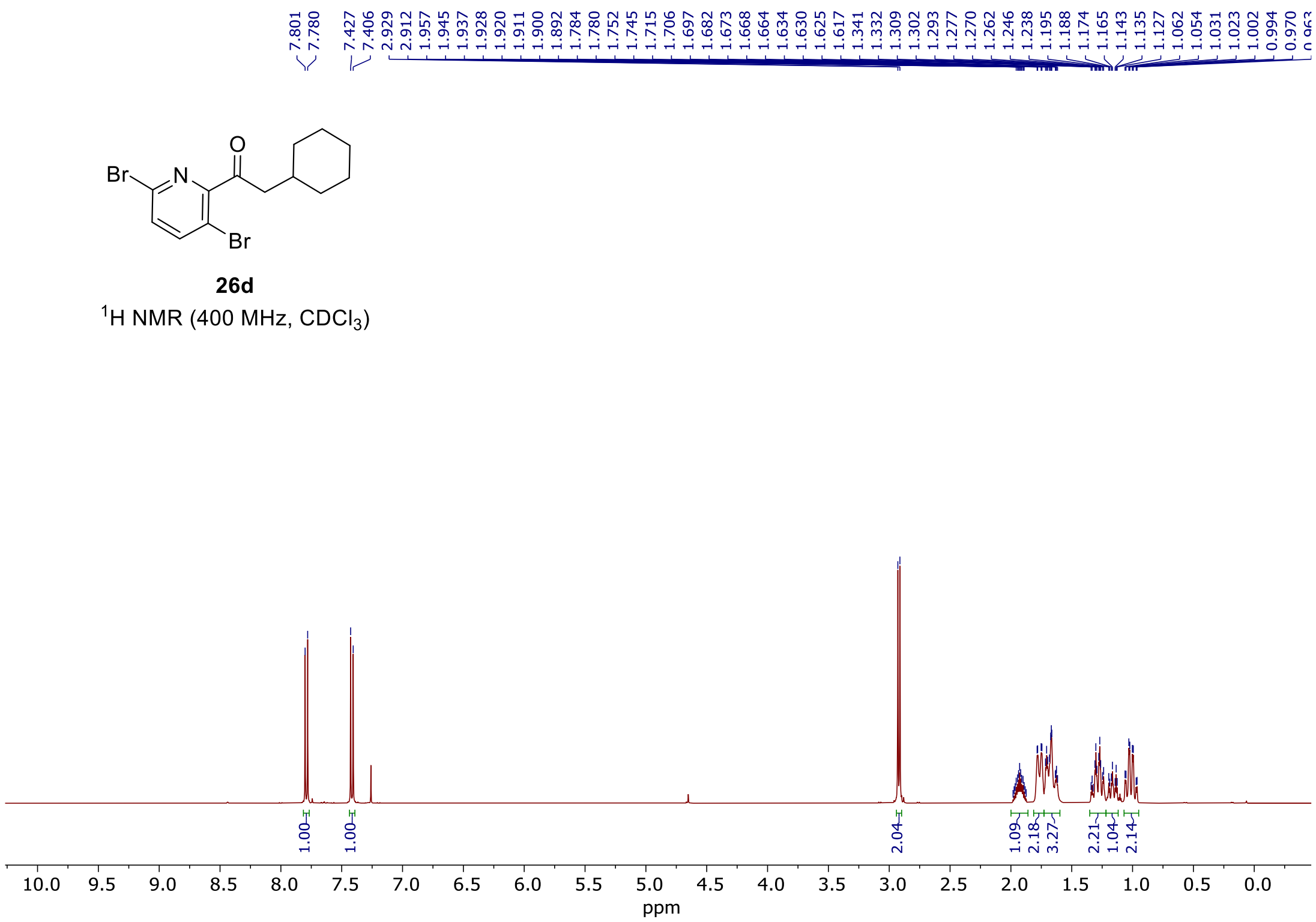


$^{13}$C NMR (100 MHz, $CDCl_3$) of compound **26d**

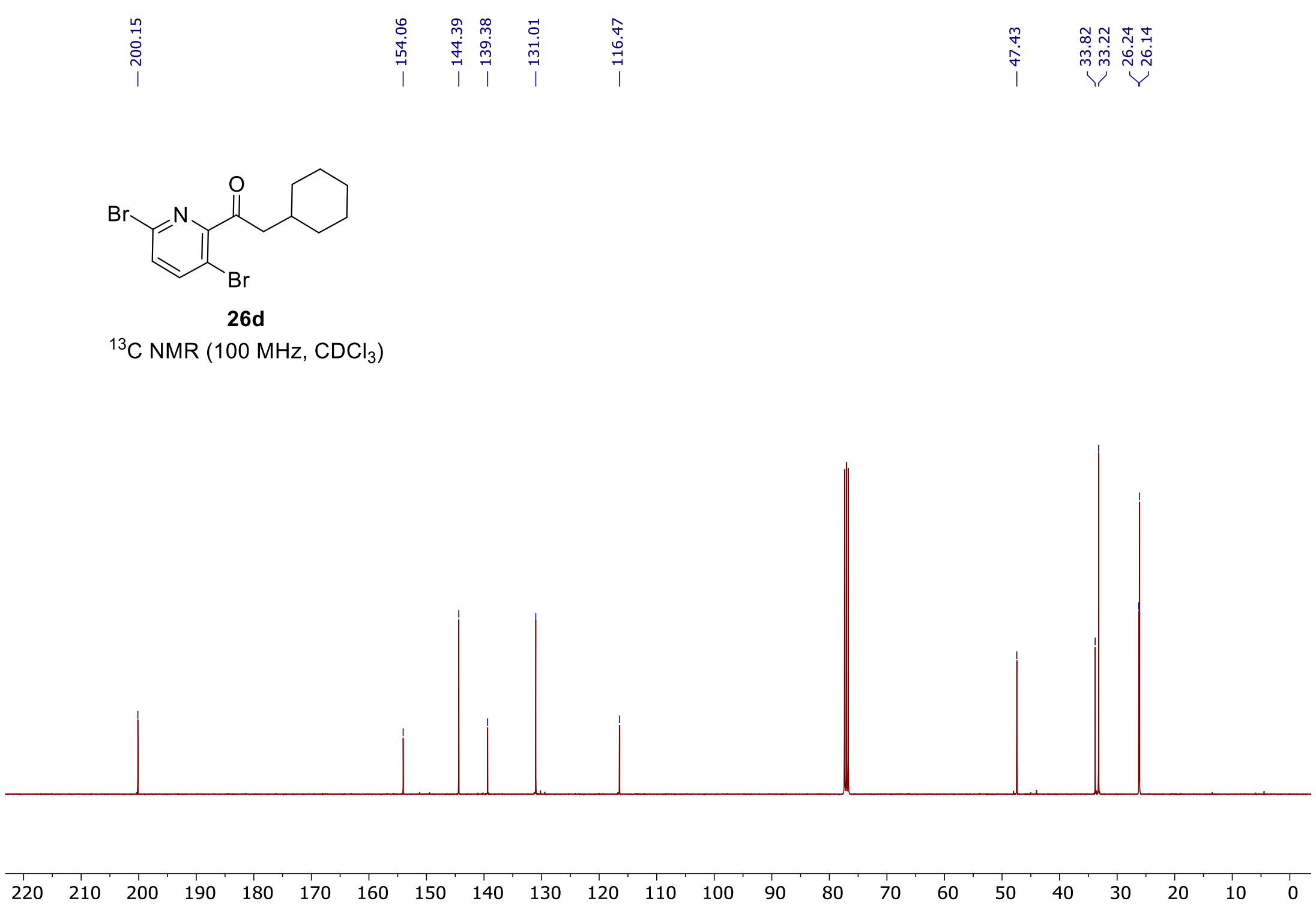

$^{1}$H NMR (400 MHz, $CDCl_3$) of compound **27a**

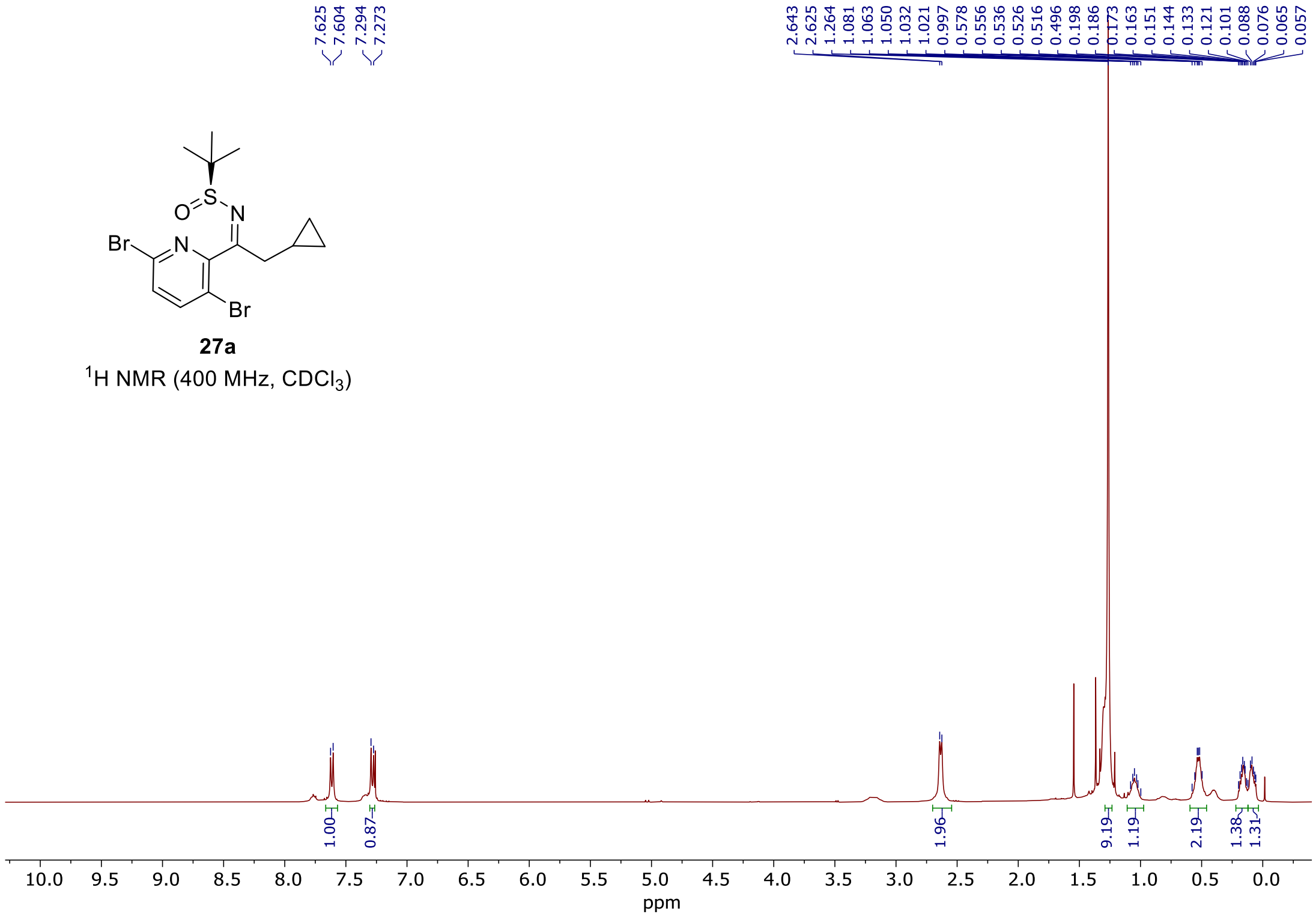


$^{13}$C NMR (100 MHz, $CDCl_3$) of compound **27a**

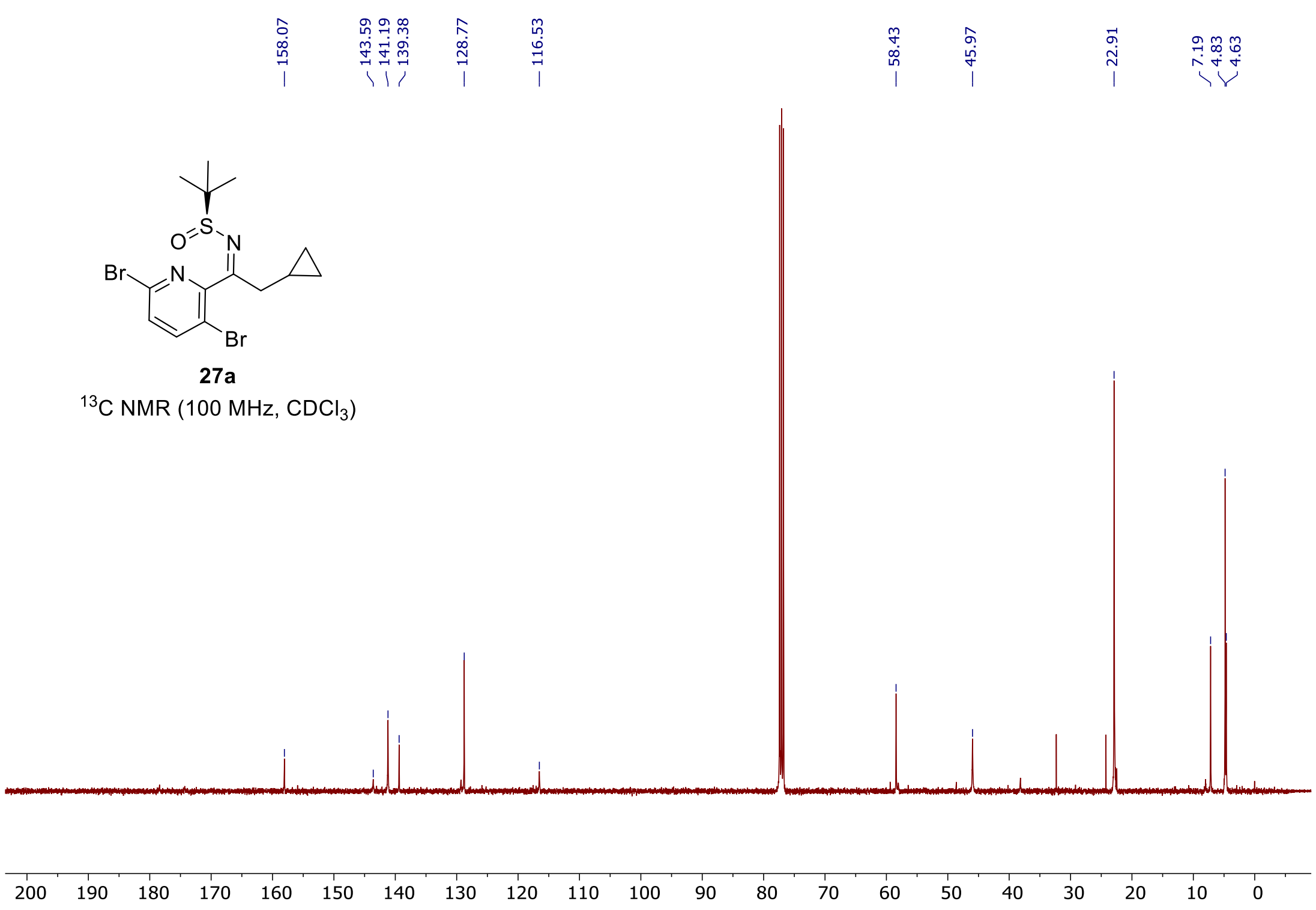

$^{1}$H NMR (400 MHz, $CDCl_3$) of the compound **27b**

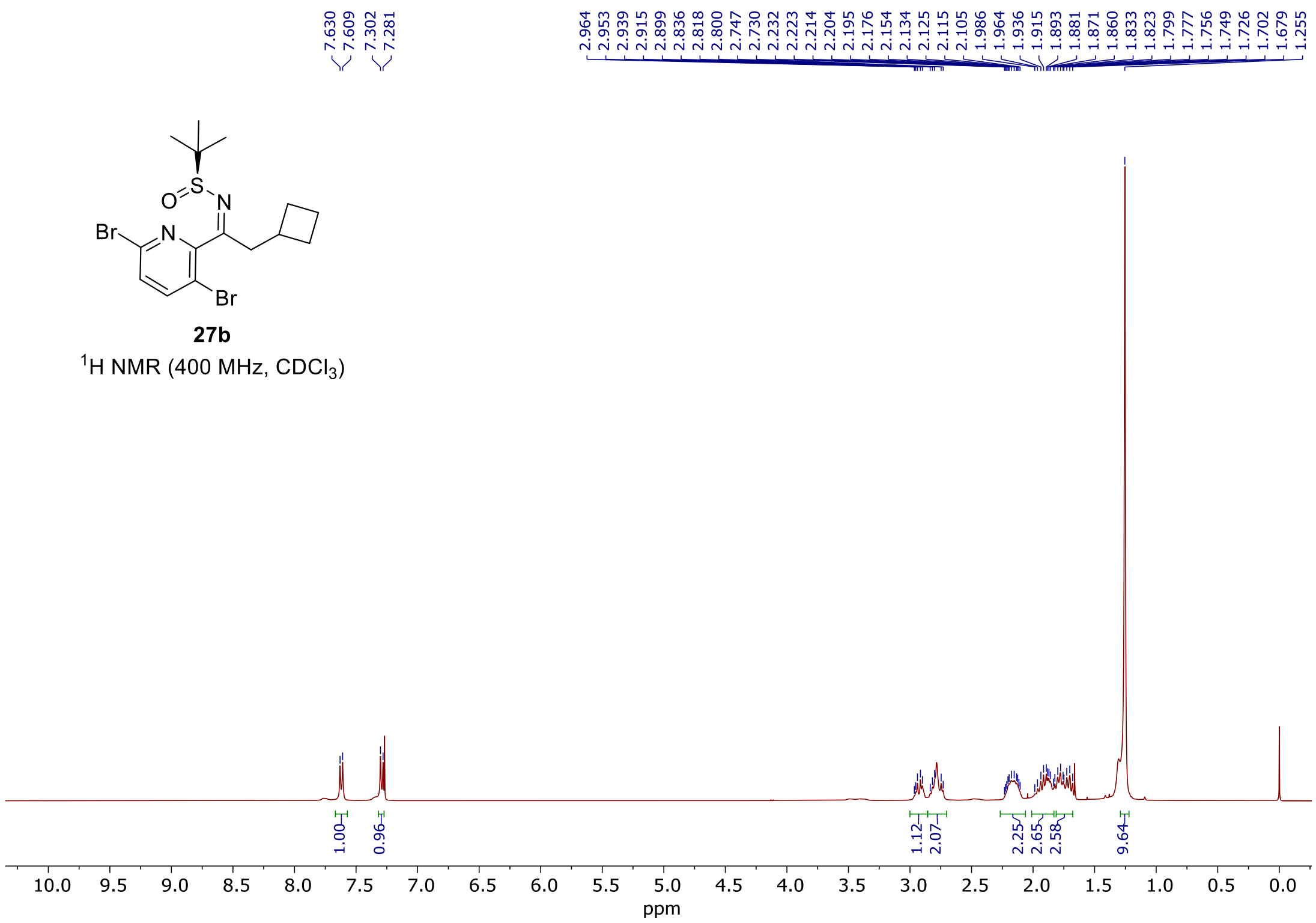


$^{13}$C NMR (100 MHz, $CDCl_3$) of compound **27b**

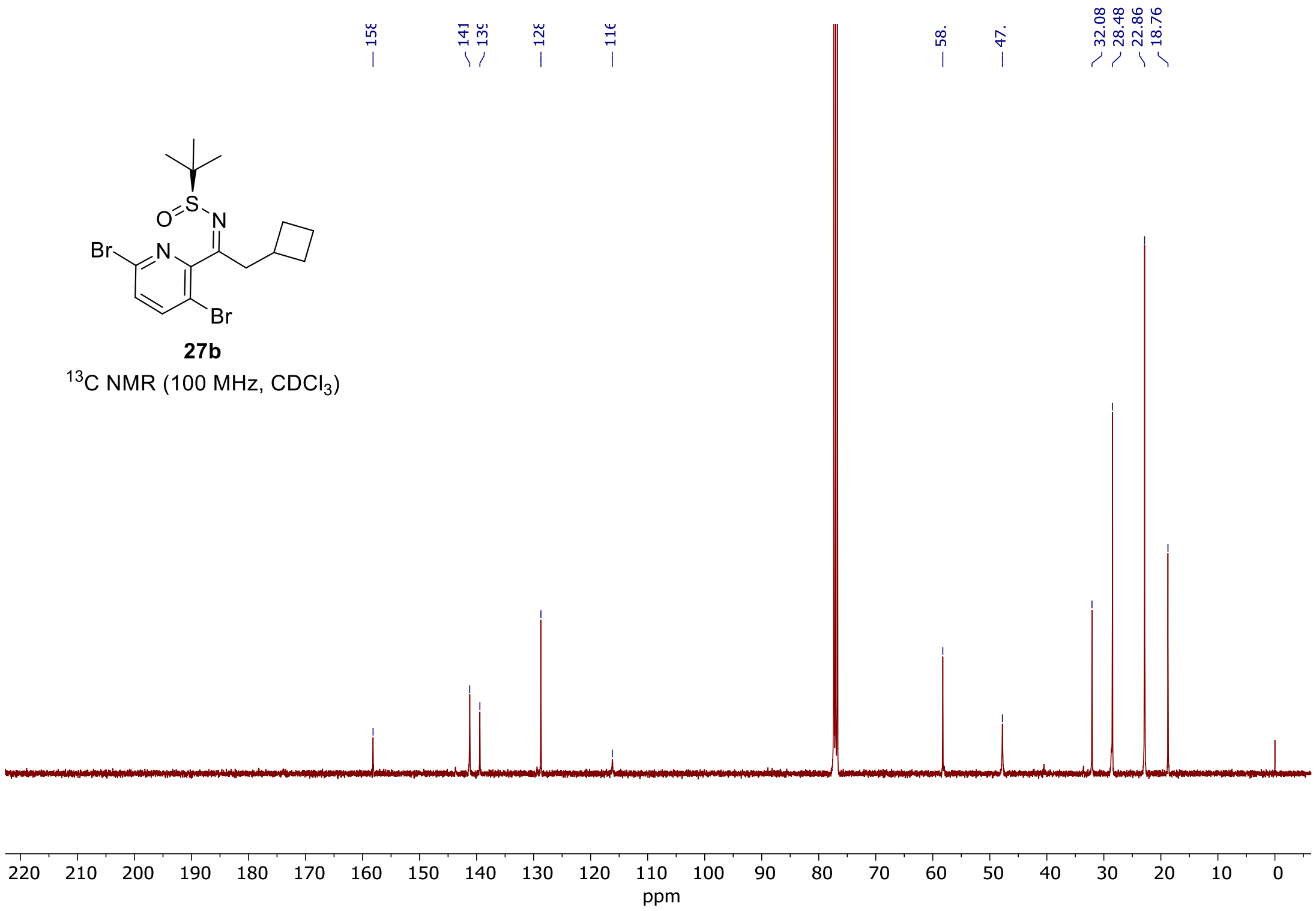

$^1$H NMR (400 MHz, $CDCl_3$) of compound **29aS**

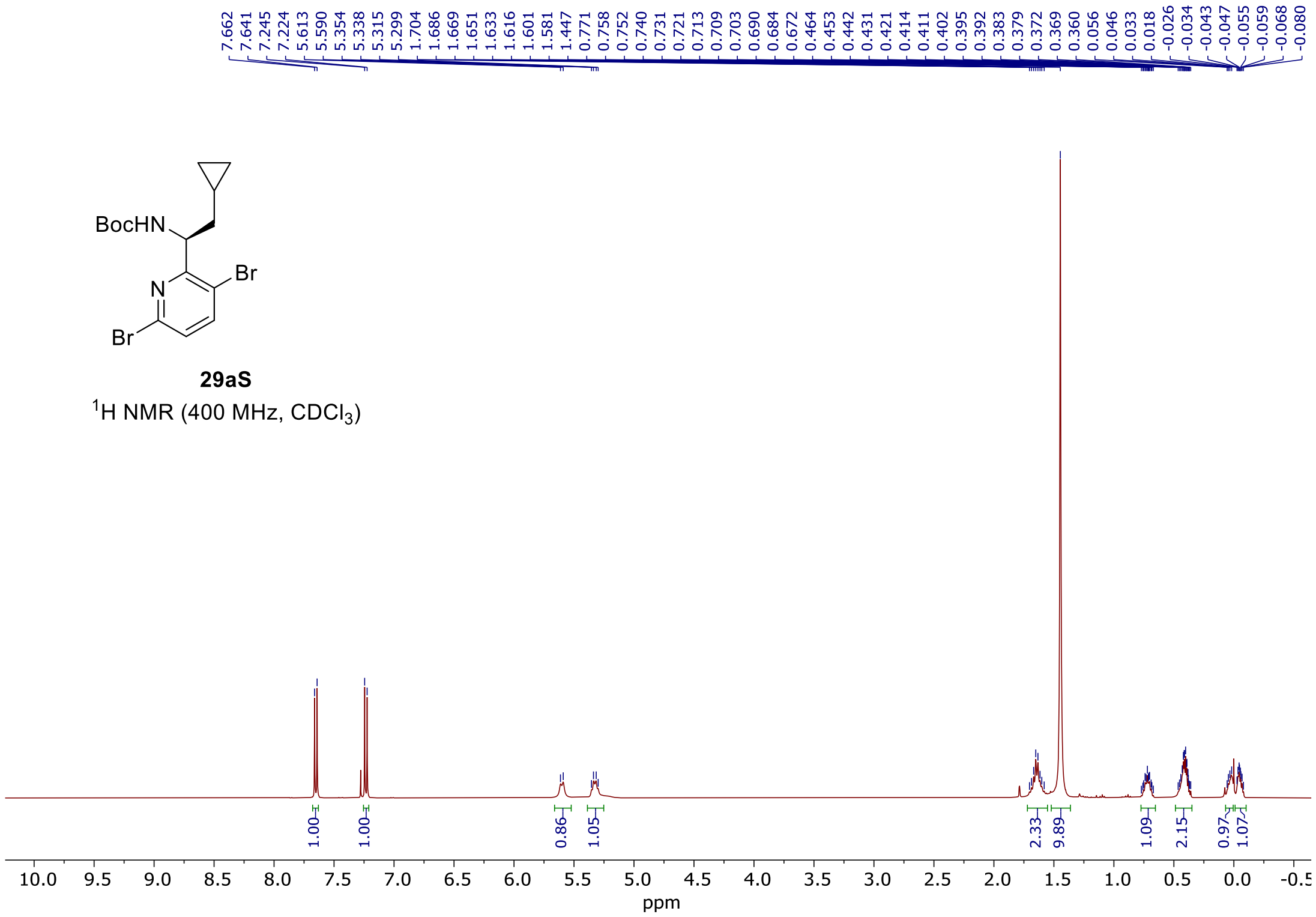


$^{13}$C NMR (100 MHz, $CDCl_3$) of compound **29aS**

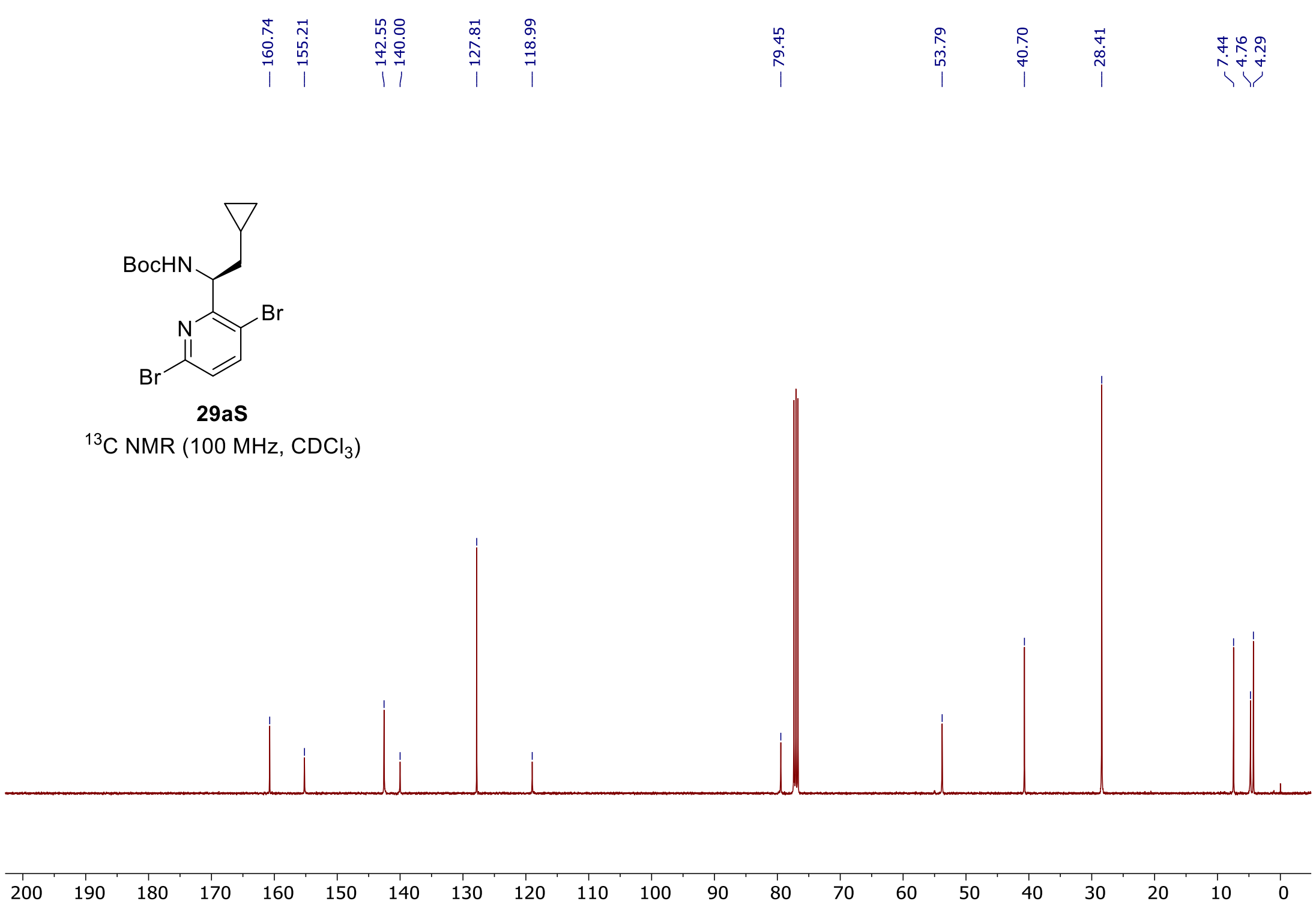

$^{1}H$ NMR (400 MHz, $CDCl_3$) of compound **29aR**

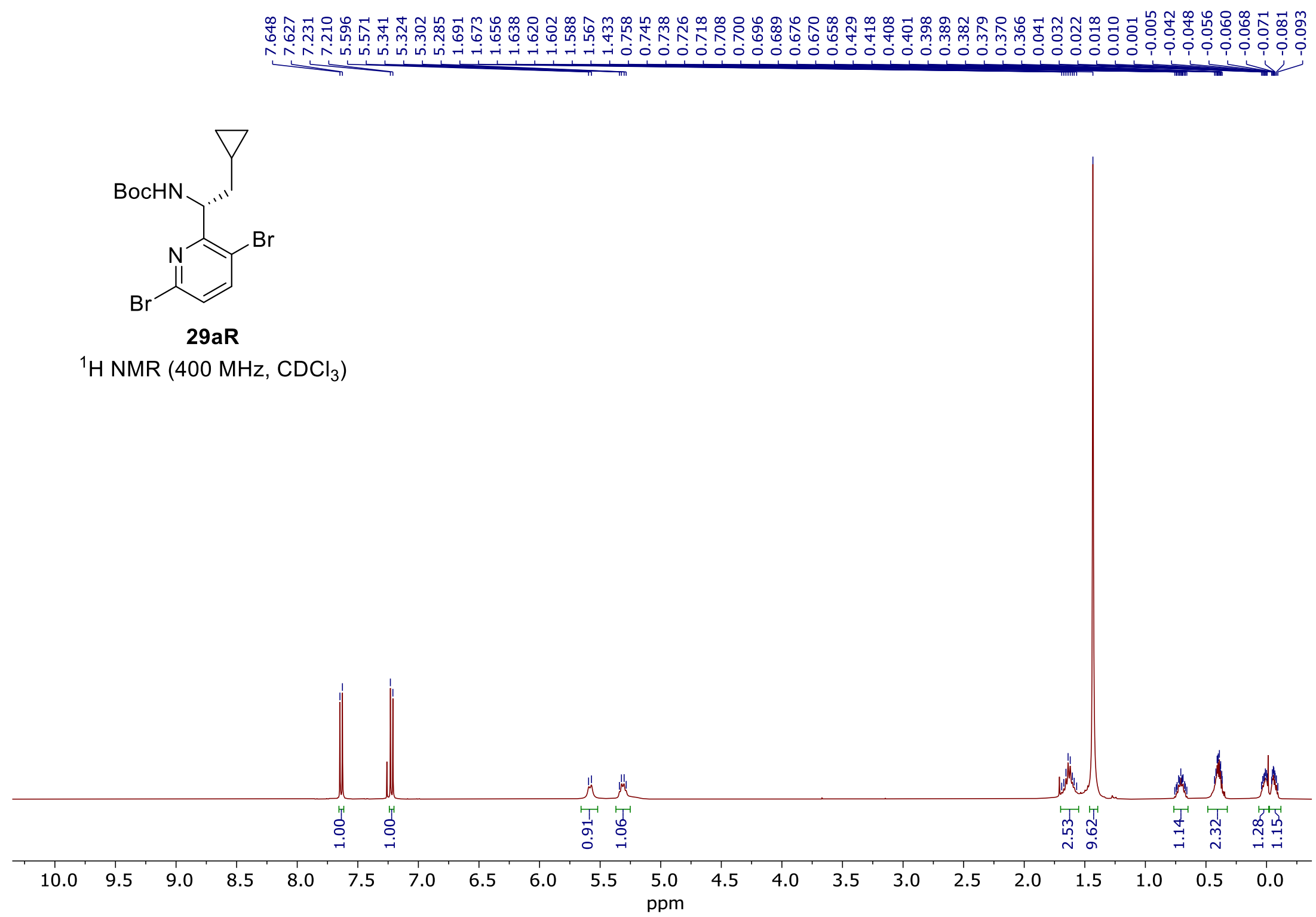

$^{13}C$ NMR (100 MHz, $CDCl_3$) of compound **29aR**

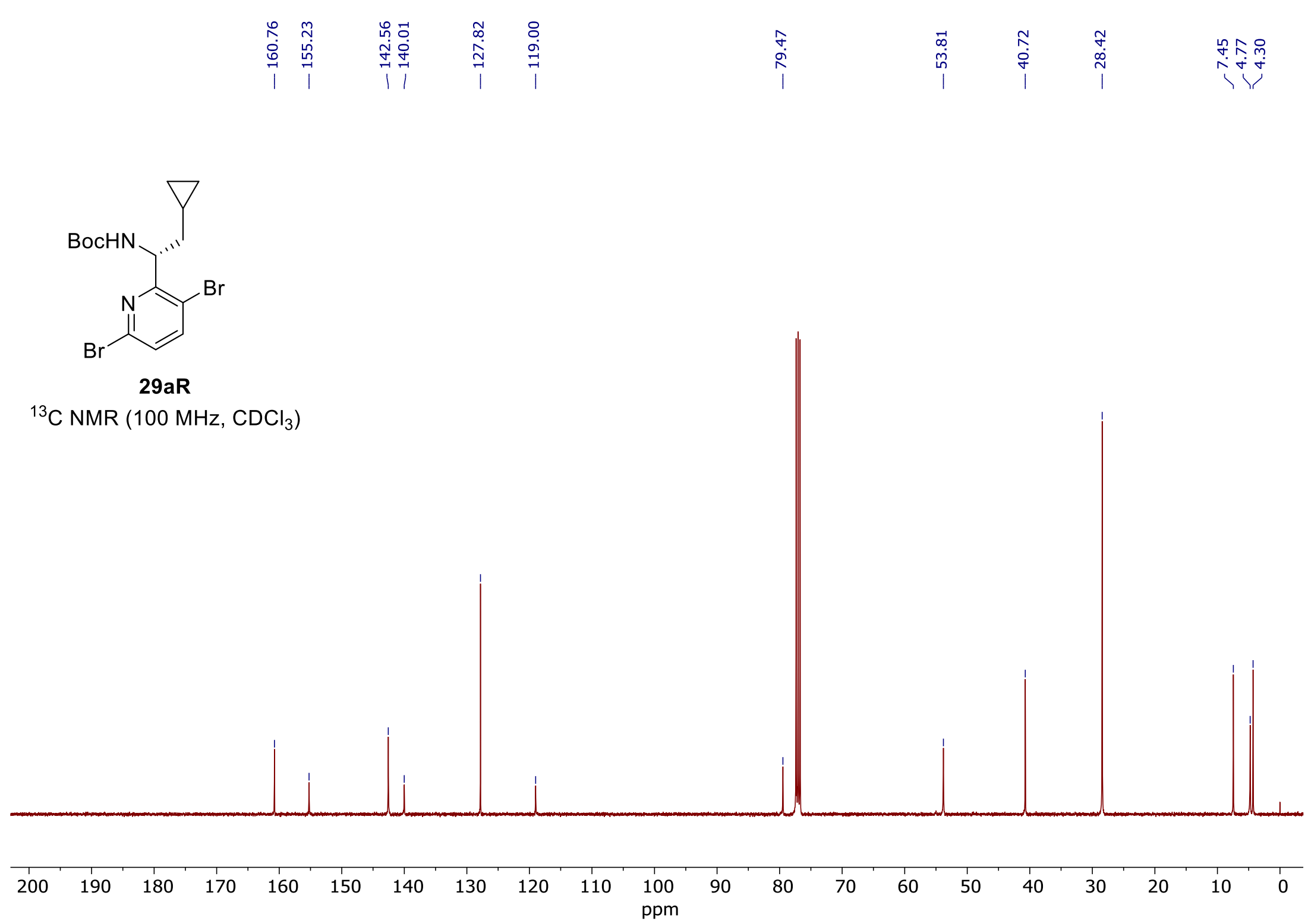

$^{1}$H NMR (400 MHz, $CDCl_3$) of the compound **29bS**

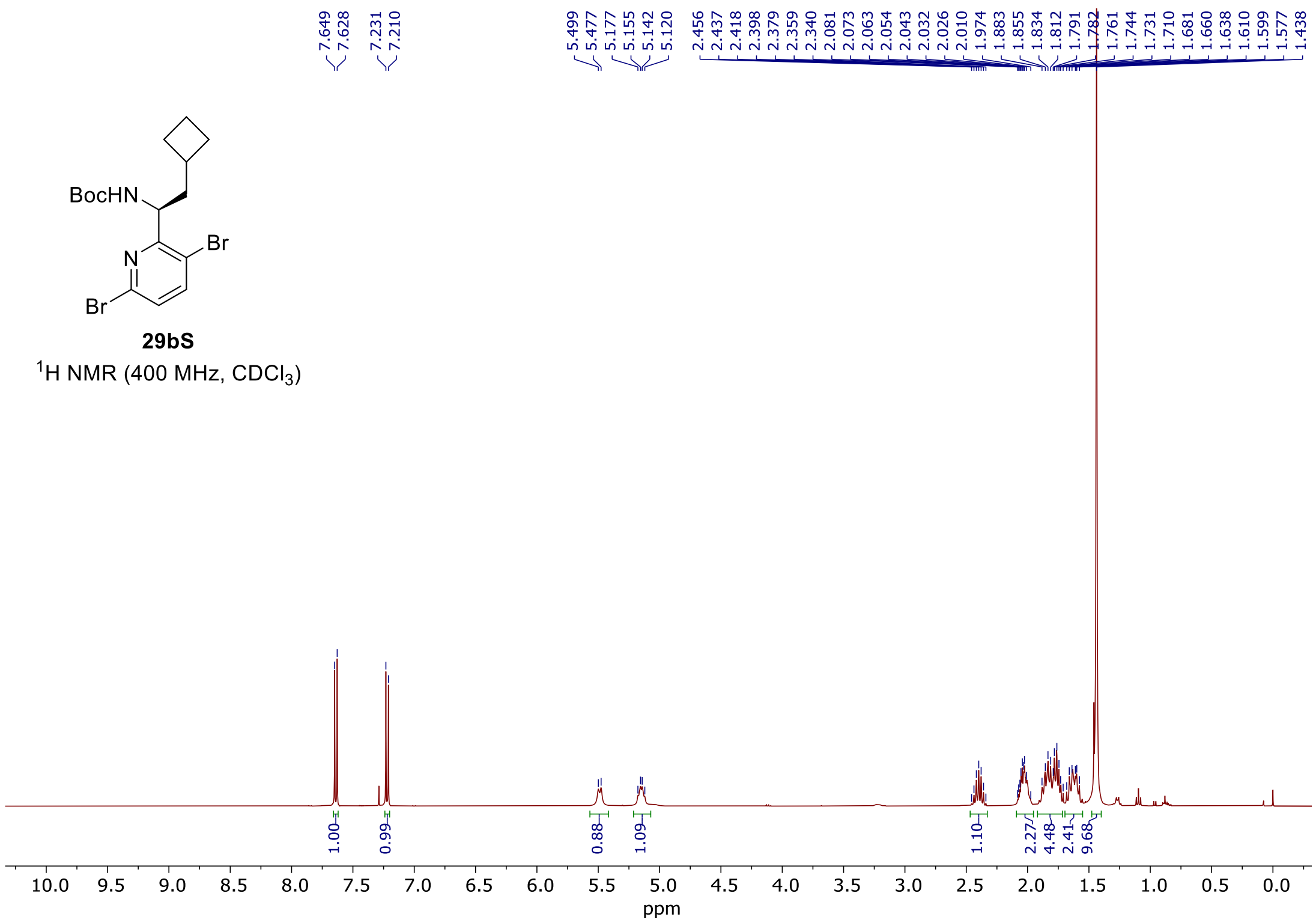


$^{13}$C NMR (100 MHz, $CDCl_3$) of compound **29bS**

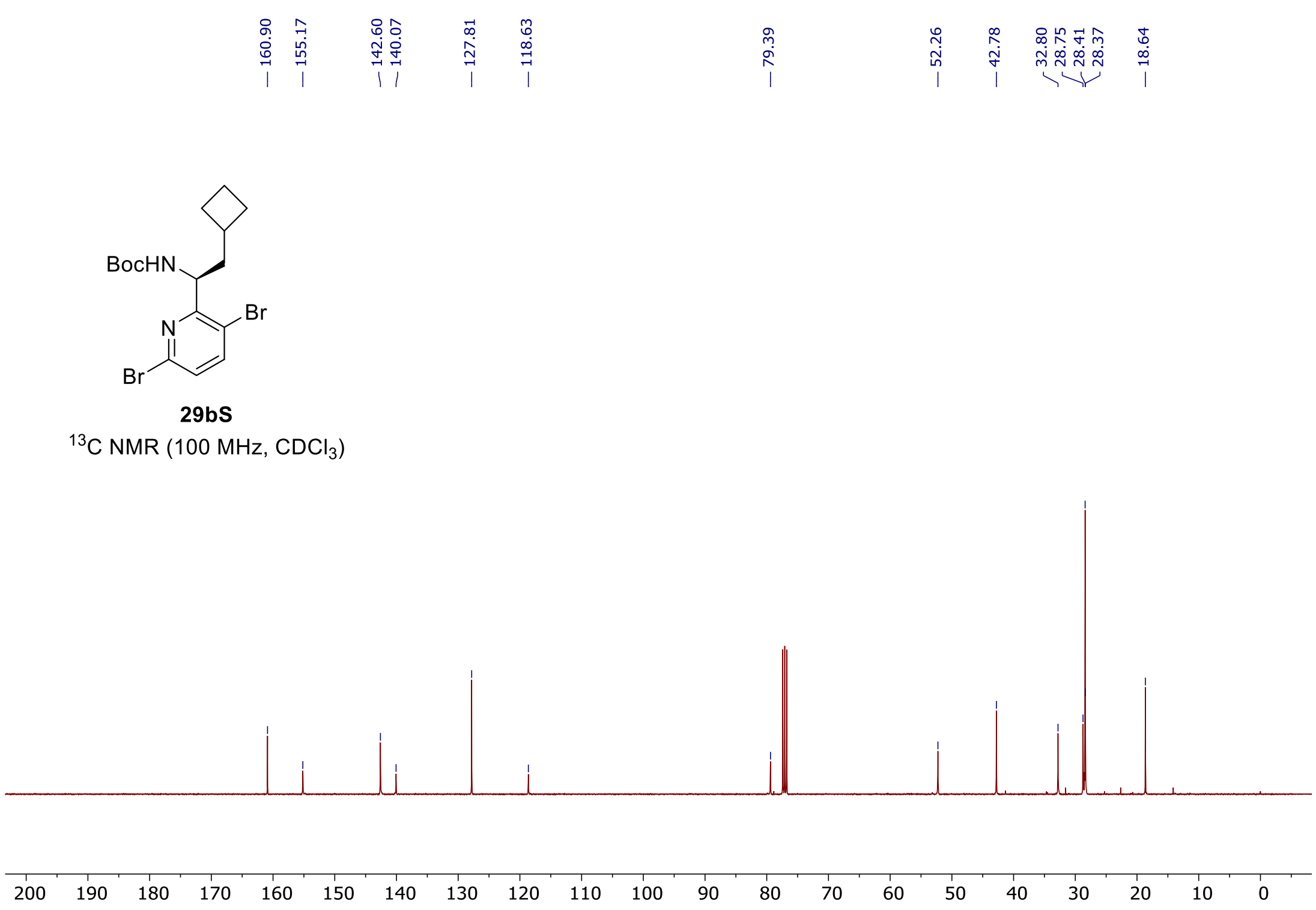

$^{1}$H NMR (400 MHz, $CDCl_3$) of the compound **29bR**

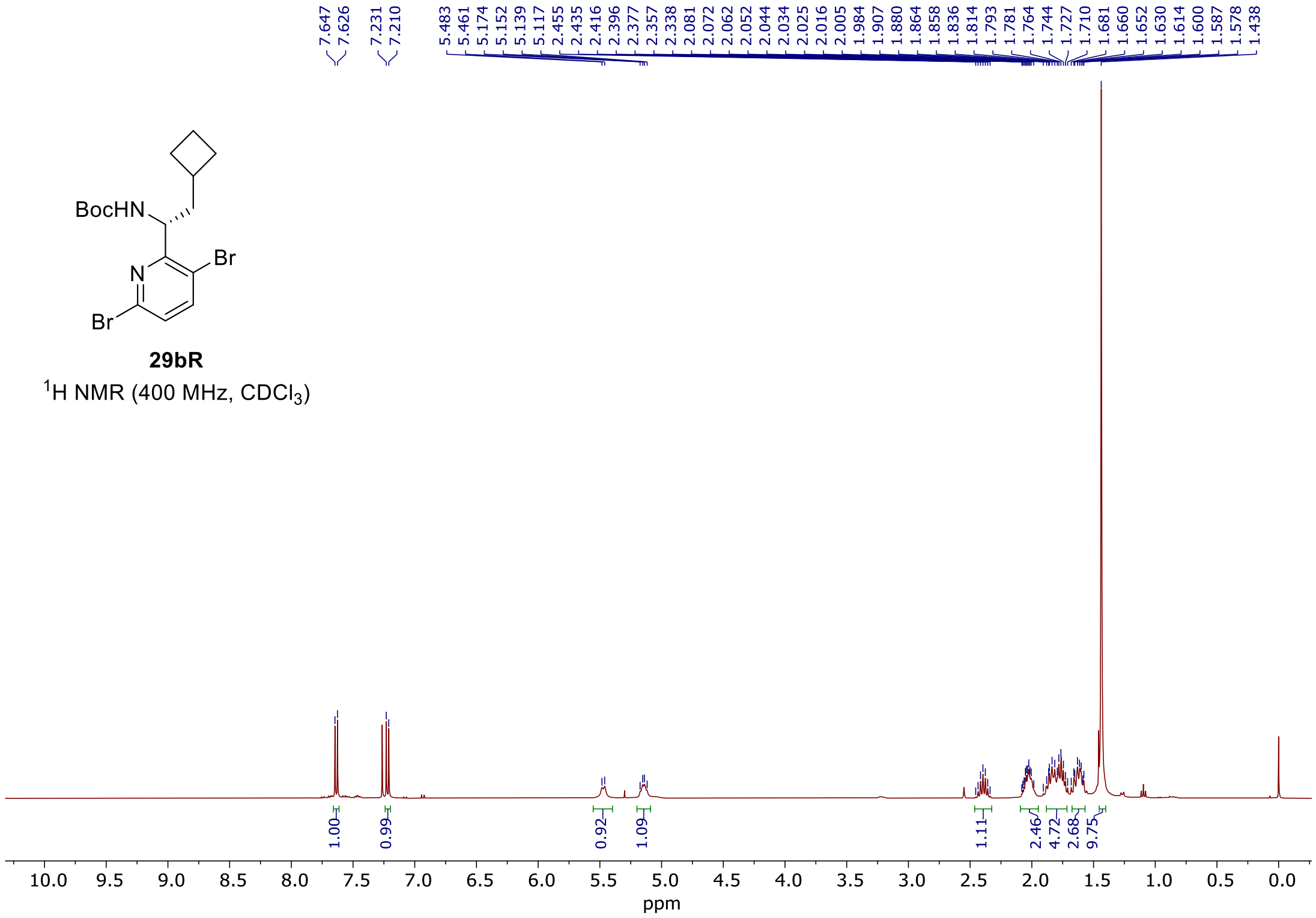


$^{13}$C NMR (100 MHz, $CDCl_3$) of compound **29bR**

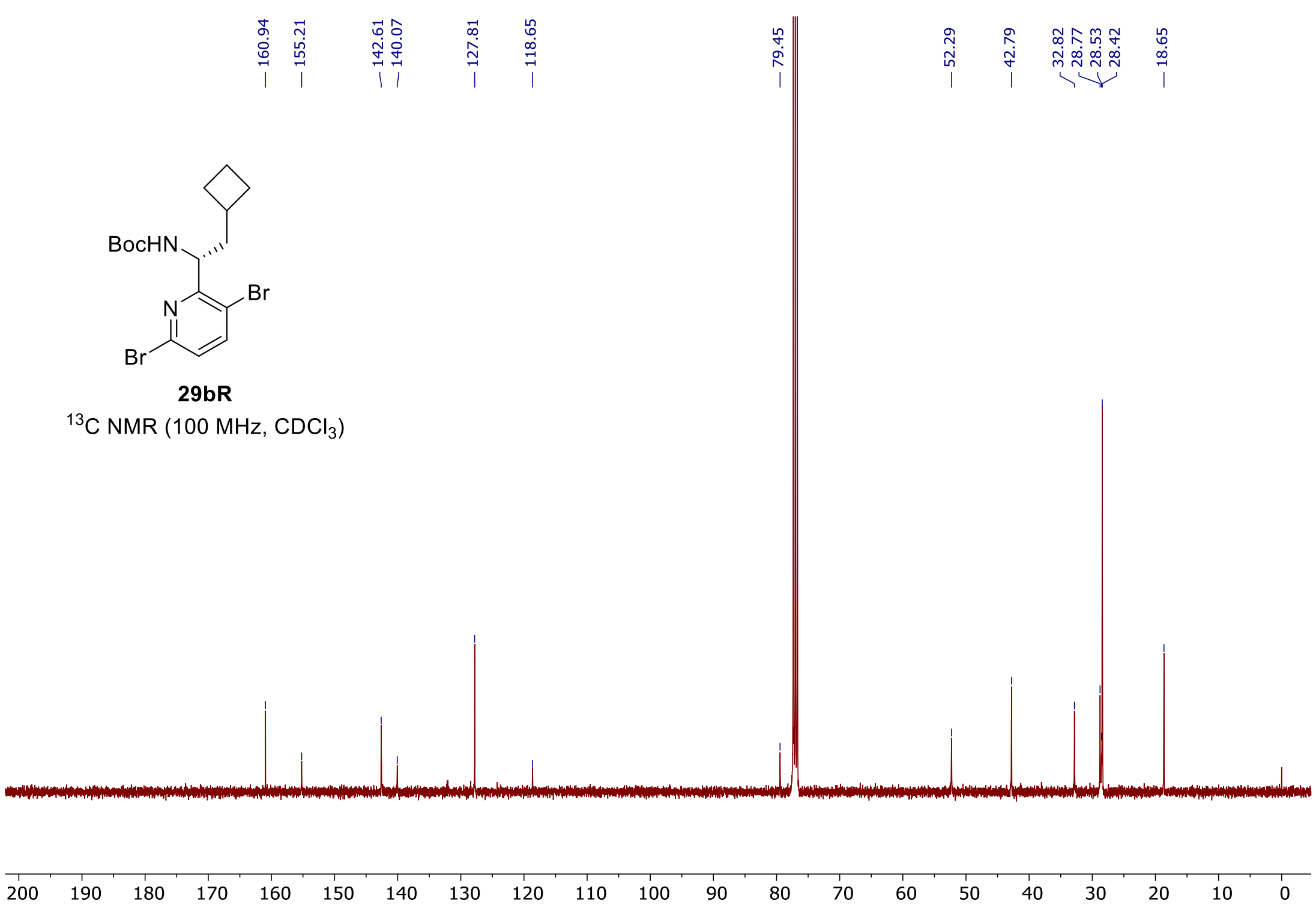

$^{1}H$ NMR (400 MHz, $CDCl_3$) of compound **29cS**

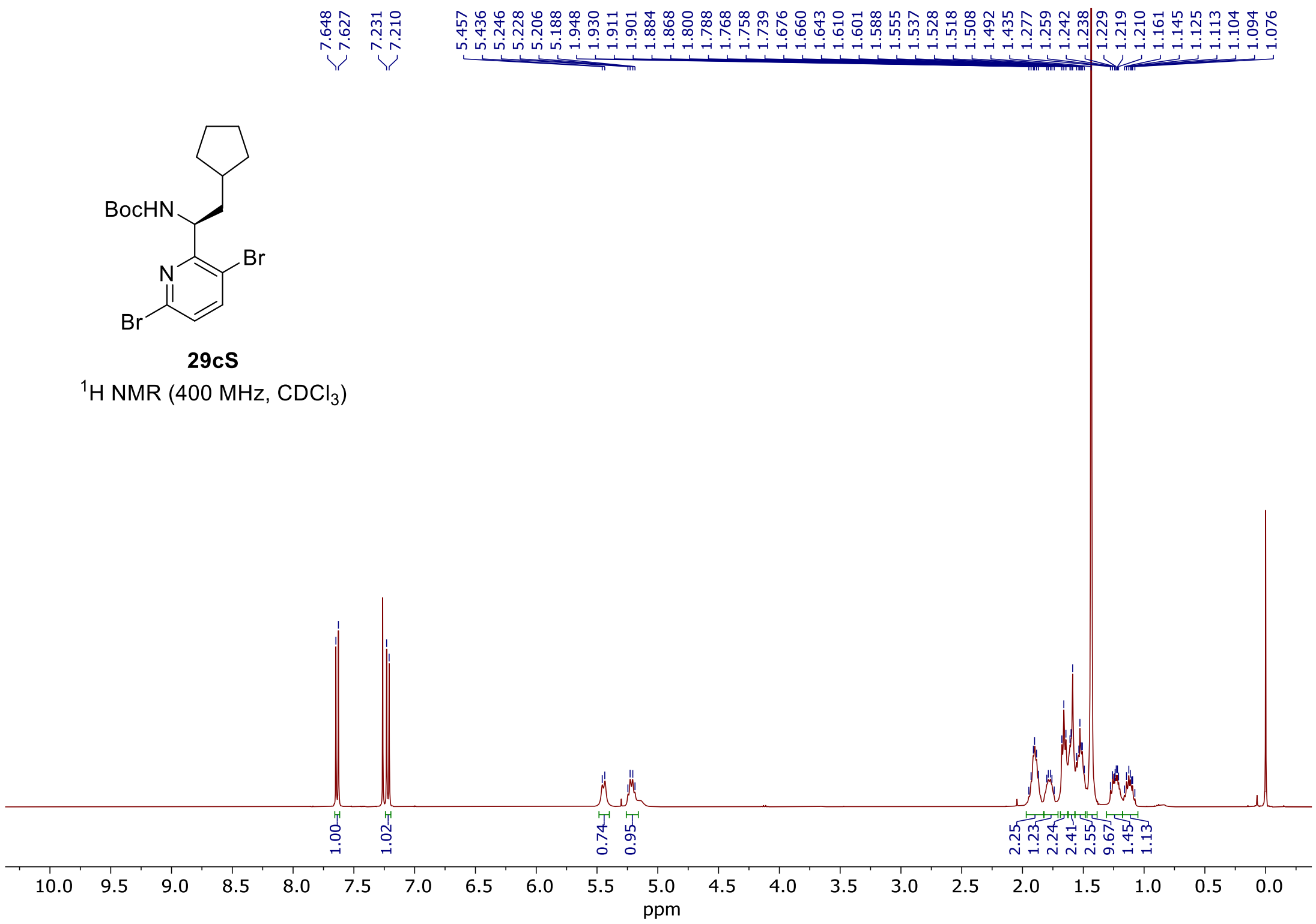


$^{13}C$ NMR (100 MHz, $CDCl_3$) of compound **29cS**

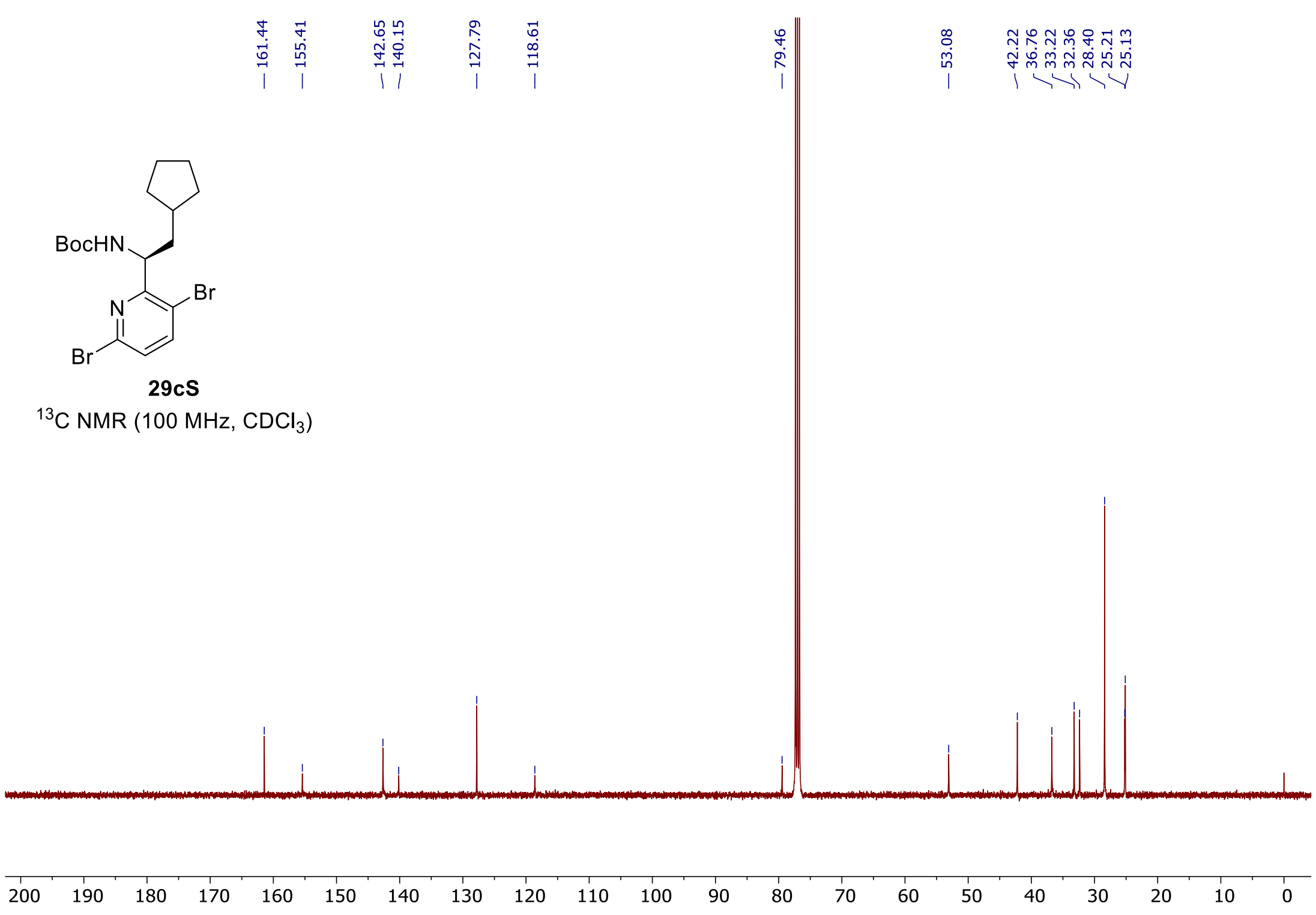

$^{1}H$ NMR (400 MHz, $CDCl_3$) of compound **29cR**

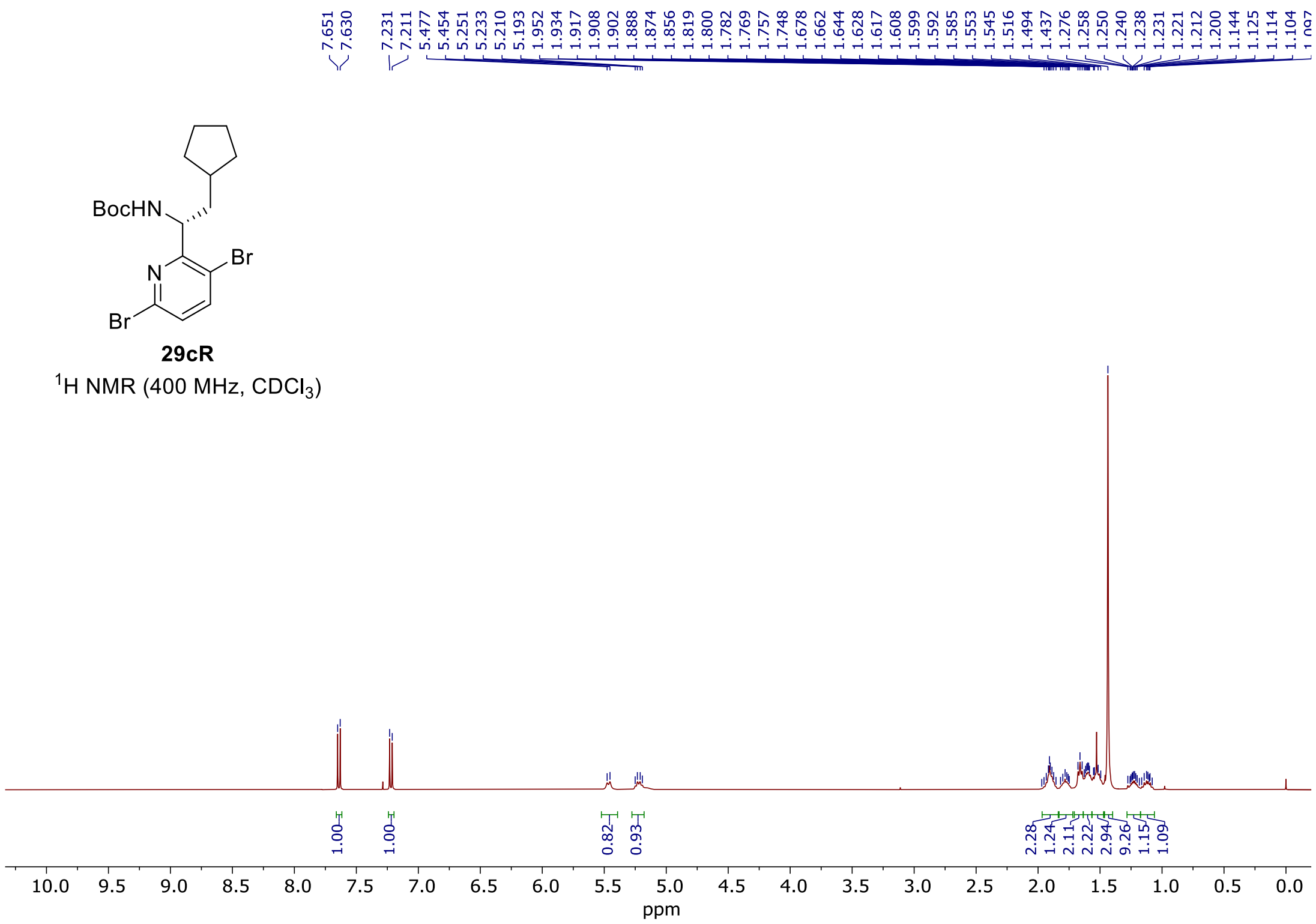


$^{13}C$ NMR (100 MHz, $CDCl_3$) of compound **29cR**

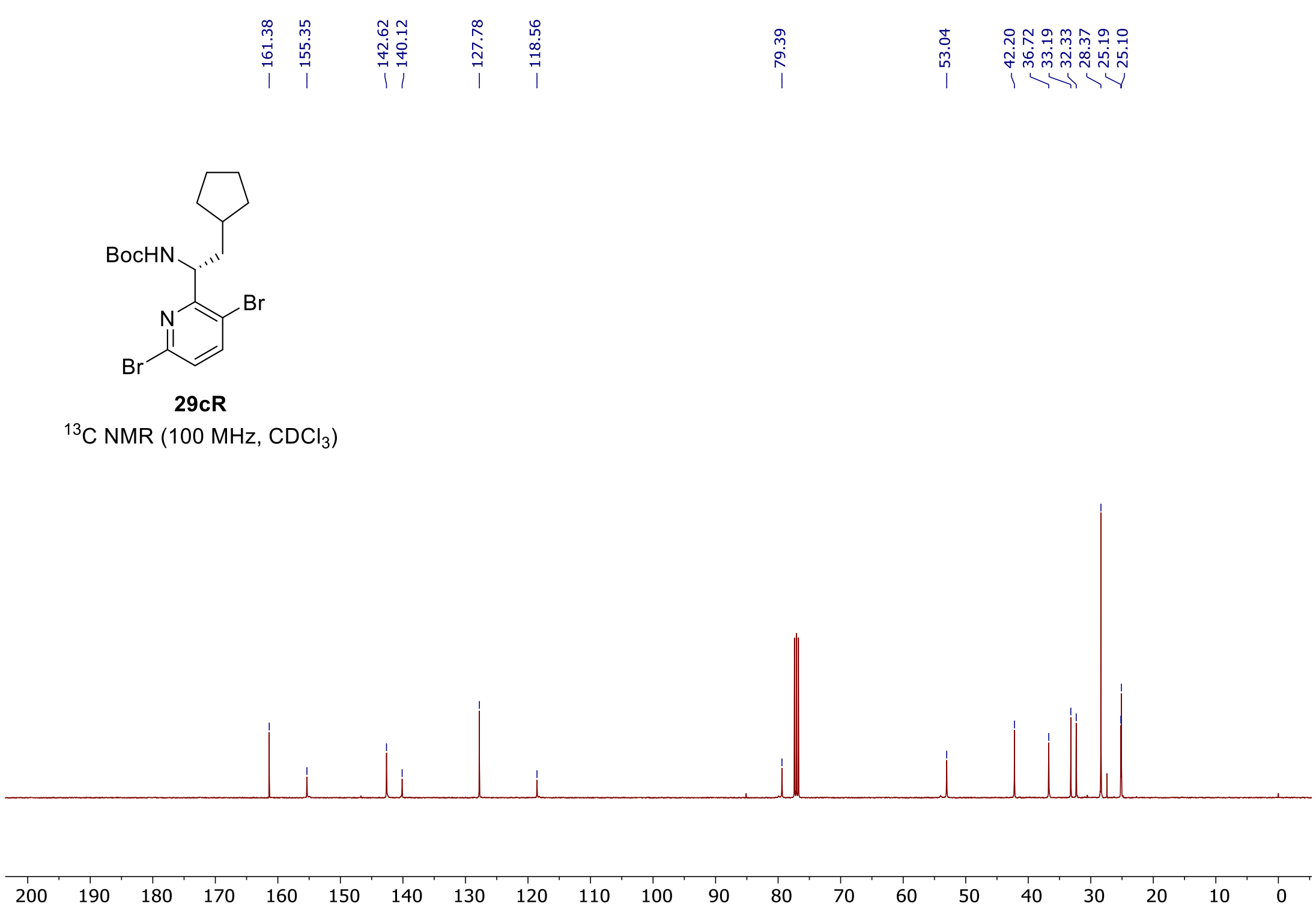

$^{1}H$ NMR (400 MHz, $CDCl_3$) of compound **29dS**

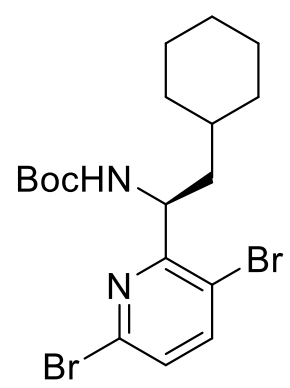

**29dS**

$^{1}H$ NMR (400 MHz, $CDCl_3$)

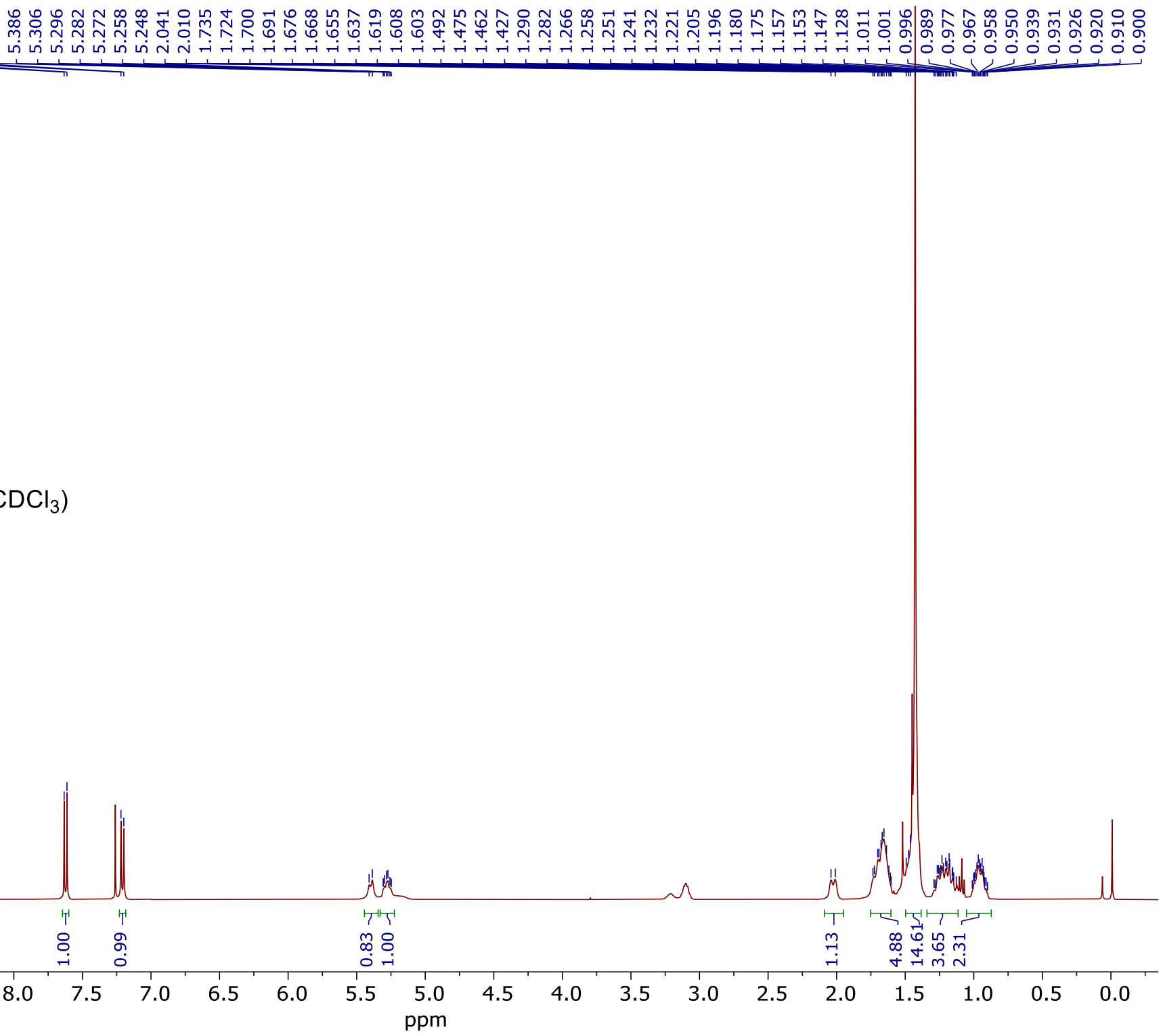


$^{13}C$ NMR (100 MHz, $CDCl_3$) of compound **29dS**

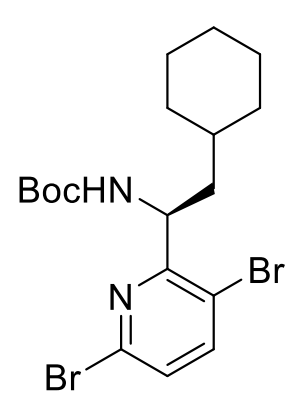

**29dS**

$^{13}C$ NMR (100 MHz, $CDCl_3$)

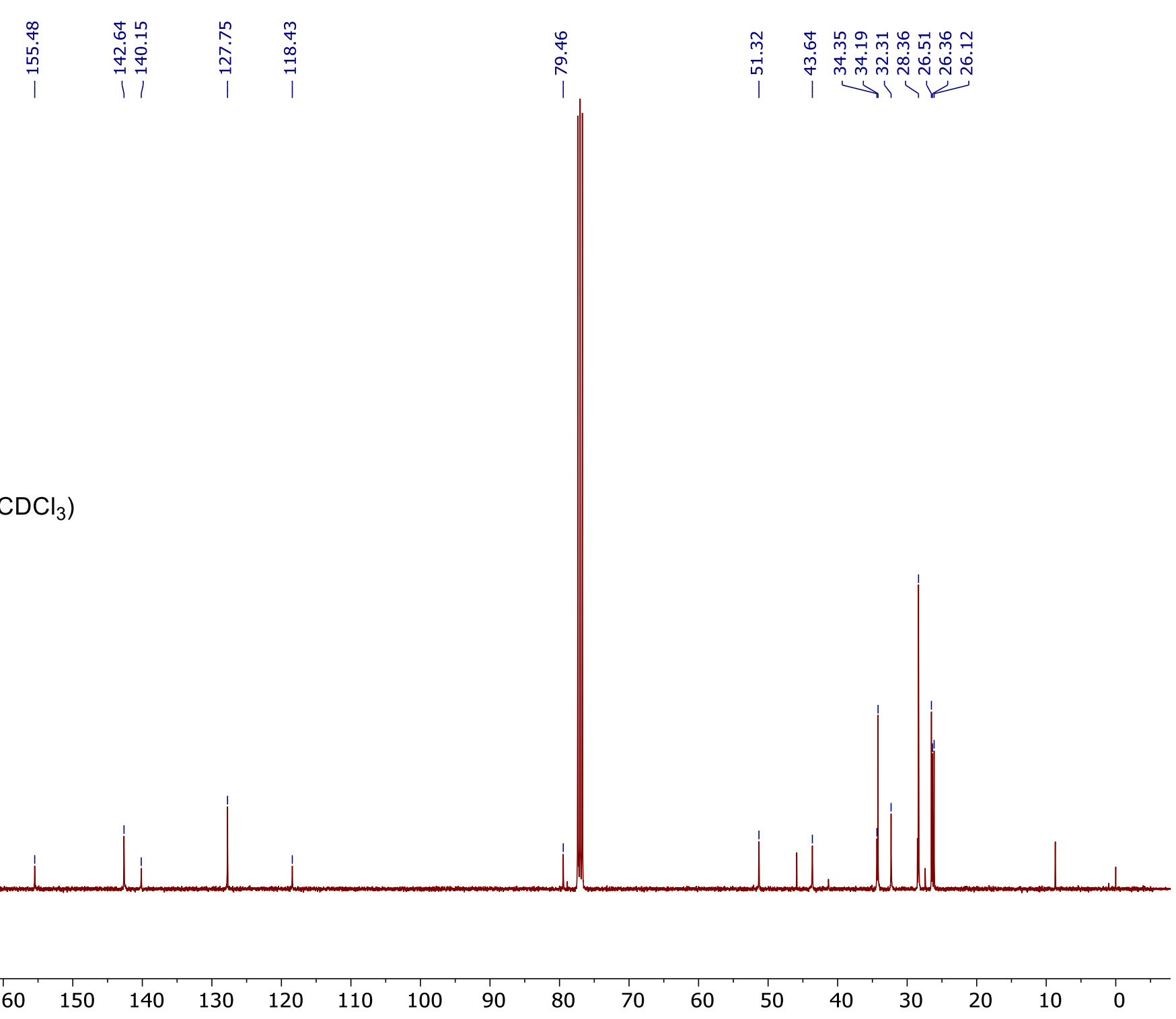

$^{1}H$ NMR (400 MHz, $CDCl_3$) of compound **29dR**

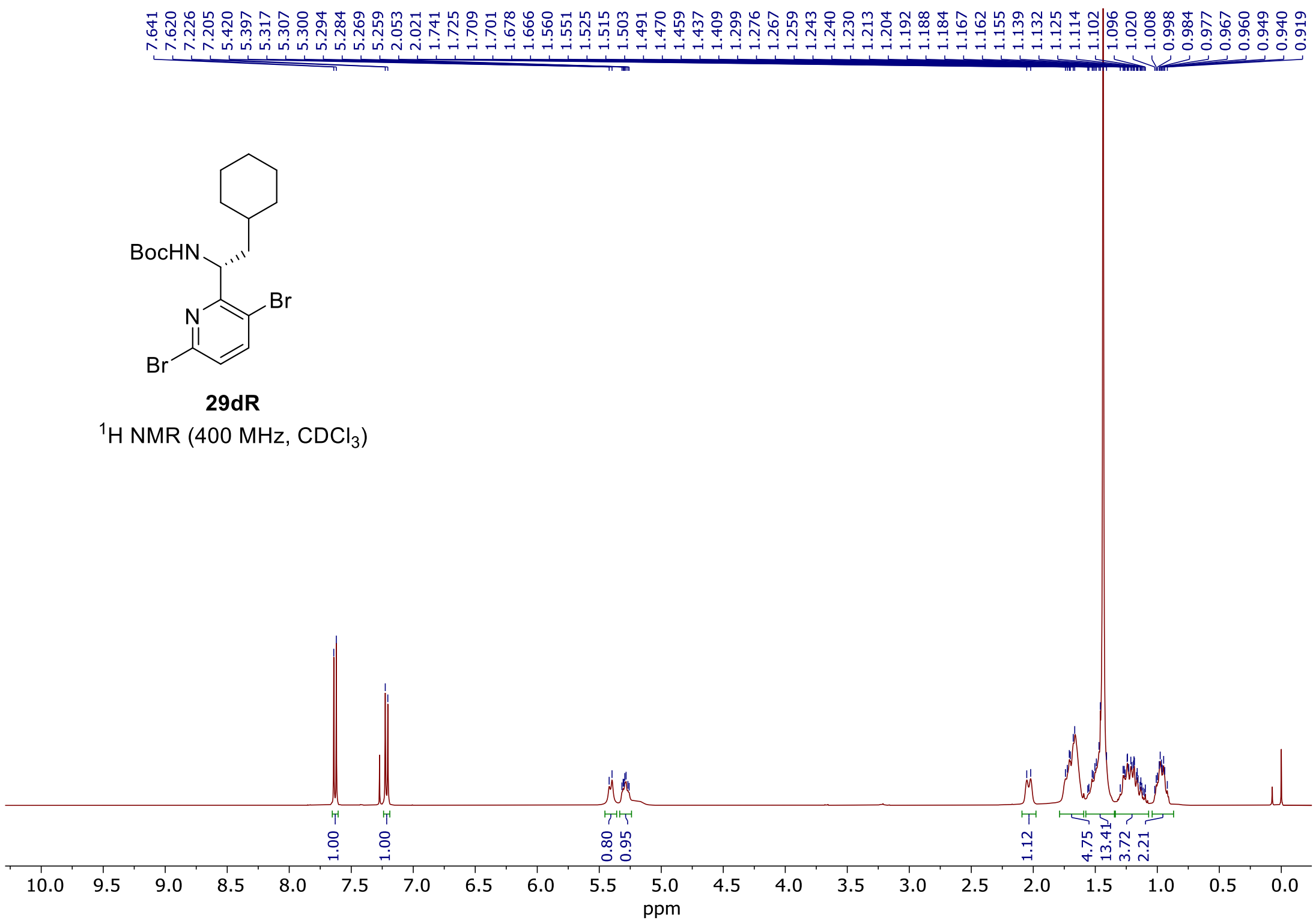


$^{13}C$ NMR (100 MHz, $CDCl_3$) of compound **29dR**

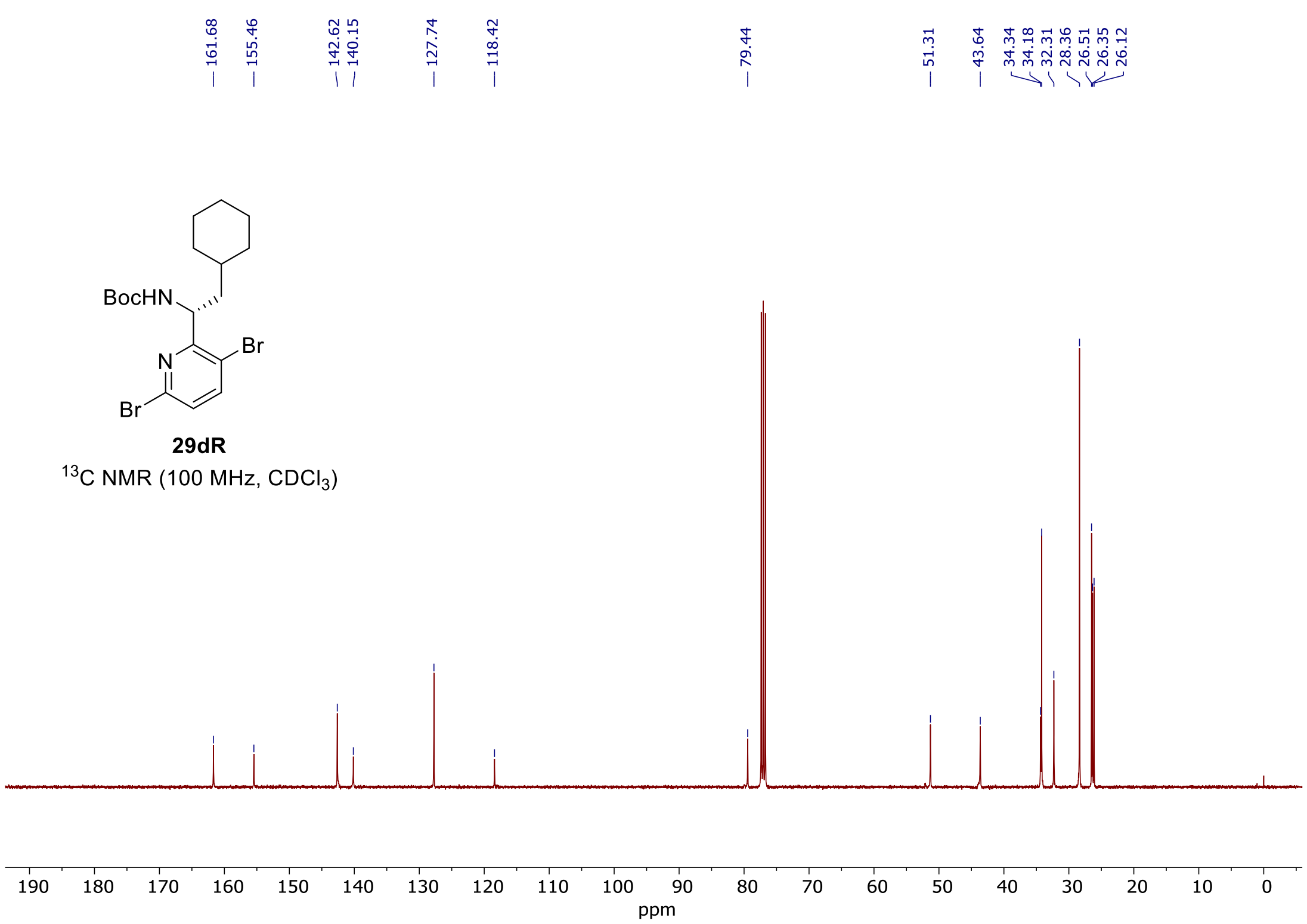

$^{1}$H NMR (400 MHz, $CDCl_3$) of compound **30aS**

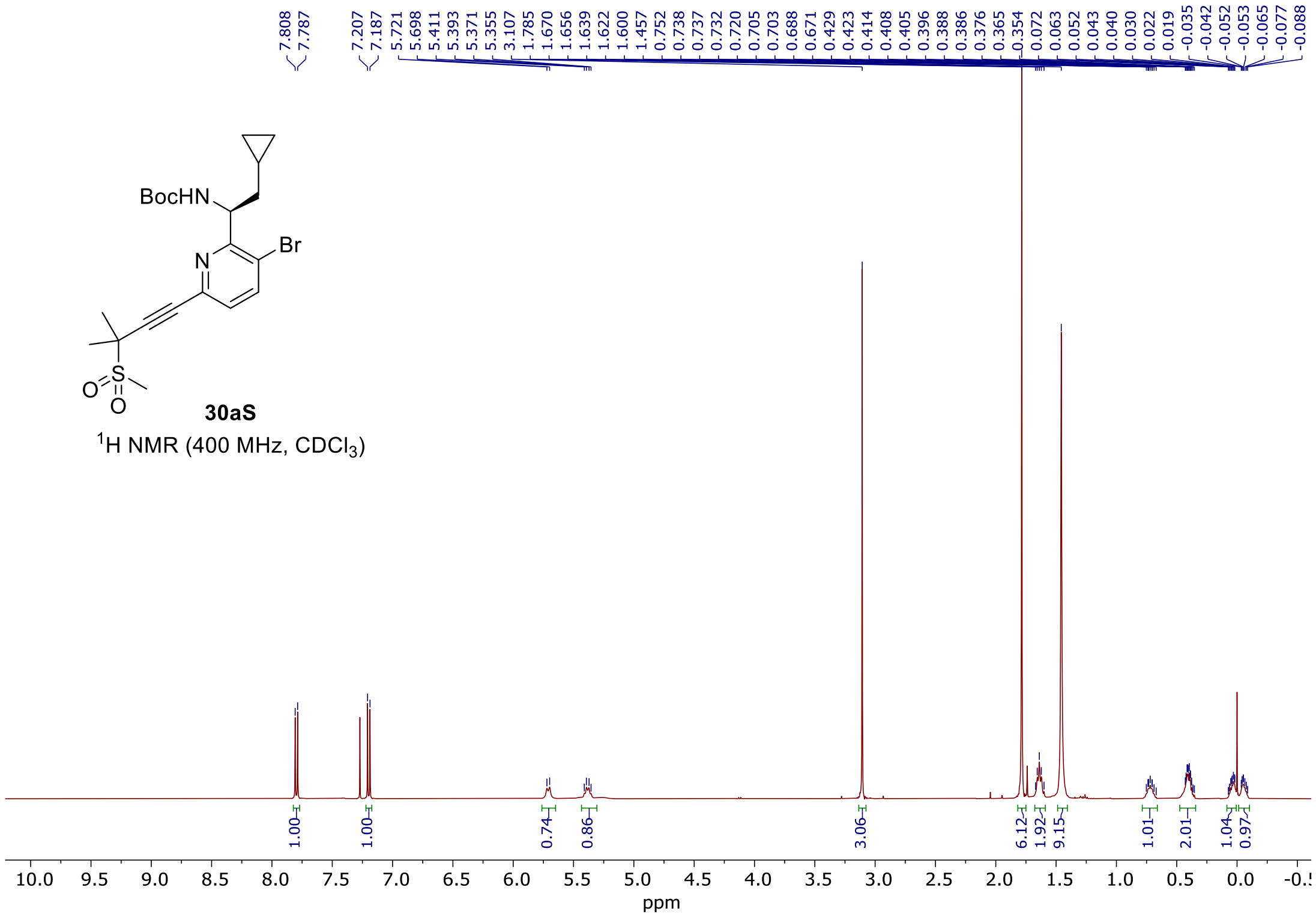


$^{13}$C NMR (100 MHz, $CDCl_3$) of compound **30aS**

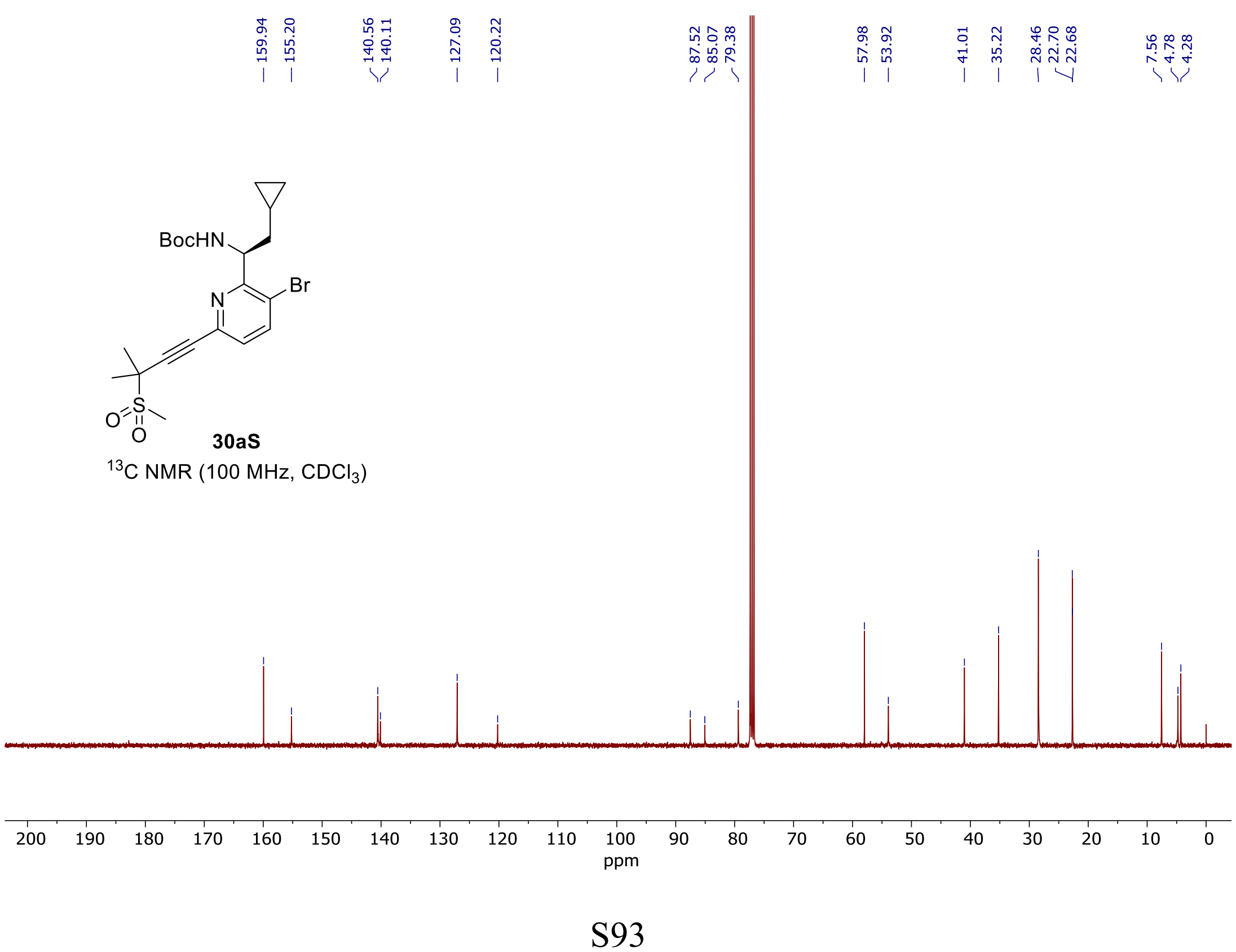

## $^{1}H$ NMR (400 MHz, $CDCl_3$) of the compound **30bS**

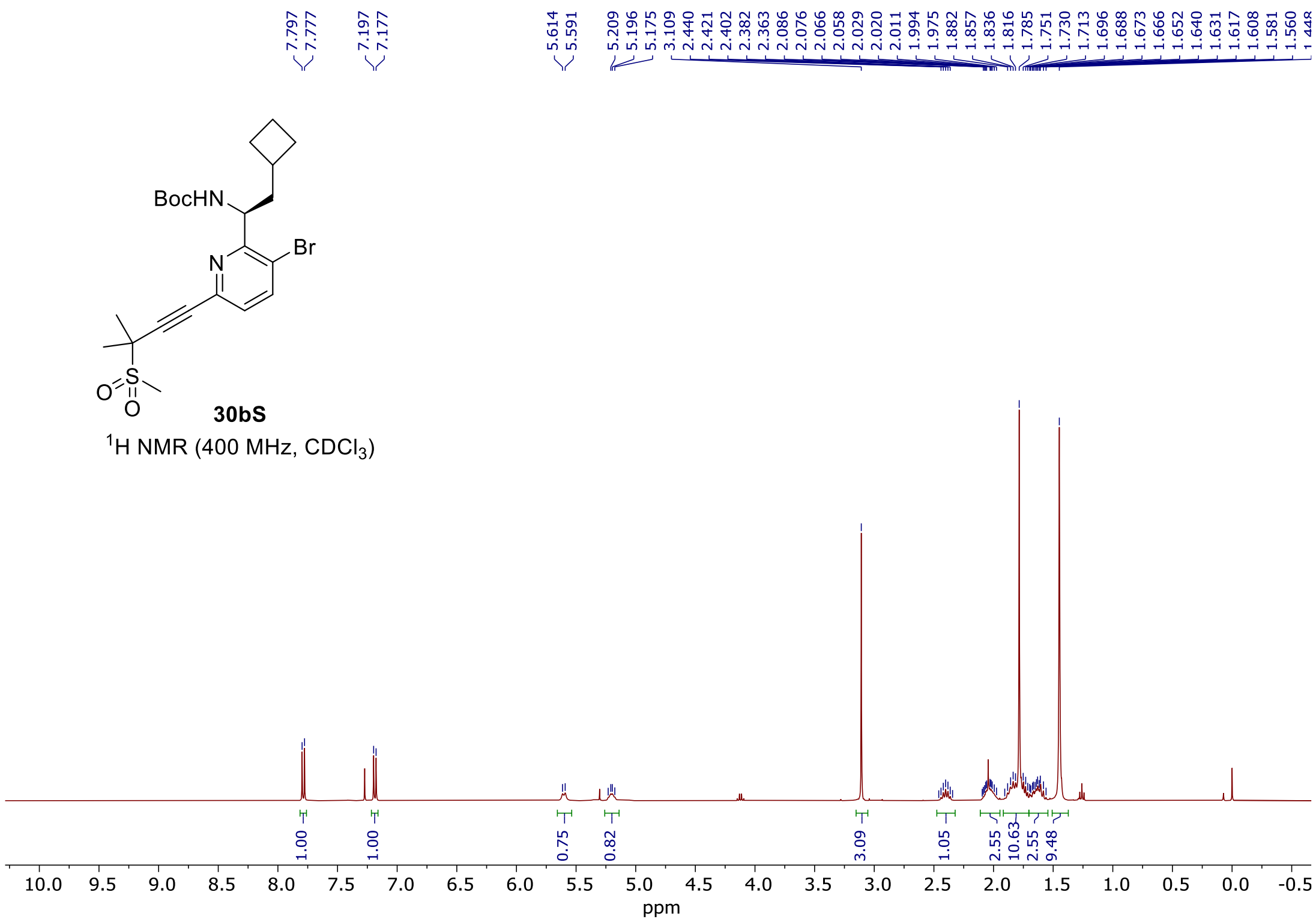


## $^{13}C$ NMR (100 MHz, $CDCl_3$) of compound **30bS**

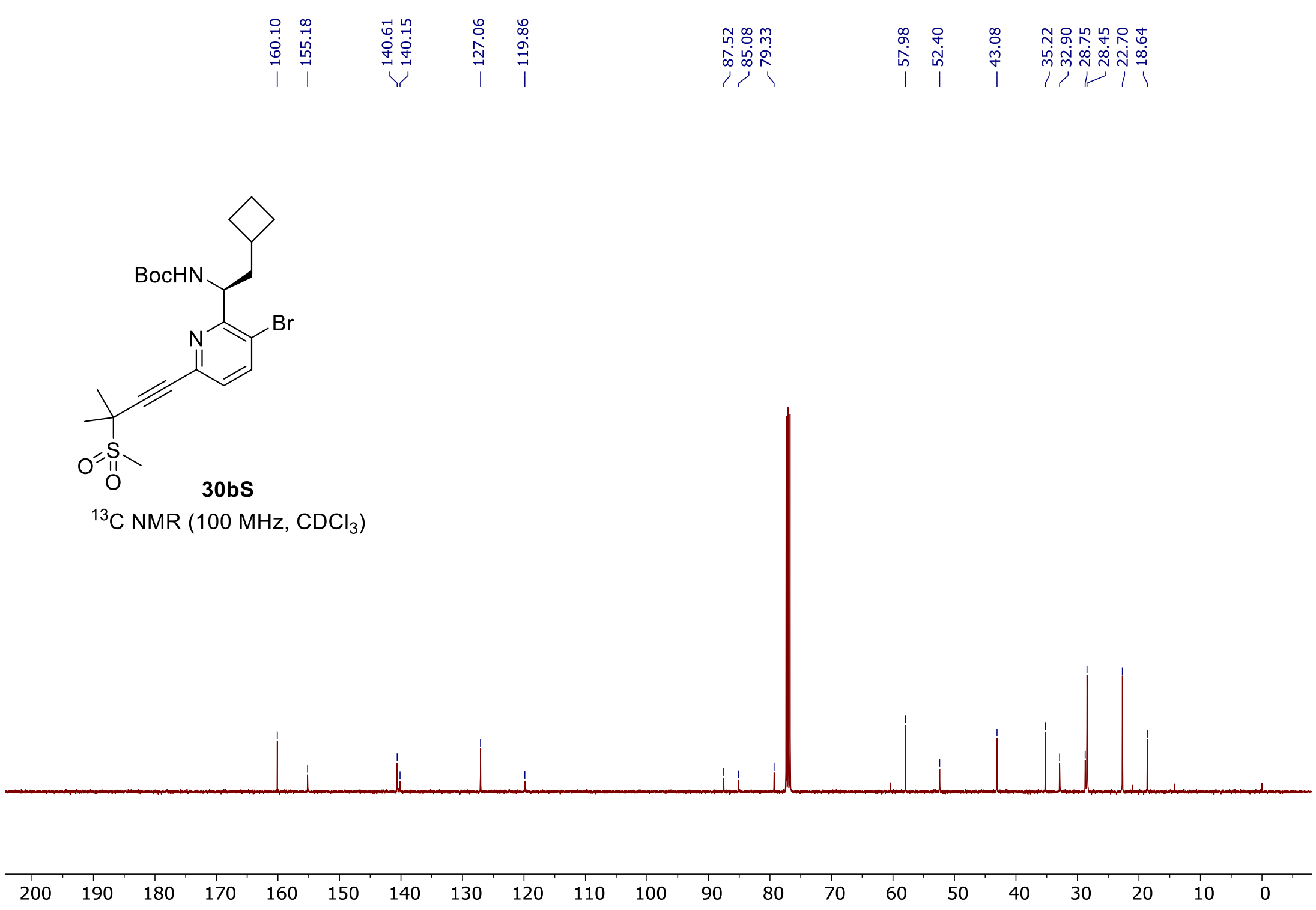

$^{1}H$ NMR (400 MHz, $CDCl_3$) of the compound **30bR**

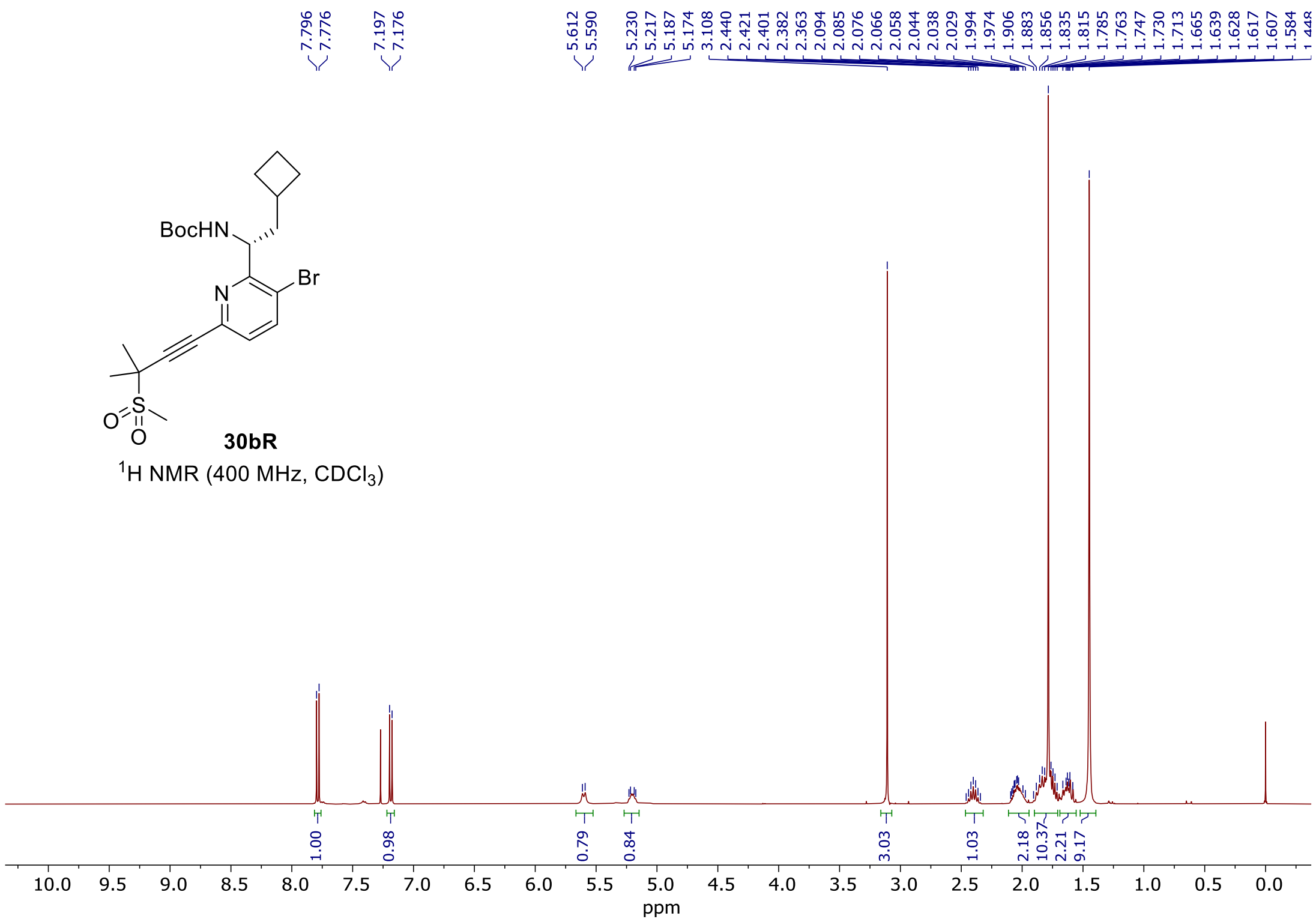

$^{13}C$ NMR (100 MHz, $CDCl_3$) of compound **30bR**

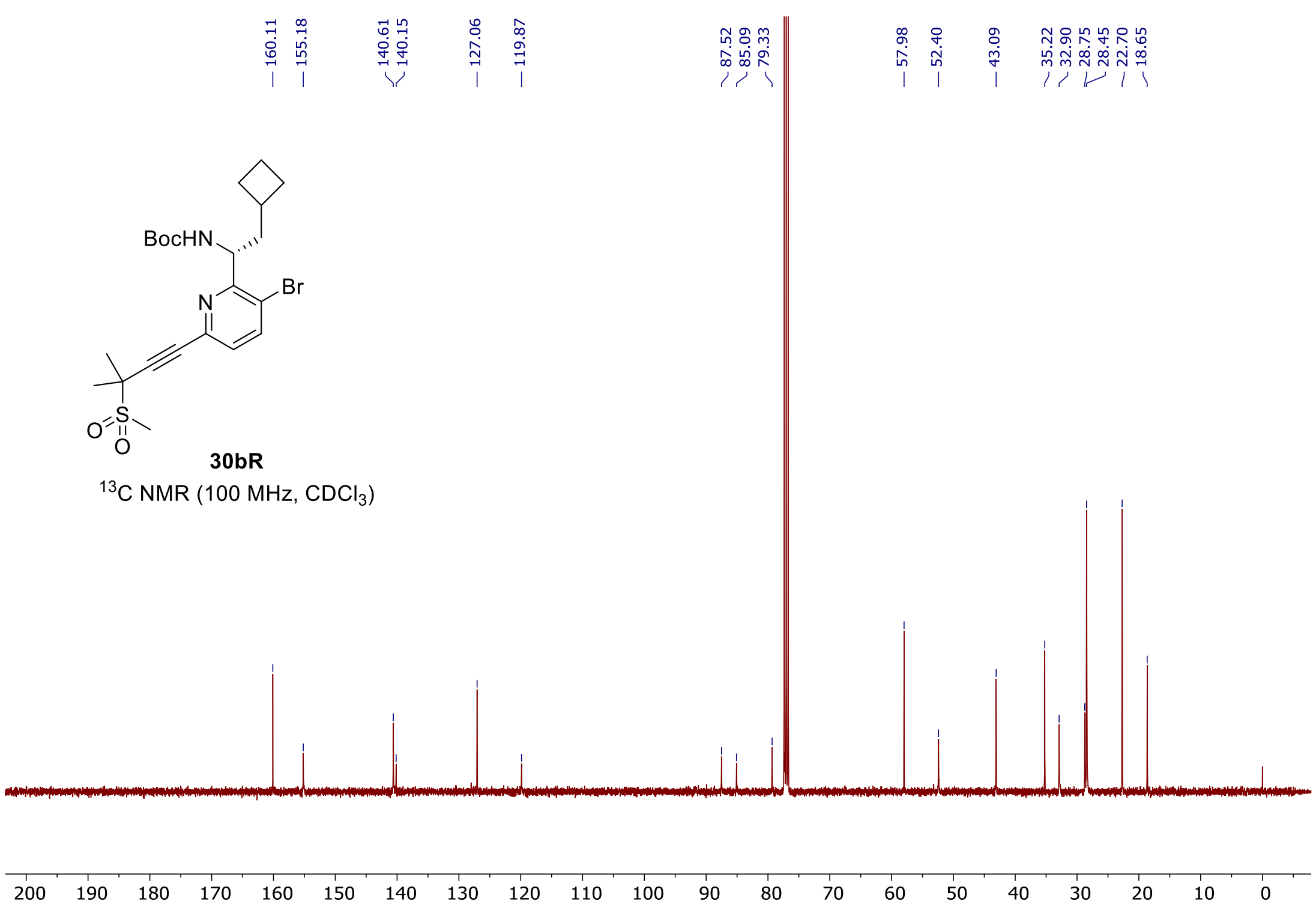

$^{1}$H NMR (400 MHz, $CDCl_3$) of compound **30cS**

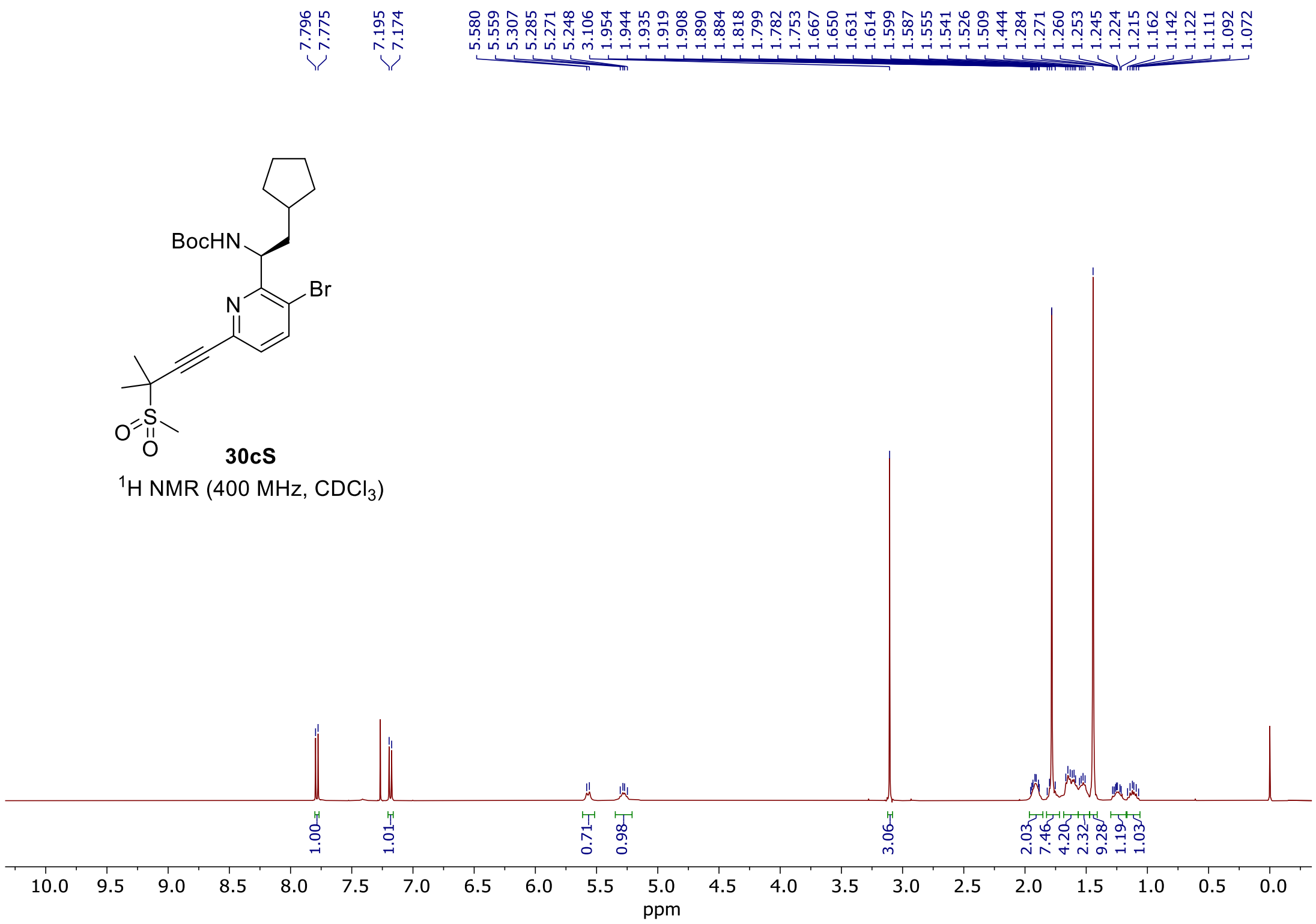

$^{13}$C NMR (100 MHz, $CDCl_3$) of compound **30cS**

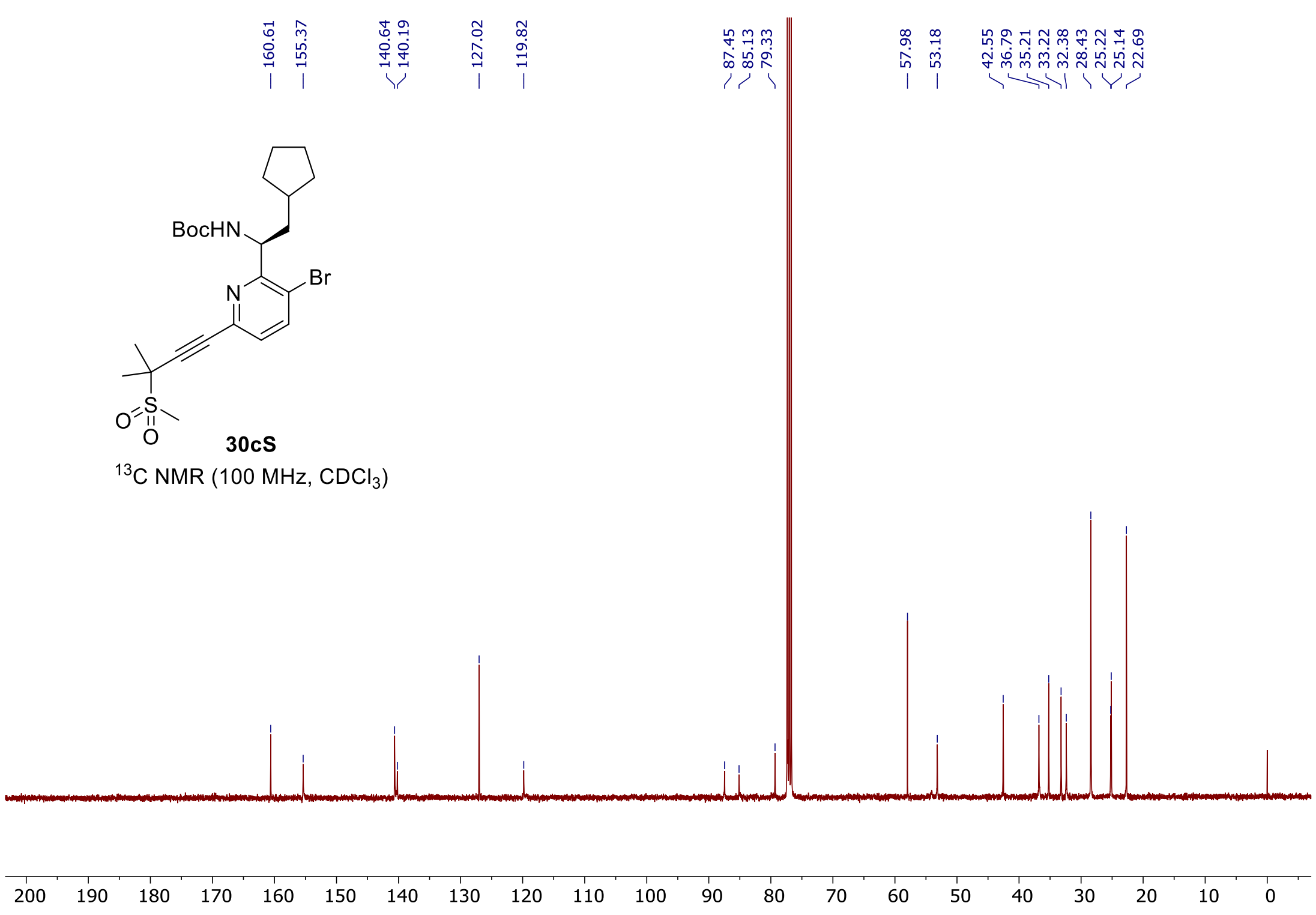

$^{1}$H NMR (400 MHz, CDCl$_3$) of compound **30cR**

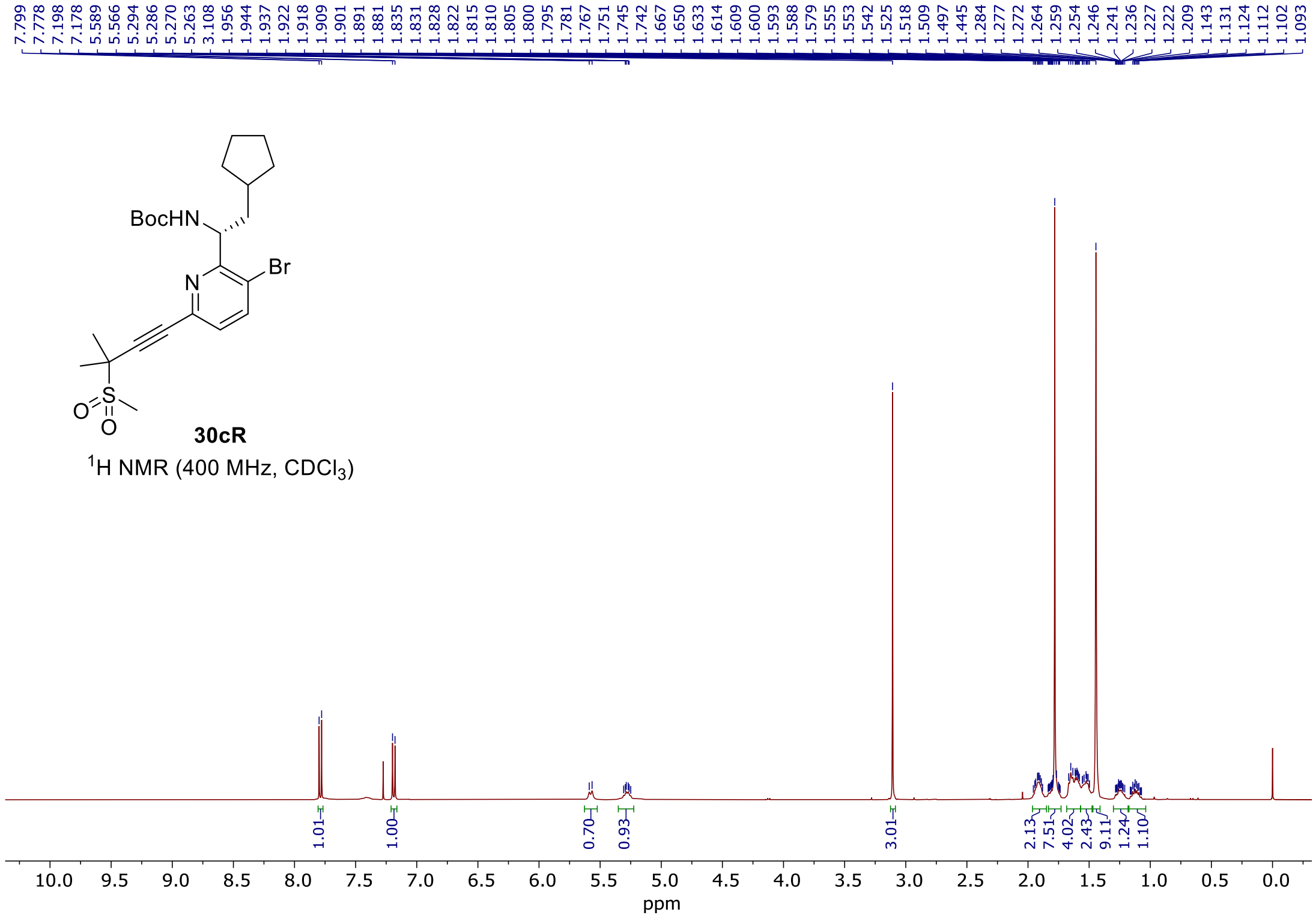


$^{13}$C NMR (100 MHz, CDCl$_3$) of compound **30cR**

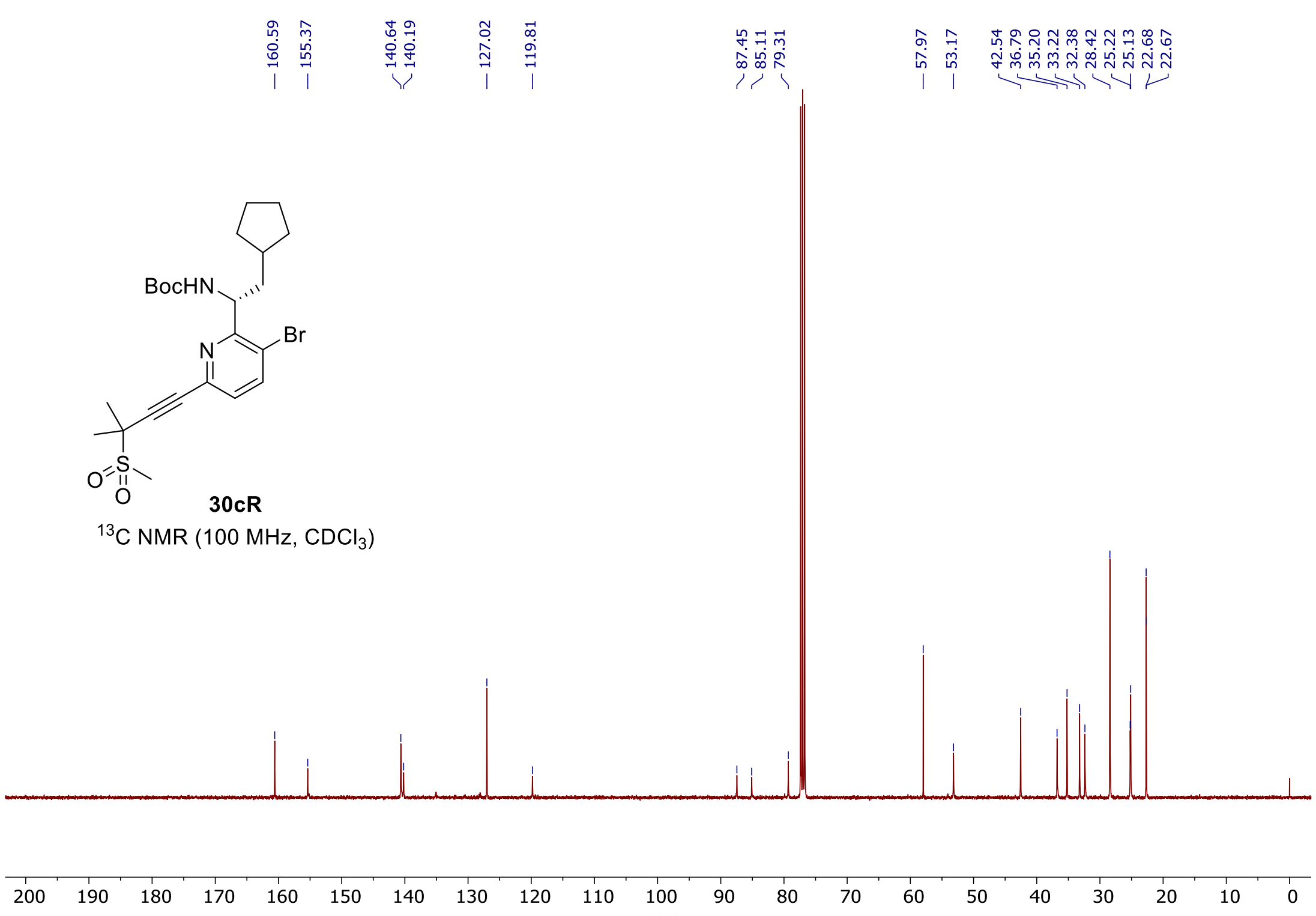

$^{1}H$ NMR (400 MHz, $CDCl_3$) of compound **30dS**

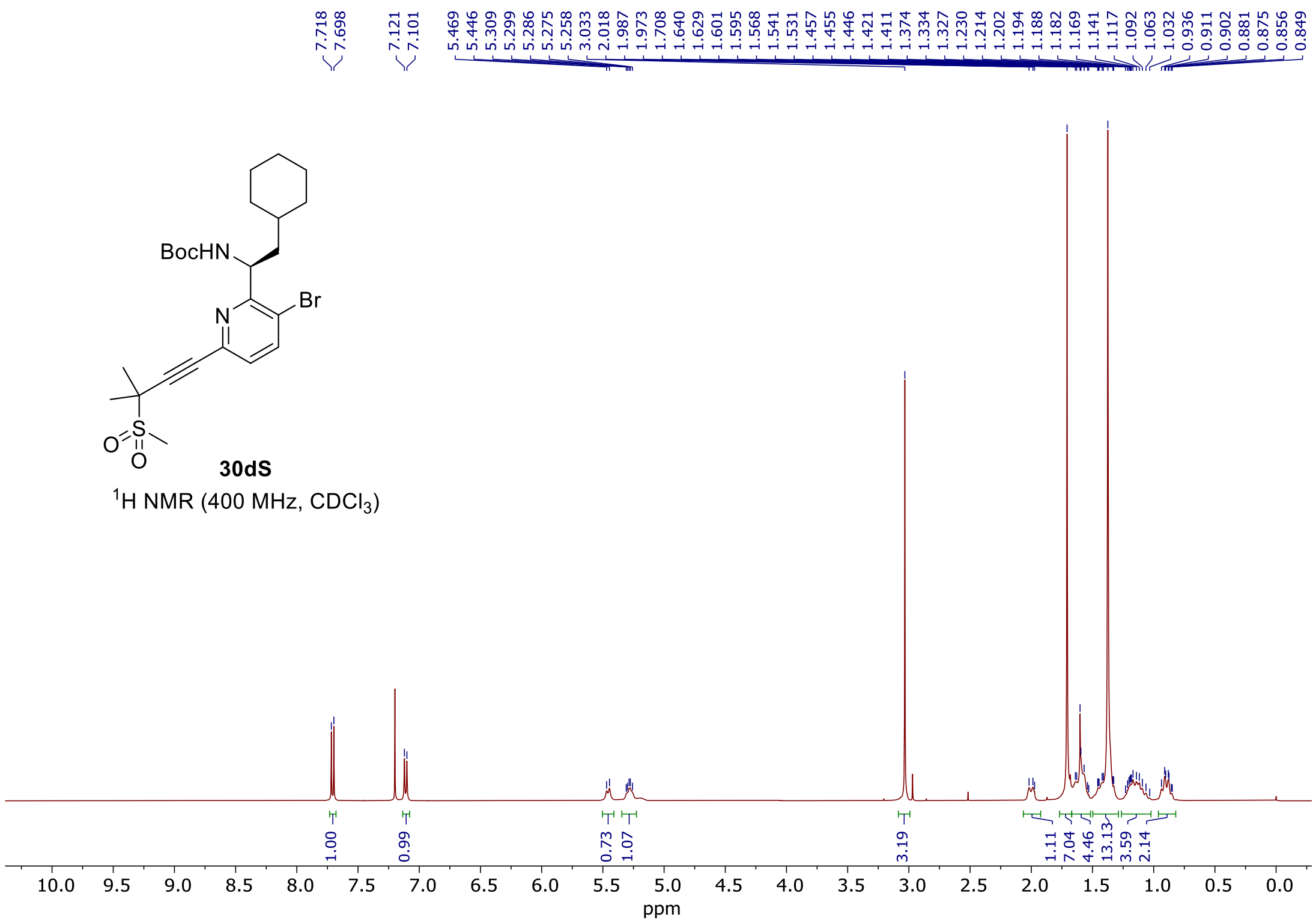


$^{13}C$ NMR (100 MHz, $CDCl_3$) of compound **30dS**

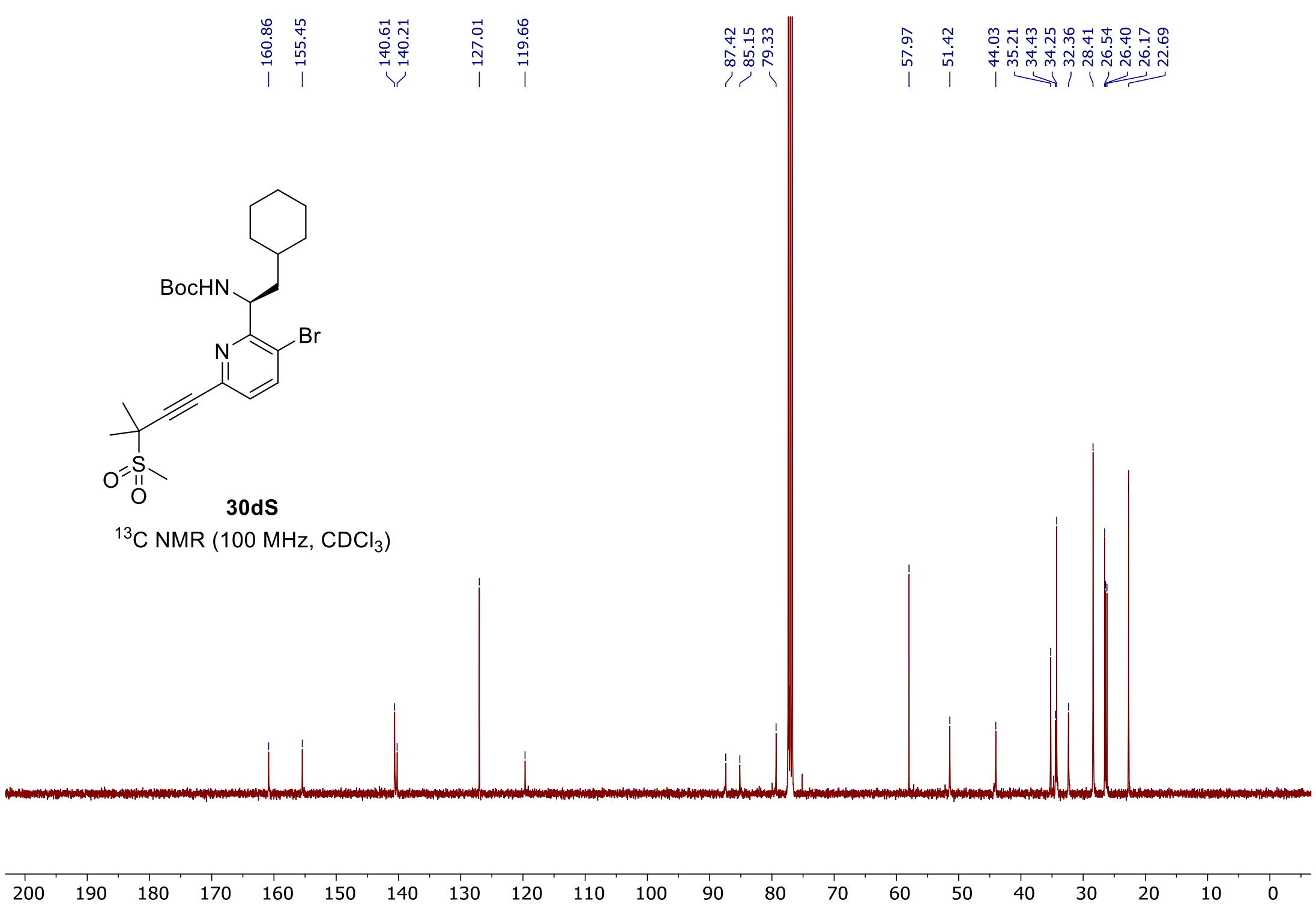

$^{1}H$ NMR (400 MHz, $CDCl_3$) of compound **30dR**

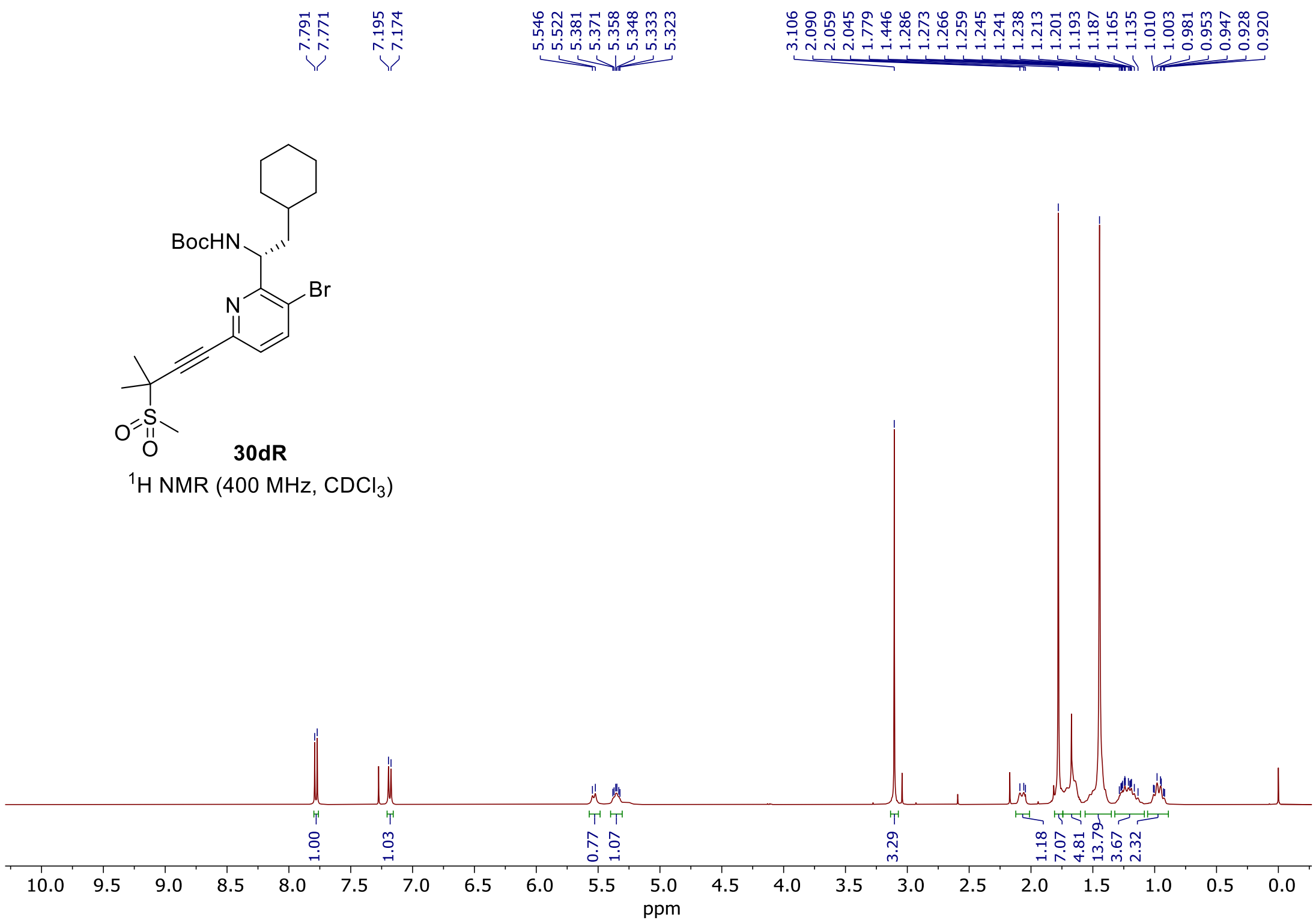


$^{13}C$ NMR (100 MHz, $CDCl_3$) of compound **30dR**

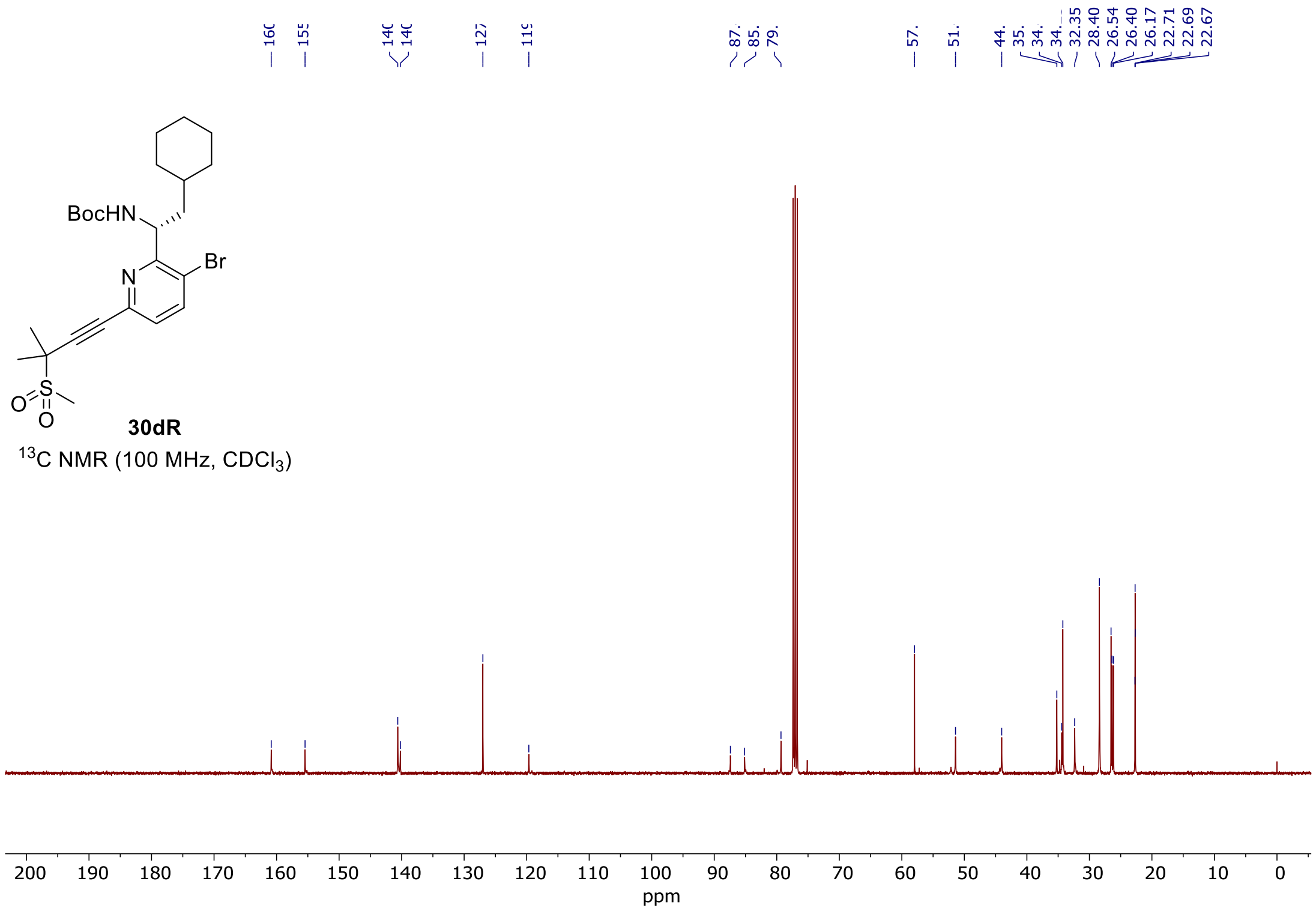

$^{1}$H NMR (400 MHz, $CDCl_3$) of compound **38aS**

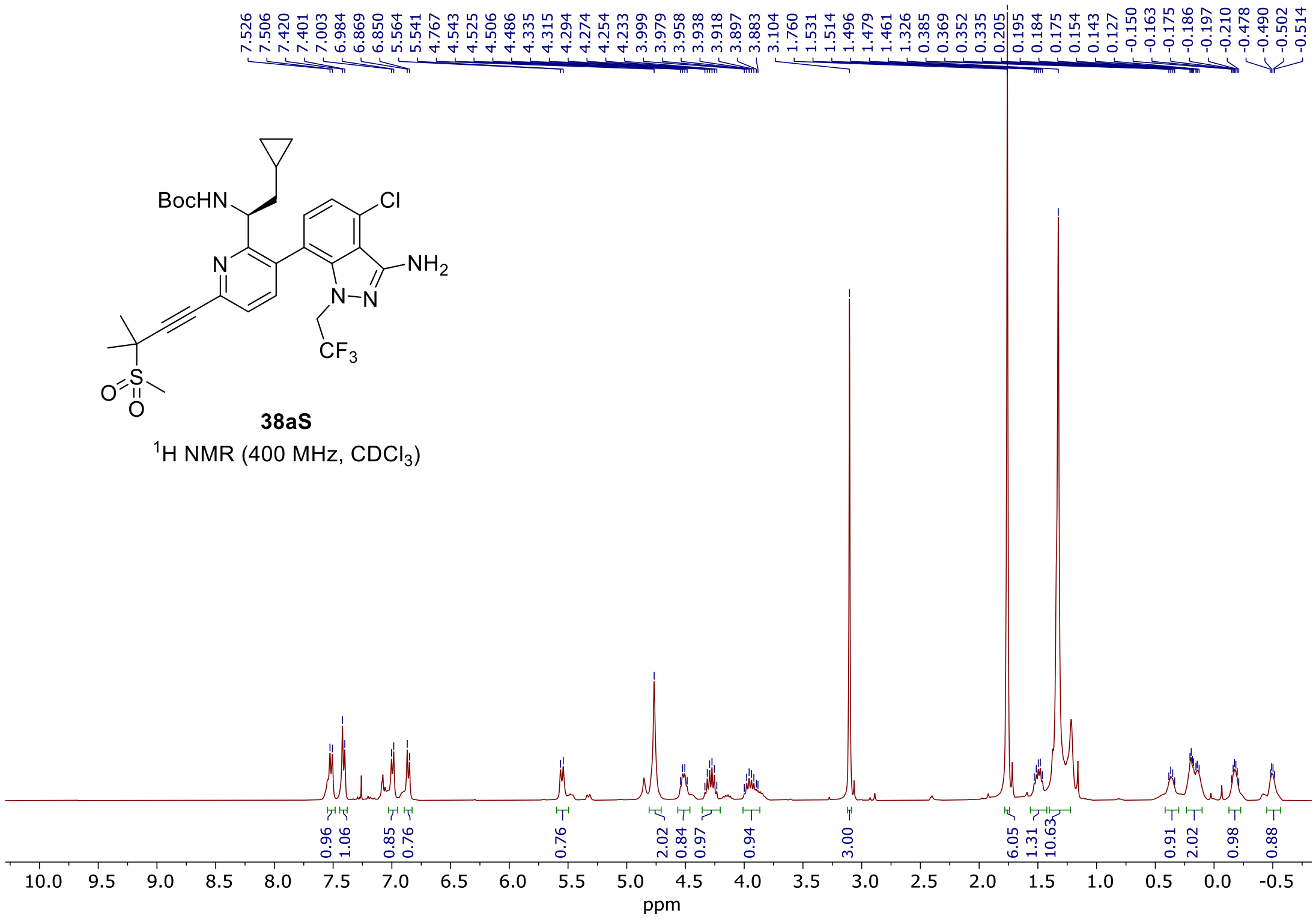


$^{13}$C NMR (100 MHz, $CDCl_3$) of compound **38aS**

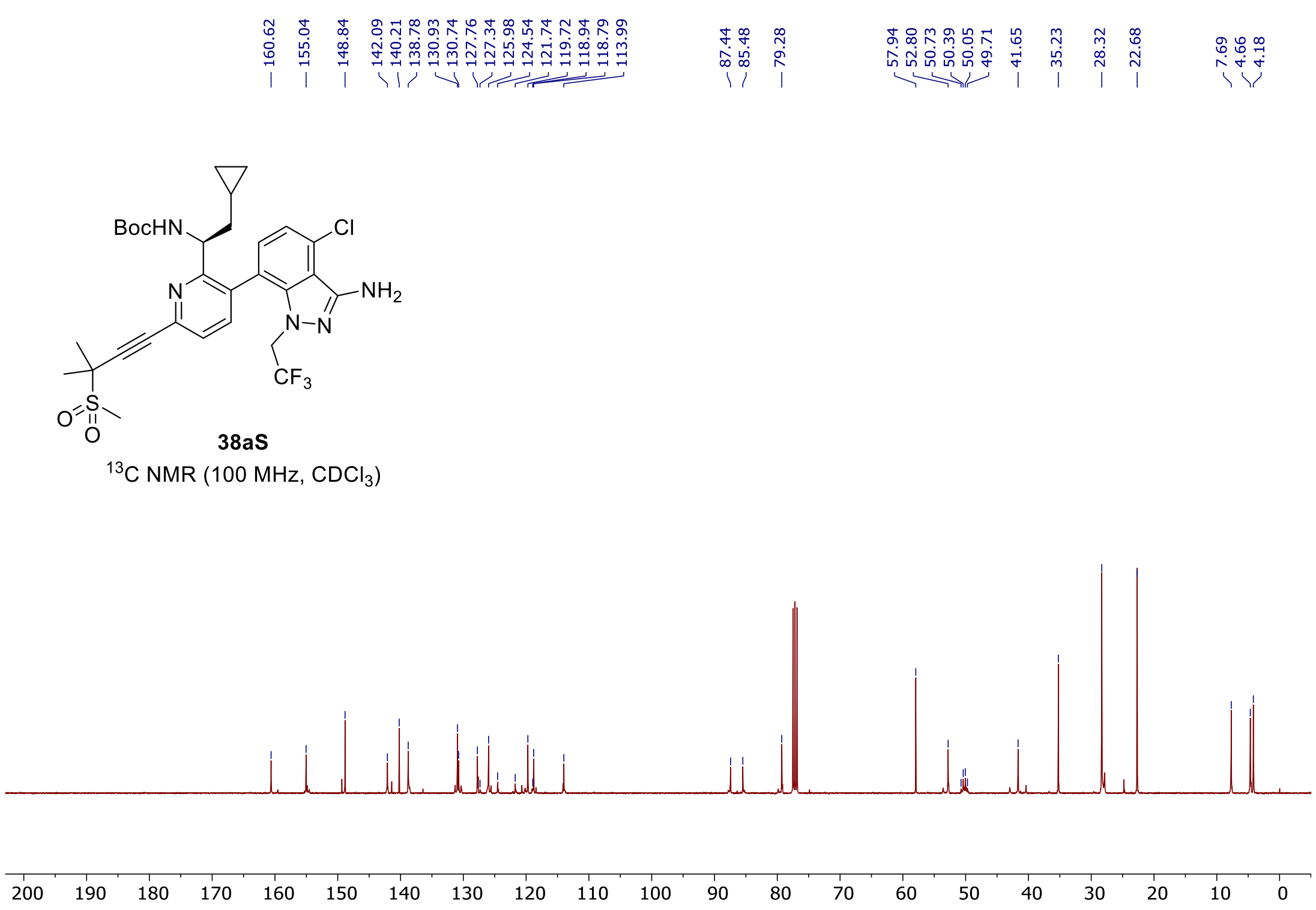

$^{19}$F NMR (376 MHz, $CDCl_3$) of compound **38aS**

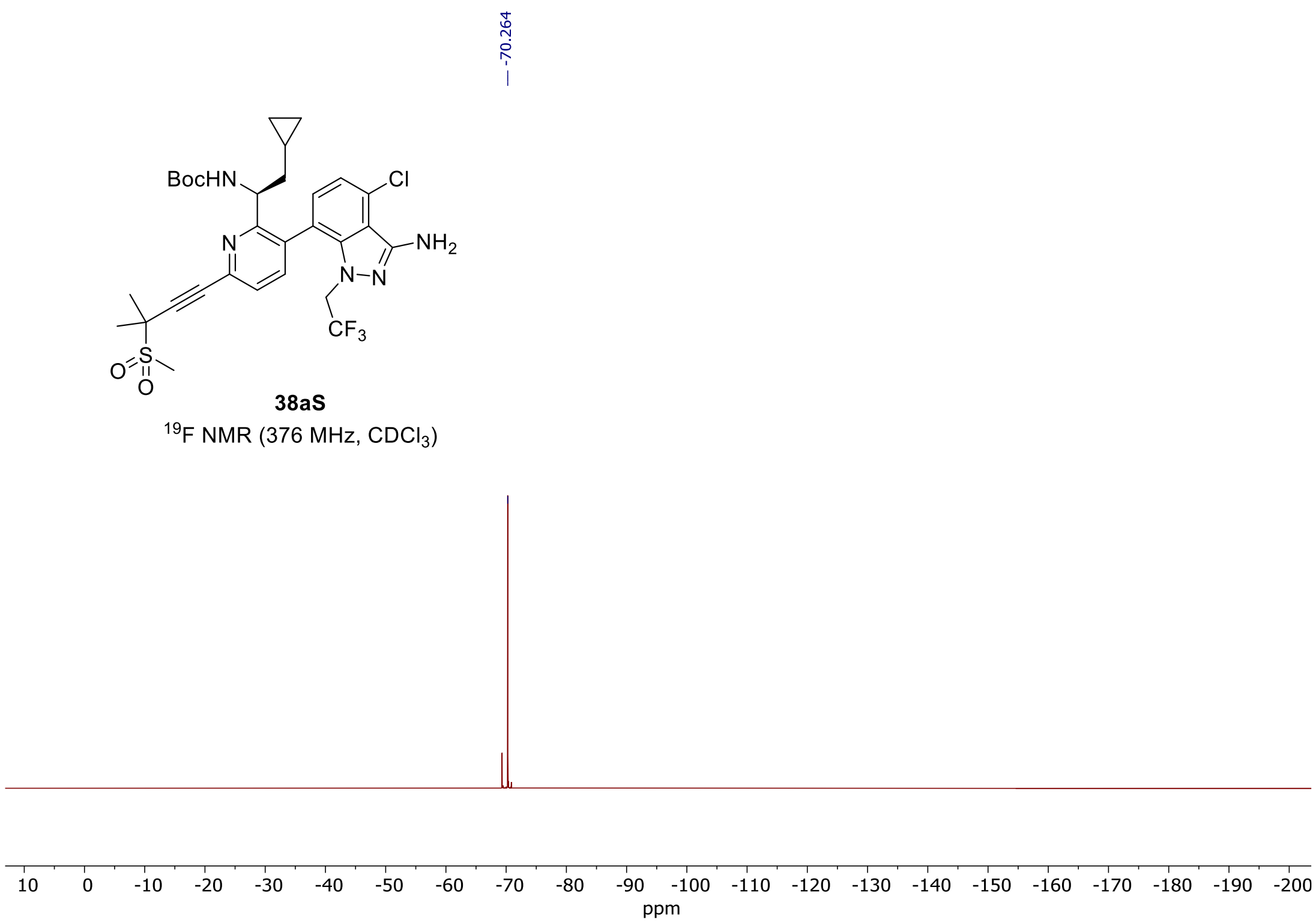

$^{1}$H NMR (400 MHz, $CDCl_3$) of compound **38aR**

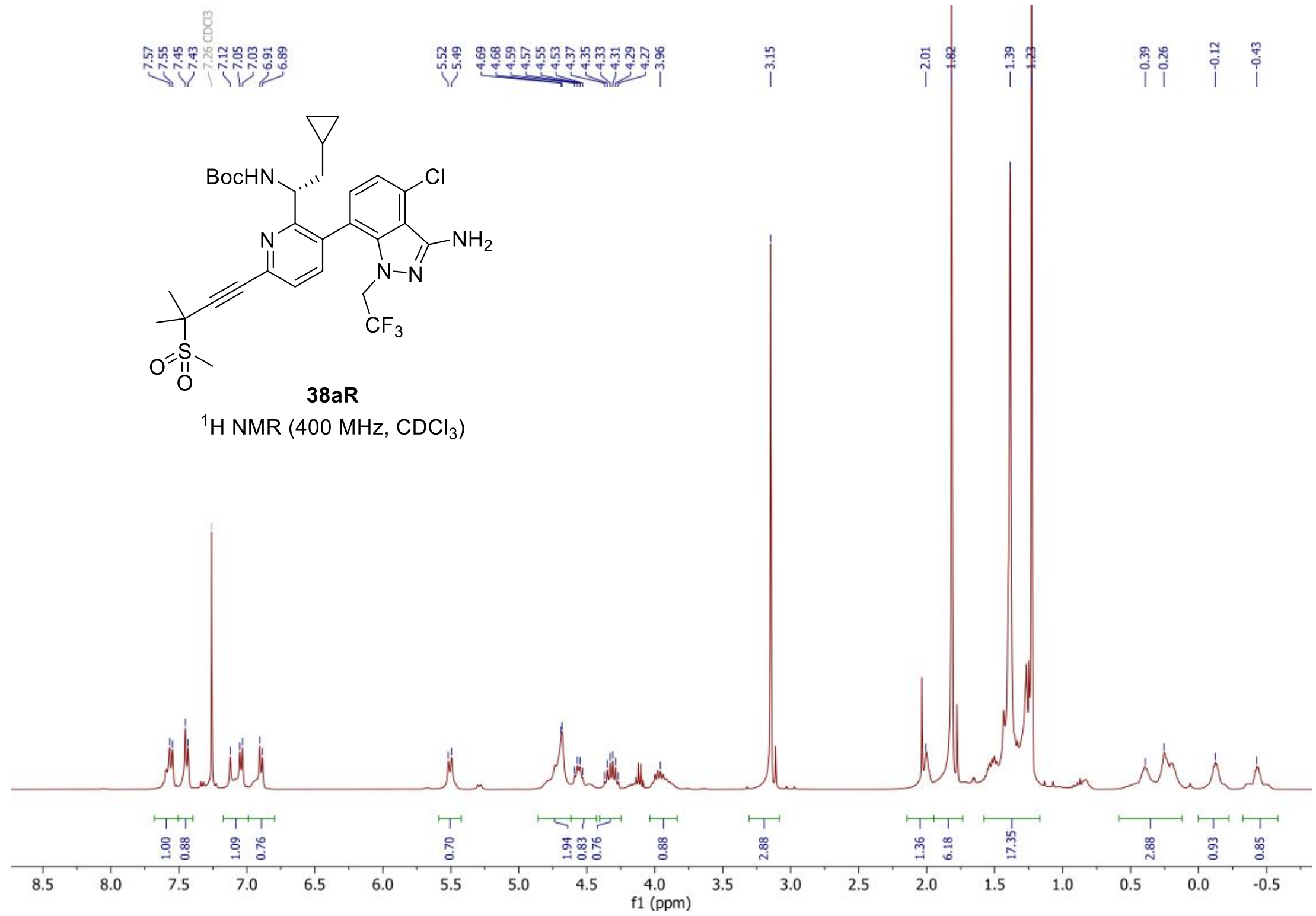


$^{13}$C NMR (100 MHz, $CDCl_3$) of compound **38aR**

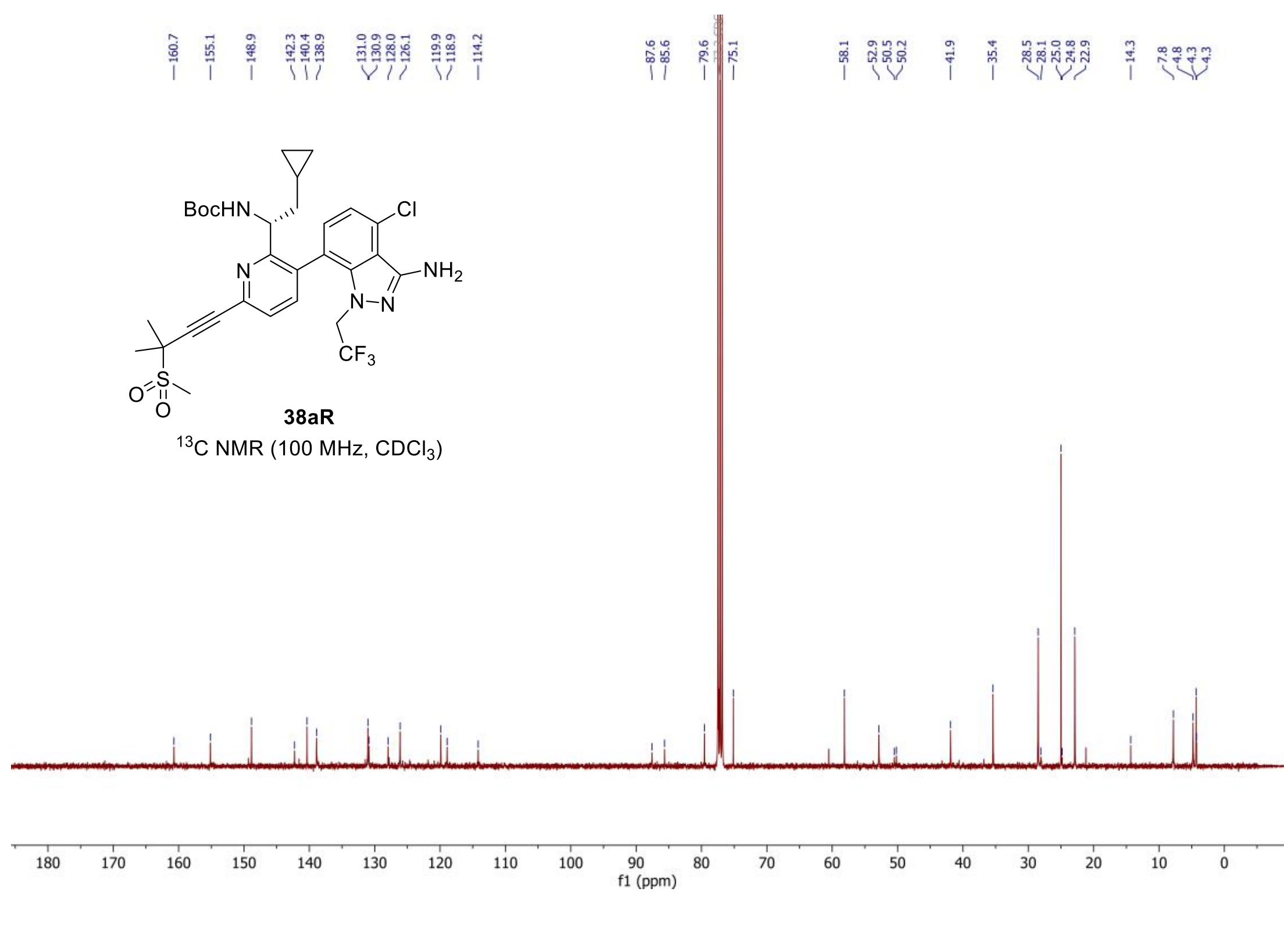

$^{1}H$ NMR (400 MHz, $CDCl_3$) of the compound **38bS**

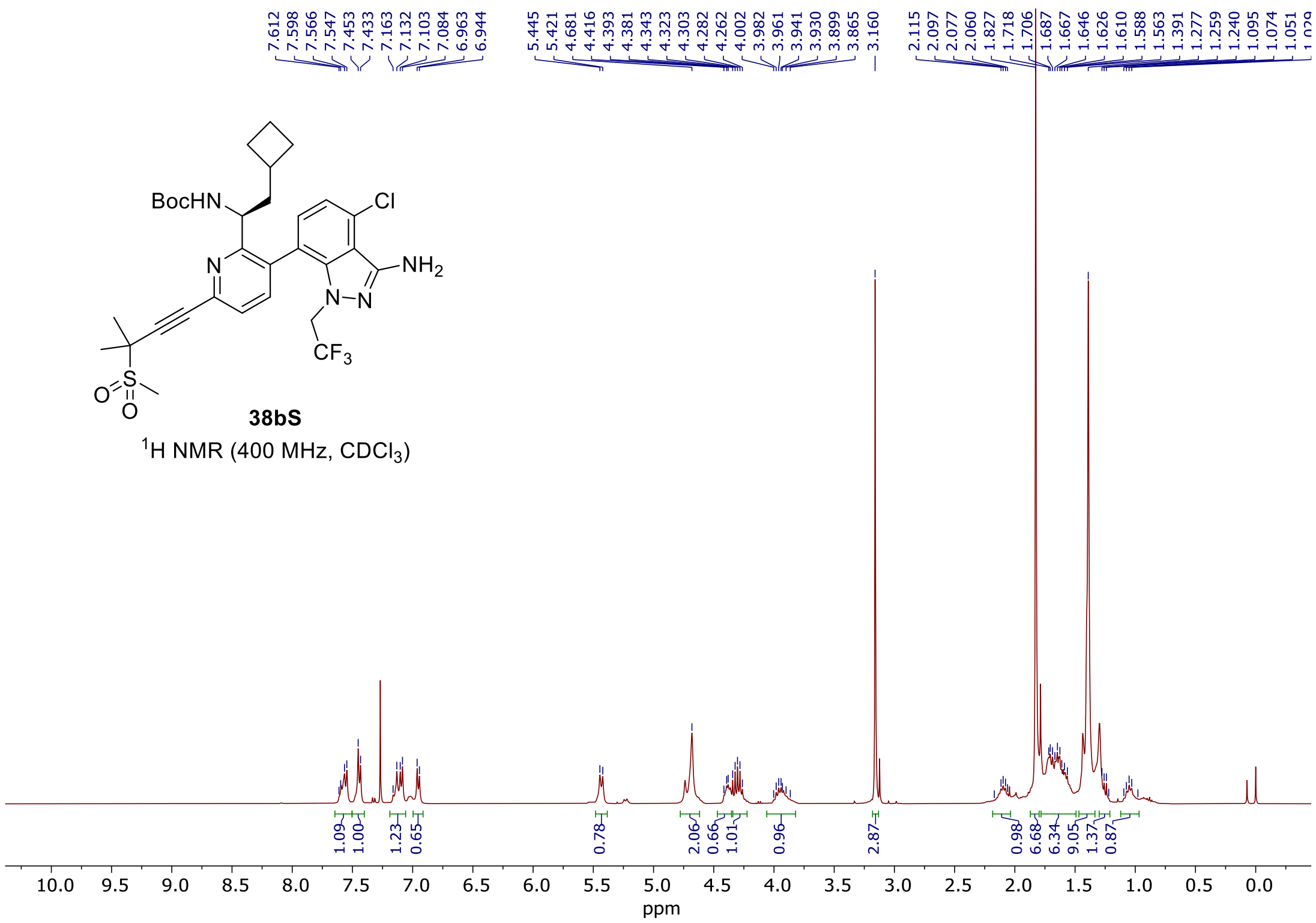


$^{13}C$ NMR (100 MHz, $CDCl_3$) of compound **38bS**

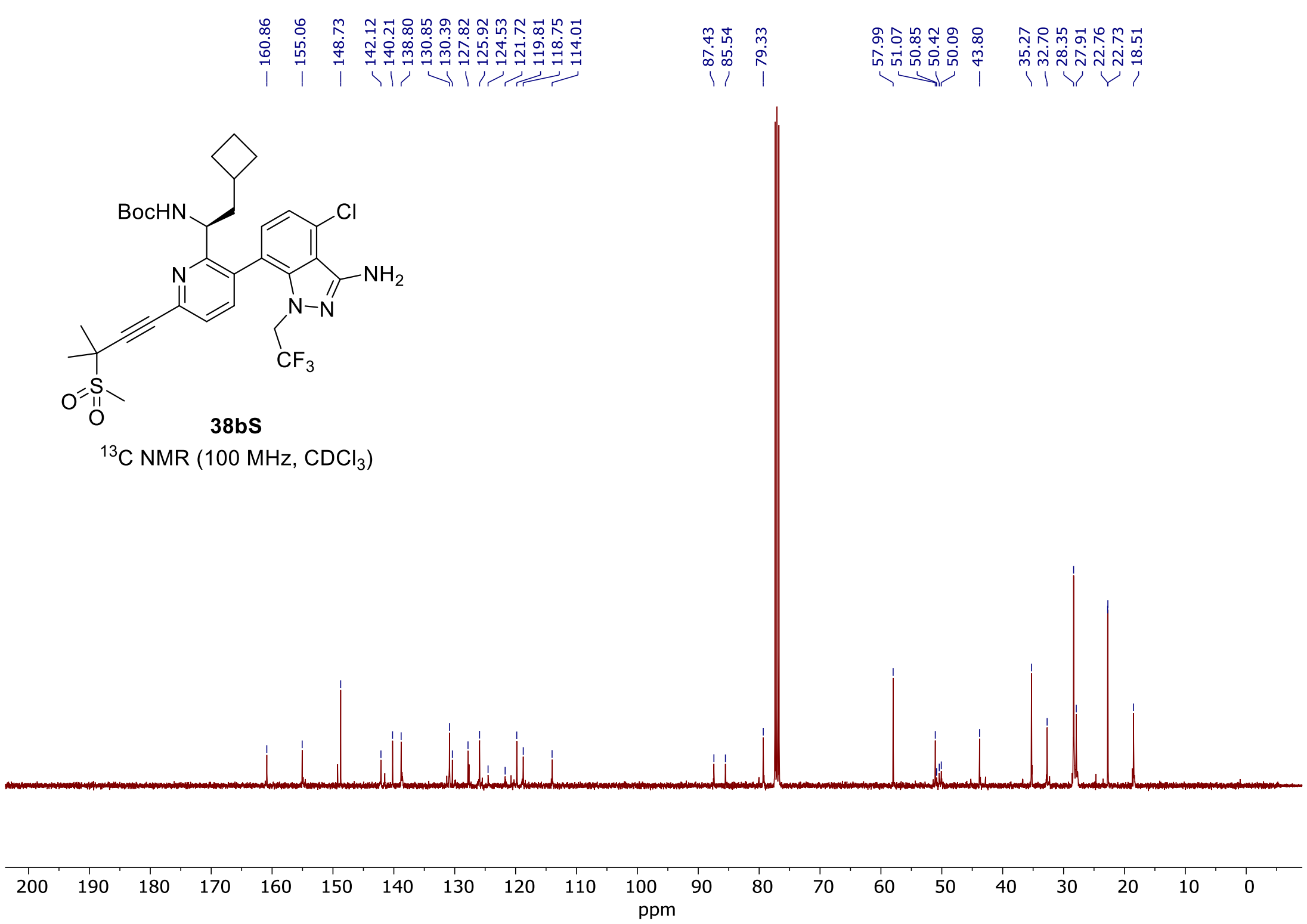

$^{19}$F NMR (376 MHz, $CDCl_3$) of compound **38bS**

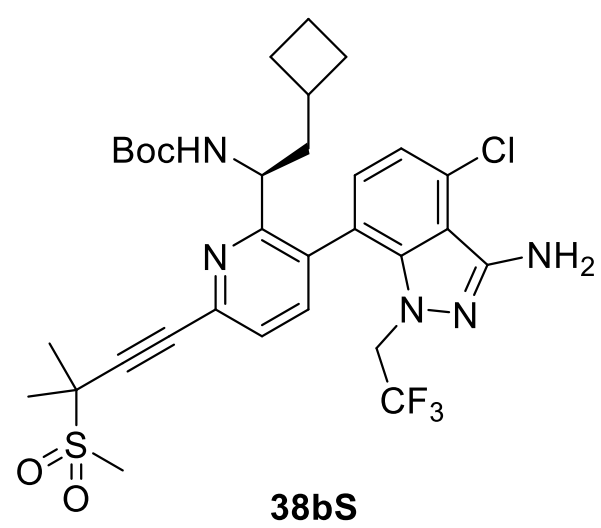

38bS

$^{19}$F NMR (376 MHz, $CDCl_3$)

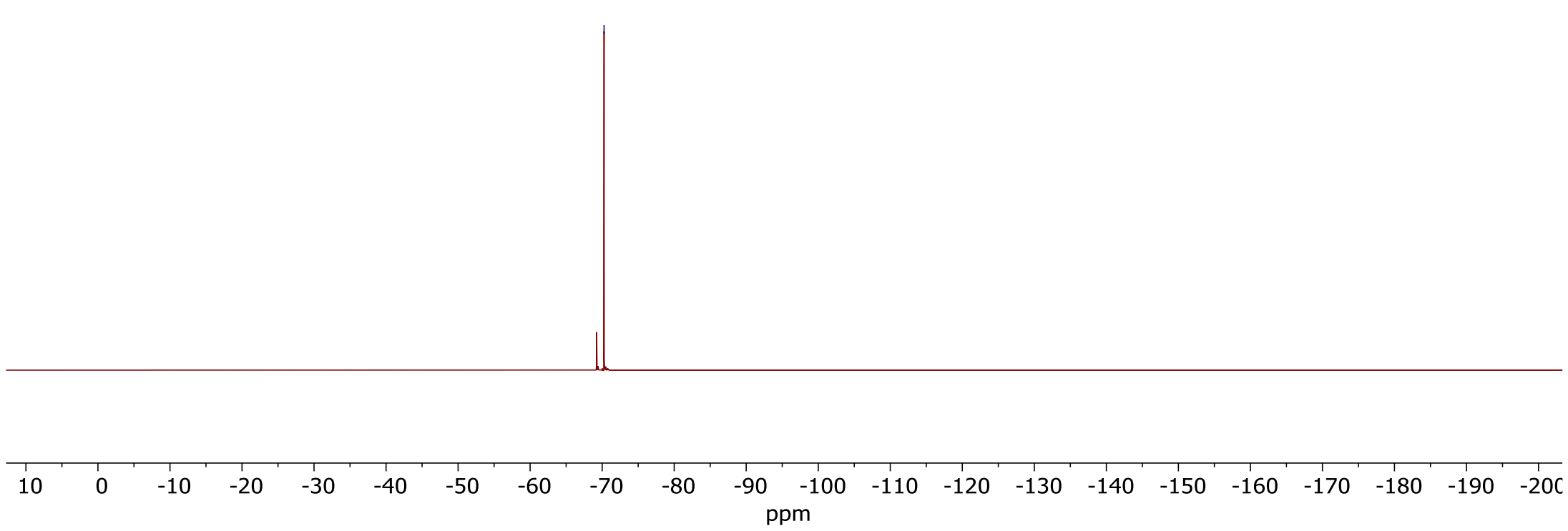

$^{1}H$ NMR (400 MHz, $CDCl_3$) of the compound **38bR**

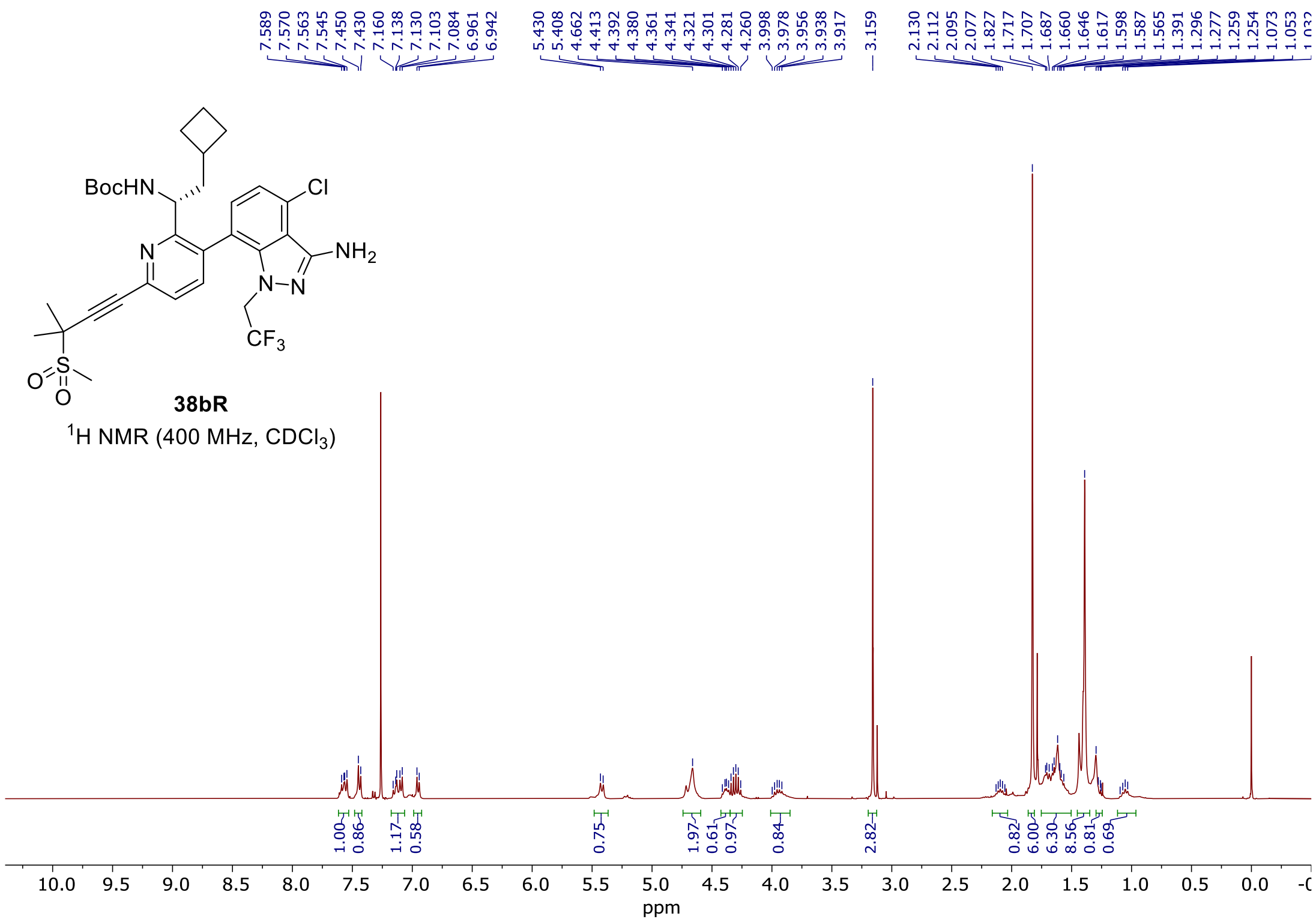


$^{13}C$ NMR (100 MHz, $CDCl_3$) of compound **38bR**

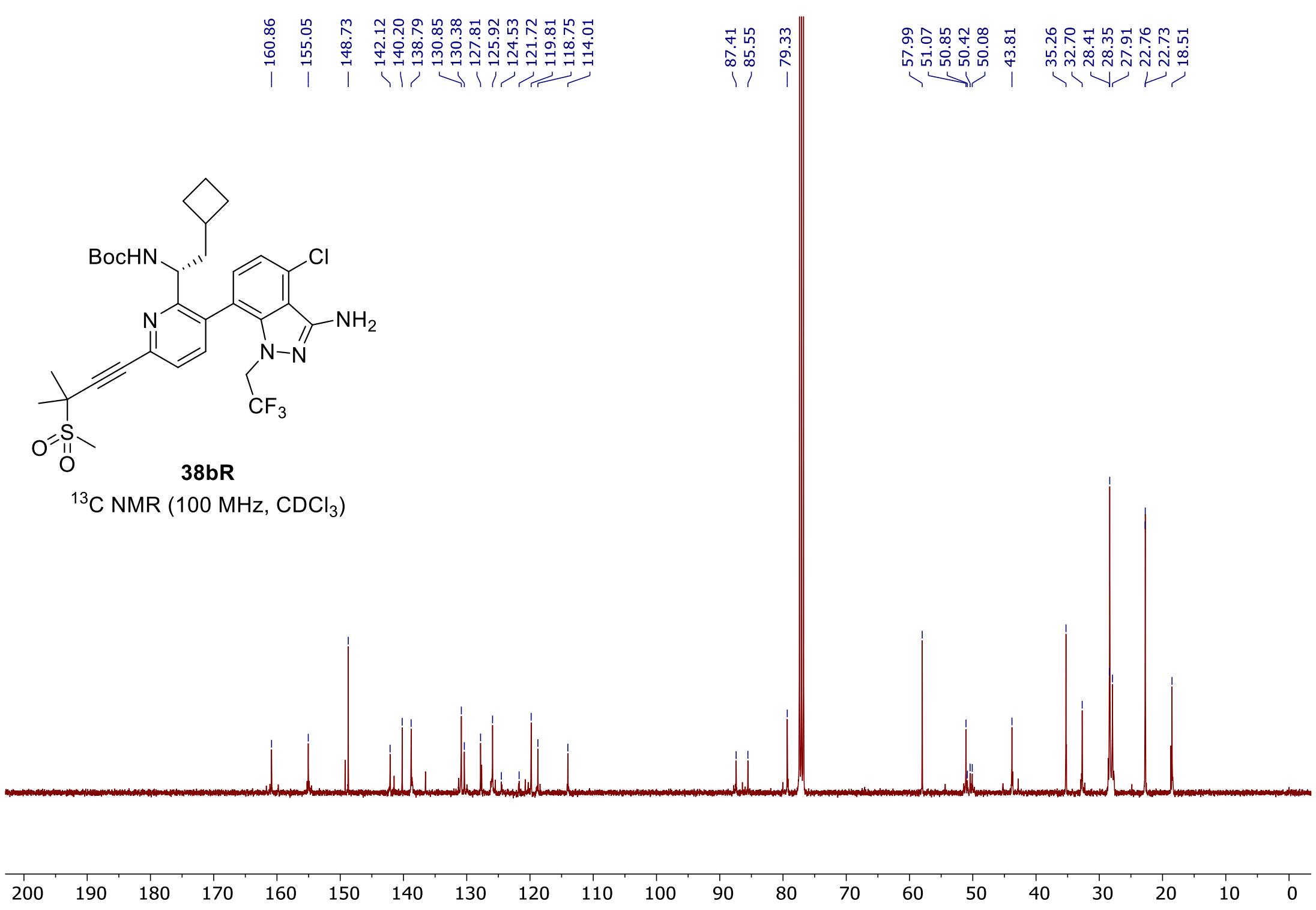

$^{19}$F NMR (376 MHz, $CDCl_3$) of compound **38bR**

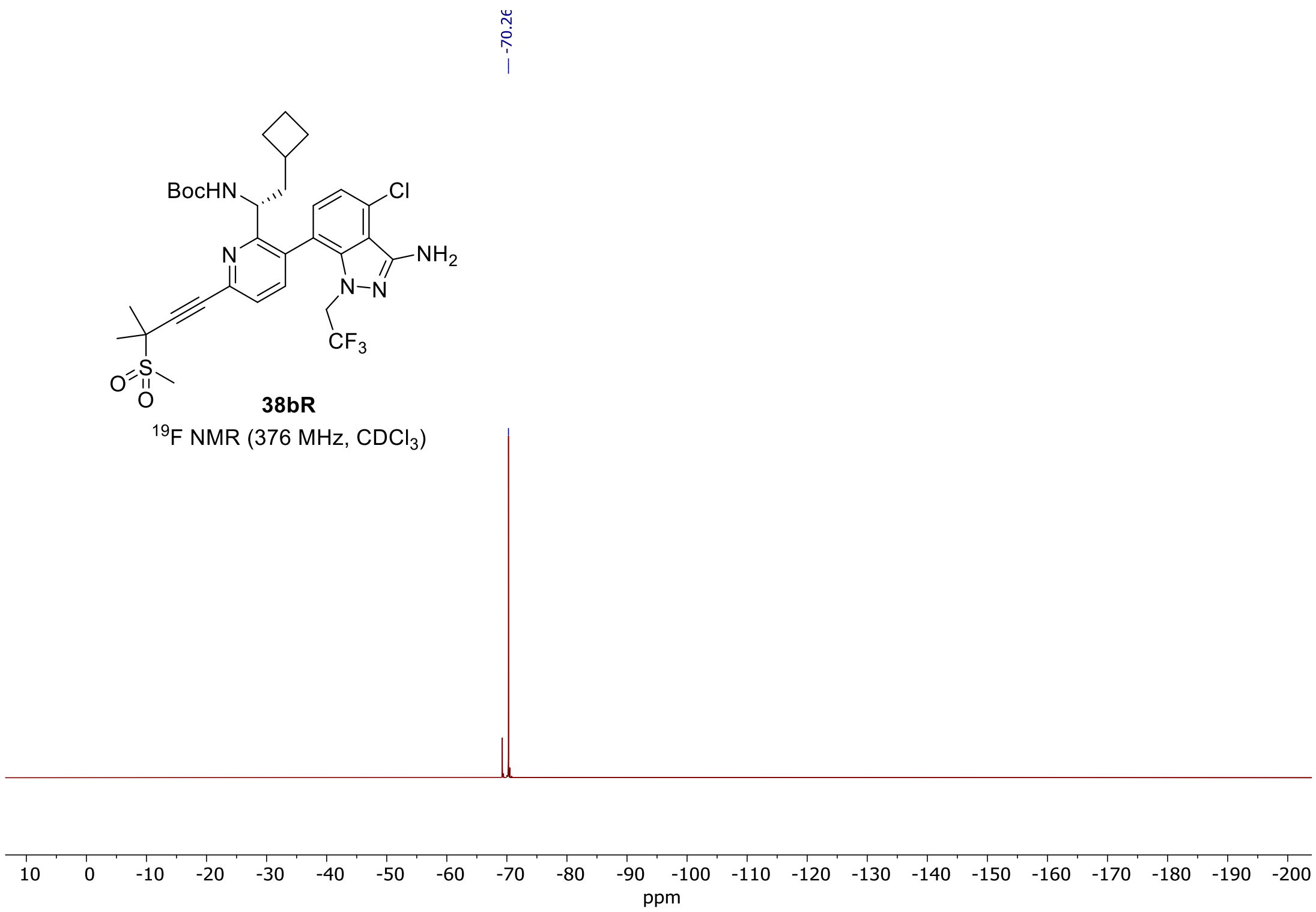


**38bR**

$^{19}$F NMR (376 MHz, $CDCl_3$)

$^{1}H$ NMR (400 MHz, $CDCl_3$) of the compound **38cS**

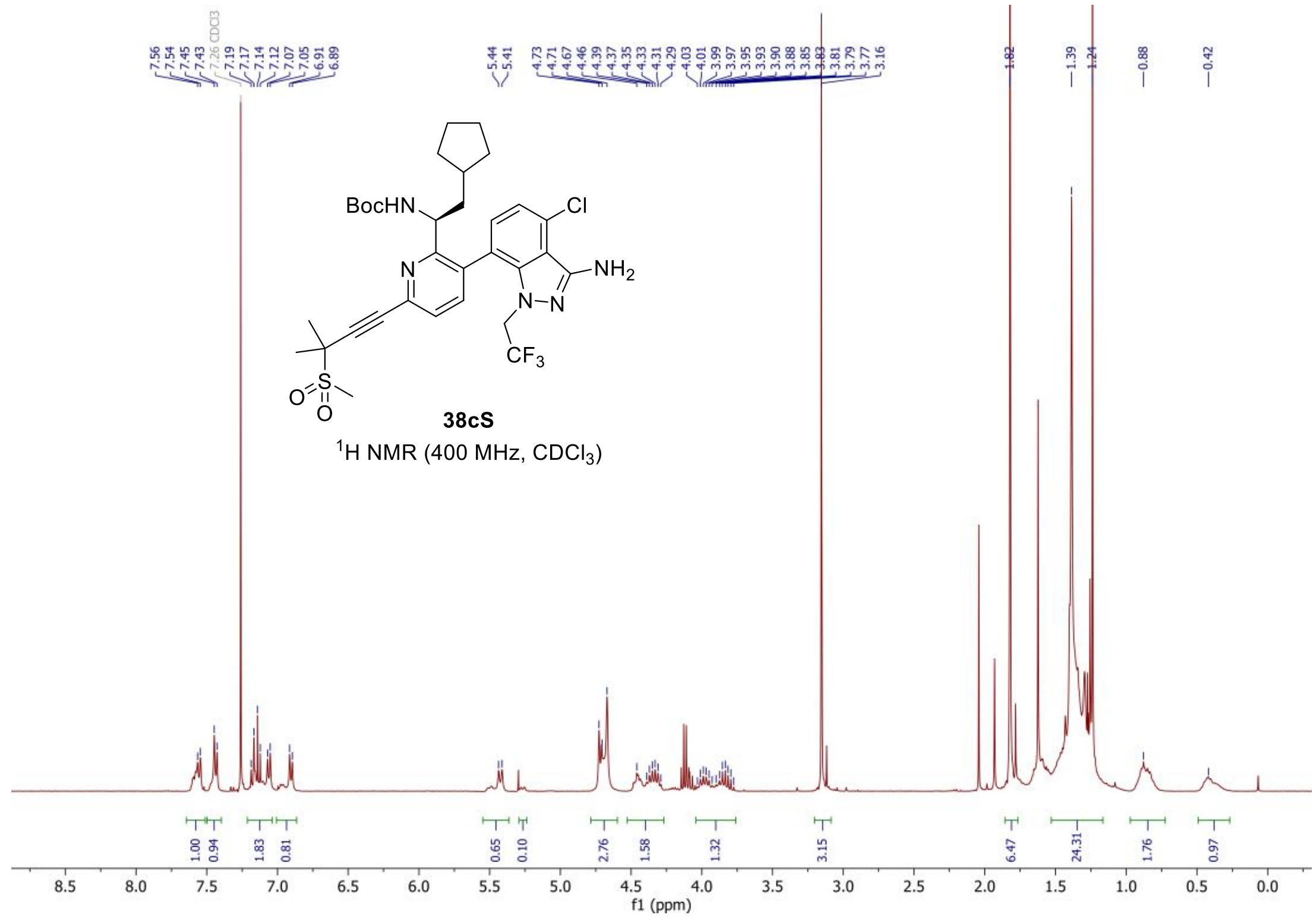


$^{13}C$ NMR (100 MHz, $CDCl_3$) of compound **38cS**

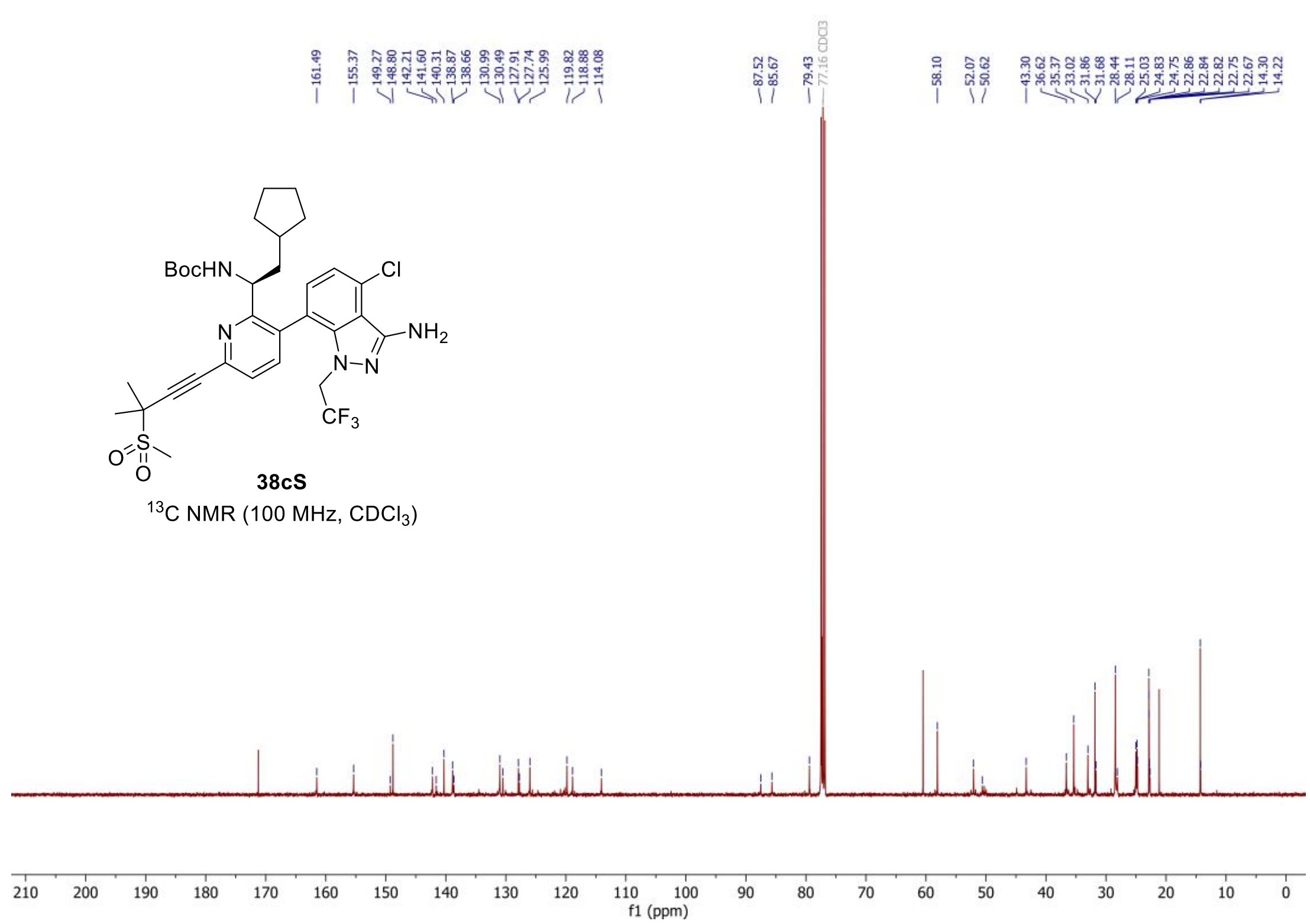

$^{1}$H NMR (400 MHz, CDCl$_3$) of compound **38cR**

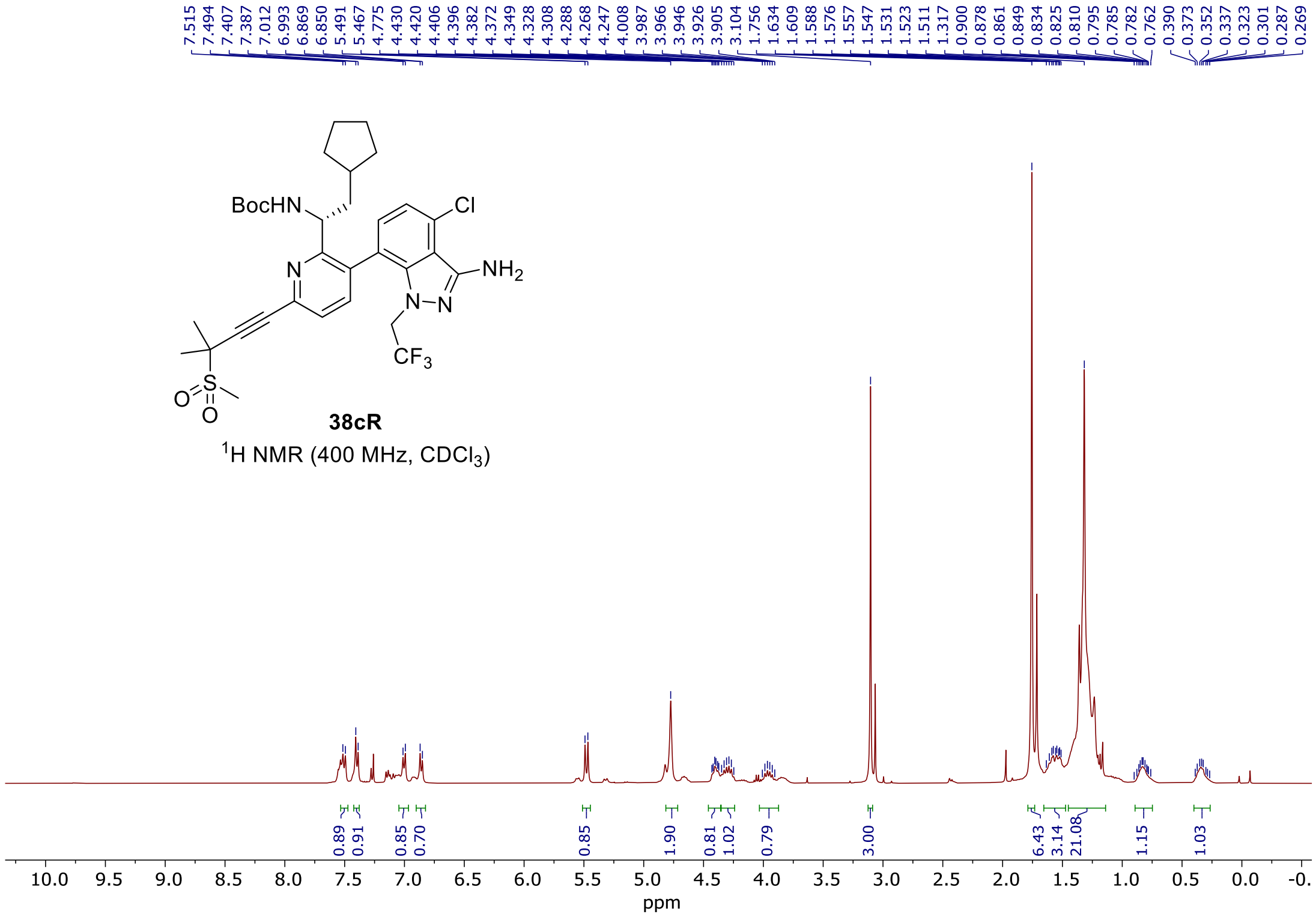


$^{13}$C NMR (100 MHz, CDCl$_3$) of compound **38cR**

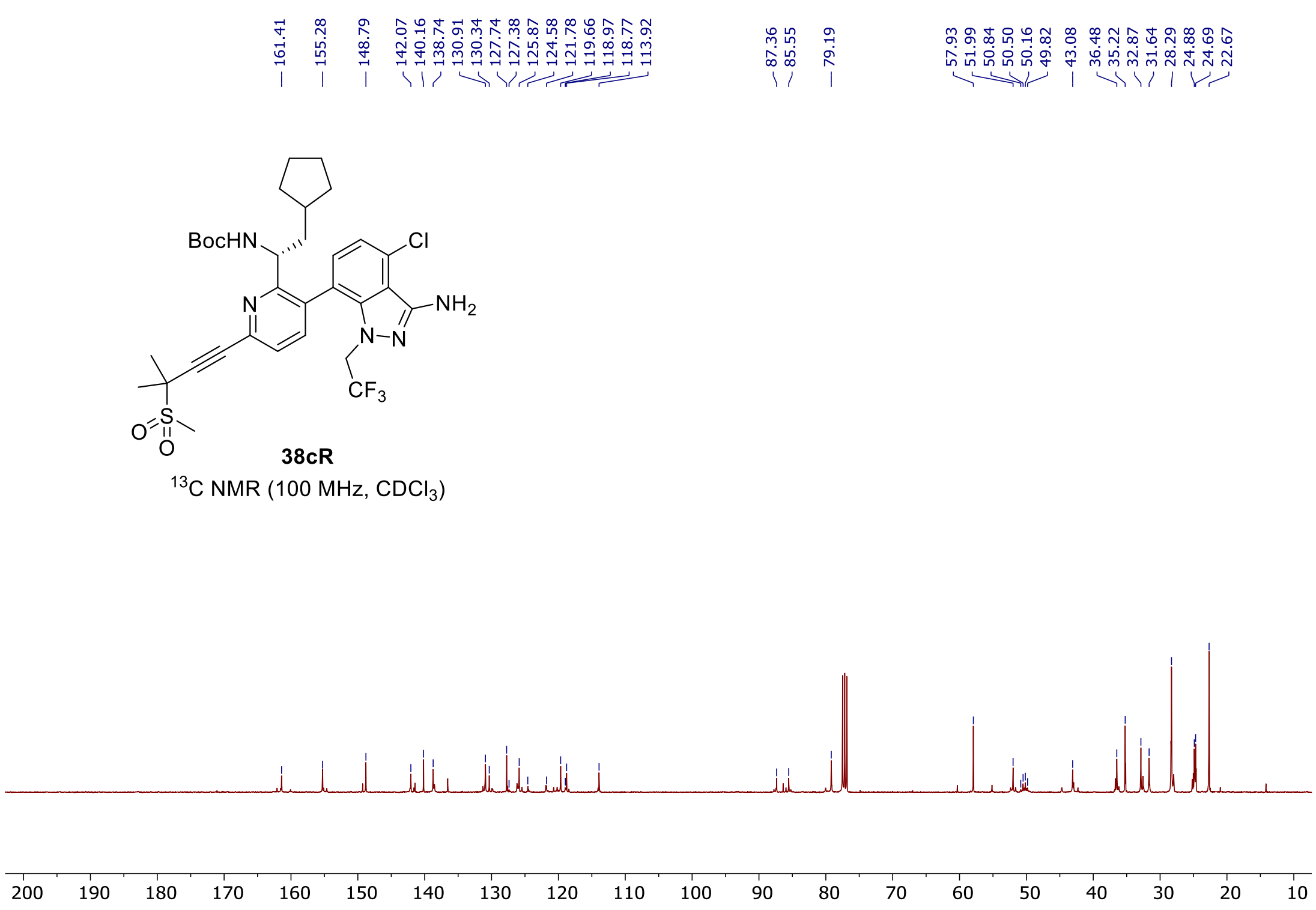

$^{19}$F NMR (376 MHz, $CDCl_3$) of compound **38cR**

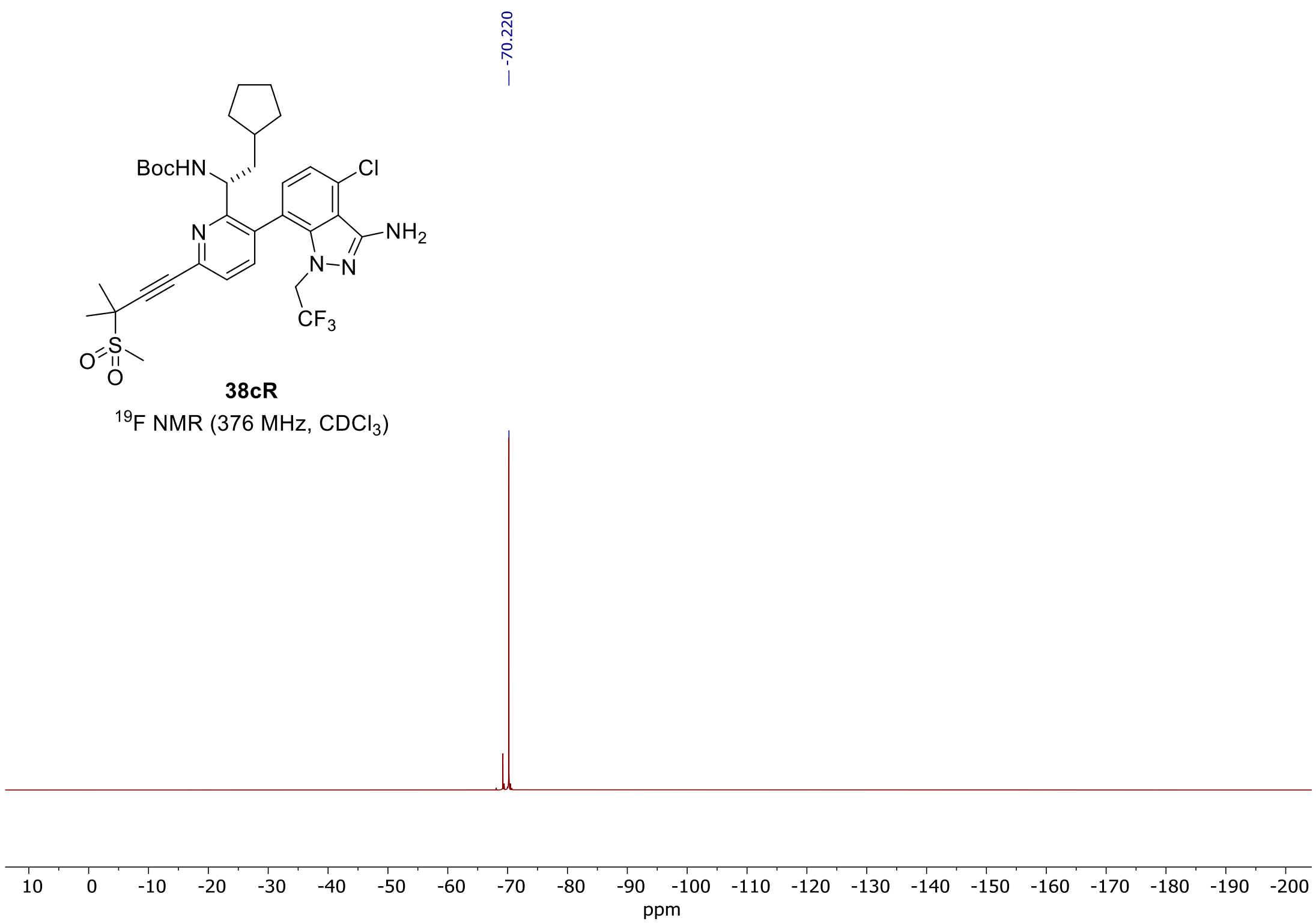

$^{1}H$ NMR (400 MHz, $CDCl_3$) of compound **38dS**

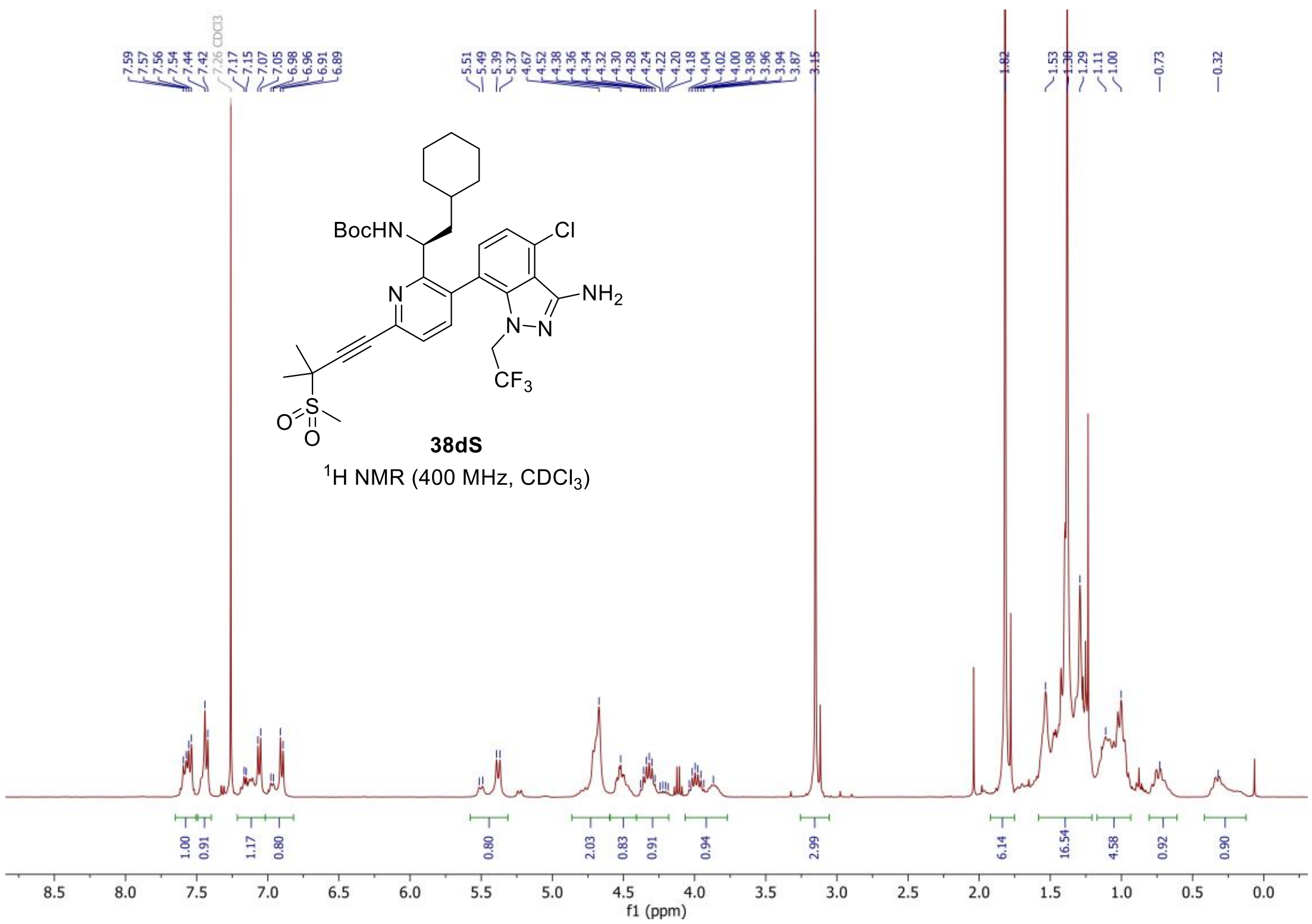


$^{13}C$ NMR (100 MHz, $CDCl_3$) of compound **38dS**

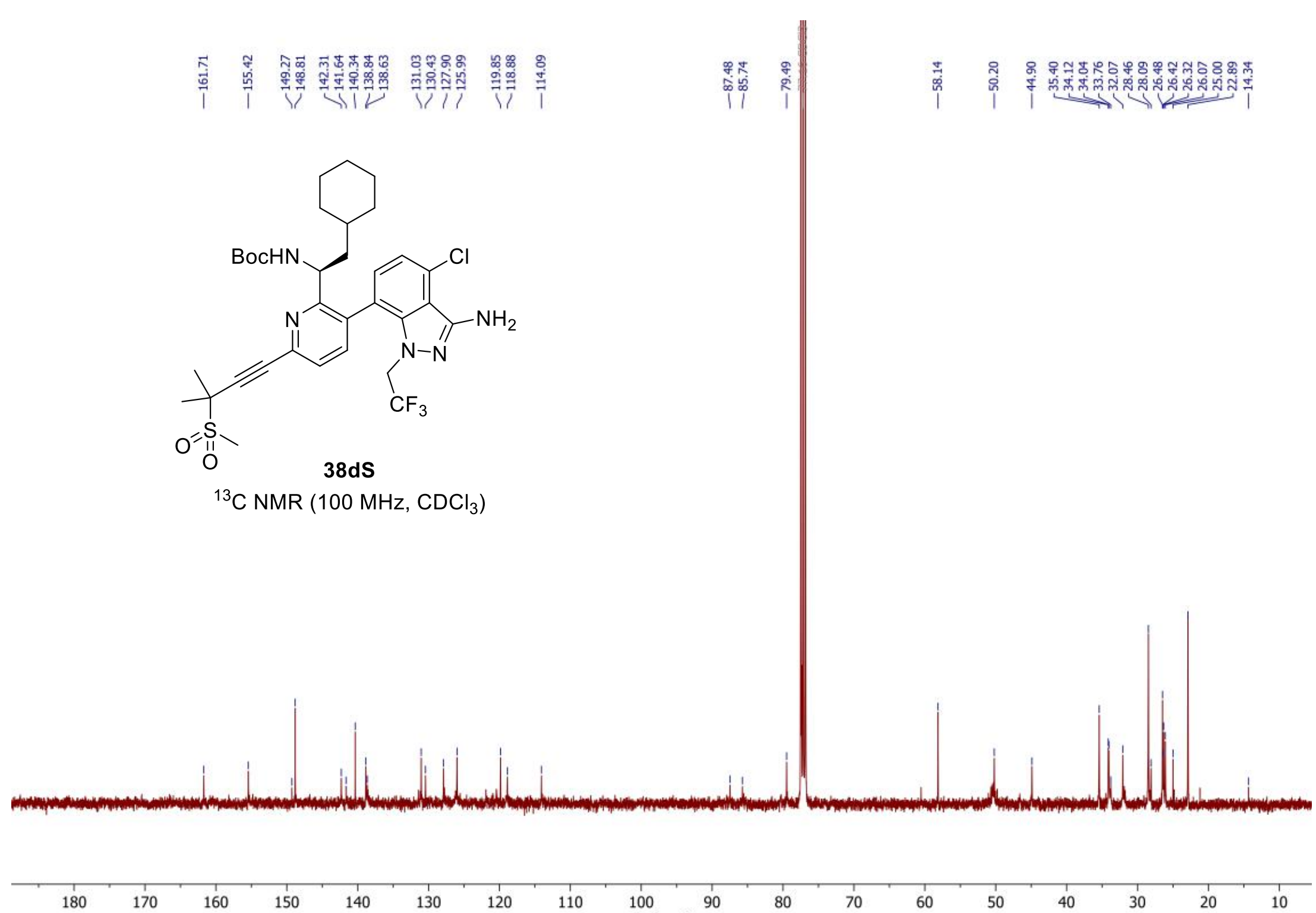

$^{1}H$ NMR (400 MHz, $CDCl_3$) of compound **38dR**

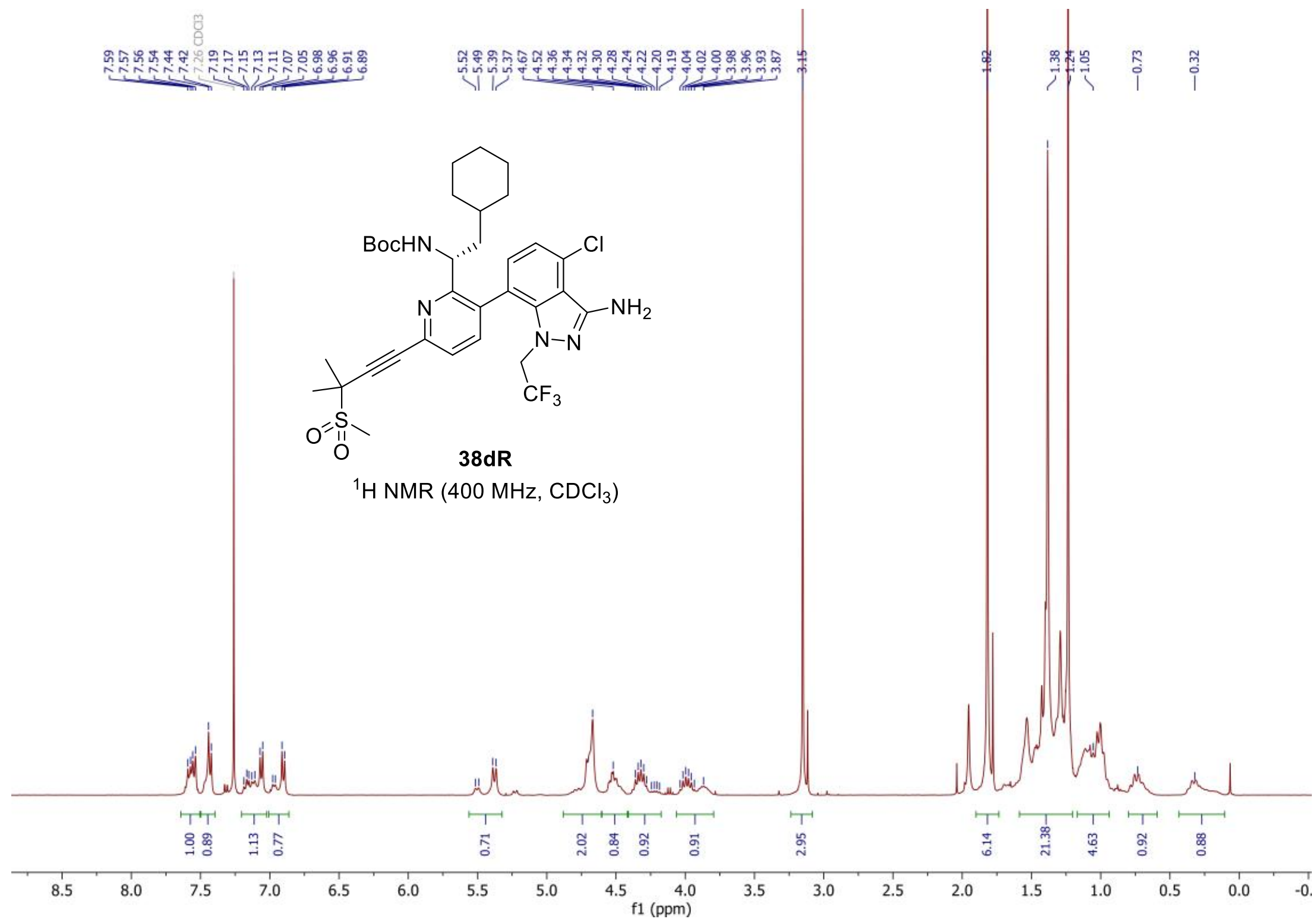

$^{13}C$ NMR (100 MHz, $CDCl_3$) of compound **38dR**

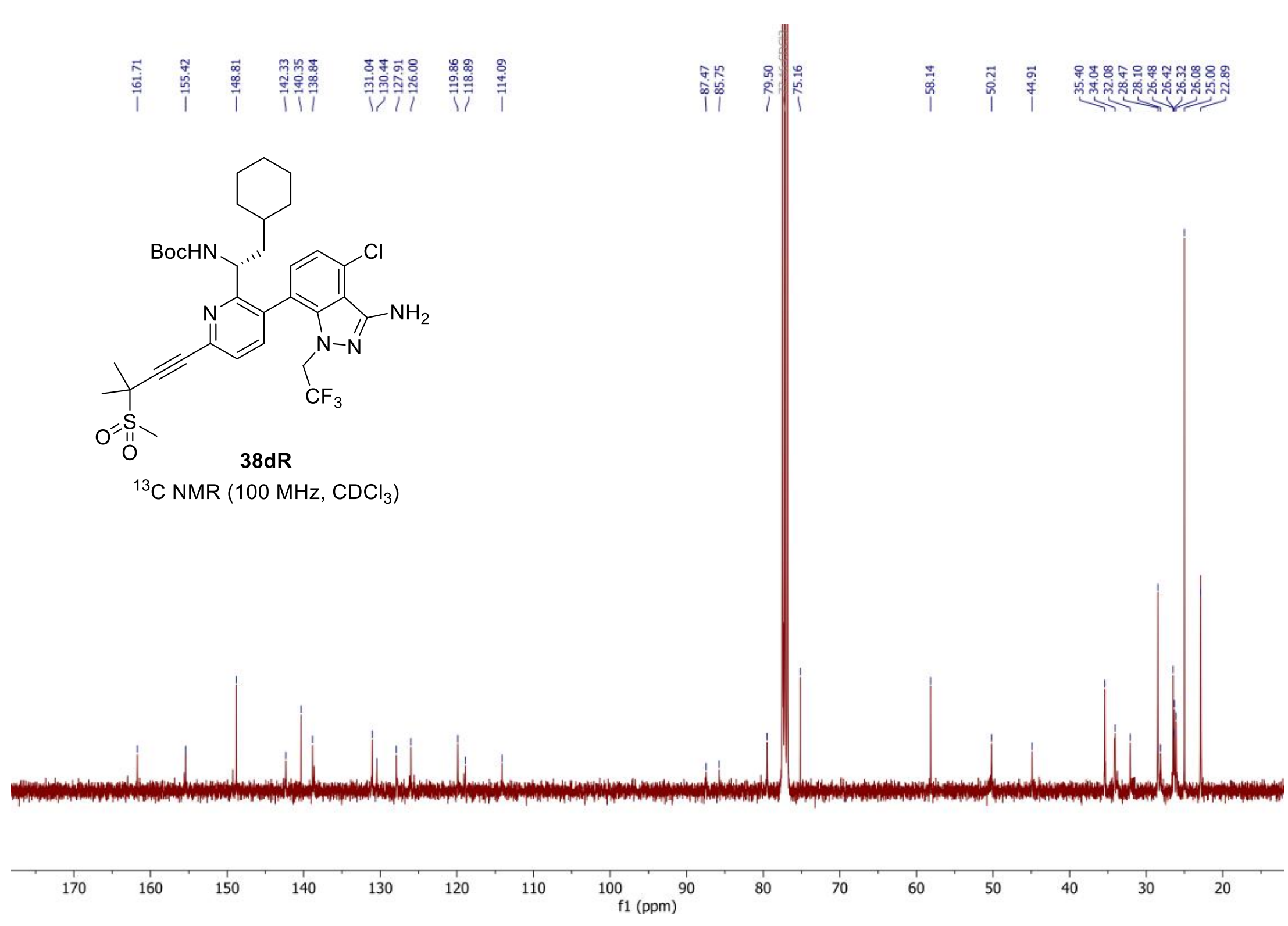

$^{1}$H NMR (400 MHz, $CDCl_3$) of compound **39aS**

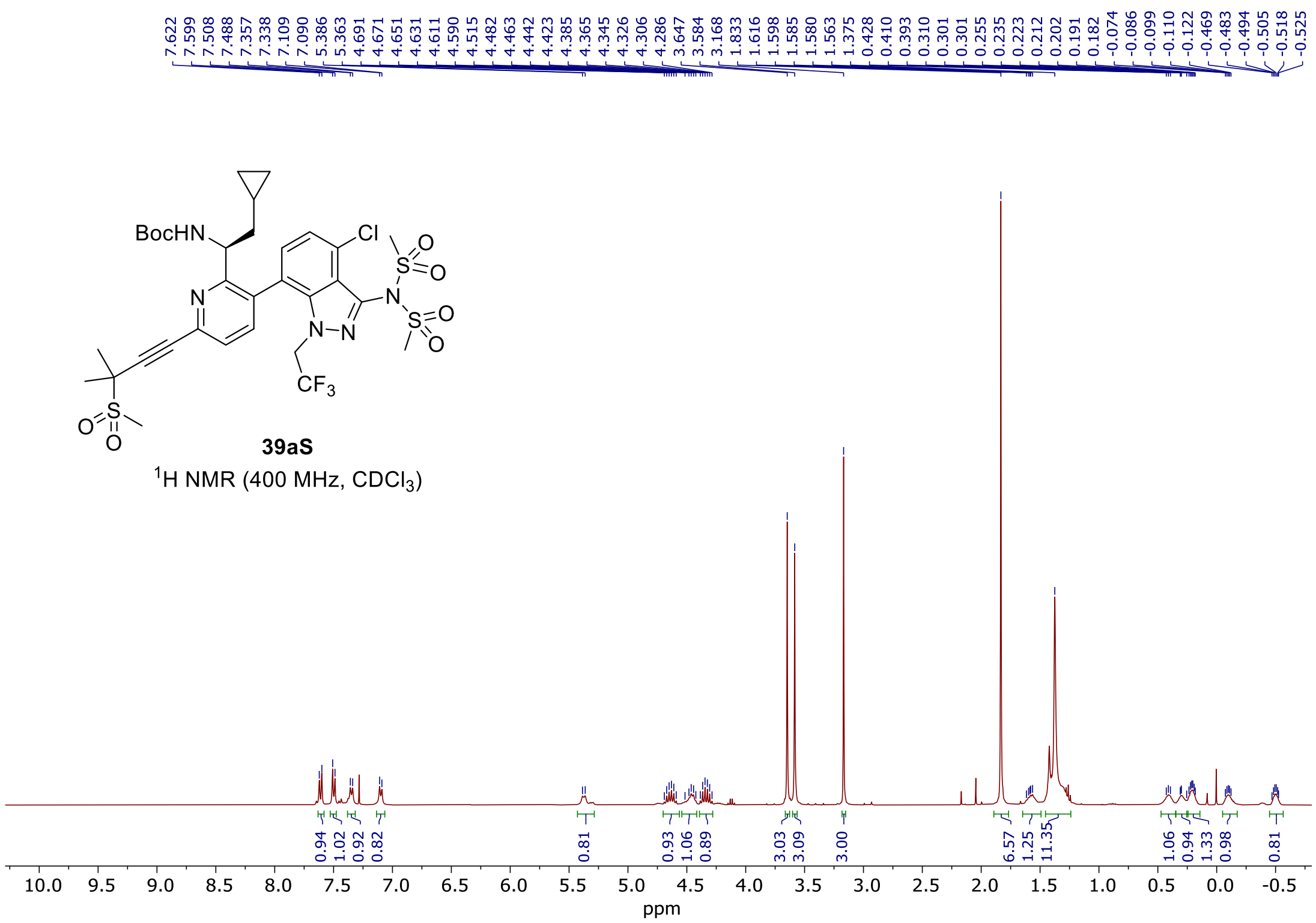


$^{13}$C NMR (100 MHz, $CDCl_3$) of compound **39aS**

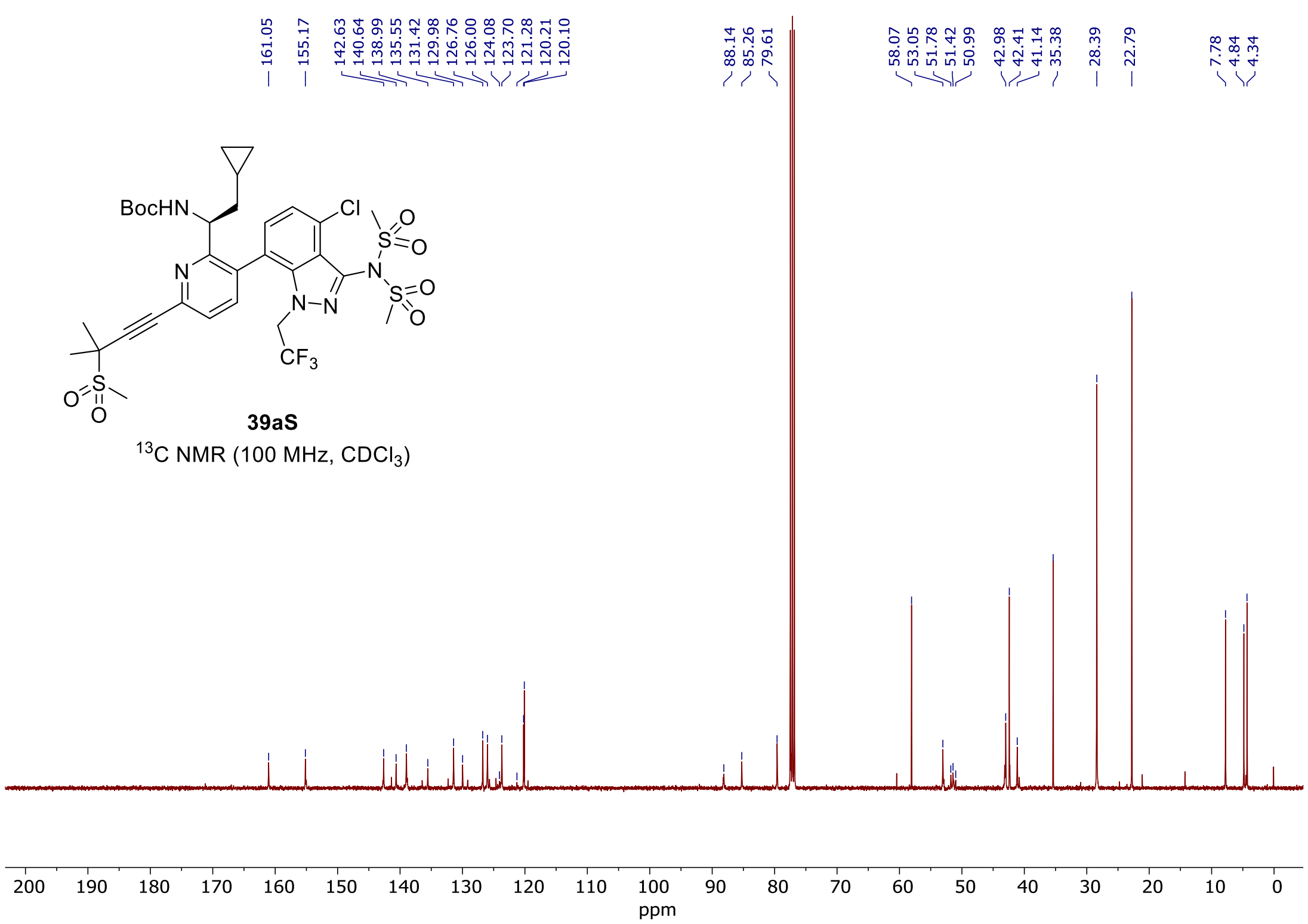

$^{19}F$ NMR (376 MHz, $CDCl_3$) of compound **39aS**

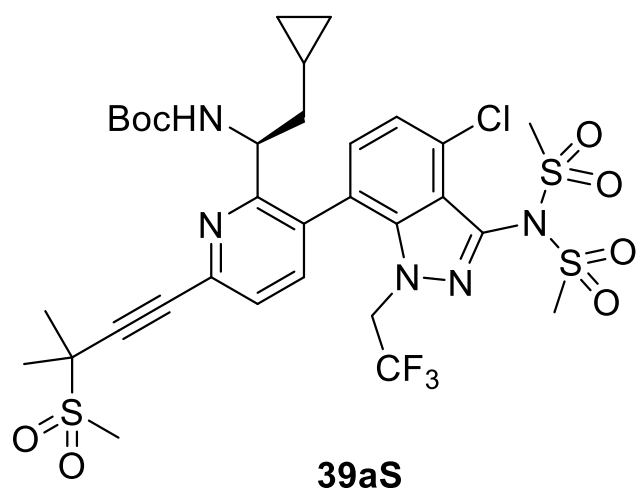

**39aS**

$^{19}F$ NMR (376 MHz, $CDCl_3$)

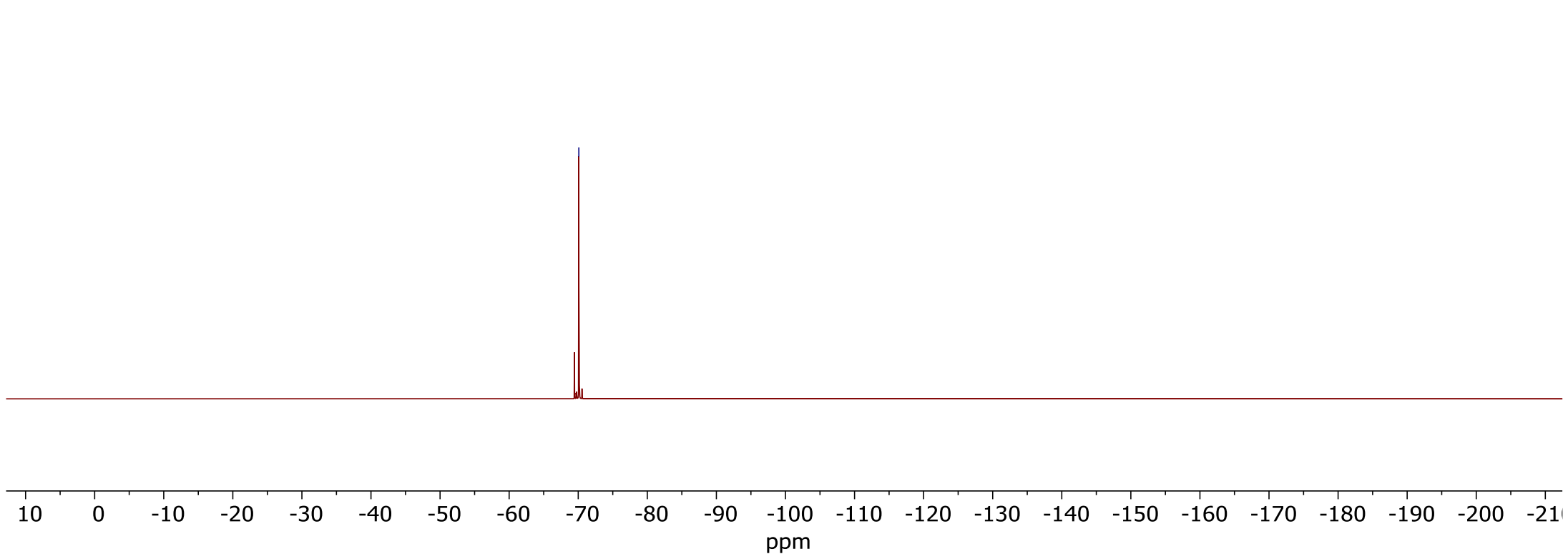

$^{1}H$ NMR (400 MHz, $CDCl_3$) of compound **39aR**

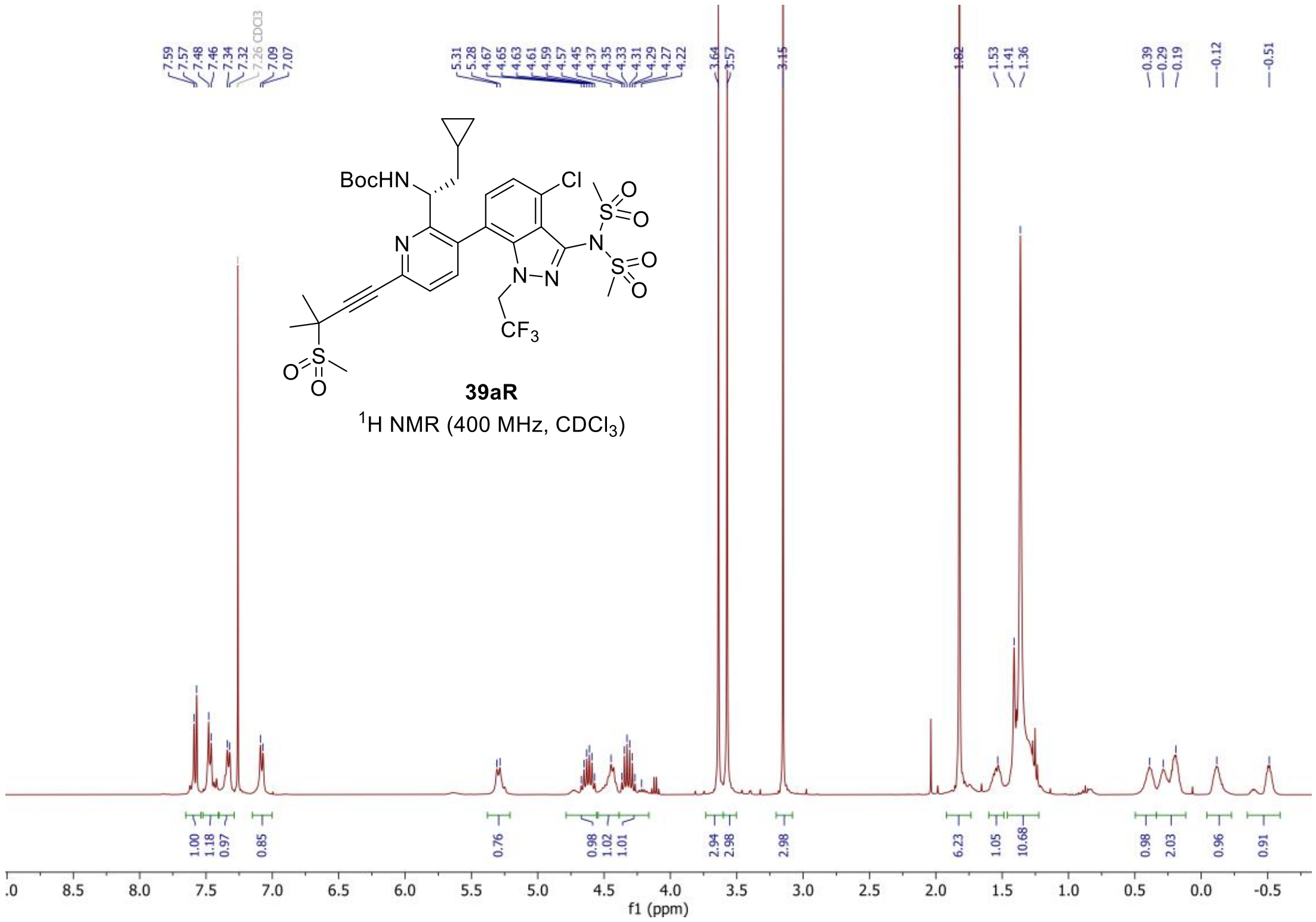


$^{13}C$ NMR (100 MHz, $CDCl_3$) of compound **39aR**

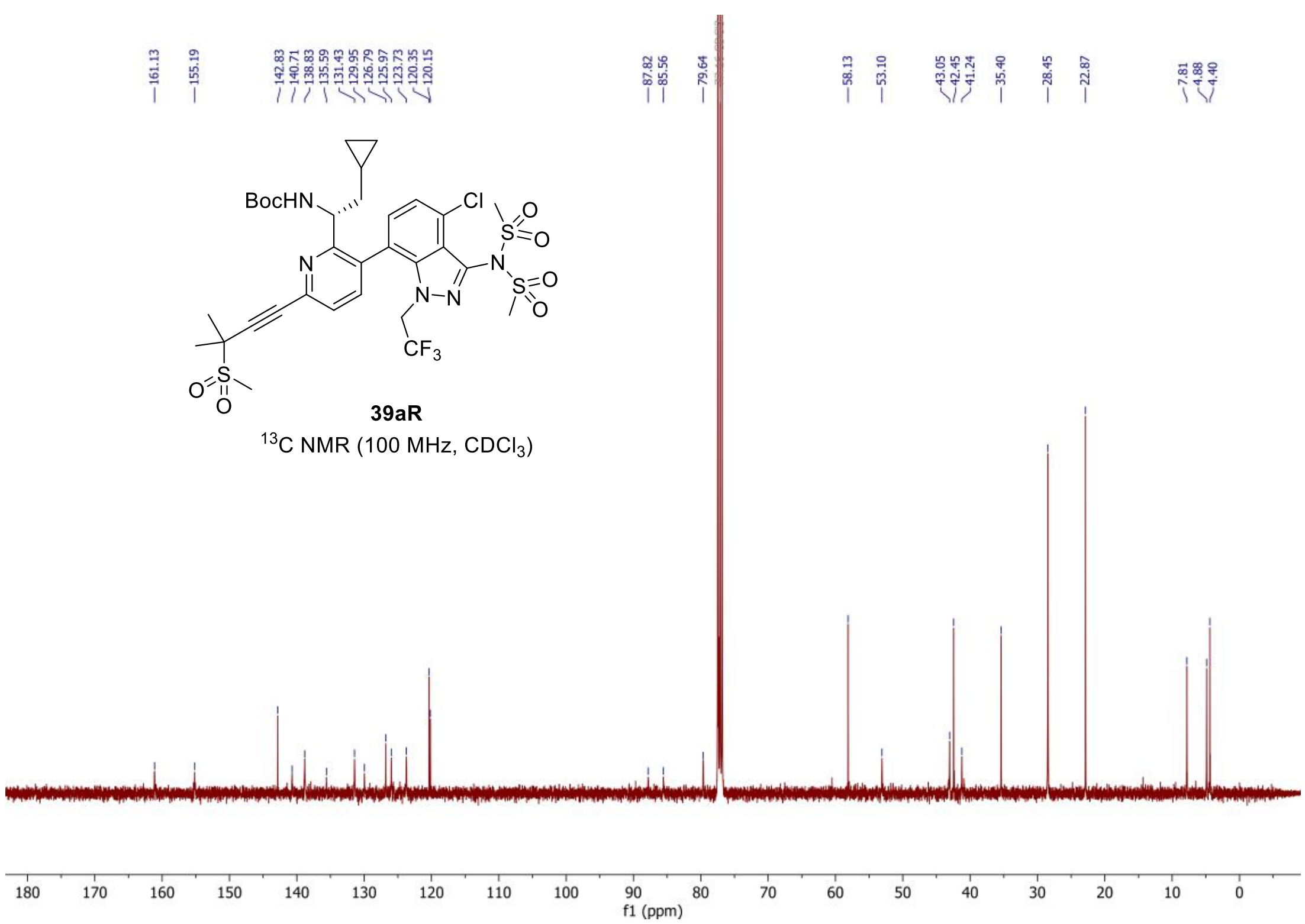

$^{19}$F NMR (376 MHz, $CDCl_3$) of compound **39aR**

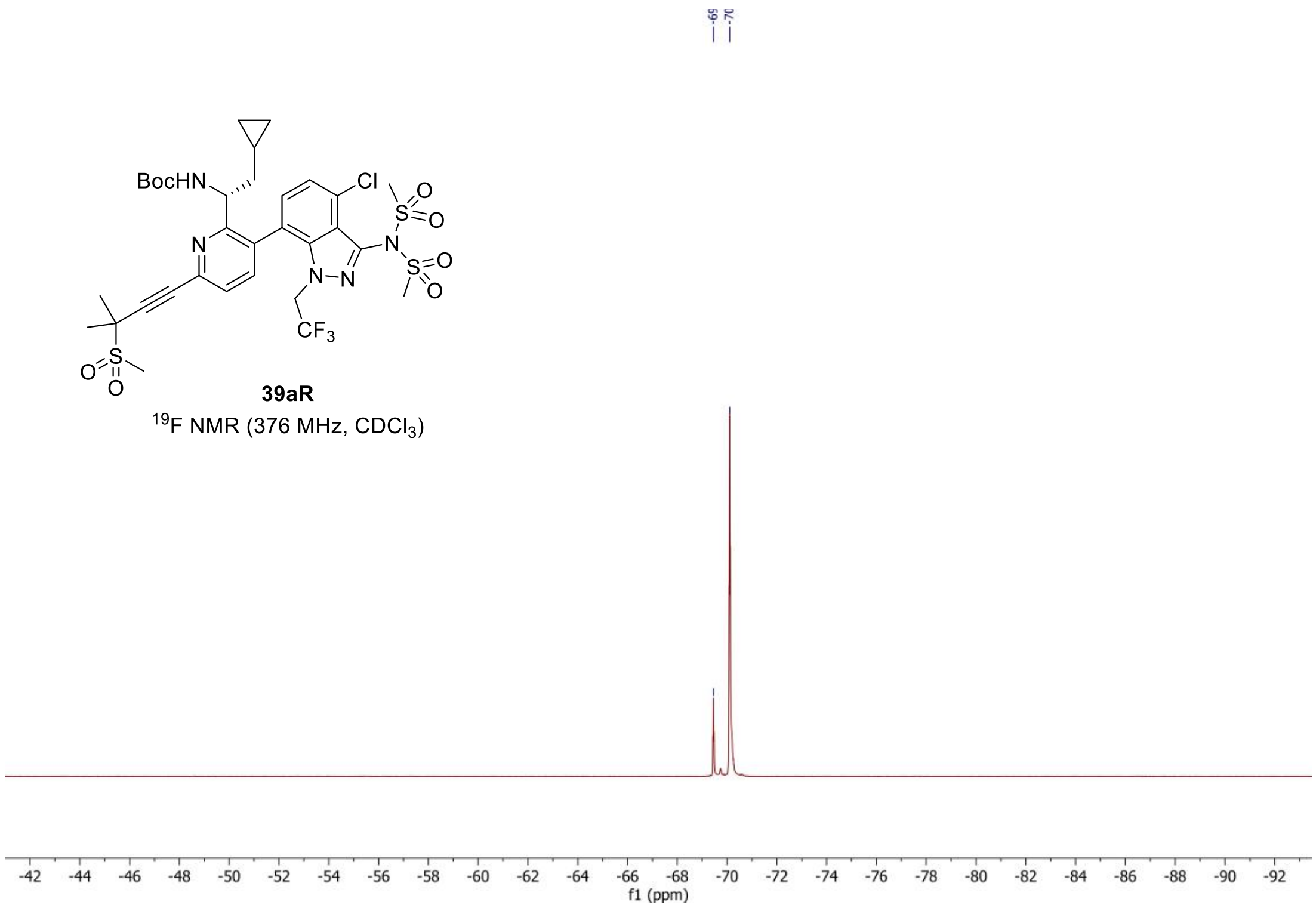

$^{1}$H NMR (400 MHz, $CDCl_3$) of the compound **39bS**

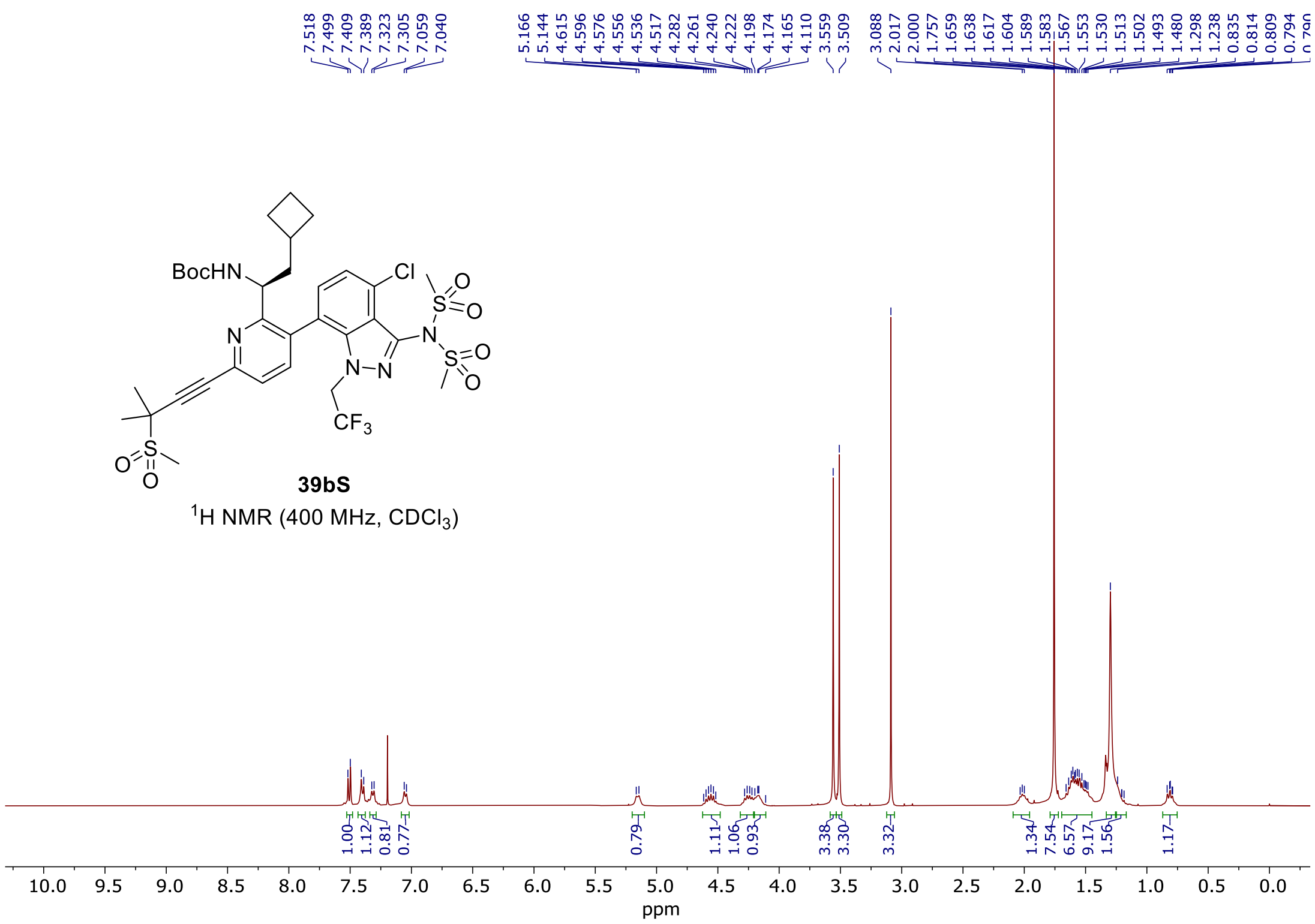


$^{13}$C NMR (100 MHz, $CDCl_3$) of compound **39bS**

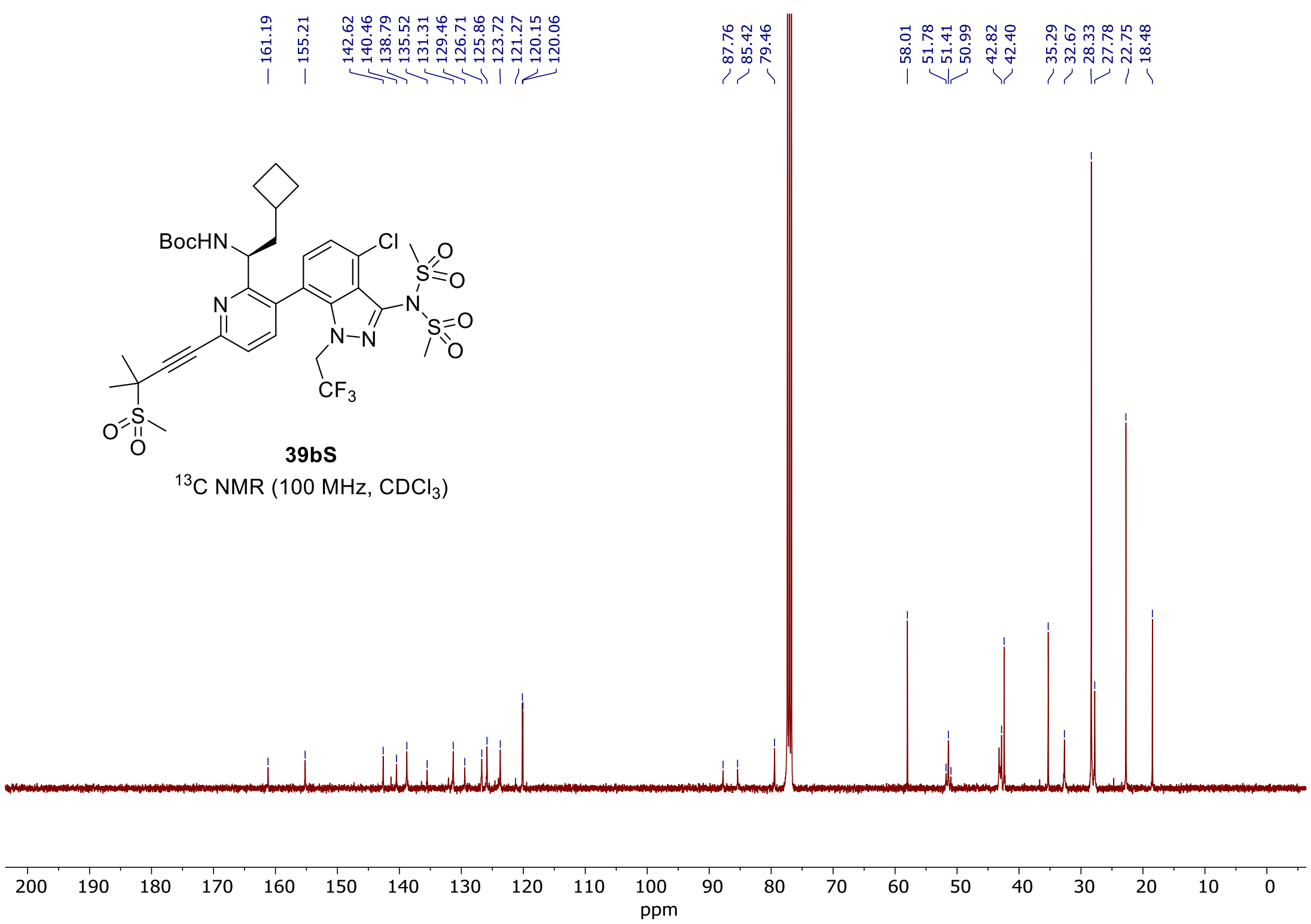

$^{19}$F NMR (376 MHz, $CDCl_3$) of compound **39bS**

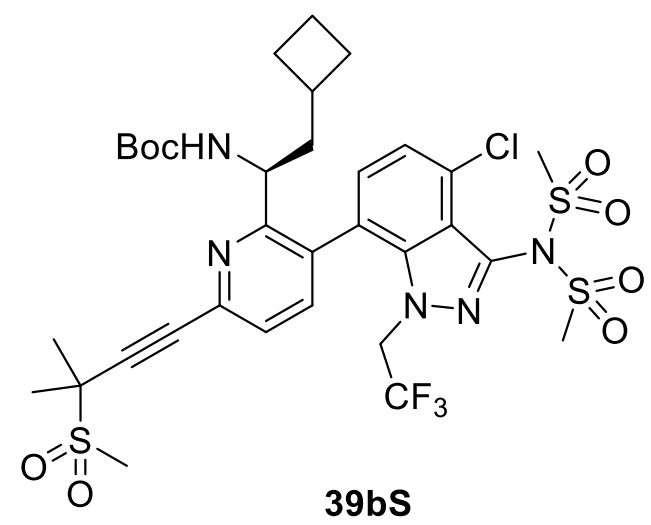


**39bS**

$^{19}$F NMR (376 MHz, $CDCl_3$)

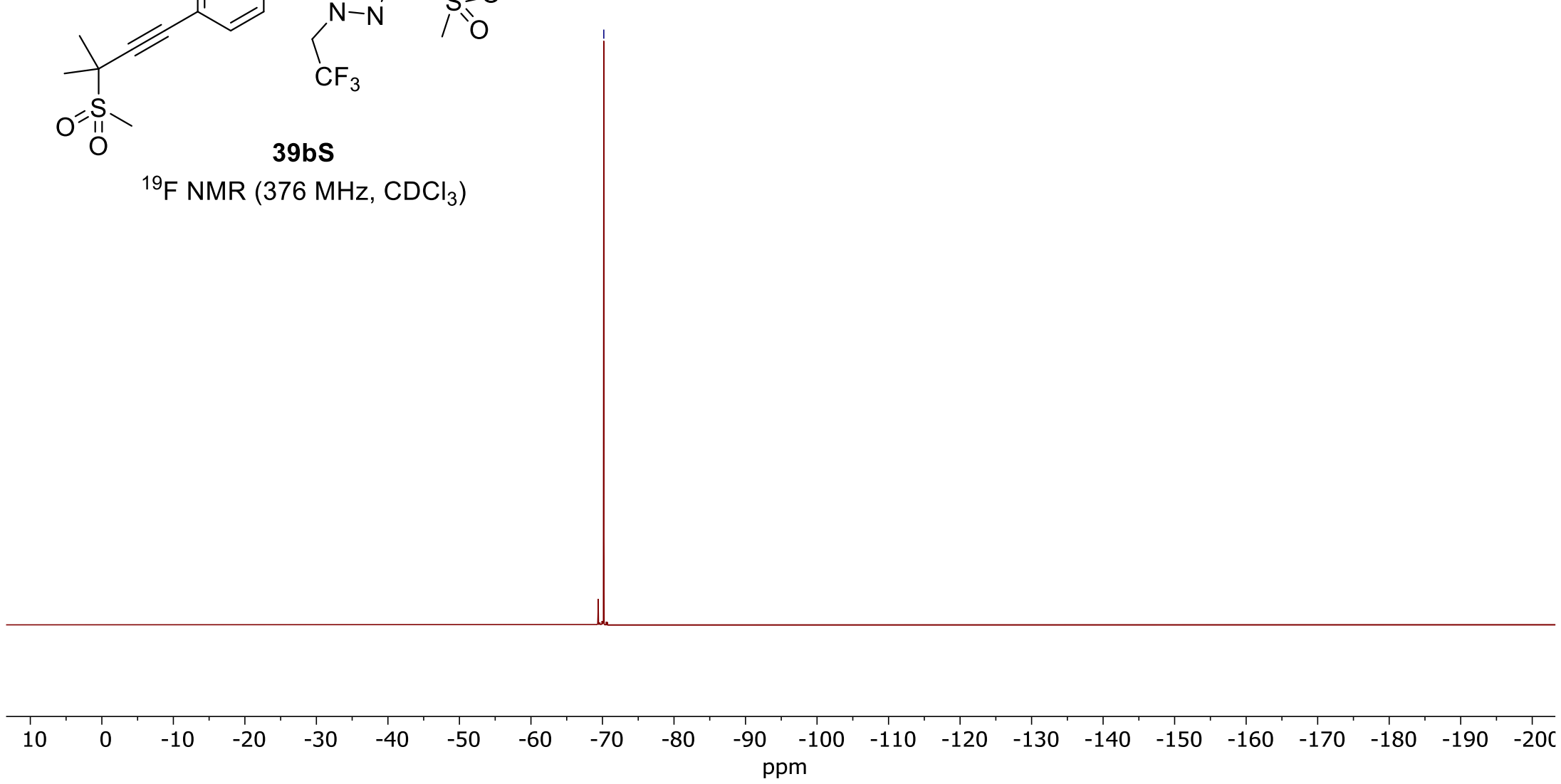

$^1$H NMR (400 MHz, $CDCl_3$) of the compound **39bR**

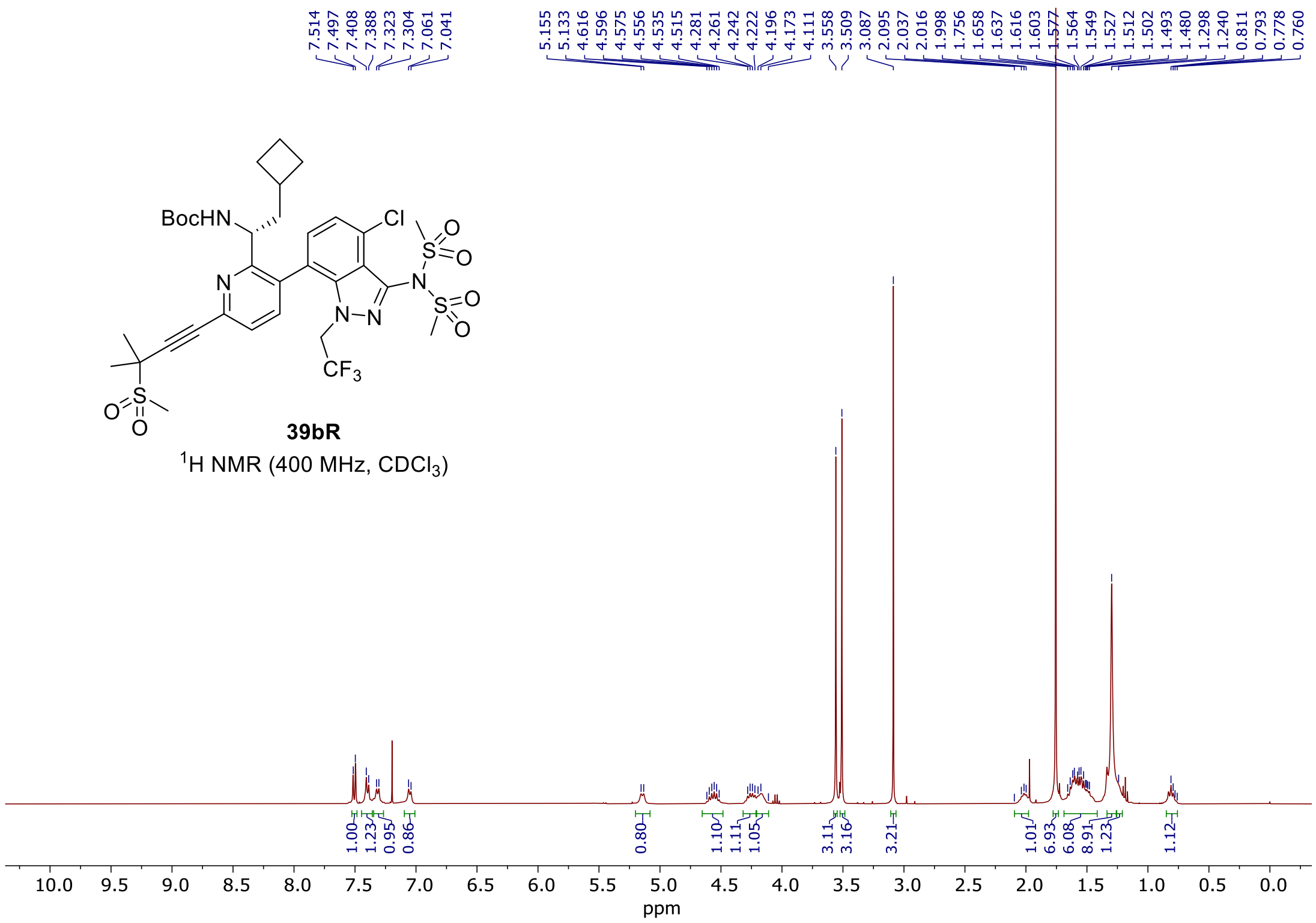


$^{13}$C NMR (100 MHz, $CDCl_3$) of compound **39bR**

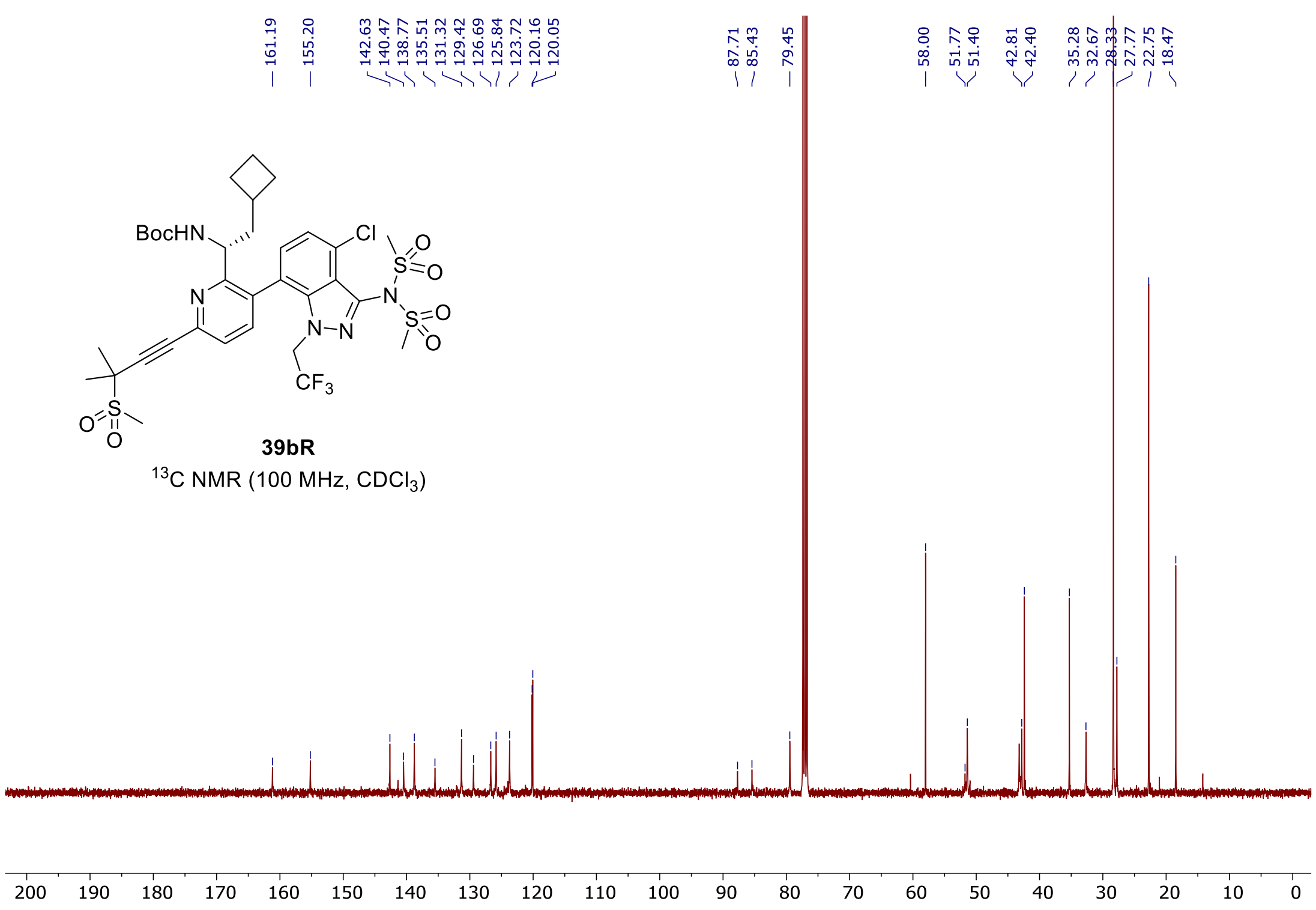

$^{19}F$ NMR (376 MHz, $CDCl_3$) of compound **39bR**

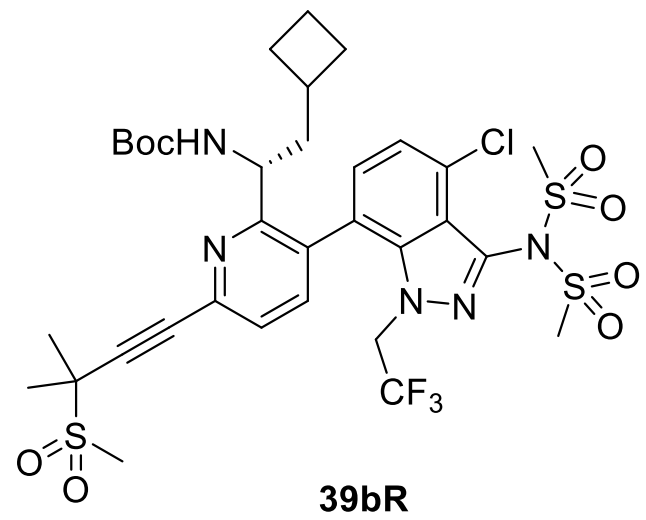


**39bR**

$^{19}F$ NMR (376 MHz, $CDCl_3$)

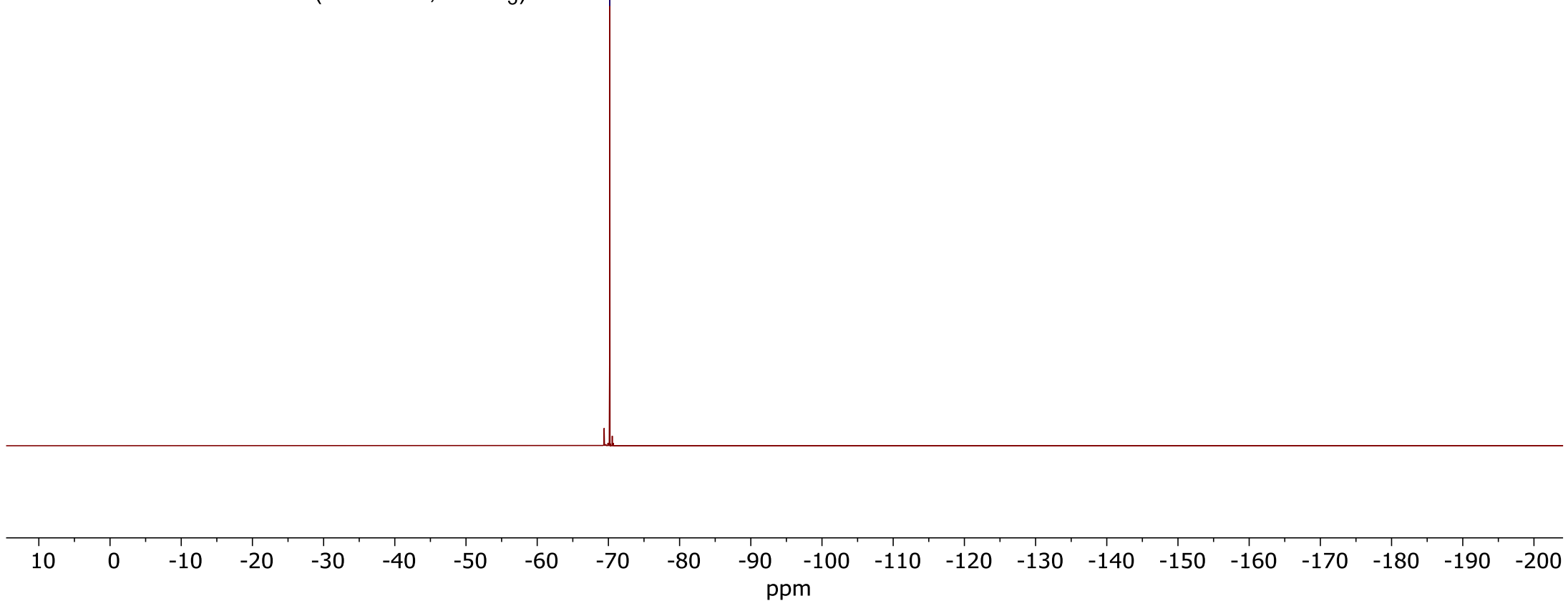

$^{1}$H NMR (400 MHz, $CDCl_3$) of the compound **39cS**

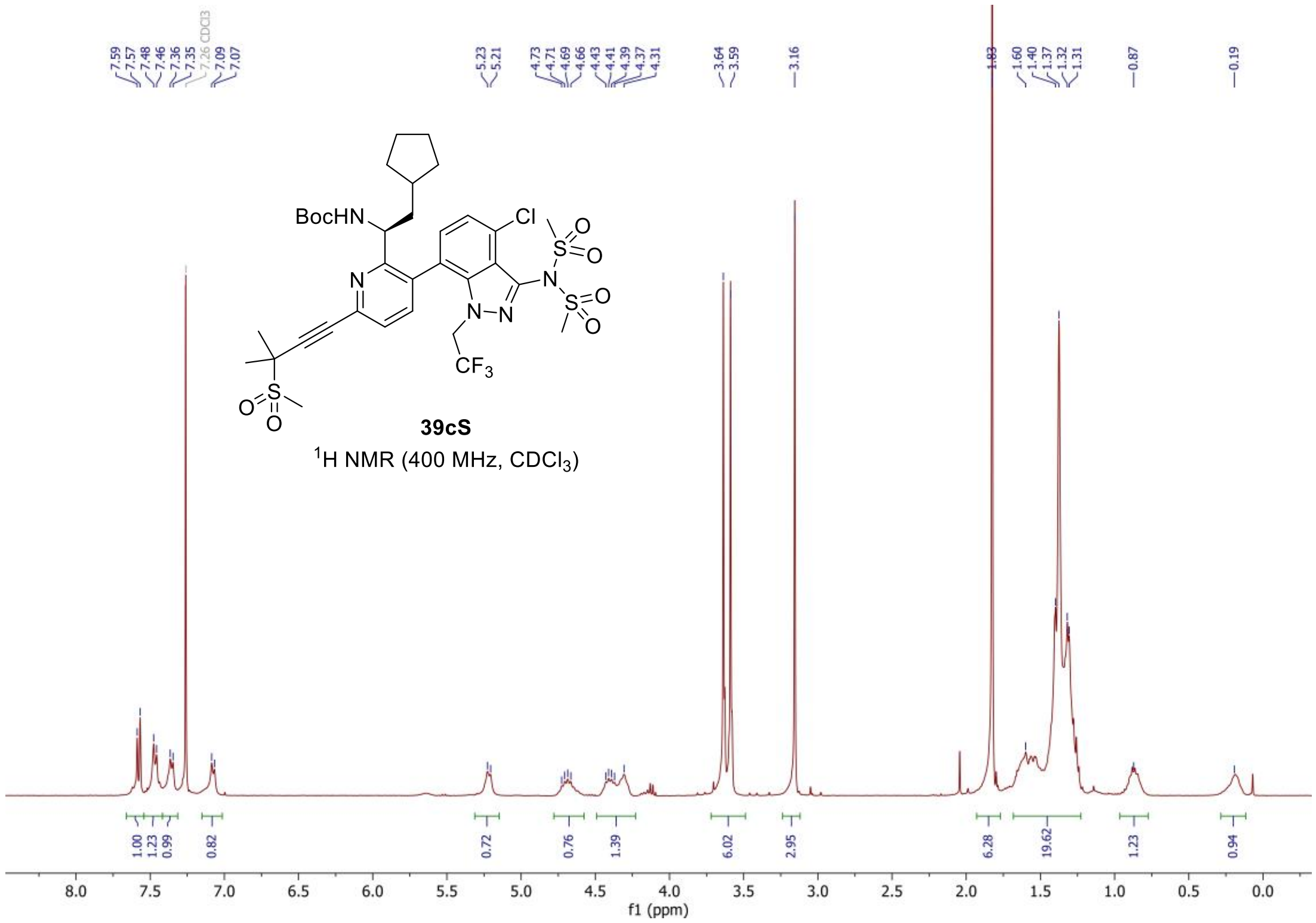


$^{13}$C NMR (100 MHz, $CDCl_3$) of compound **39cS**

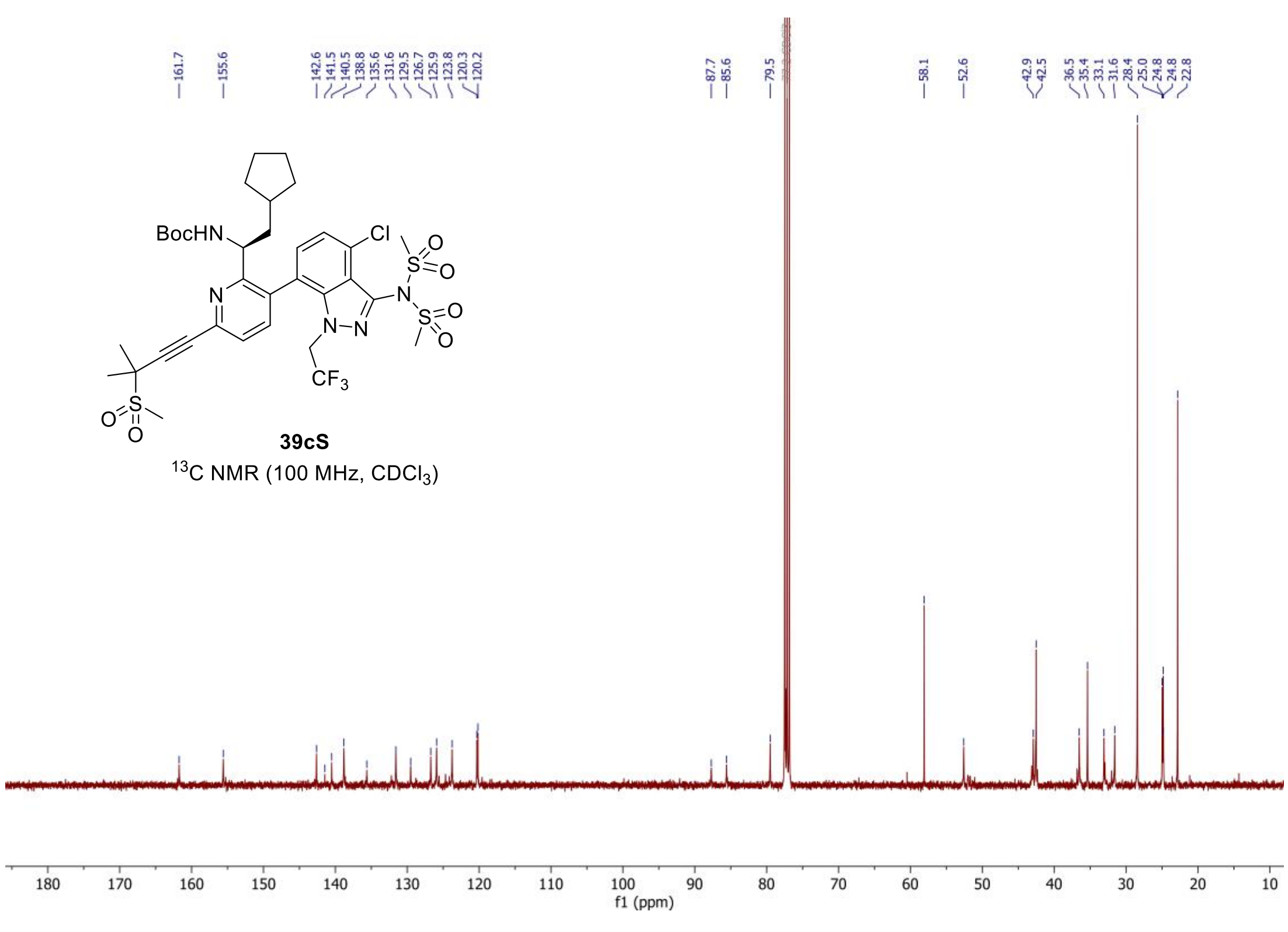

$^{1}H$ NMR (400 MHz, $CDCl_3$) of compound **39cR**

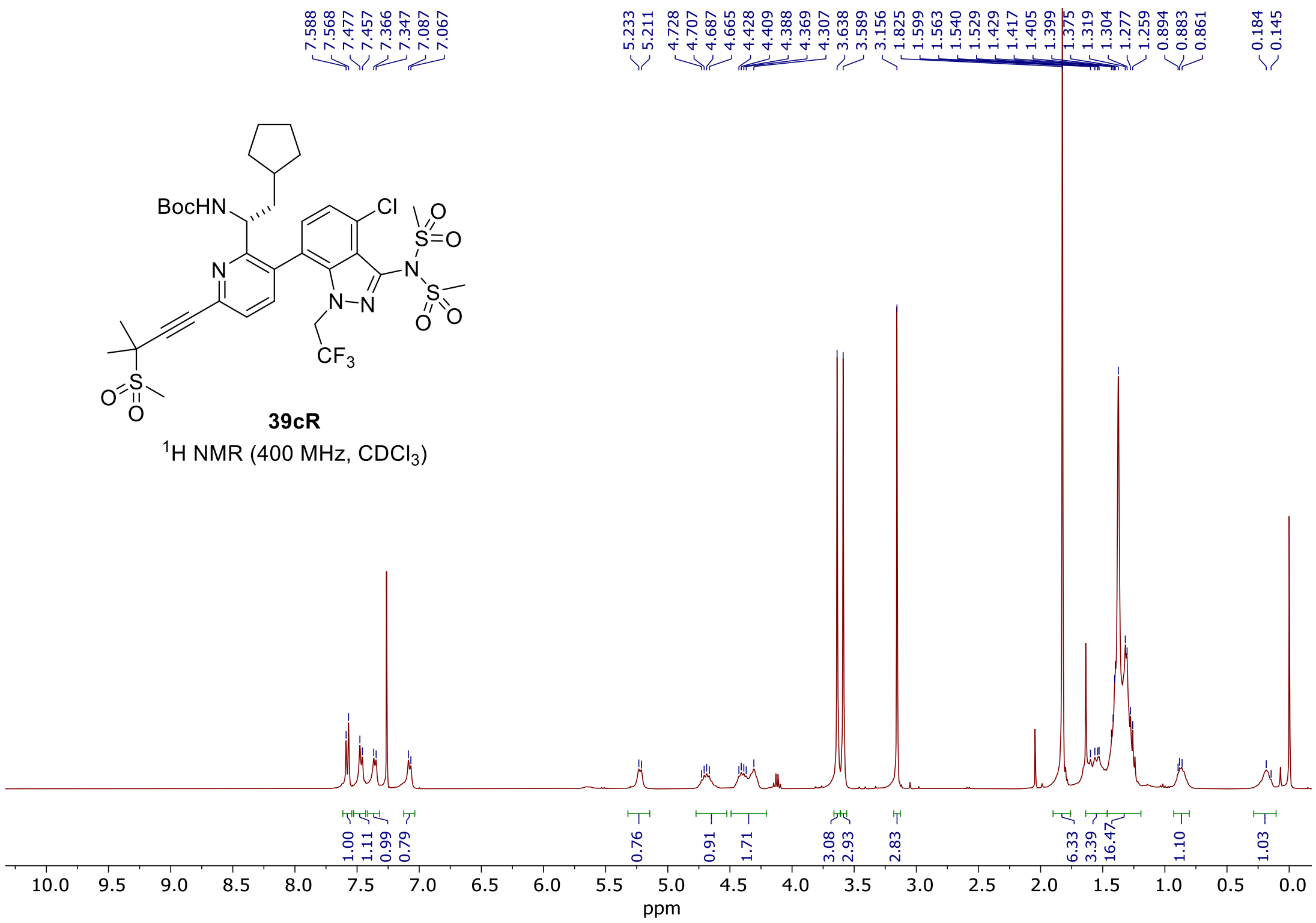


$^{13}C$ NMR (100 MHz, $CDCl_3$) of compound **39cR**

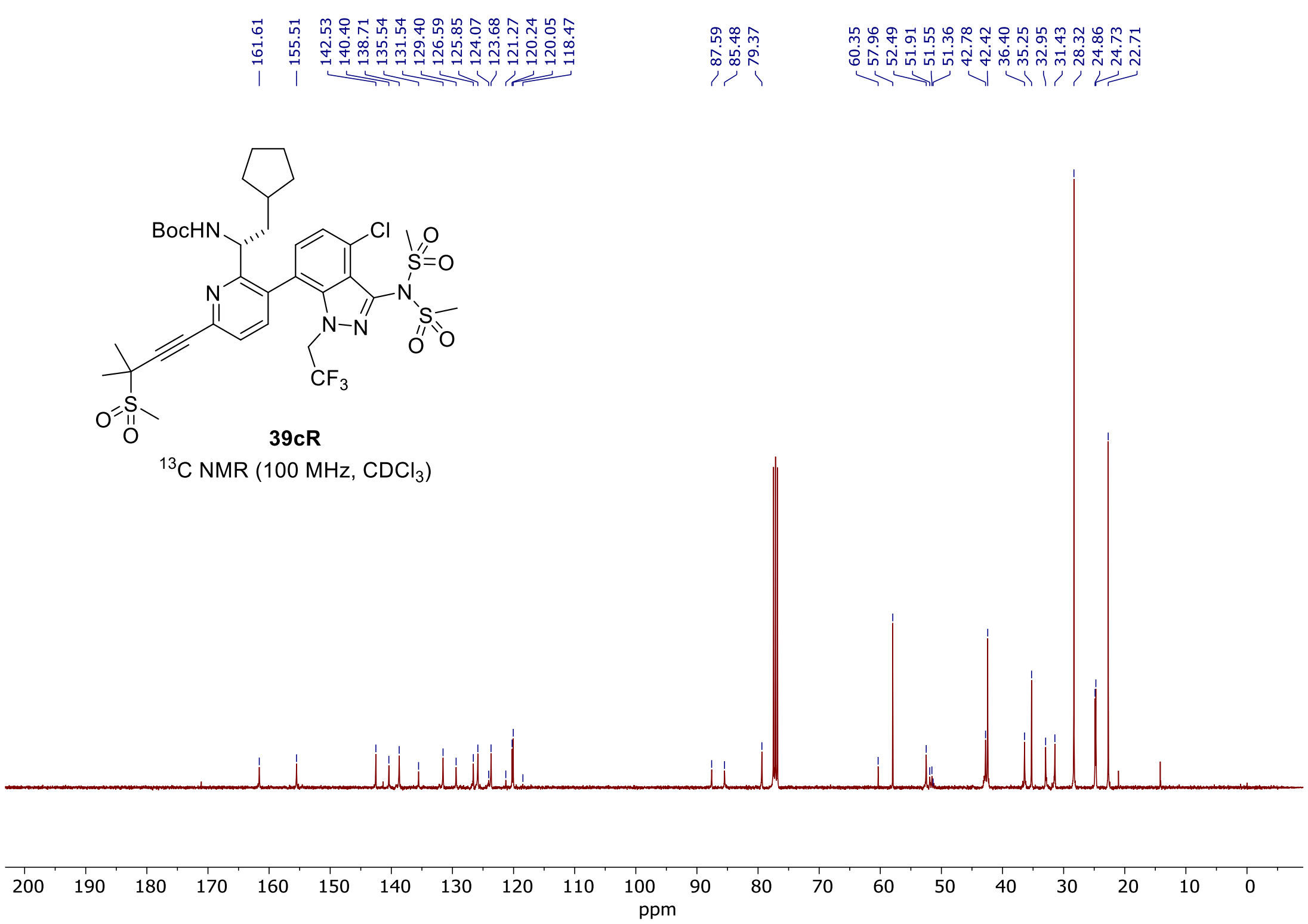

$^{19}$F NMR (376 MHz, $CDCl_3$) of compound **39cR**

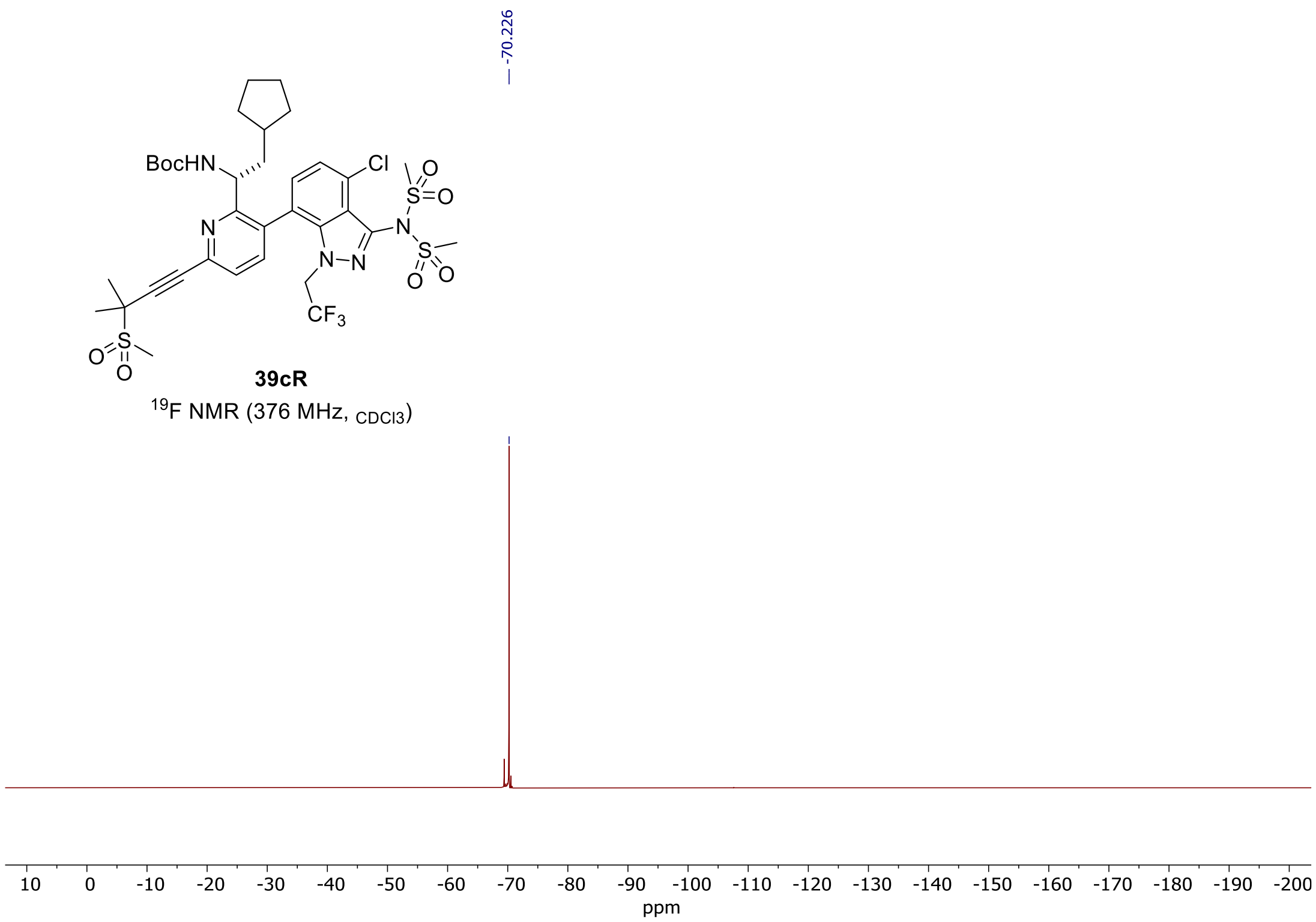

$^{1}$H NMR (400 MHz, $CDCl_3$) of compound **39dS**

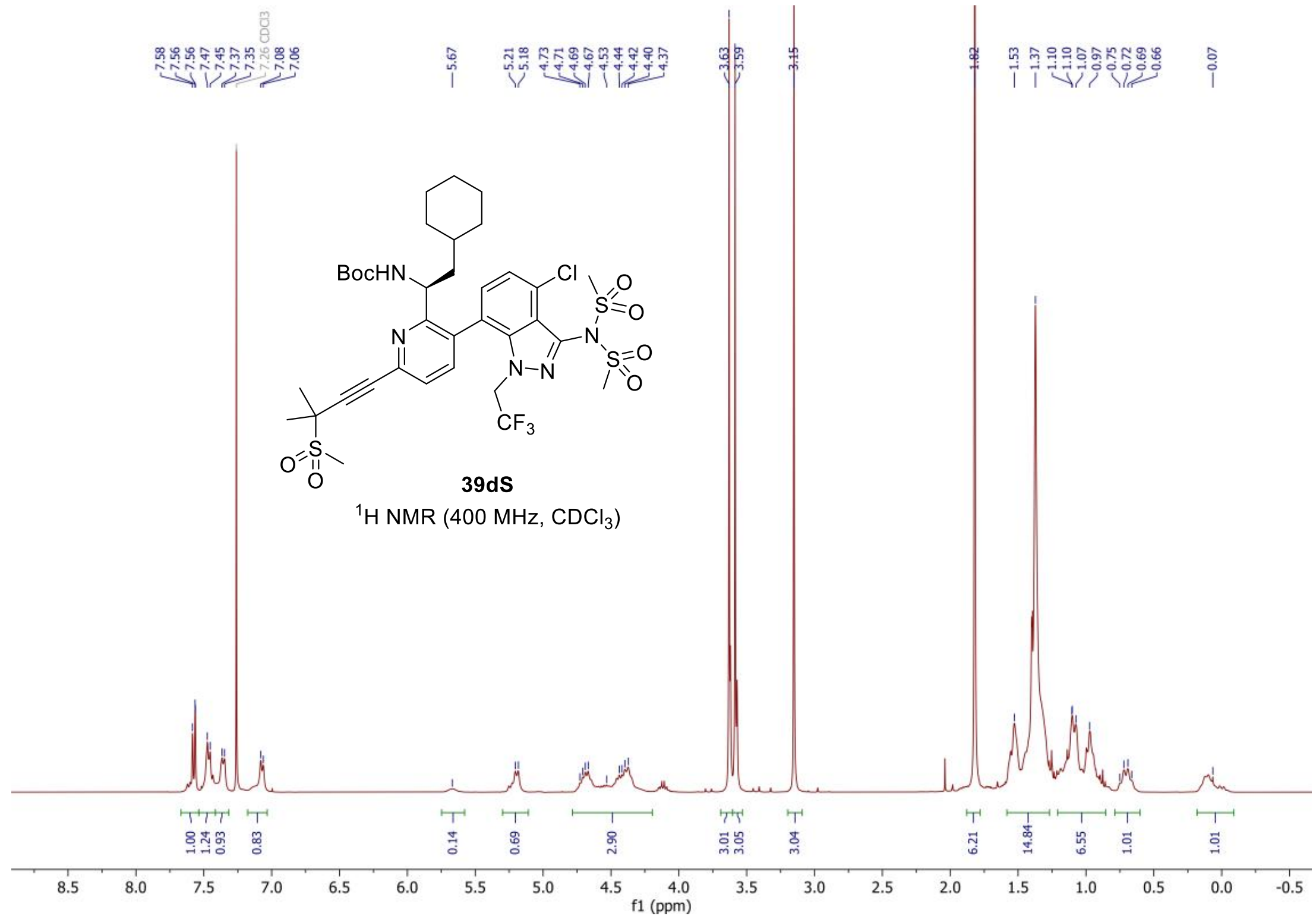

$^{13}$C NMR (100 MHz, $CDCl_3$) of compound **39dS**

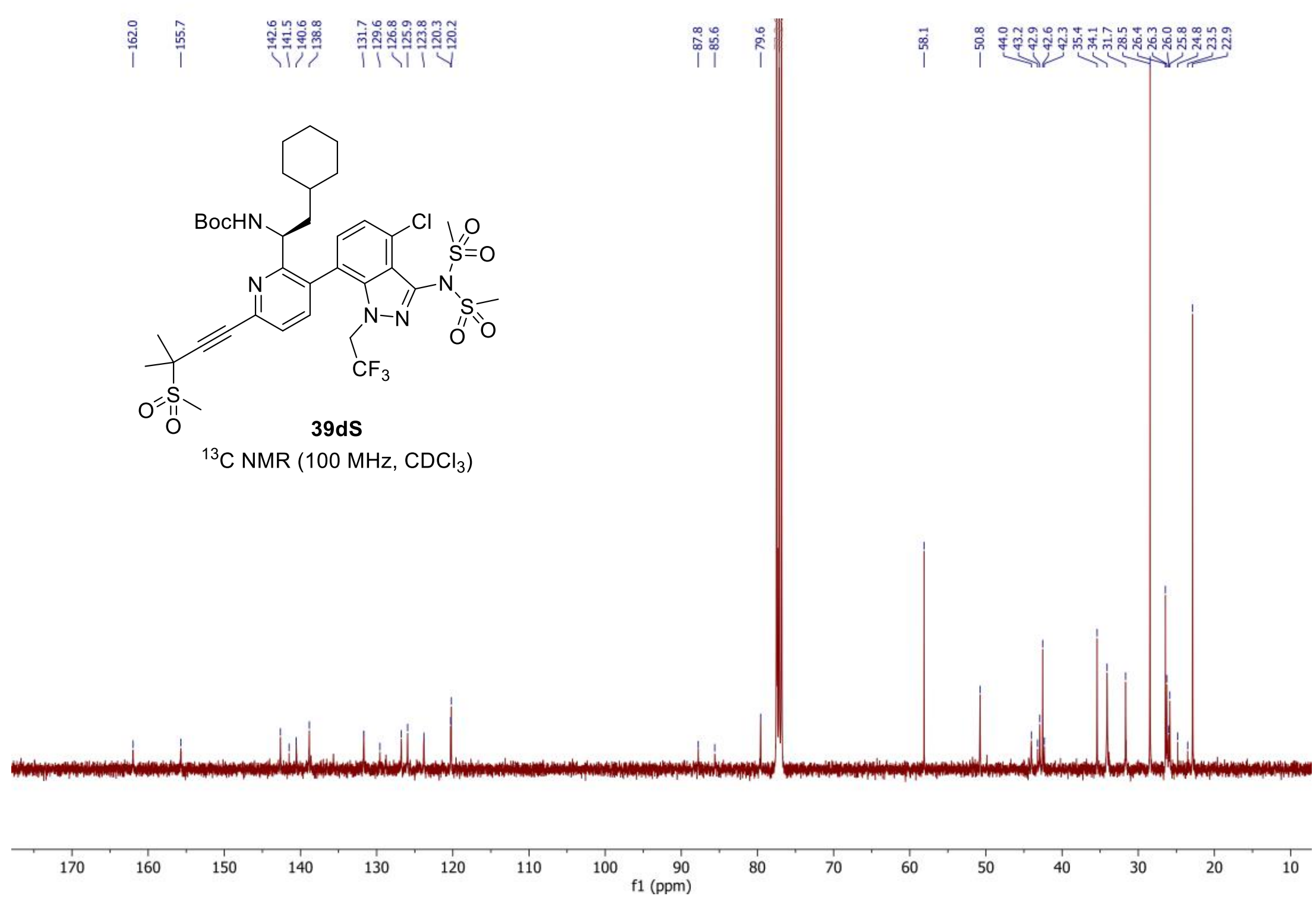

$^{1}$H NMR (400 MHz, $CDCl_3$) of compound **39dR**

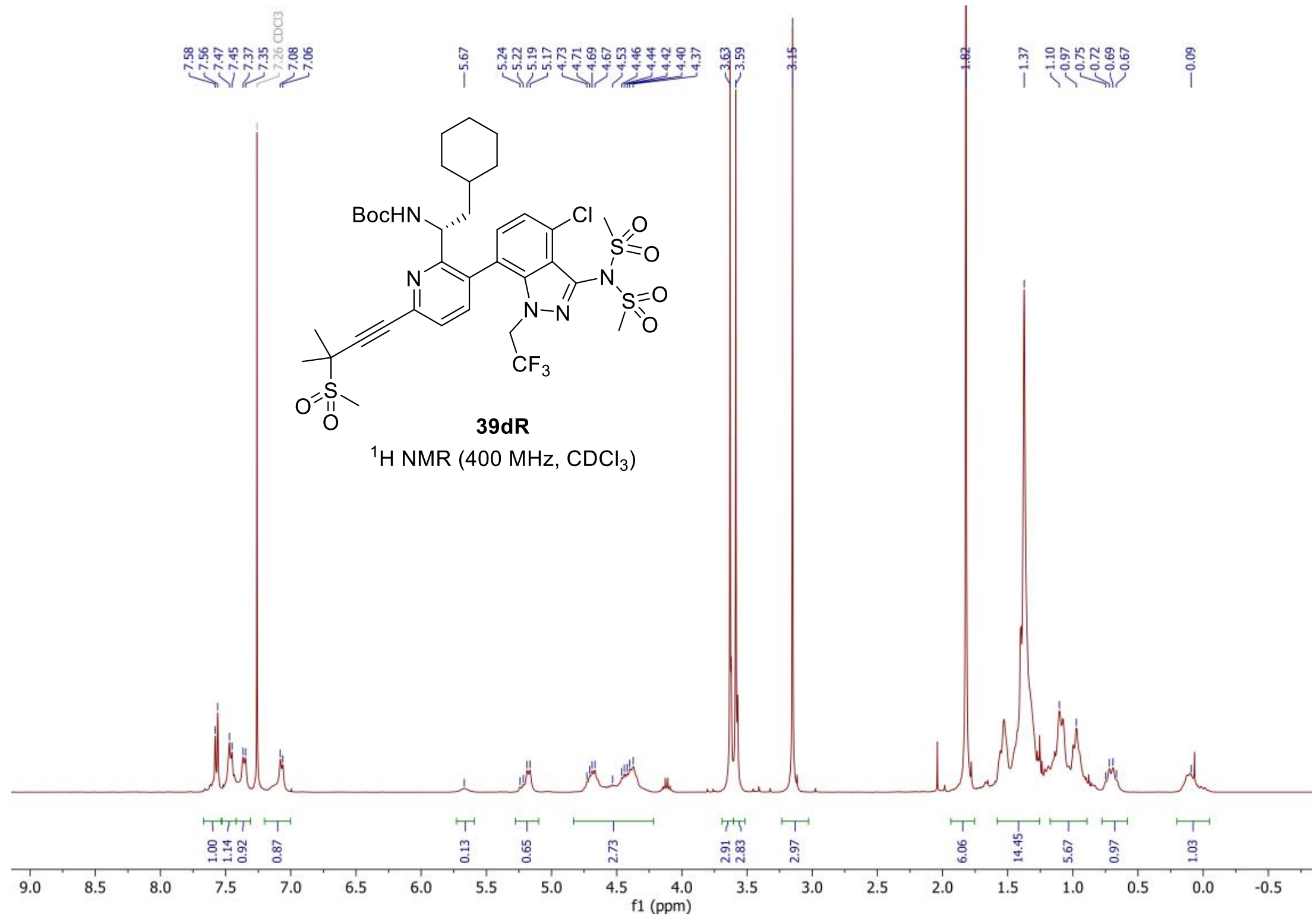


$^{13}$C NMR (100 MHz, $CDCl_3$) of compound **39dR**

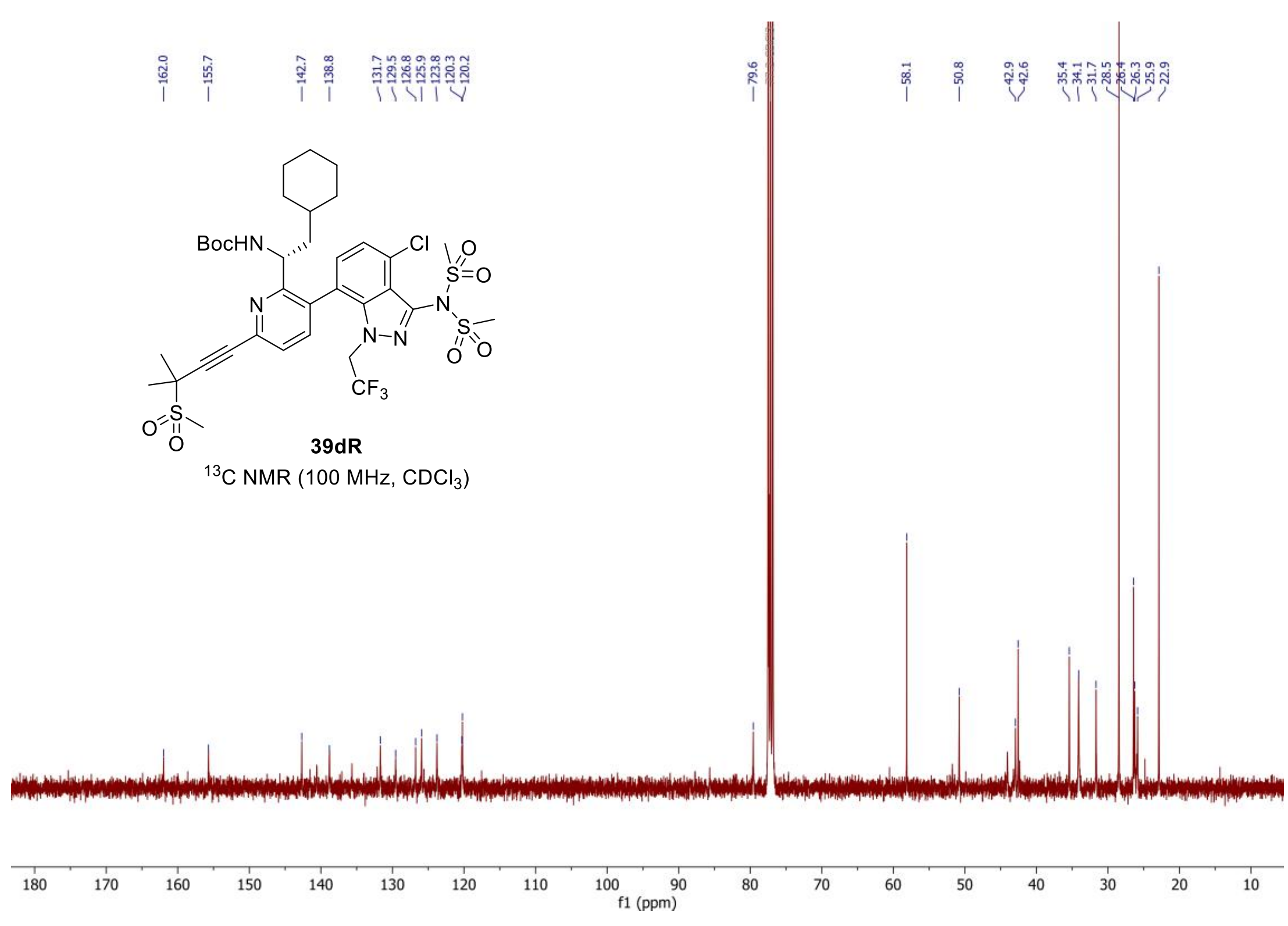

$^{1}H$ NMR (400 MHz, $CDCl_3$) of compound **1S**

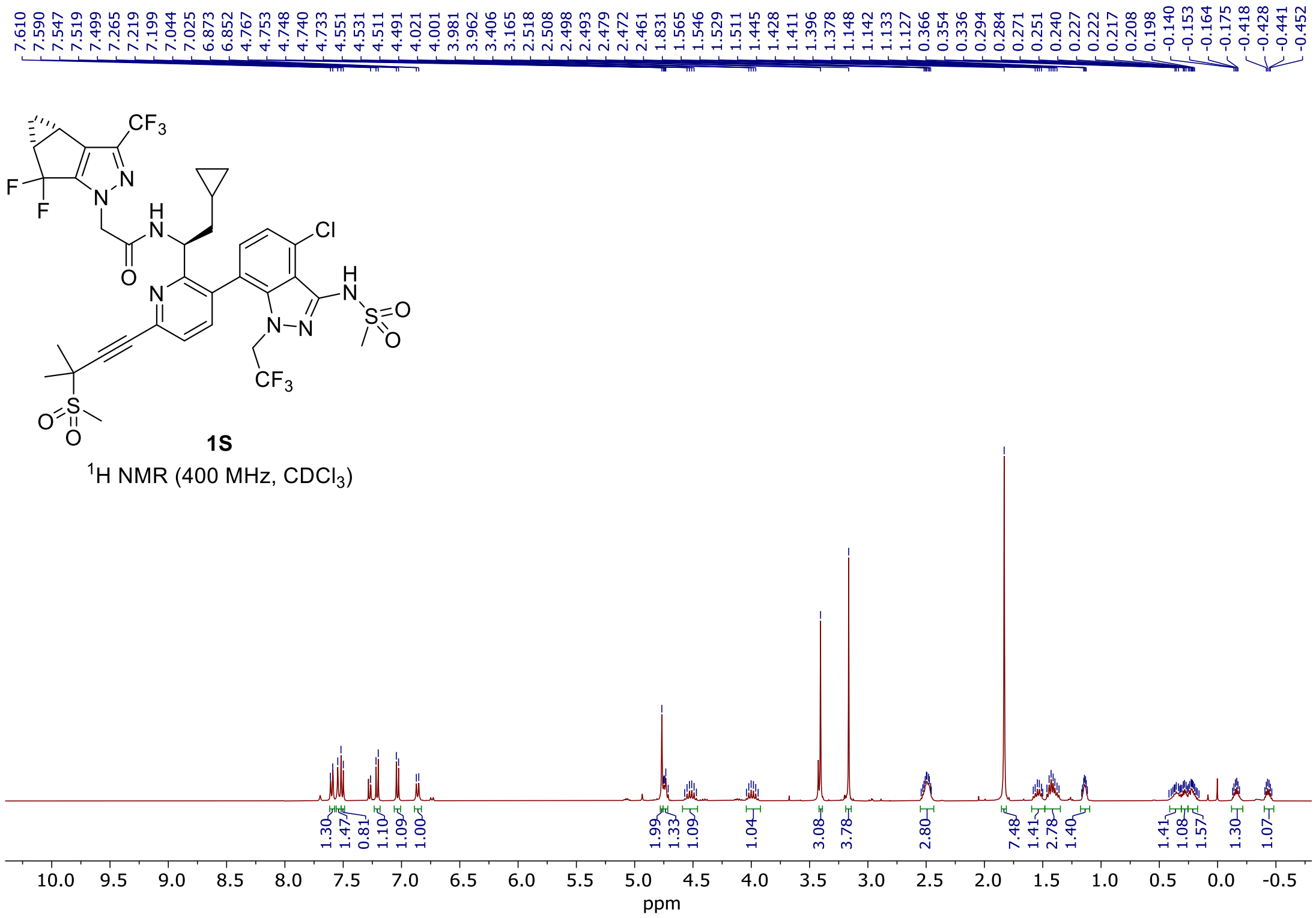


$^{13}C$ NMR (100 MHz, $CDCl_3$) of compound **1S**

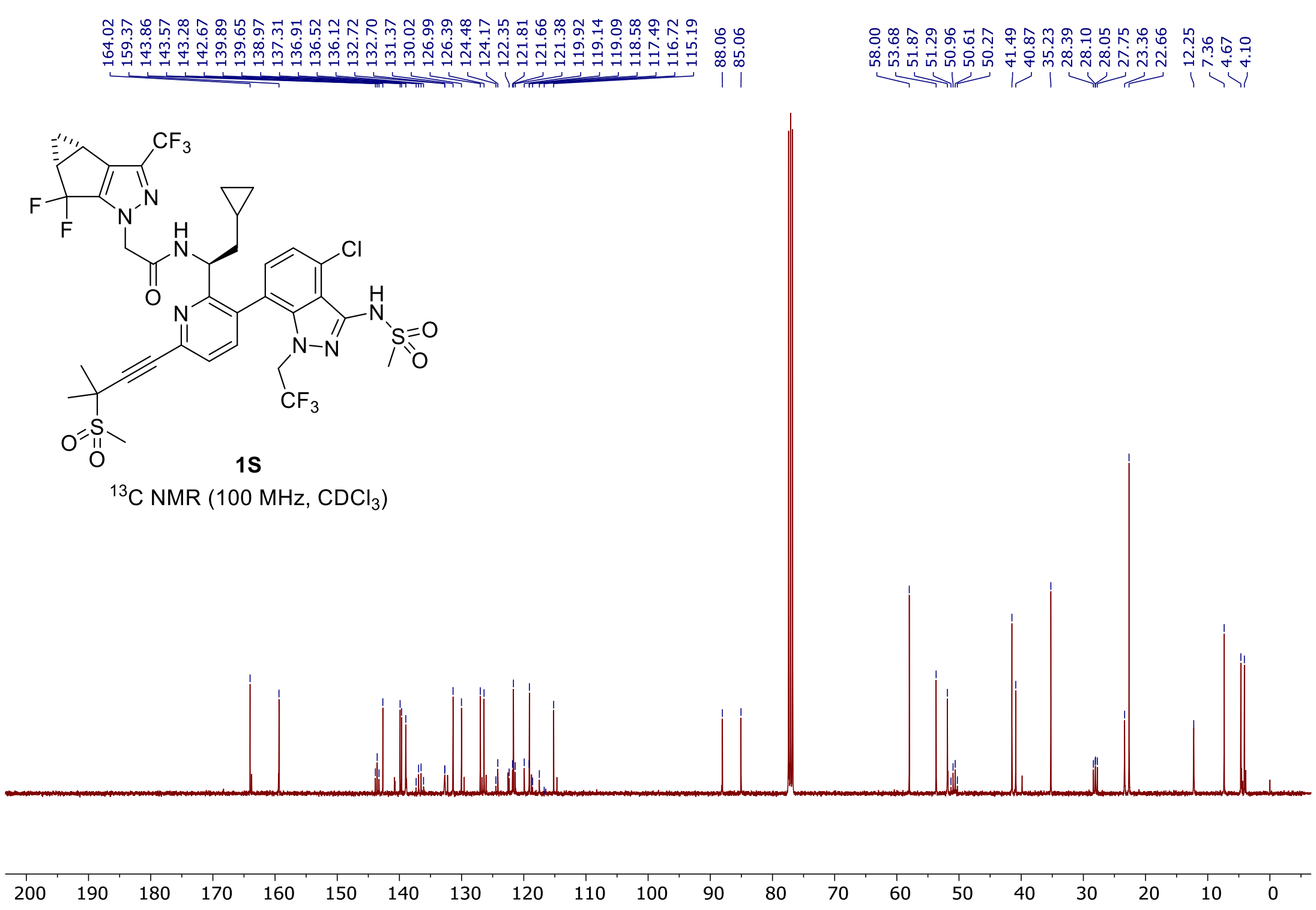

$^{19}$F NMR (376 MHz, $CDCl_3$) of compound **1S**

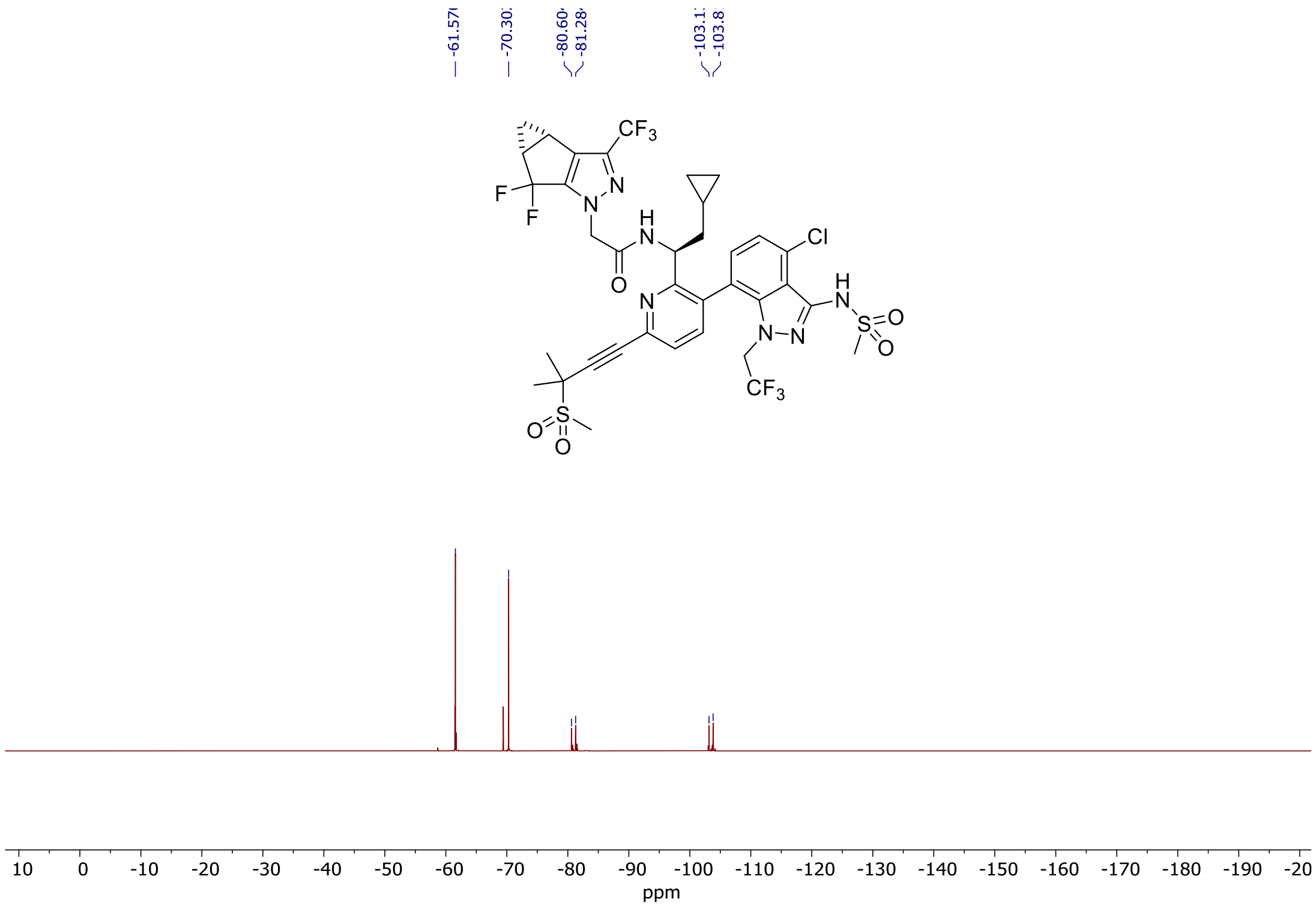


HPLC data of compound **1S**

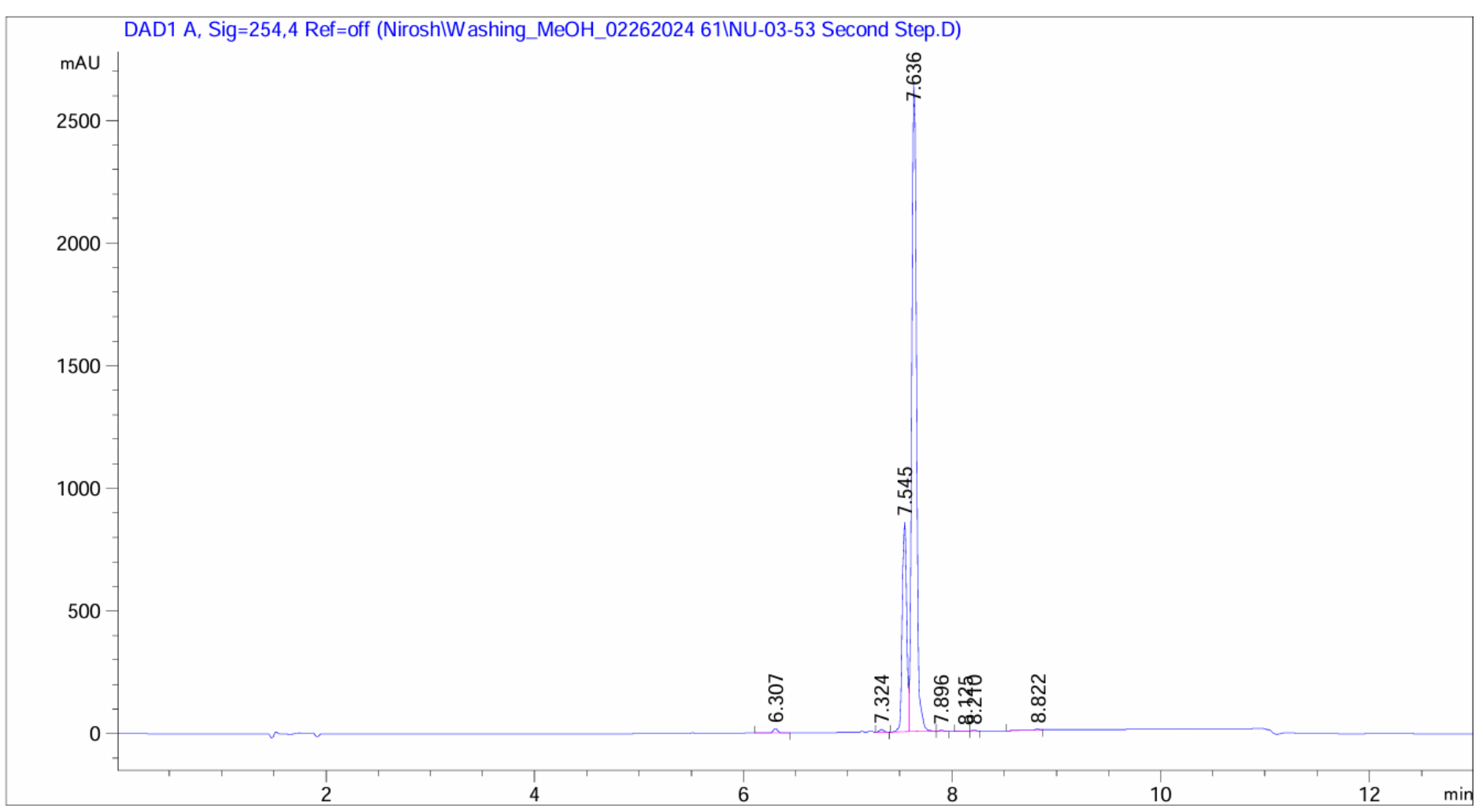


| Peak # | RetTime [min] | Type | Width [min] | Area [mAU*s] | Height [mAU] | Area % | |
|---|---|---|---|---|---|---|---|
| 1 | 6.307 | BV R | 0.0484 | 52.44106 | 16.81845 | 0.4815 | |
| 2 | 7.324 | VB | 0.0478 | 29.69723 | 10.26278 | 0.2727 | |
| 3 | 7.545 | BV | 0.0456 | 2467.40967 | 855.76593 | 22.6550 | (atropisomer) |
| 4 | 7.636 | VB | 0.0507 | 8298.75000 | 2639.34937 | 76.1965 | (atropisomer) |
| 5 | 7.896 | BB | 0.0435 | 9.60847 | 3.56082 | 0.0882 | |
| 6 | 8.125 | BV | 0.0655 | 8.18660 | 1.78611 | 0.0752 | |
| 7 | 8.210 | VB | 0.0453 | 11.35900 | 3.97661 | 0.1043 | |
| 8 | 8.822 | BB | 0.0608 | 13.79661 | 3.29512 | 0.1267 | |
| Totals : | | | | 1.08912e4 | 3534.81520 | | |

$^{1}$H NMR (400 MHz, $CDCl_3$) of compound **1R**

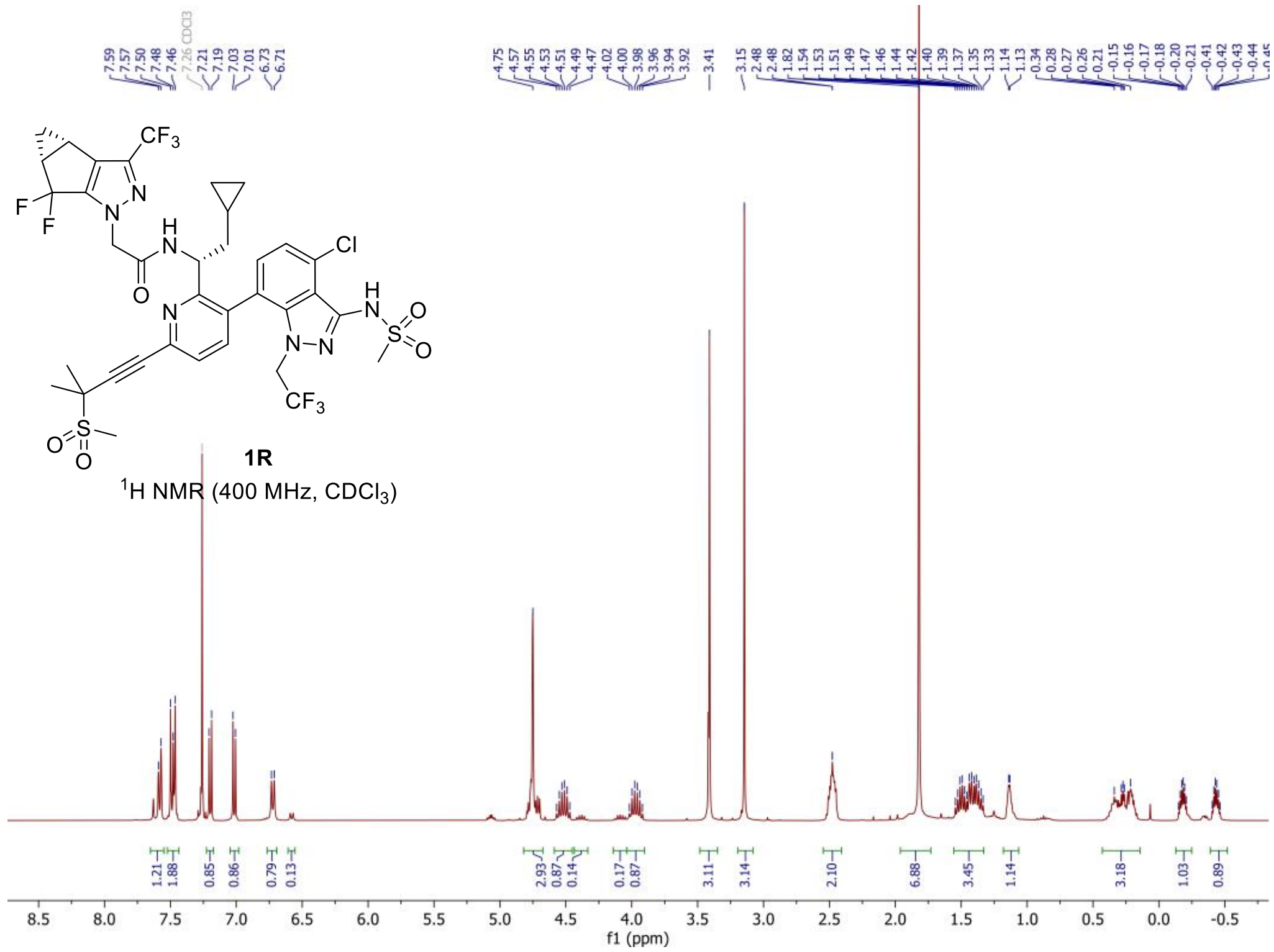


$^{13}$C NMR (100 MHz, $CDCl_3$) of compound **1R**

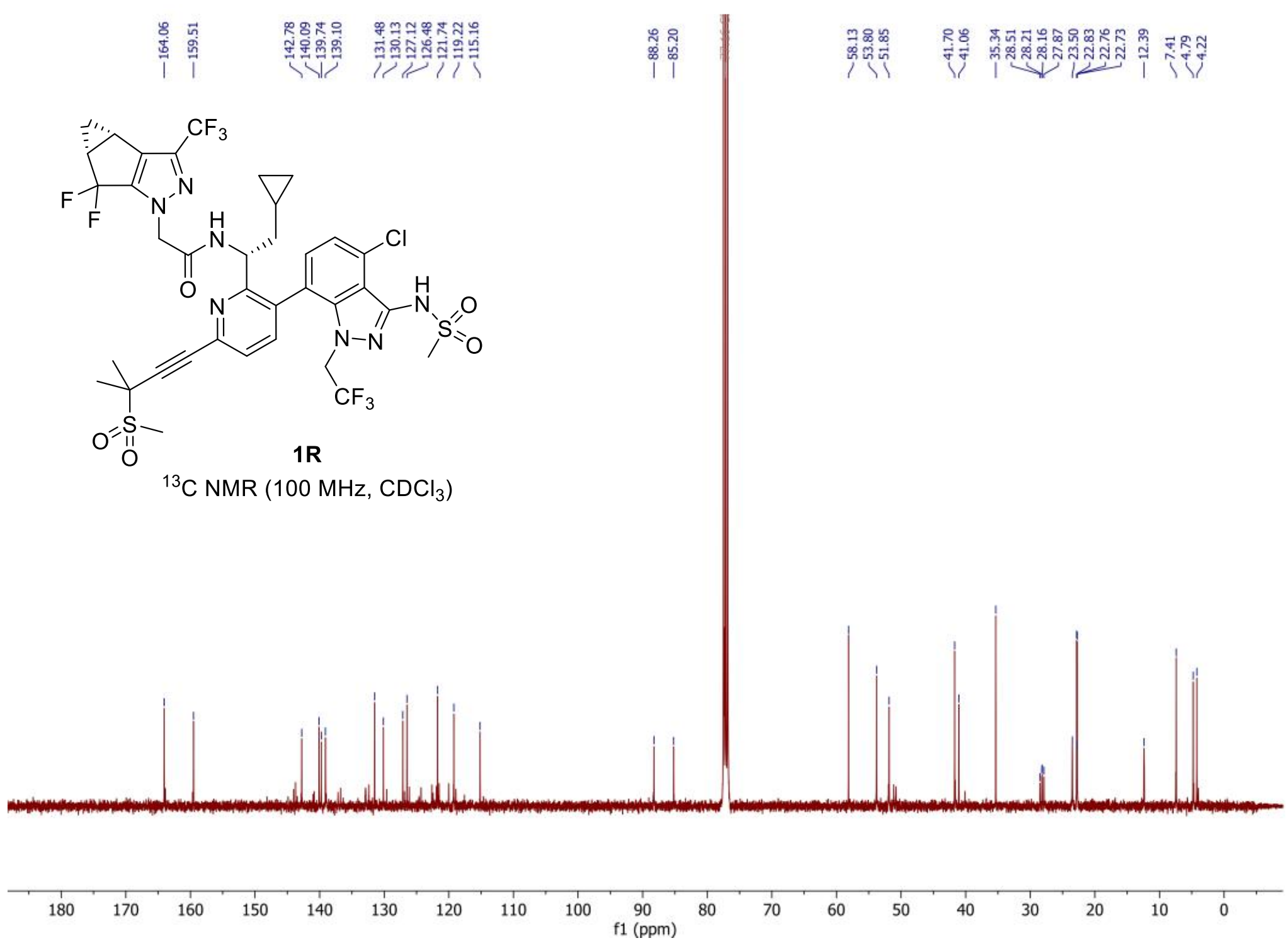

HPLC data of compound **1R**

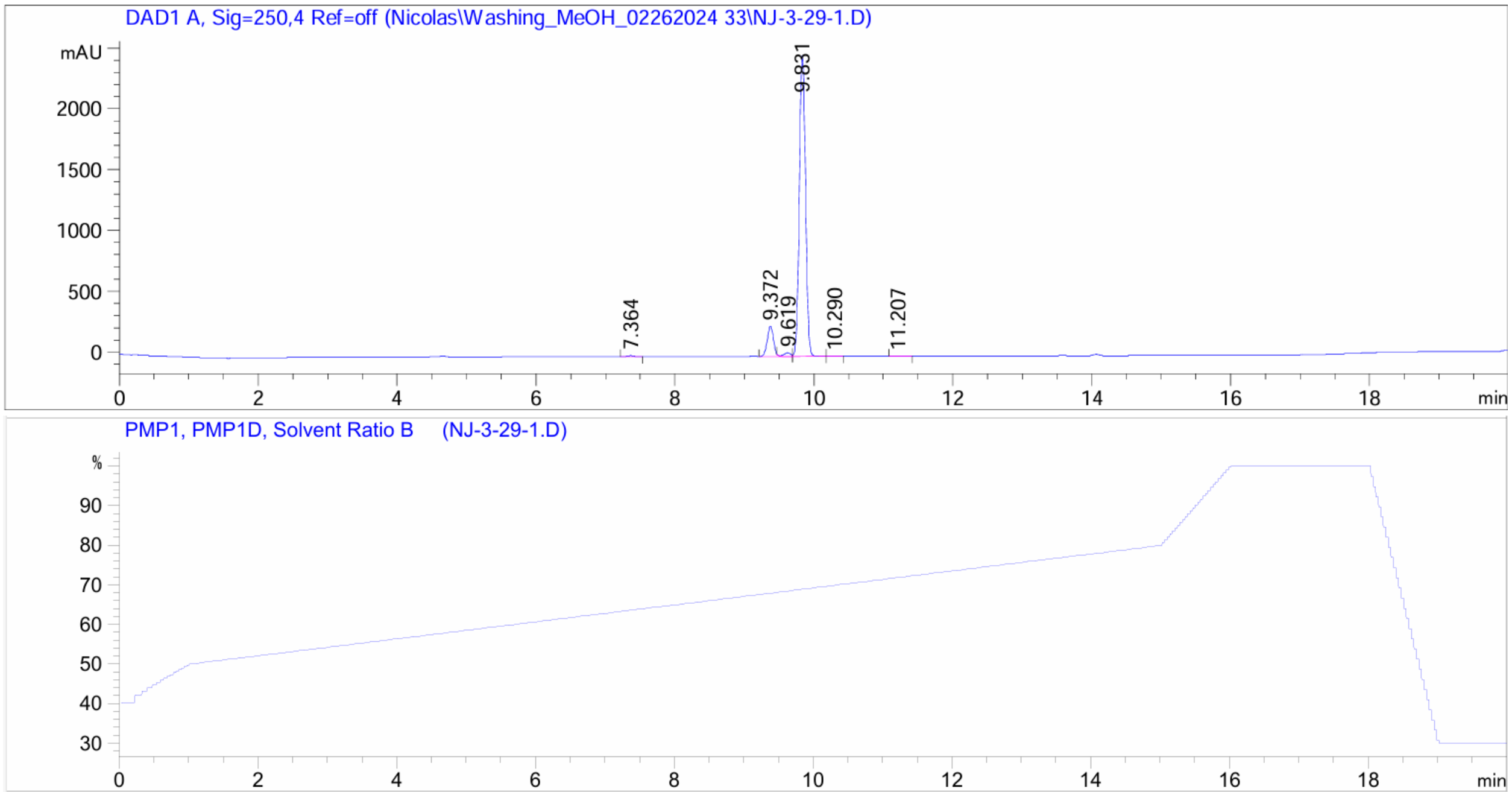


| Peak # | RetTime [min] | Type | Width [min] | Area [mAU*s] | Height [mAU] | Area % | |
|---|---|---|---|---|---|---|---|
| 1 | 7.364 | BB | 0.0962 | 53.29251 | 8.61041 | 0.3117 | |
| 2 | 9.372 | BV R | 0.0963 | 1538.86975 | 248.15282 | 9.0015 | (atropisomer) |
| 3 | 9.619 | VV E | 0.0968 | 185.91624 | 28.98806 | 1.0875 | |
| 4 | 9.831 | VB | 0.0982 | 1.52749e4 | 2468.86523 | 89.3497 | (atropisomer) |
| 5 | 10.290 | BB | 0.0913 | 16.56424 | 2.87147 | 0.0969 | |
| 6 | 11.207 | VB | 0.0975 | 26.09621 | 4.14398 | 0.1526 | |
| Totals : | | | | 1.70956e4 | 2761.63198 | | |

$^{1}H$ NMR (400 MHz, $CDCl_3$) of the compound **2S**

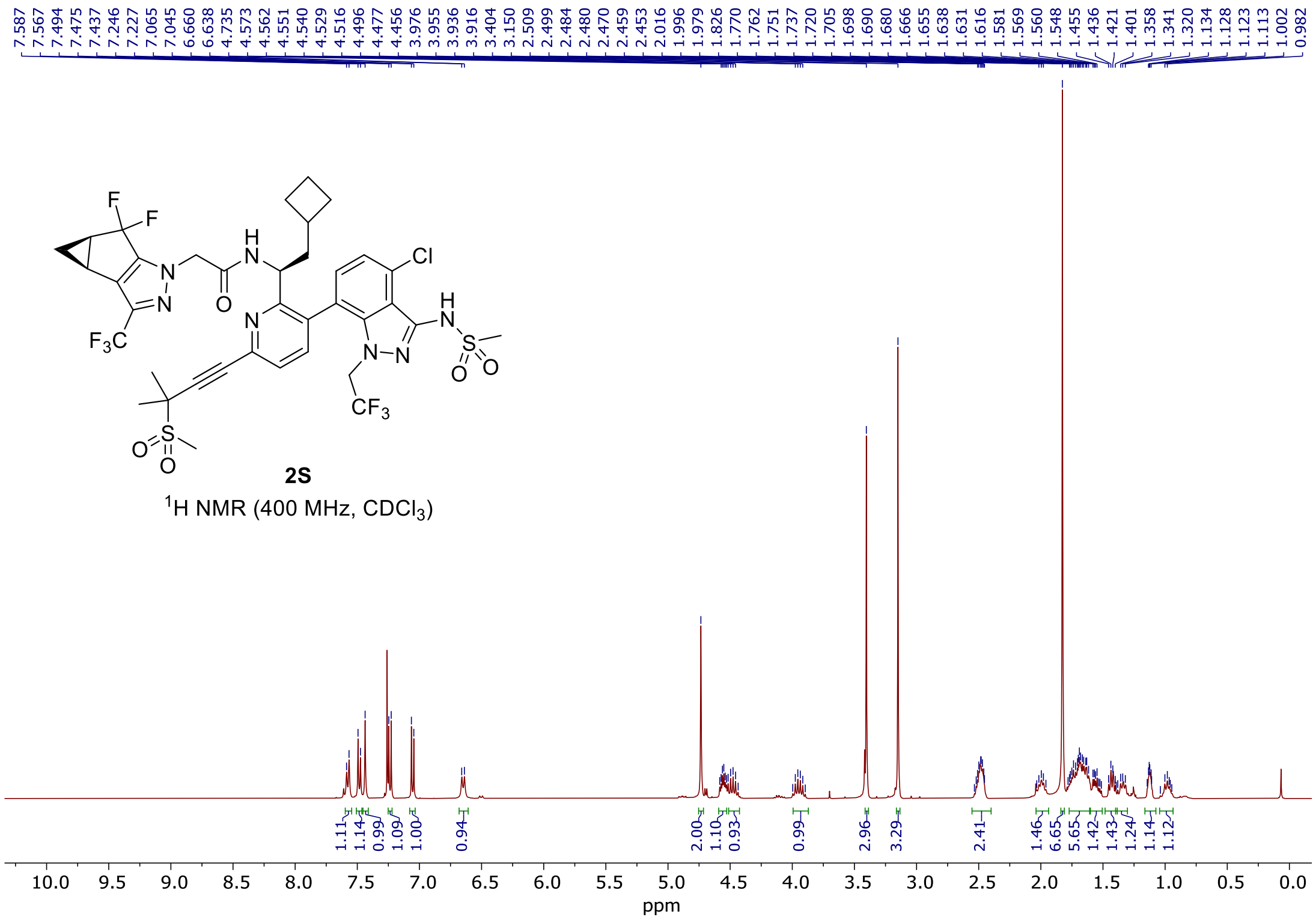


$^{13}C$ NMR (100 MHz, $CDCl_3$) of compound **2S**

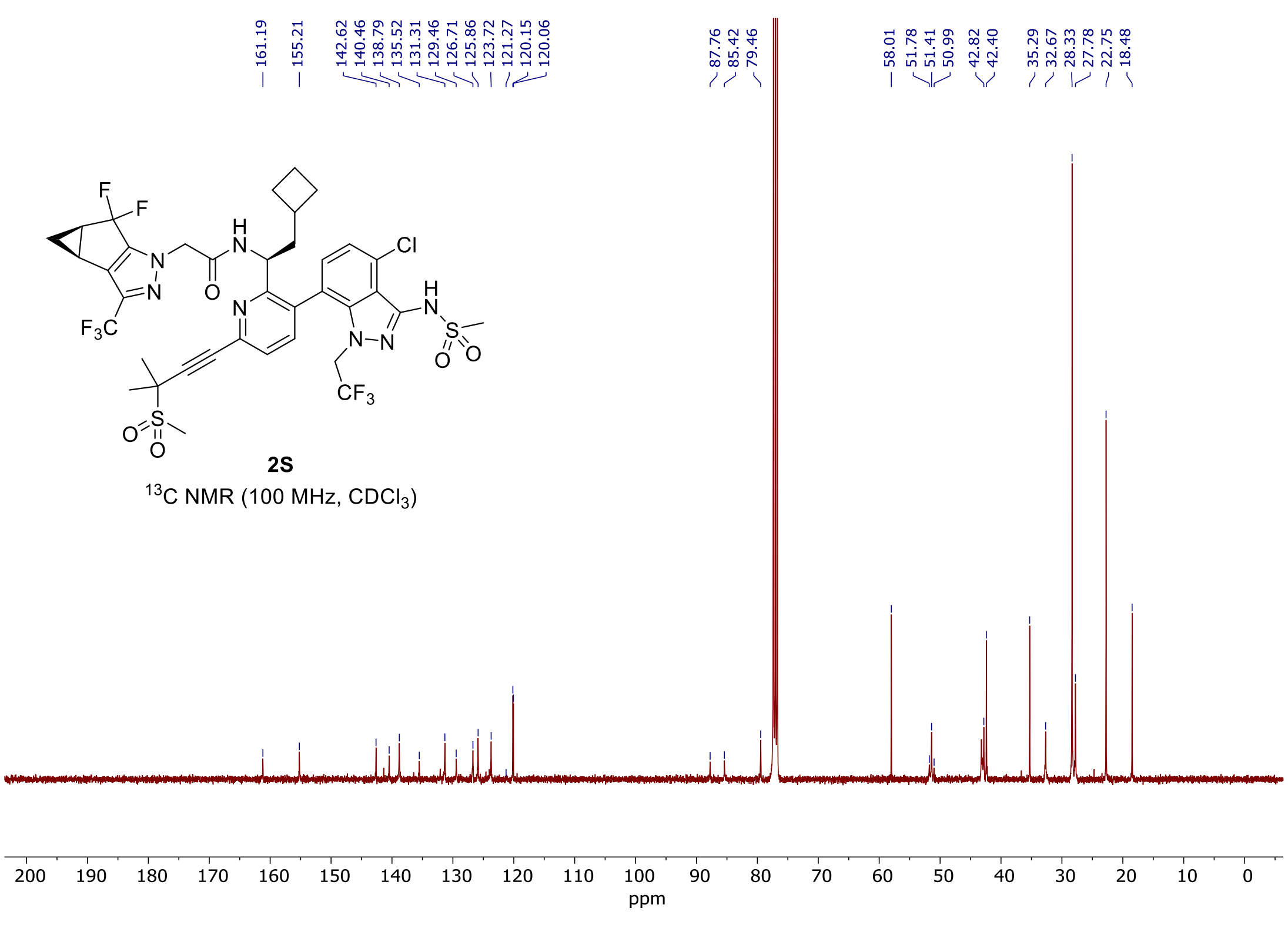

$^{19}$F NMR (376 MHz, $CDCl_3$) of compound **2S**

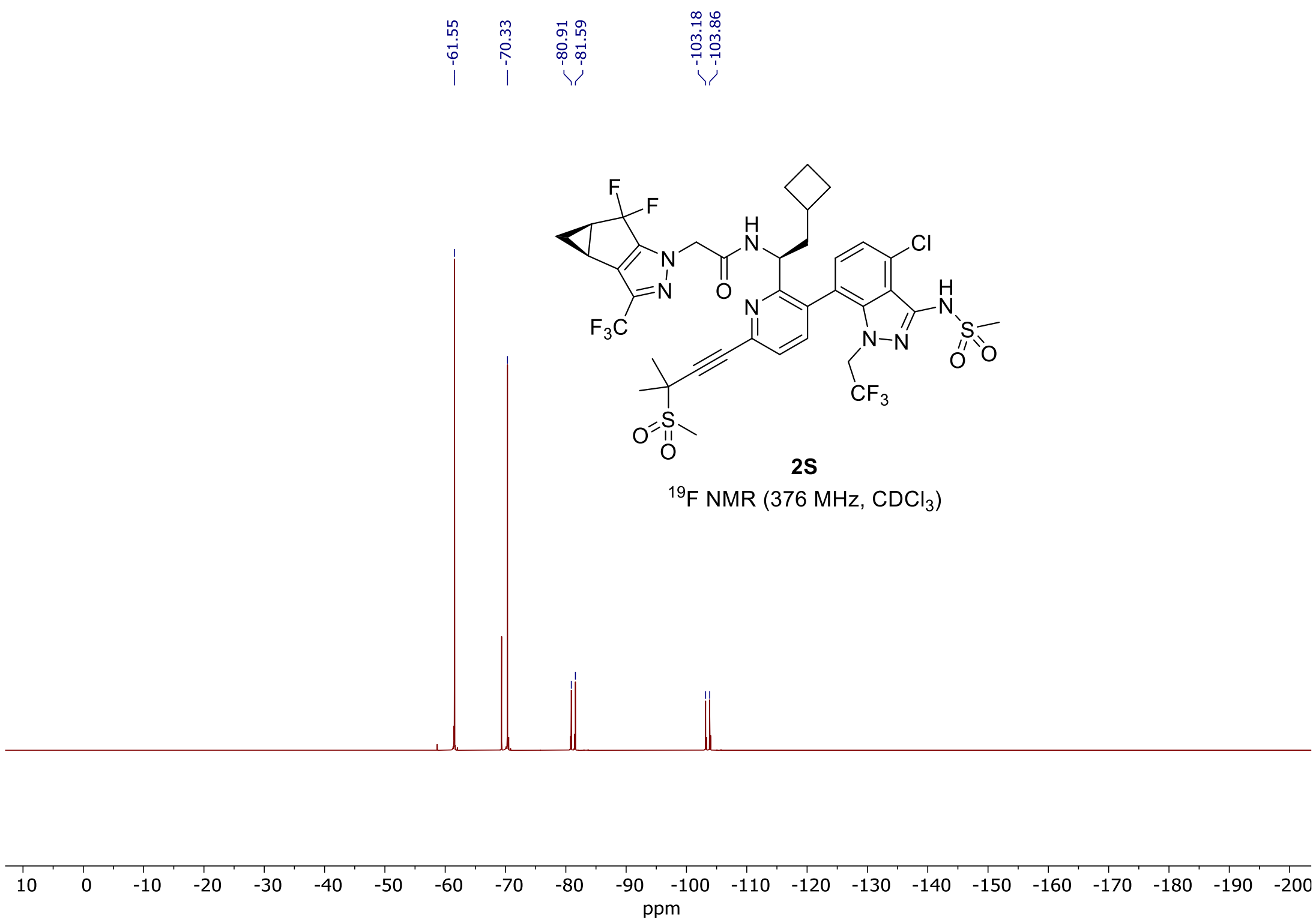


HPLC data of compound **2S**

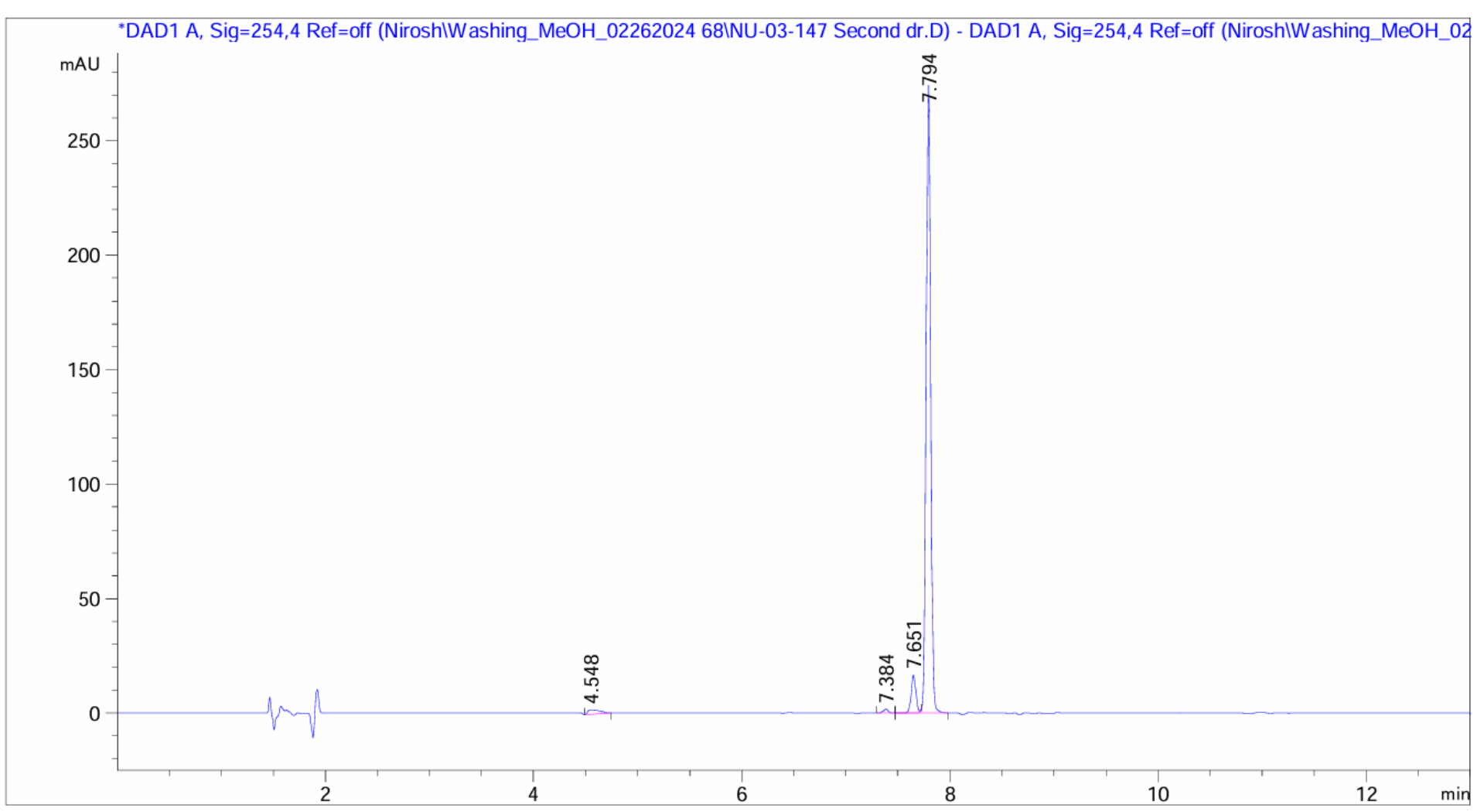


| Peak # | RetTime [min] | Type | Width [min] | Area [mAU*s] | Height [mAU] | Area % | |
|---|---|---|---|---|---|---|---|
| 1 | 4.548 | BB | 0.1321 | 16.63982 | 2.06195 | 1.8255 | |
| 2 | 7.384 | BB | 0.0520 | 5.91825 | 1.72791 | 0.6493 | |
| 3 | 7.651 | BV E | 0.0486 | 52.20947 | 16.64256 | 5.7277 | (atropisomer) |
| 4 | 7.794 | VB R | 0.0496 | 836.75977 | 274.09189 | 91.7976 | (atropisomer) |

Totals : 911.52731 294.52432

$^{1}H$ NMR (400 MHz, $CDCl_3$) of the compound **2R**

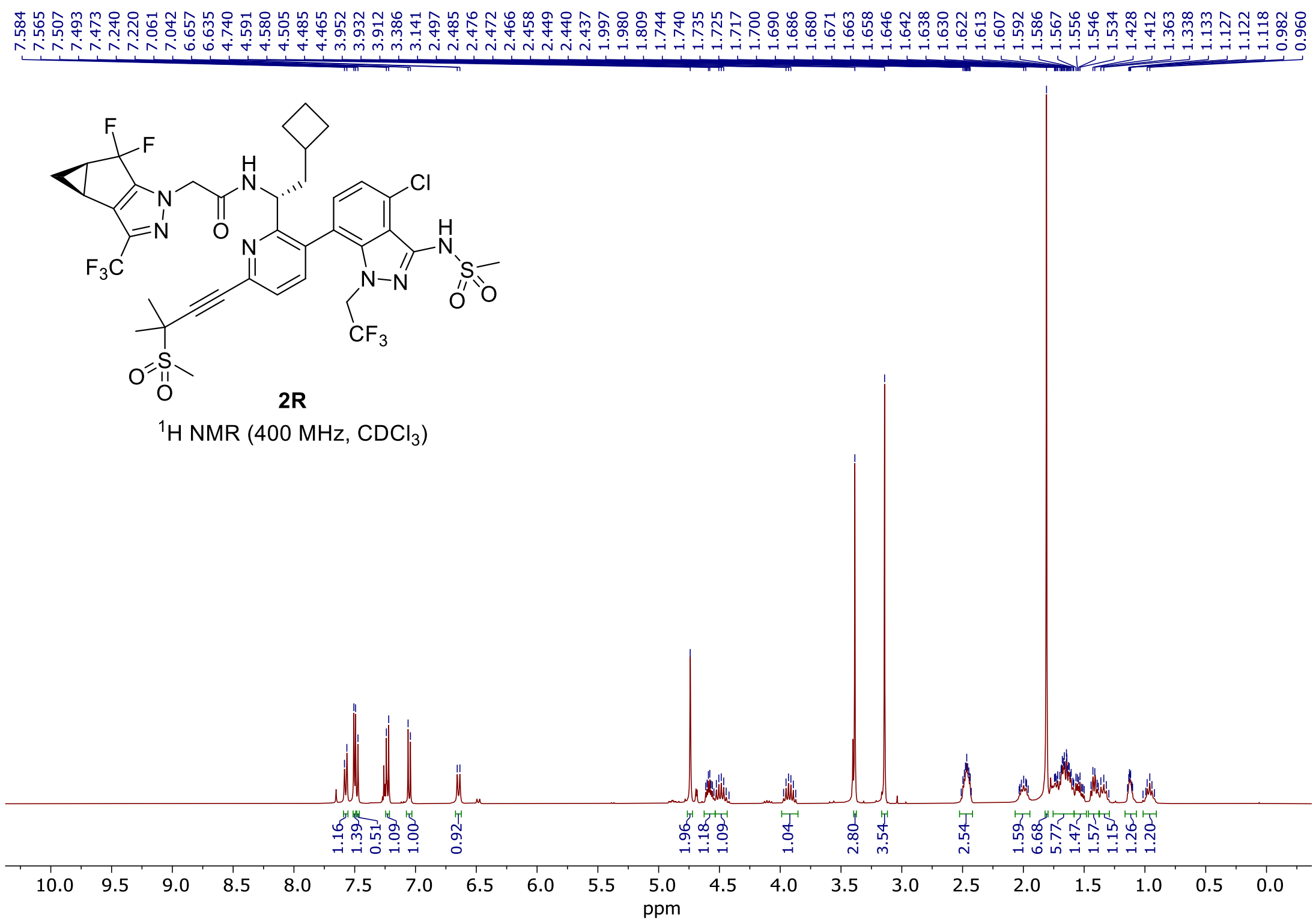


$^{13}C$ NMR (100 MHz, $CDCl_3$) of compound **2R**

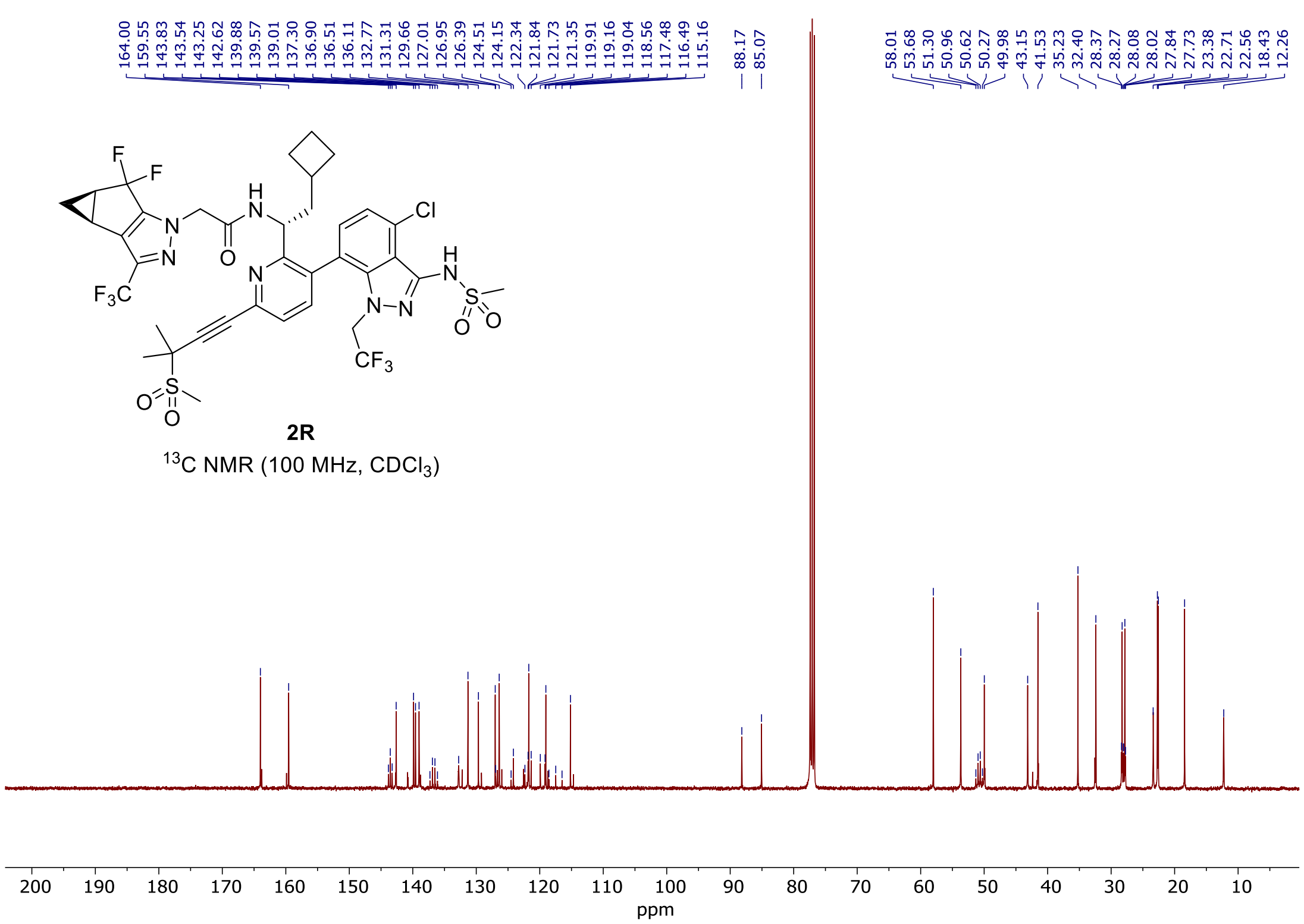

$^{19}$F NMR (376 MHz, $CDCl_3$) of compound **2R**

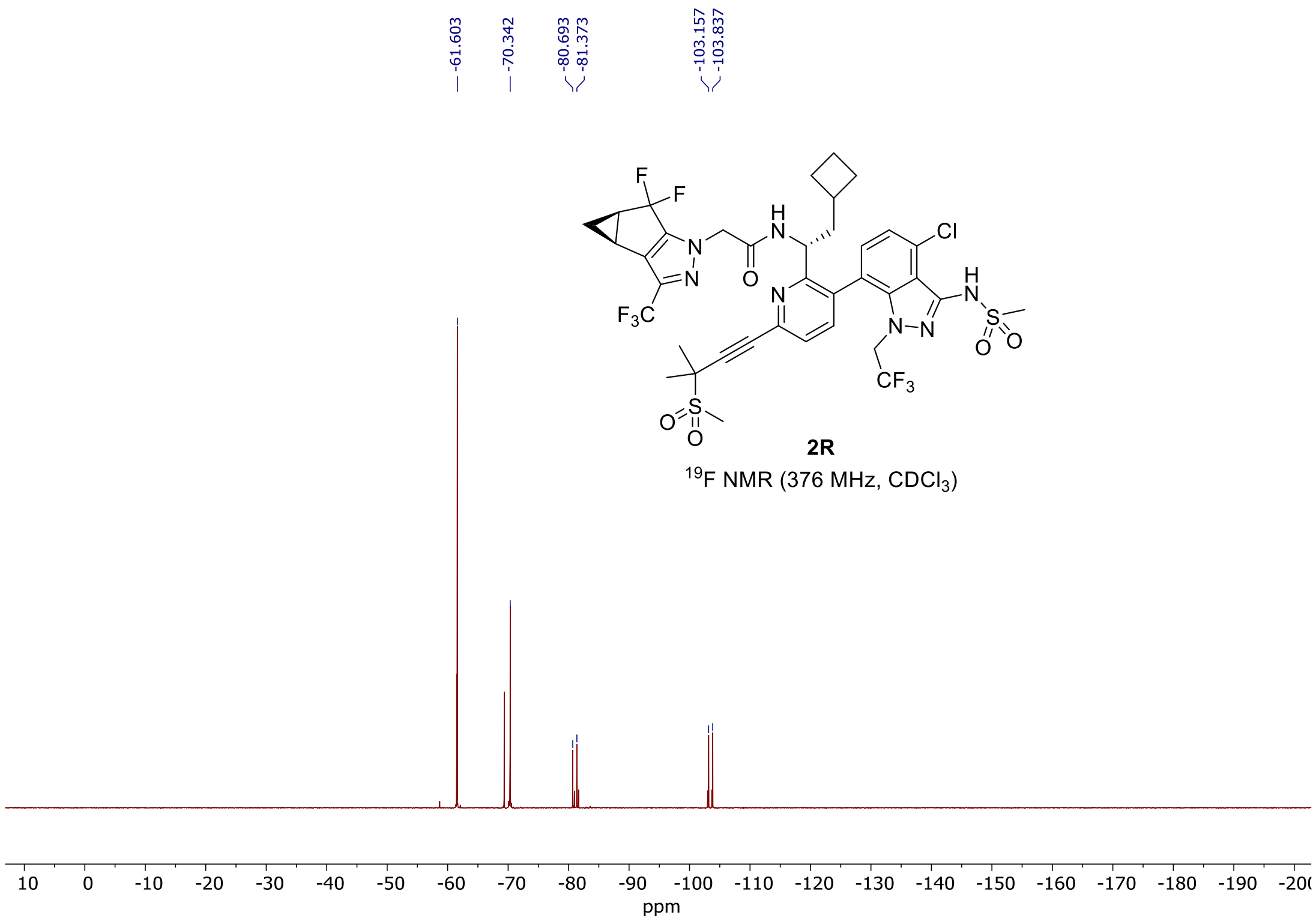


HPLC data of compound **2R**

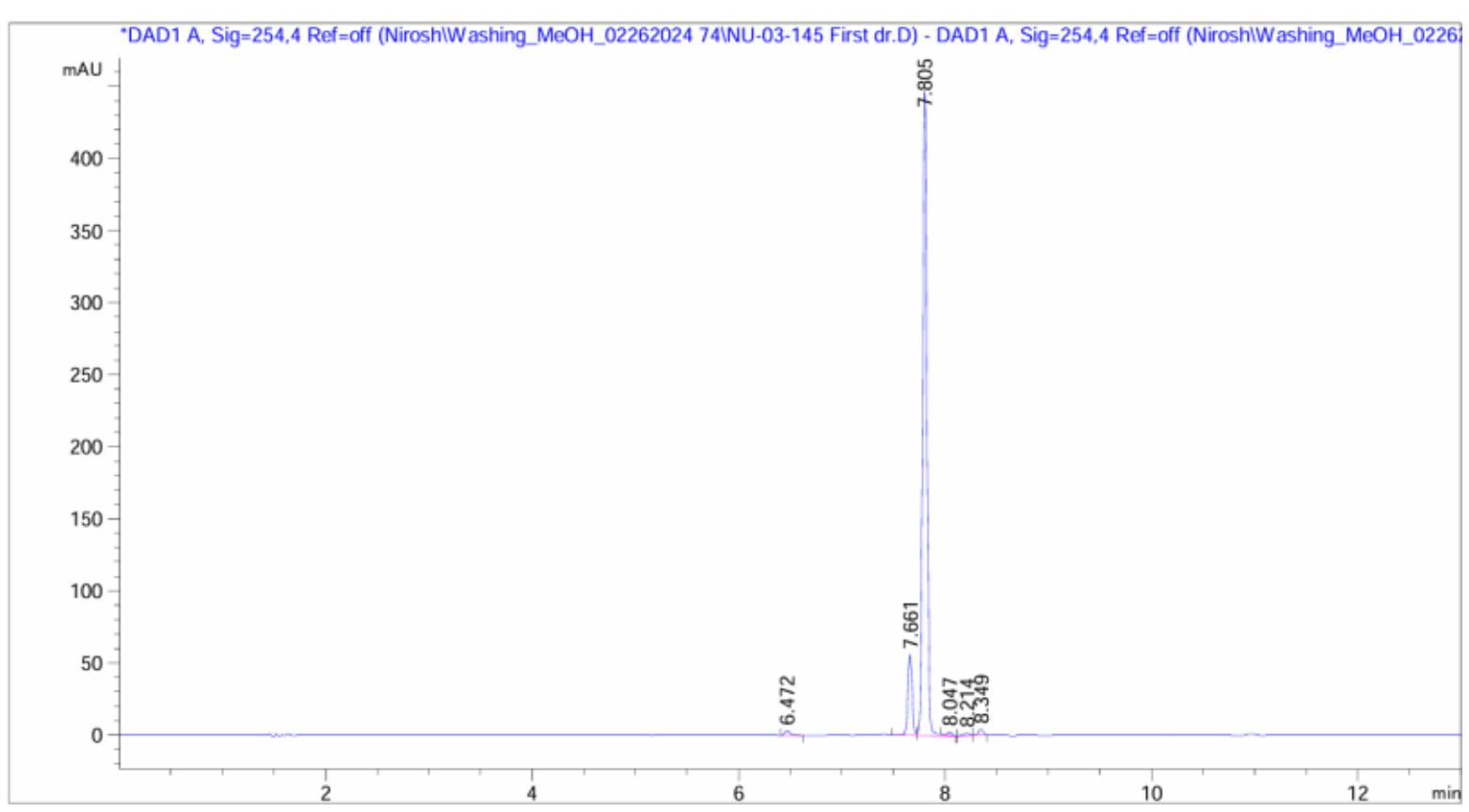


| Peak # | RetTime [min] | Type | Width [min] | Area [mAU*s] | Height [mAU] | Area % | |
|---|---|---|---|---|---|---|---|
| 1 | 6.472 | BB | 0.0589 | 12.15000 | 3.30309 | 0.8078 | |
| 2 | 7.661 | BV | 0.0457 | 161.82933 | 55.96597 | 10.7596 | (atropisomer) |
| 3 | 7.805 | VV R | 0.0456 | 1301.96899 | 451.65540 | 86.5648 | (atropisomer) |
| 4 | 8.047 | VB E | 0.0503 | 8.02291 | 2.44110 | 0.5334 | |
| 5 | 8.214 | BV | 0.0631 | 7.40945 | 1.69197 | 0.4926 | |
| 6 | 8.349 | VB | 0.0496 | 12.65904 | 4.15149 | 0.8417 | |
| Totals : | | | | 1504.03973 | 519.20900 | | |

$^{1}$H NMR (400 MHz, $CDCl_3$) of compound **3S**

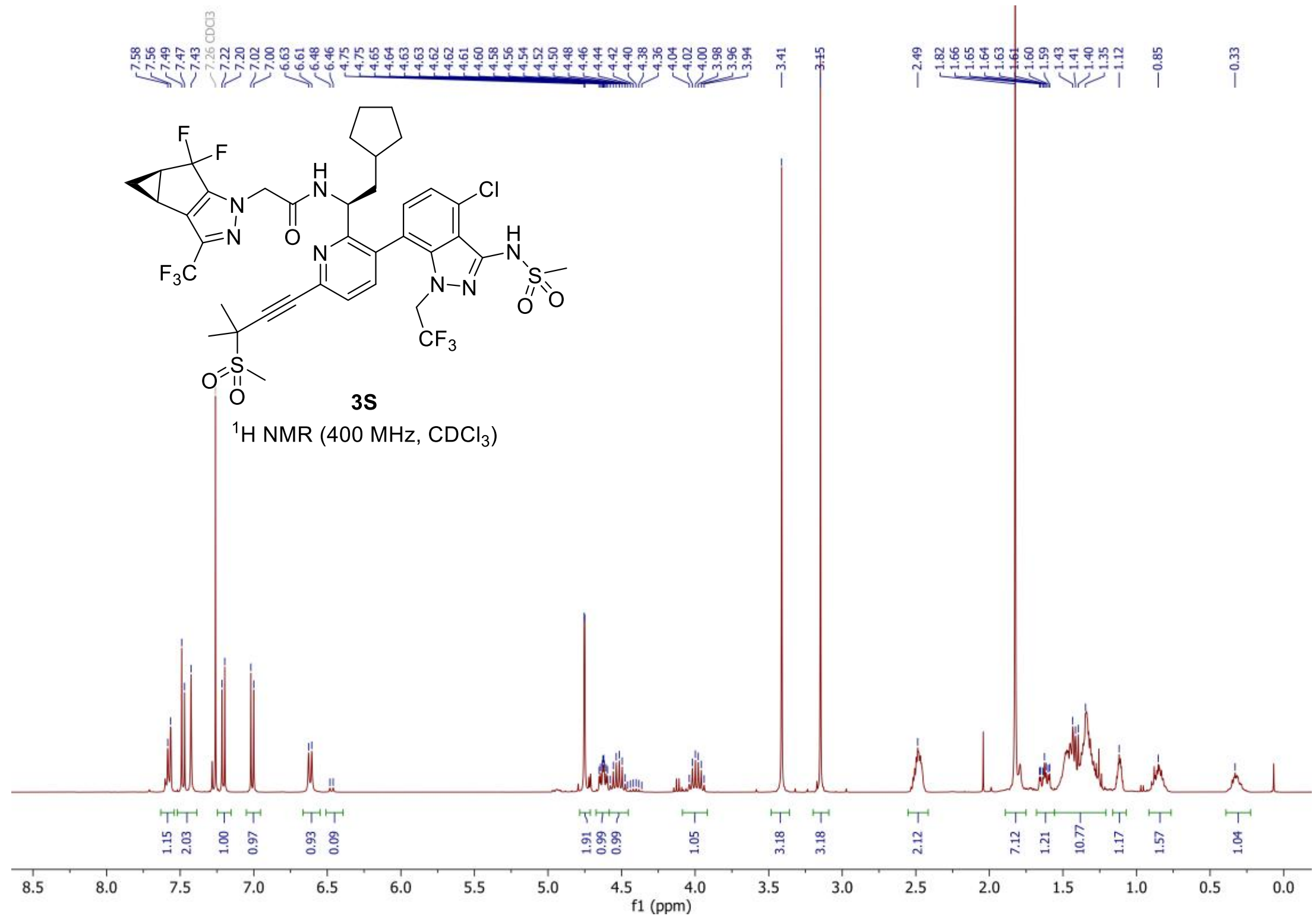

$^{13}$C NMR (100 MHz, $CDCl_3$) of compound **3S**

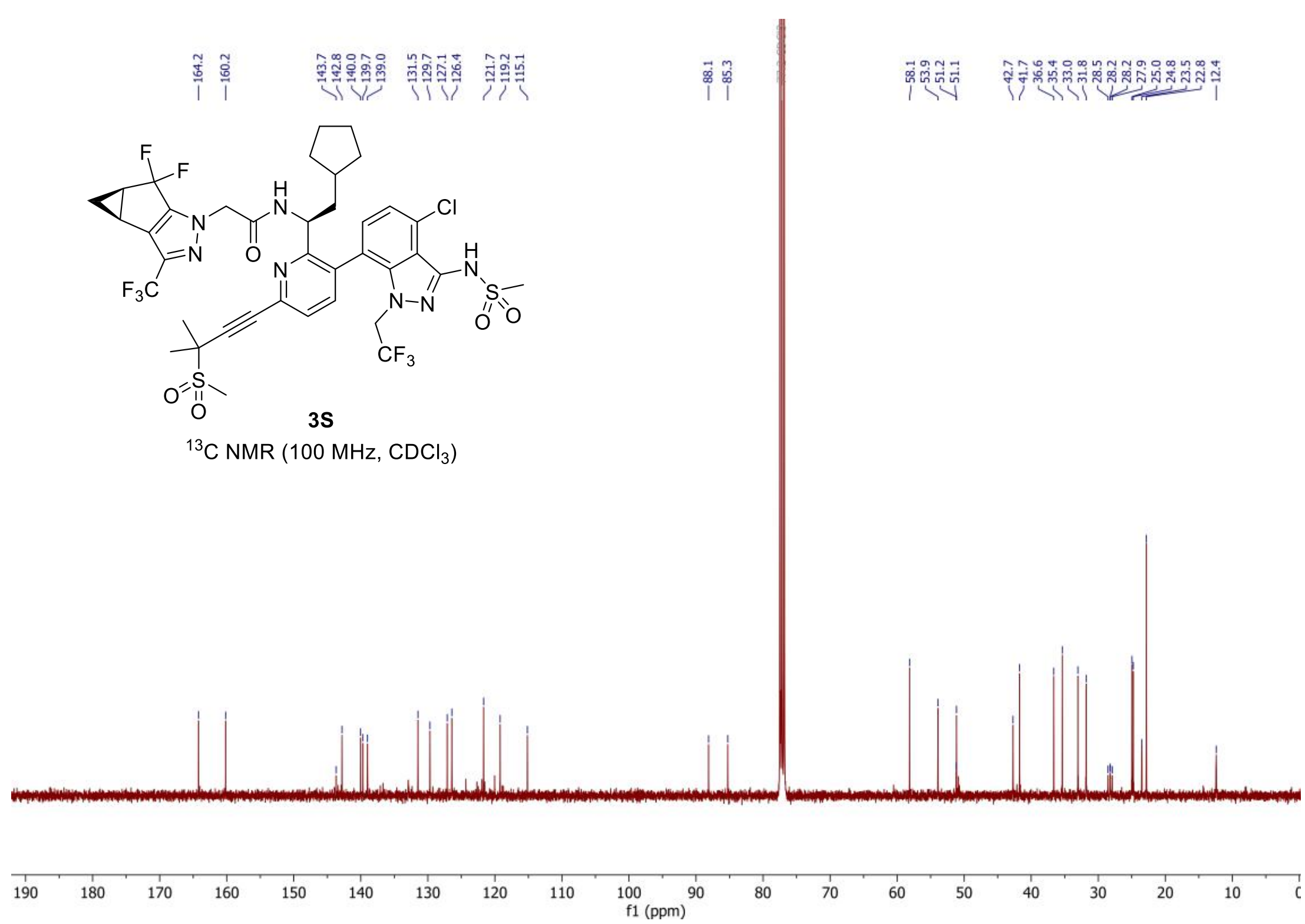

COSY NMR (400 MHz, $CDCl_3$) of compound **3S**

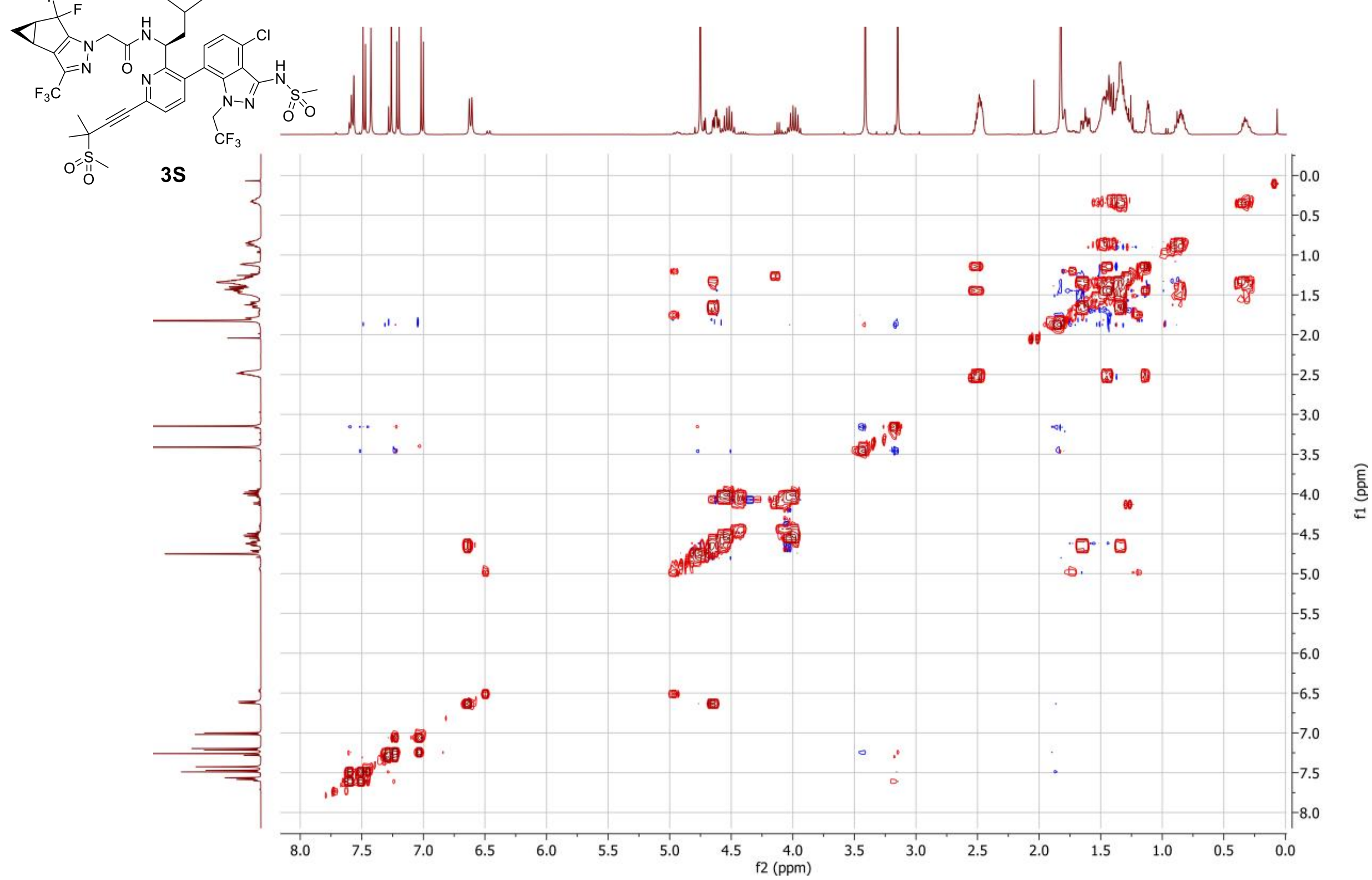


NOESY NMR (400 MHz, $CDCl_3$) of compound **3S**

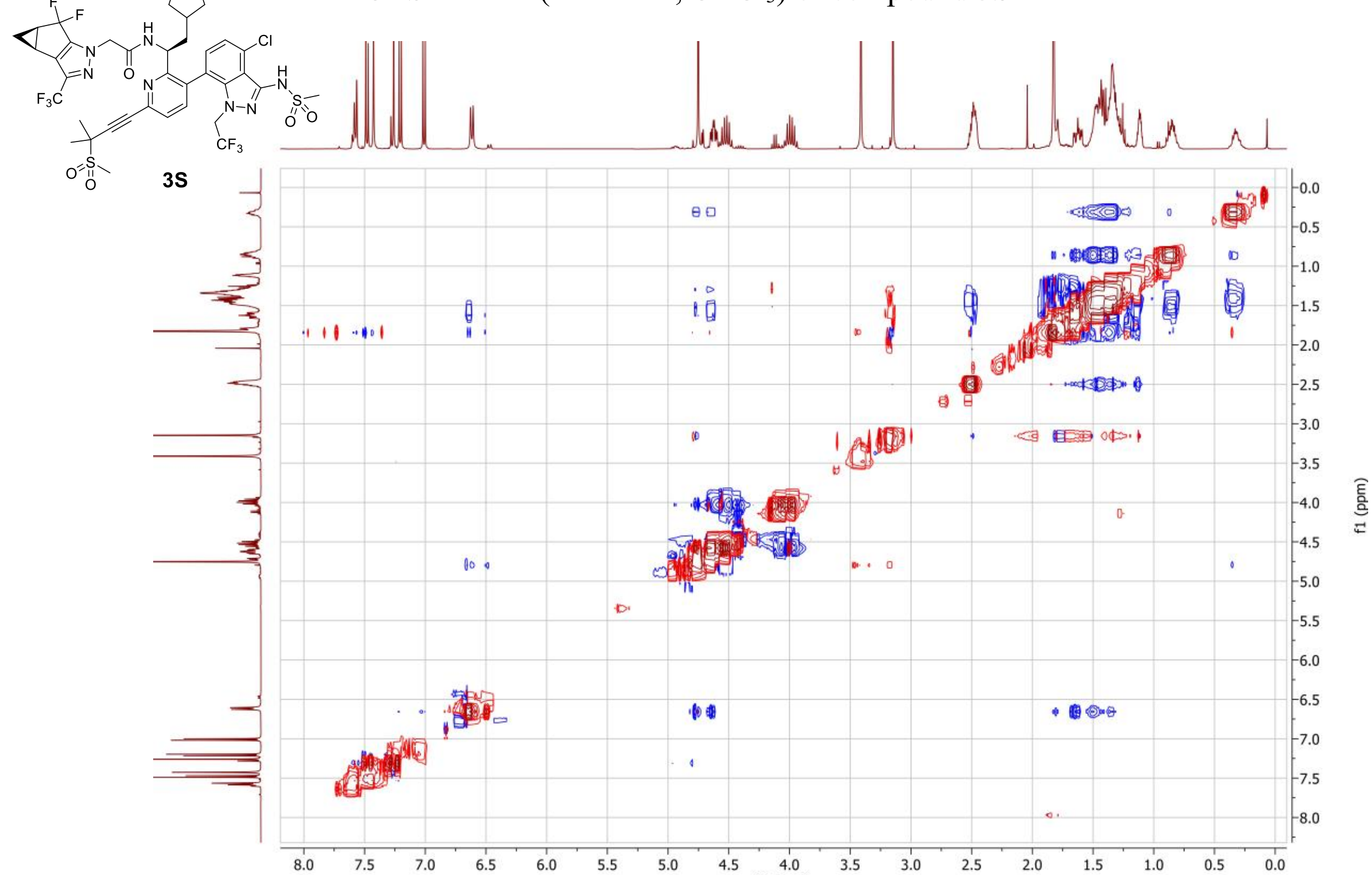

$^{19}$F NMR (376 MHz, $CDCl_3$) of compound **3S**

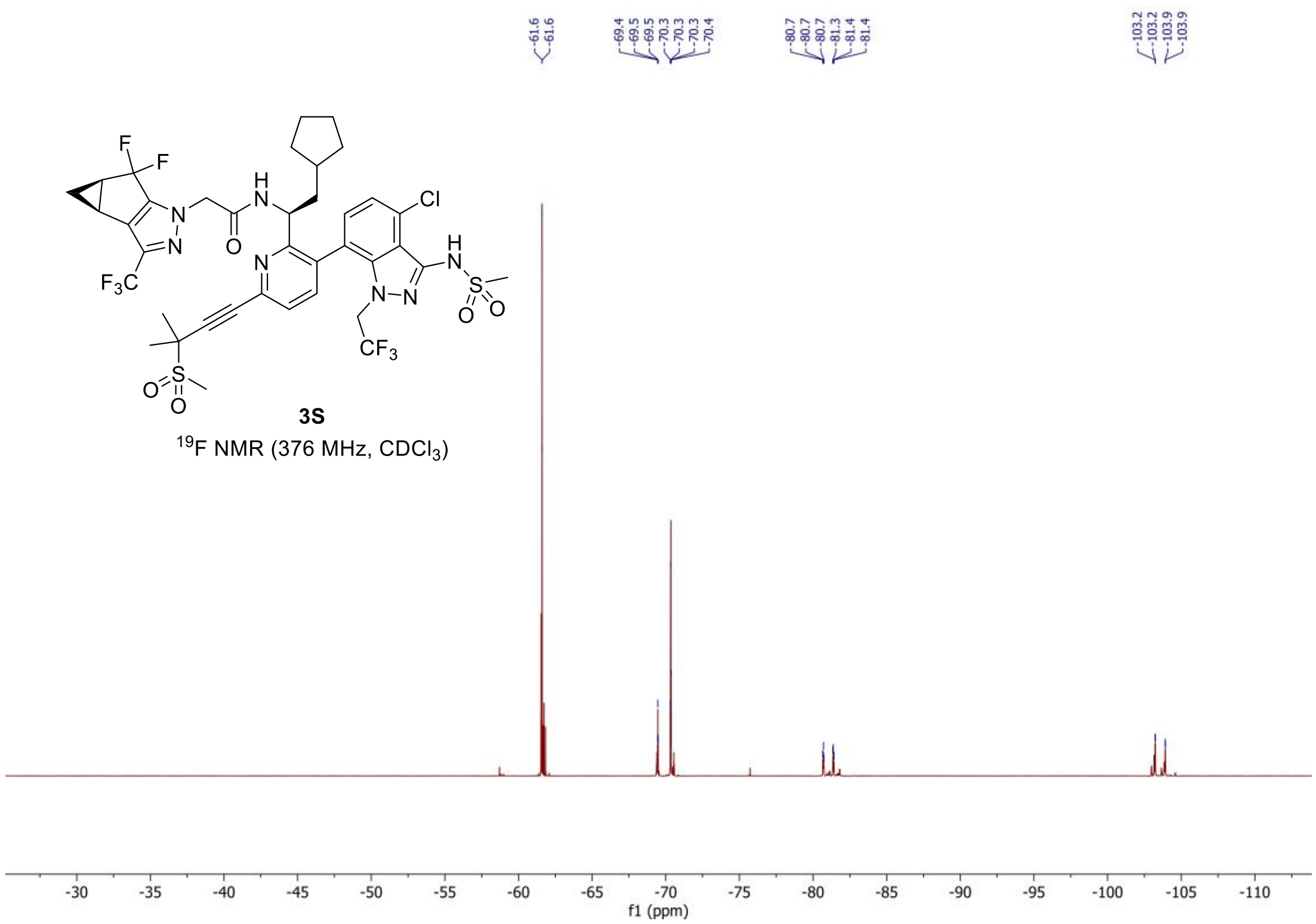


HPLC data of compound **3S**

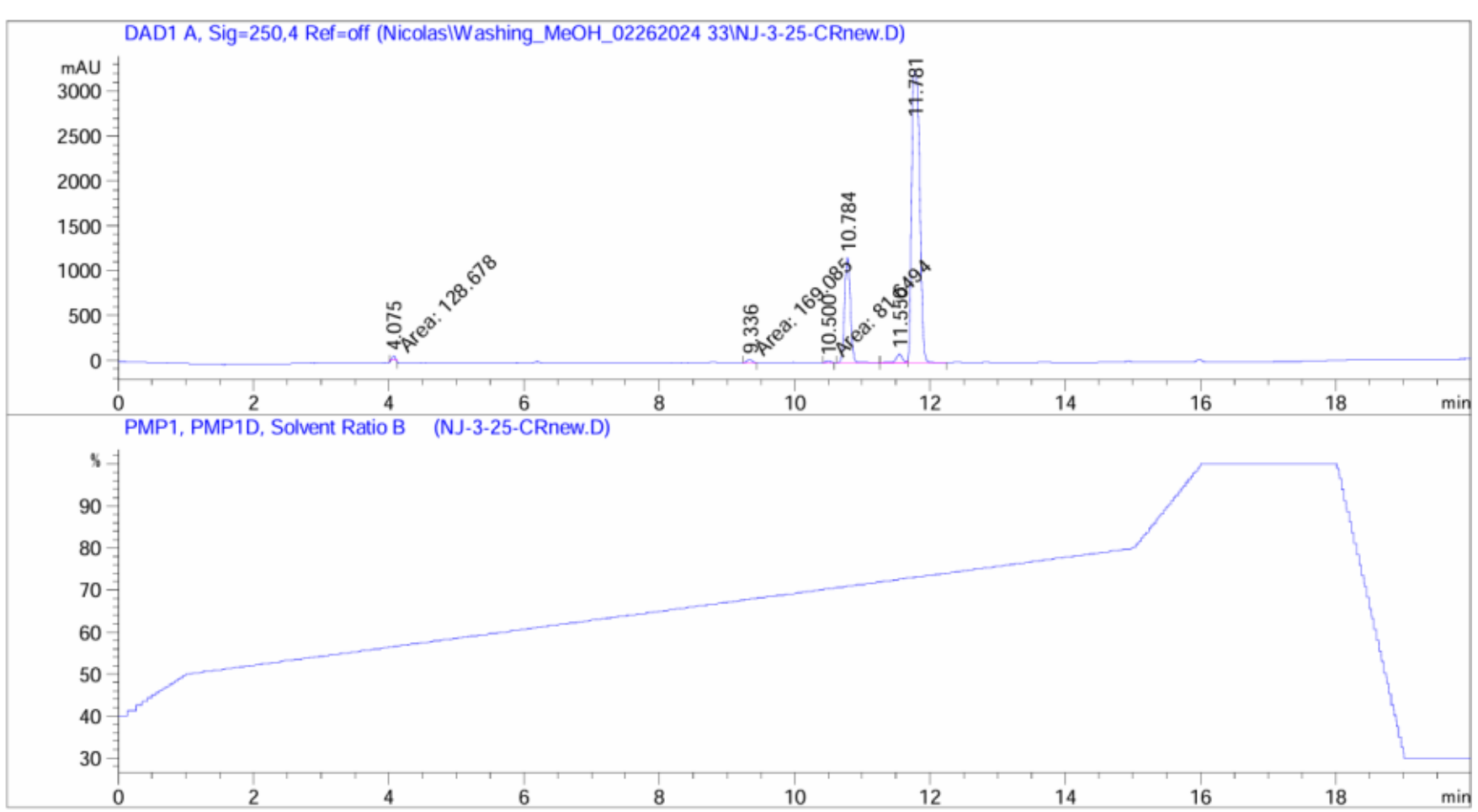


| Peak # | RetTime [min] | Type | Width [min] | Area [mAU*s] | Height [mAU] | Area % | |
|---|---|---|---|---|---|---|---|
| 1 | 4.075 | MM | 0.0460 | 128.67812 | 46.65988 | 0.3709 | |
| 2 | 9.336 | MM | 0.0867 | 169.08549 | 32.49770 | 0.4874 | |
| 3 | 10.500 | MM | 0.0792 | 81.64939 | 17.18834 | 0.2354 | |
| 4 | 10.784 | BV | 0.0915 | 6965.52441 | 1169.27368 | 20.0782 | (atropisomer) |
| 5 | 11.550 | VV E | 0.0974 | 626.56482 | 96.92489 | 1.8061 | |
| 6 | 11.781 | VB R | 0.1336 | 2.67205e4 | 3260.34692 | 77.0221 | (atropisomer) |

Totals : 3.46920e4 4622.89141

$^{1}H$ NMR (400 MHz, $CDCl_3$) of compound **3R**

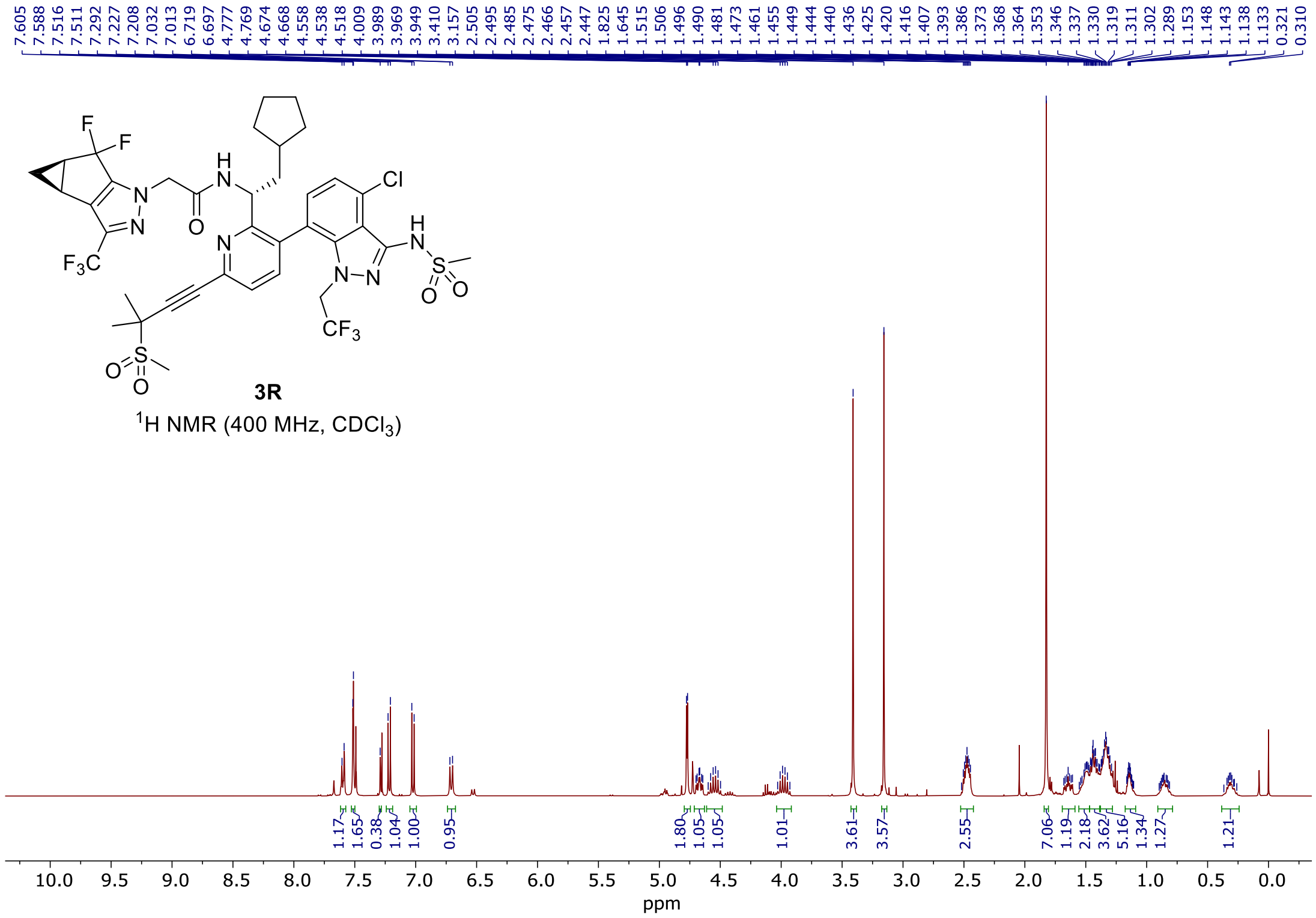


$^{13}C$ NMR (100 MHz, $CDCl_3$) of compound **3R**

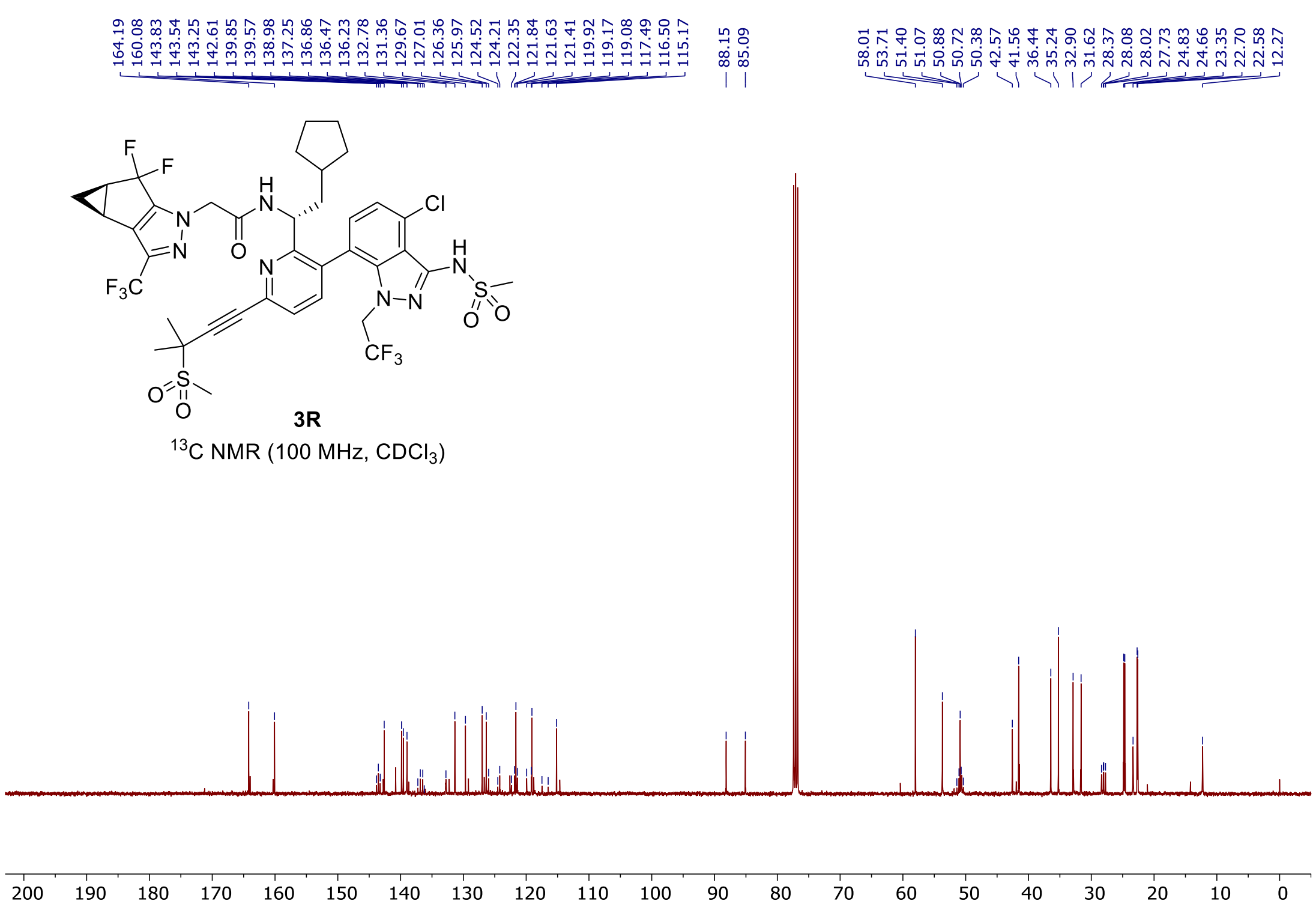

$^{19}$F NMR (376 MHz, $CDCl_3$) of compound **3R**

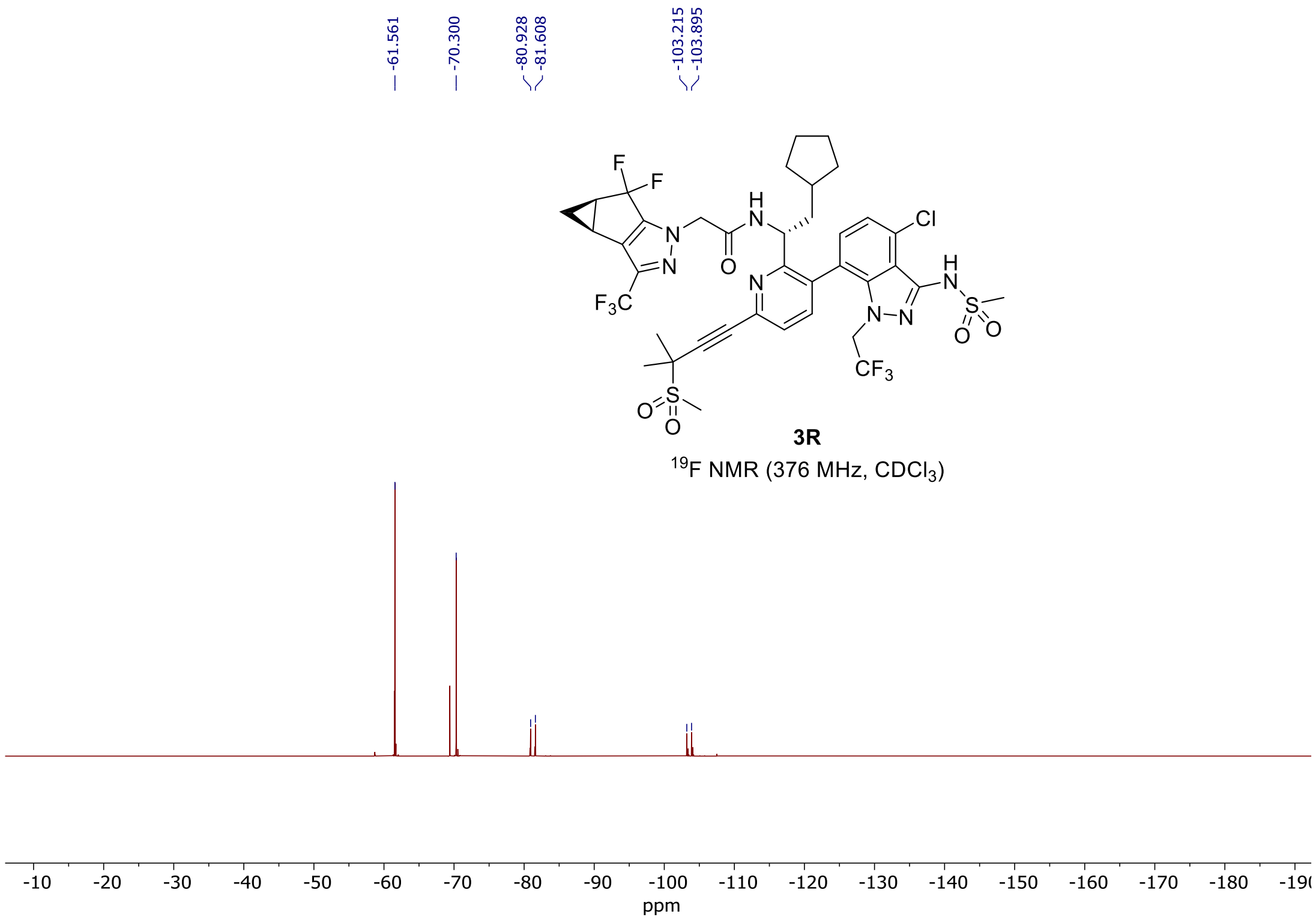


HPLC data of compound **3R**

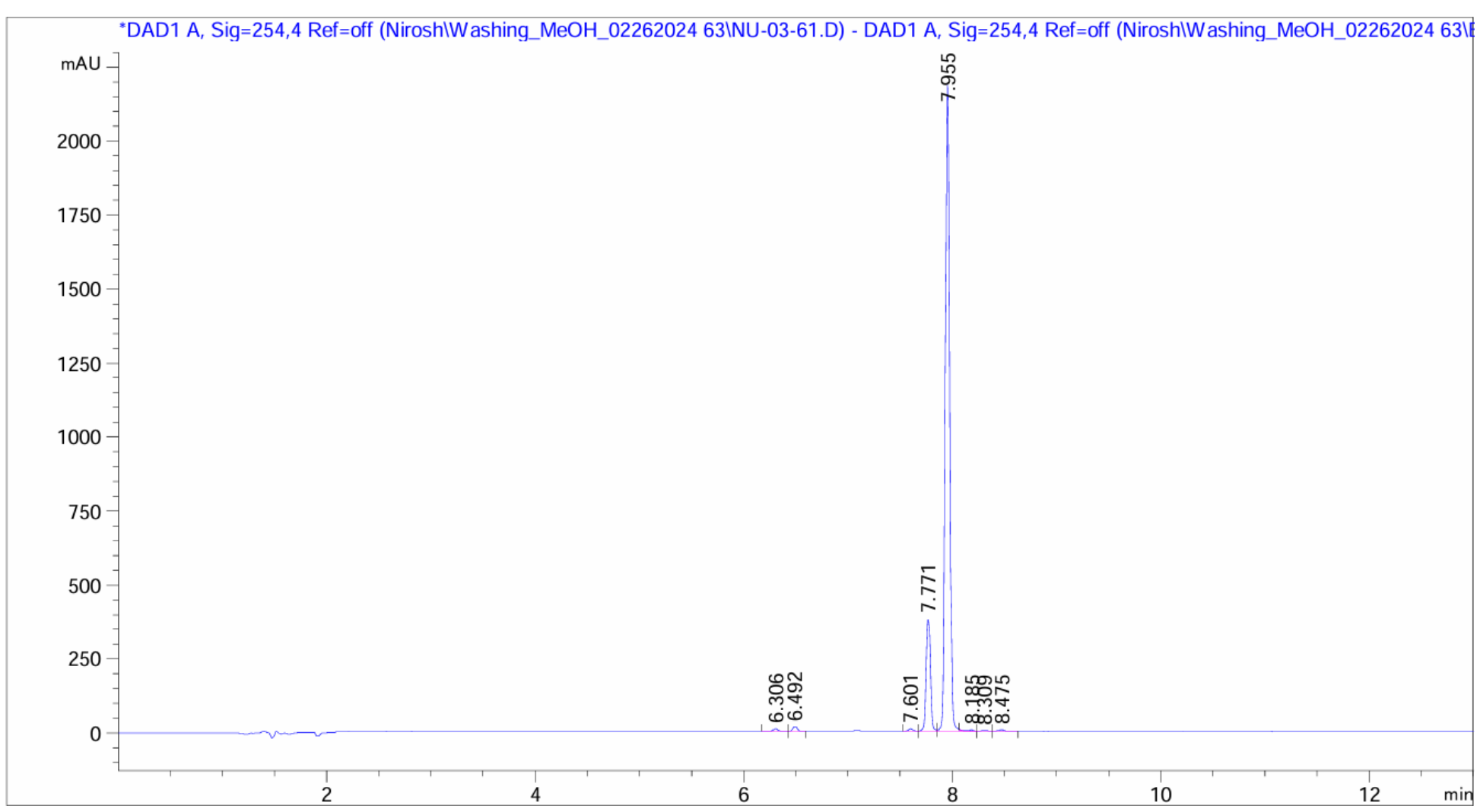


| Peak # | RetTime [min] | Type | Width [min] | Area [mAU*s] | Height [mAU] | Area % | |
|---|---|---|---|---|---|---|---|
| 1 | 6.306 | VB R | 0.0502 | 25.74482 | 7.86423 | 0.3373 | |
| 2 | 6.492 | BB | 0.0463 | 45.61037 | 15.49321 | 0.5977 | |
| 3 | 7.601 | BB | 0.0481 | 22.64483 | 7.75857 | 0.2967 | |
| 4 | 7.771 | BV | 0.0452 | 1083.33789 | 380.99332 | 14.1955 | (atropisomer) |
| 5 | 7.955 | VV R | 0.0461 | 6403.22314 | 2191.42700 | 83.9045 | (atropisomer) |
| 6 | 8.185 | VB E | 0.0585 | 18.78985 | 4.71155 | 0.2462 | |
| 7 | 8.309 | BV | 0.0729 | 13.01720 | 2.57971 | 0.1706 | |
| 8 | 8.475 | VB | 0.0640 | 19.19392 | 4.30386 | 0.2515 | |
| Totals : | | | | 7631.56202 | 2615.13145 | | |

$^{1}$H NMR (400 MHz, $CDCl_3$) of compound **4S**

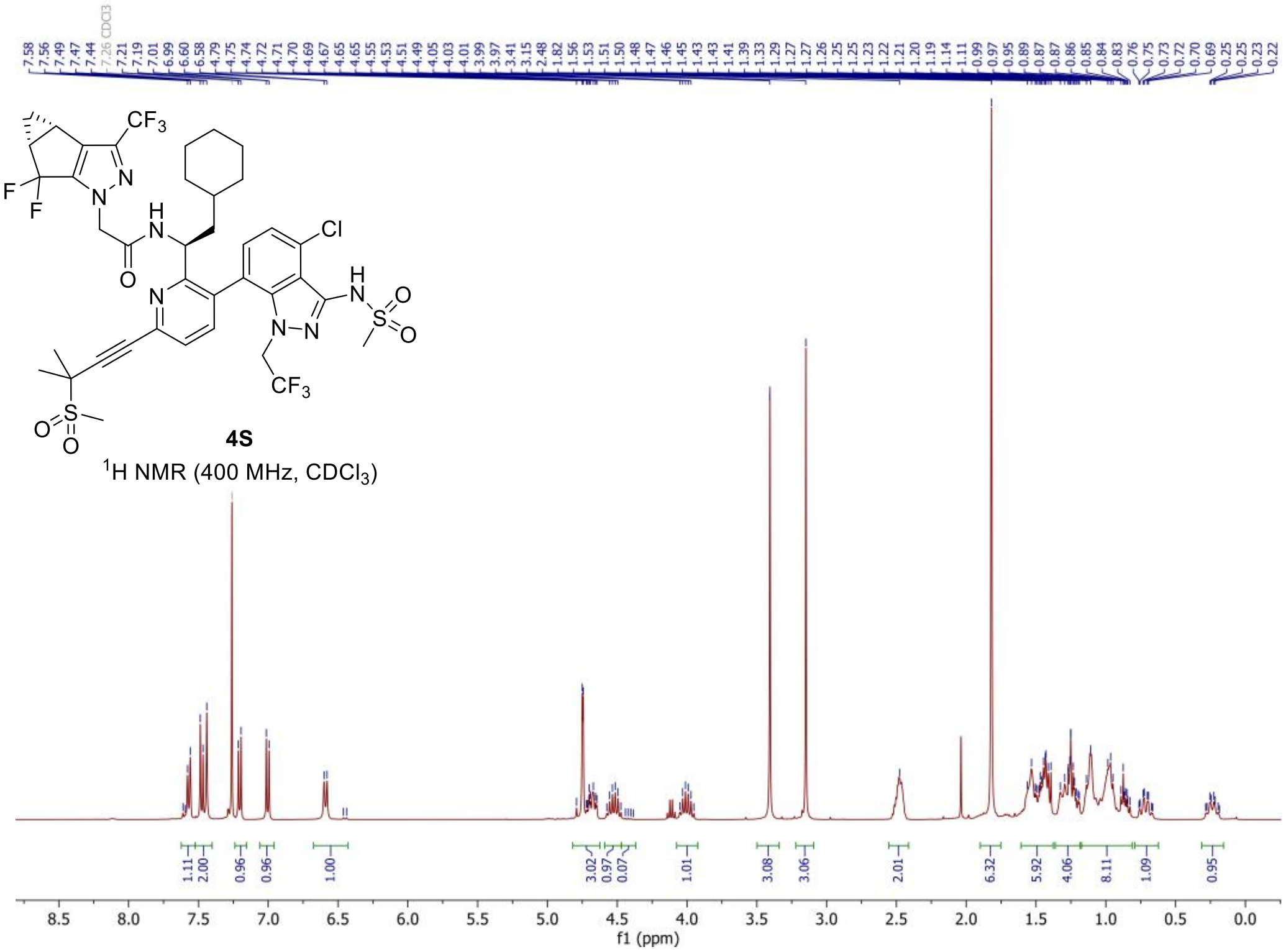


$^{13}$C NMR (100 MHz, $CDCl_3$) of compound **4S**

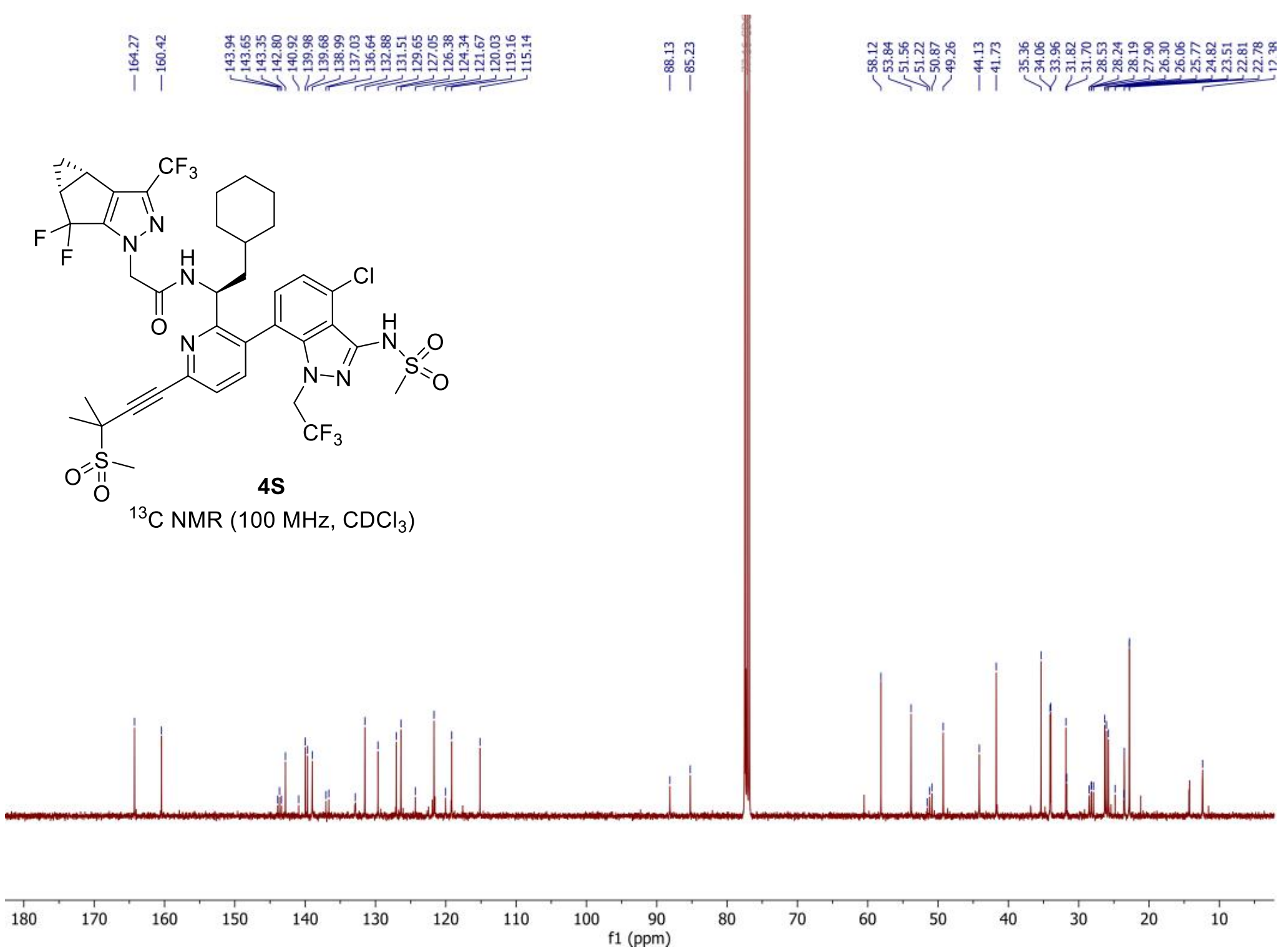

$^{19}$F NMR (376 MHz, $CDCl_3$) of compound **4S**

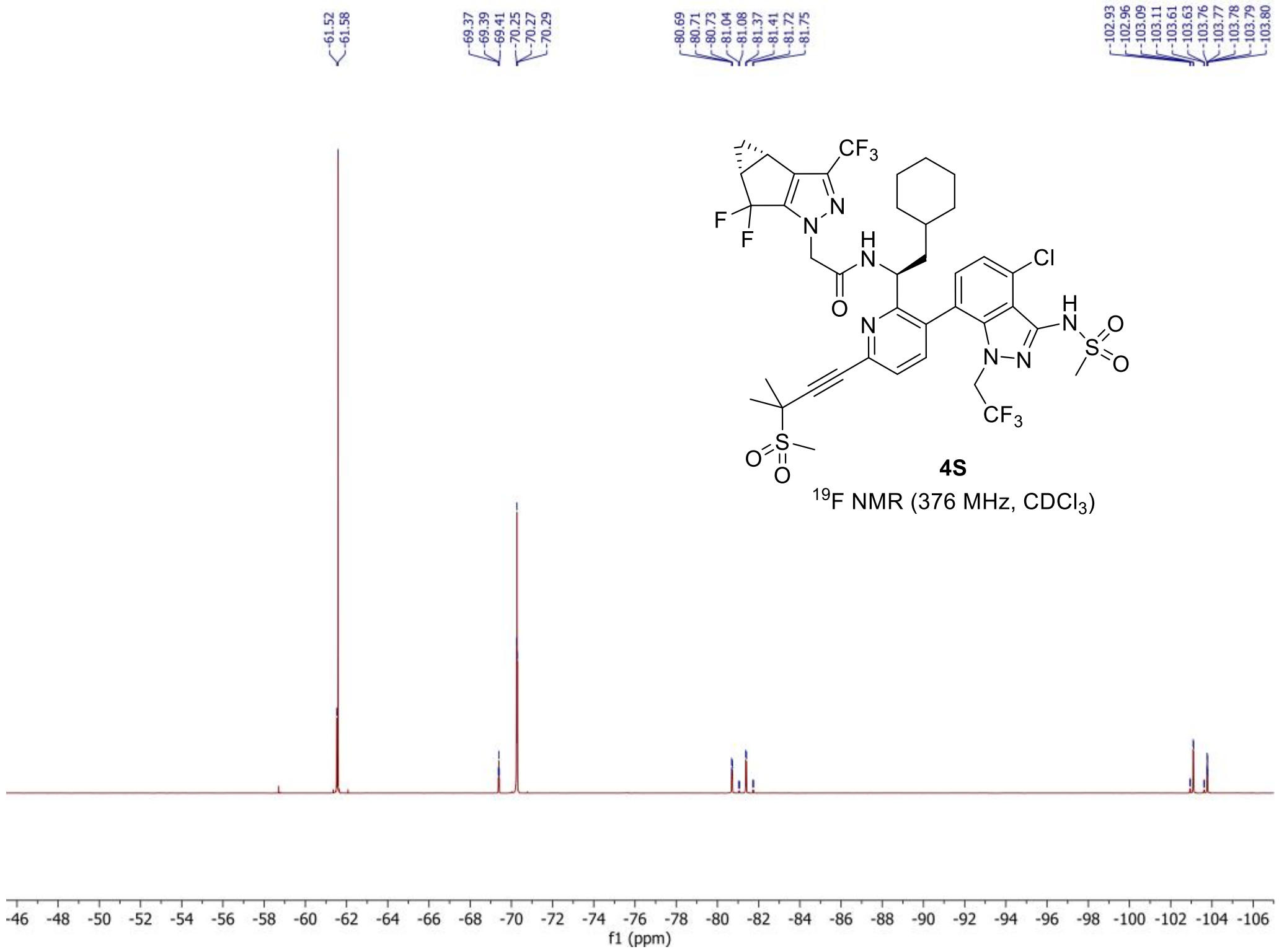


HPLC data of compound **4S**

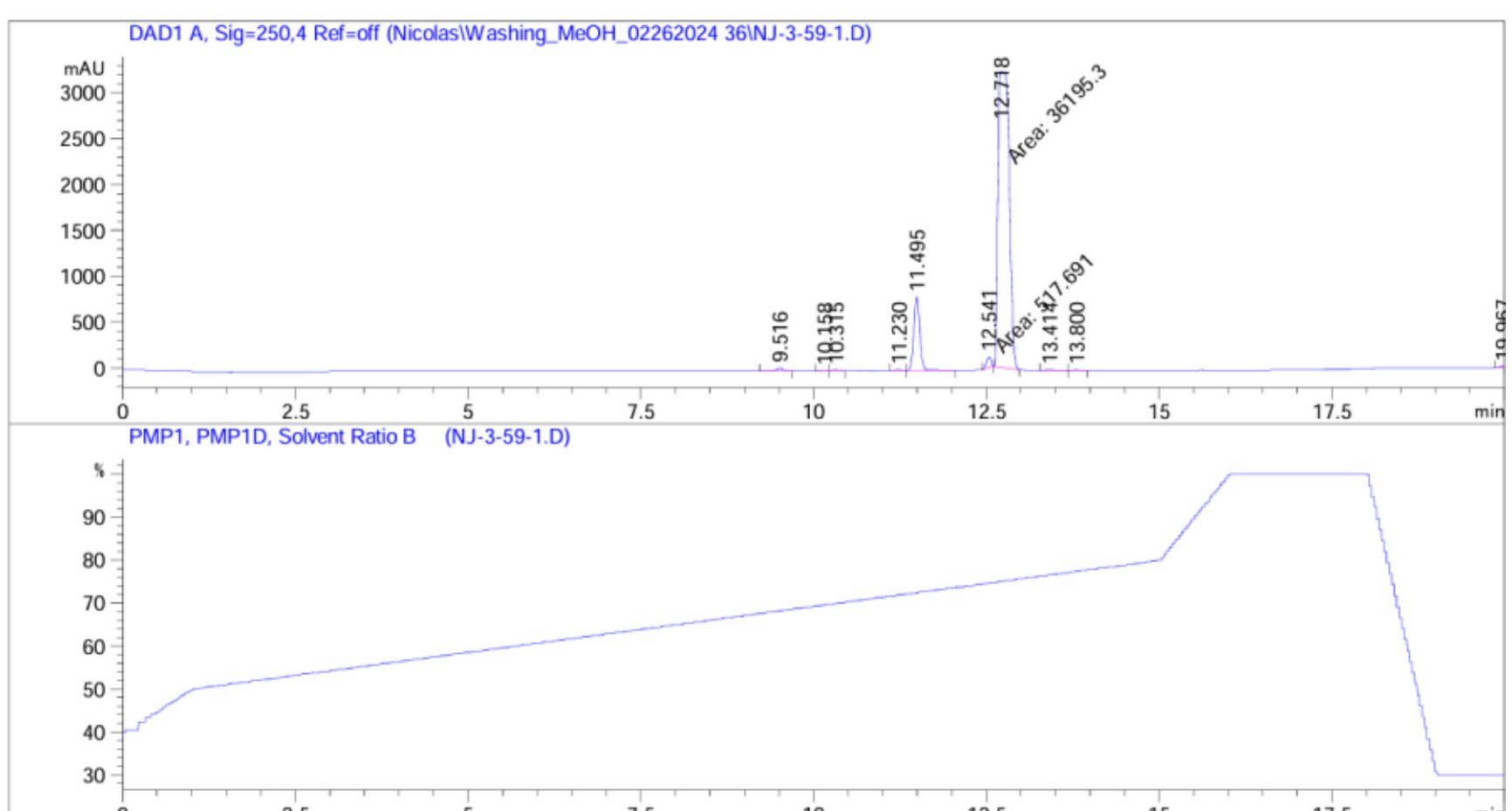


| Peak # | RetTime [min] | Type | Width [min] | Area [mAU*s] | Height [mAU] | Area % | |
|---|---|---|---|---|---|---|---|
| 1 | 9.516 | BB | 0.0981 | 193.04976 | 30.39156 | 0.4593 | |
| 2 | 10.158 | BV | 0.0903 | 23.69815 | 4.04673 | 0.0564 | |
| 3 | 10.315 | VB | 0.0922 | 61.76194 | 10.26143 | 0.1469 | |
| 4 | 11.230 | BB | 0.0876 | 68.47078 | 12.18463 | 0.1629 | |
| 5 | 11.495 | BV R | 0.0930 | 4867.83203 | 800.35339 | 11.5820 | (atropisomer) |
| 6 | 12.541 | MM | 0.0757 | 517.69128 | 113.92522 | 1.2317 | |
| 7 | 12.718 | MM | 0.1867 | 3.61953e4 | 3230.61108 | 86.1189 | (atropisomer) |
| 8 | 13.414 | BV R | 0.0999 | 54.42125 | 8.58596 | 0.1295 | |
| 9 | 13.800 | BB | 0.0907 | 25.78797 | 4.50729 | 0.0614 | |
| 10 | 19.967 | BBA | 0.0670 | 21.42544 | 5.33326 | 0.0510 | |
| Totals : | | | | 4.20294e4 | 4220.20056 | | |

$^{1}$H NMR (400 MHz, $CDCl_3$) of compound **4R**

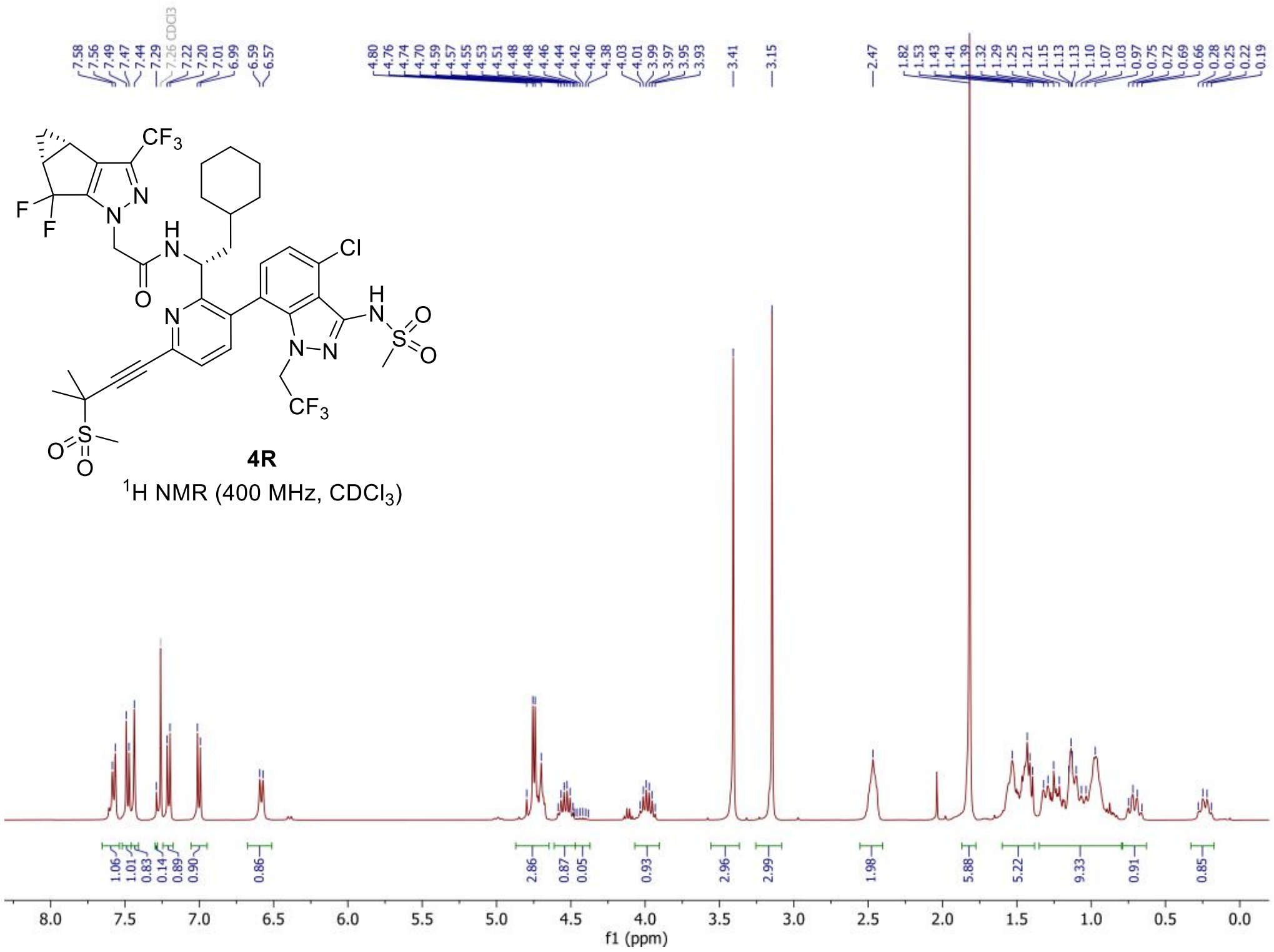

$^{13}$C NMR (100 MHz, $CDCl_3$) of compound **4R**

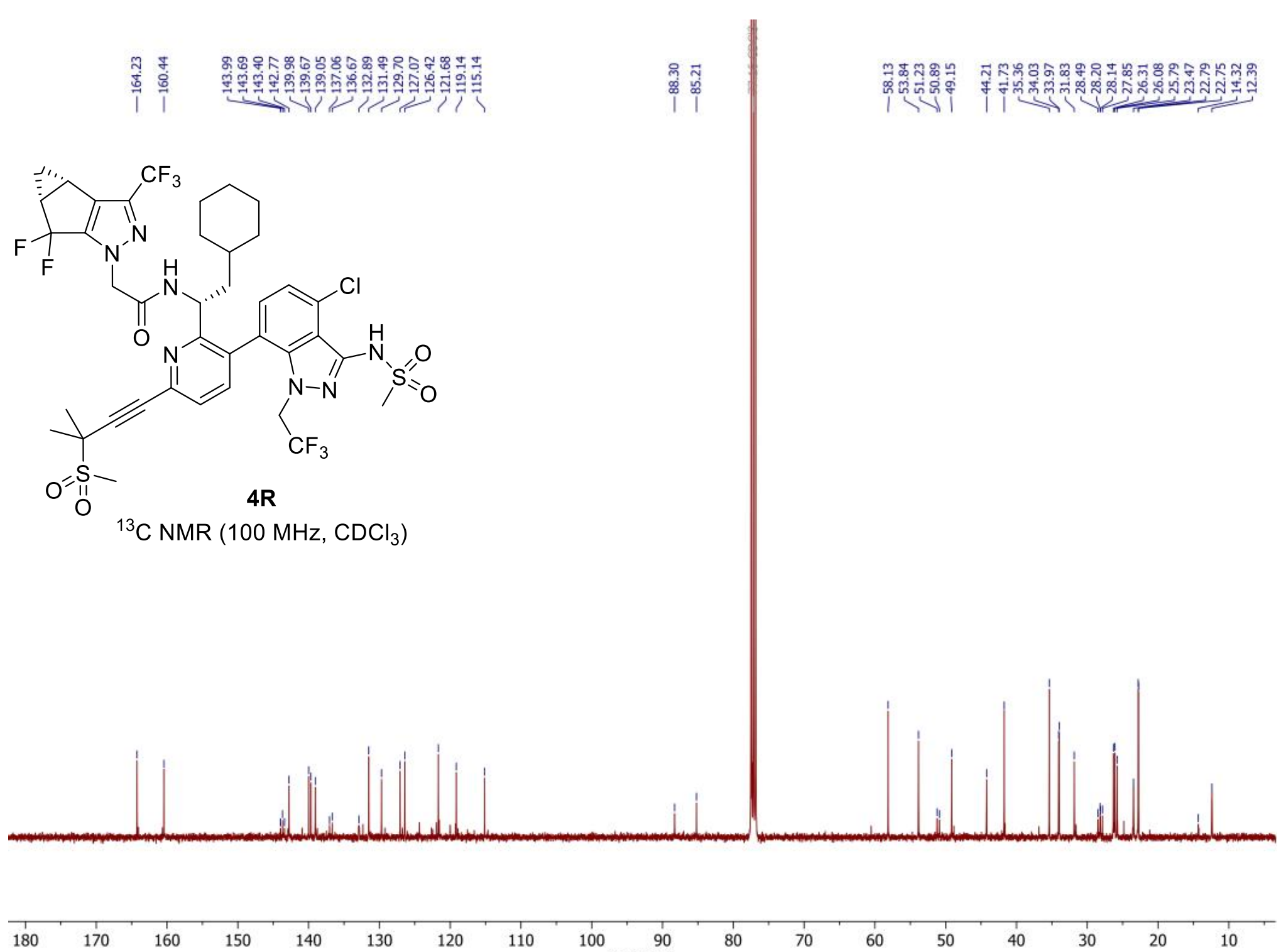

## $^{19}$F NMR (376 MHz, $CDCl_3$) of compound **4R**

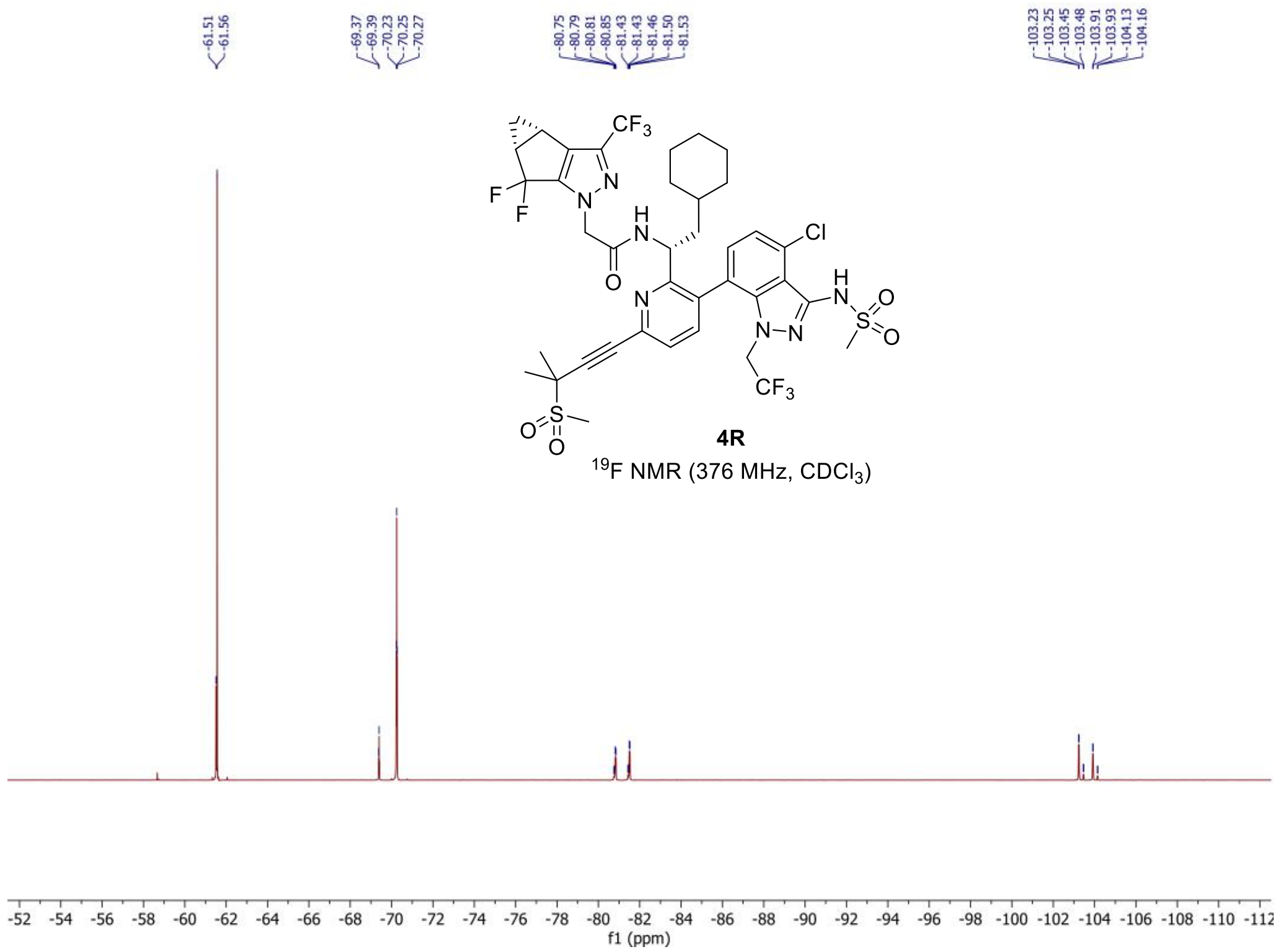


## HPLC data of compound **4R**

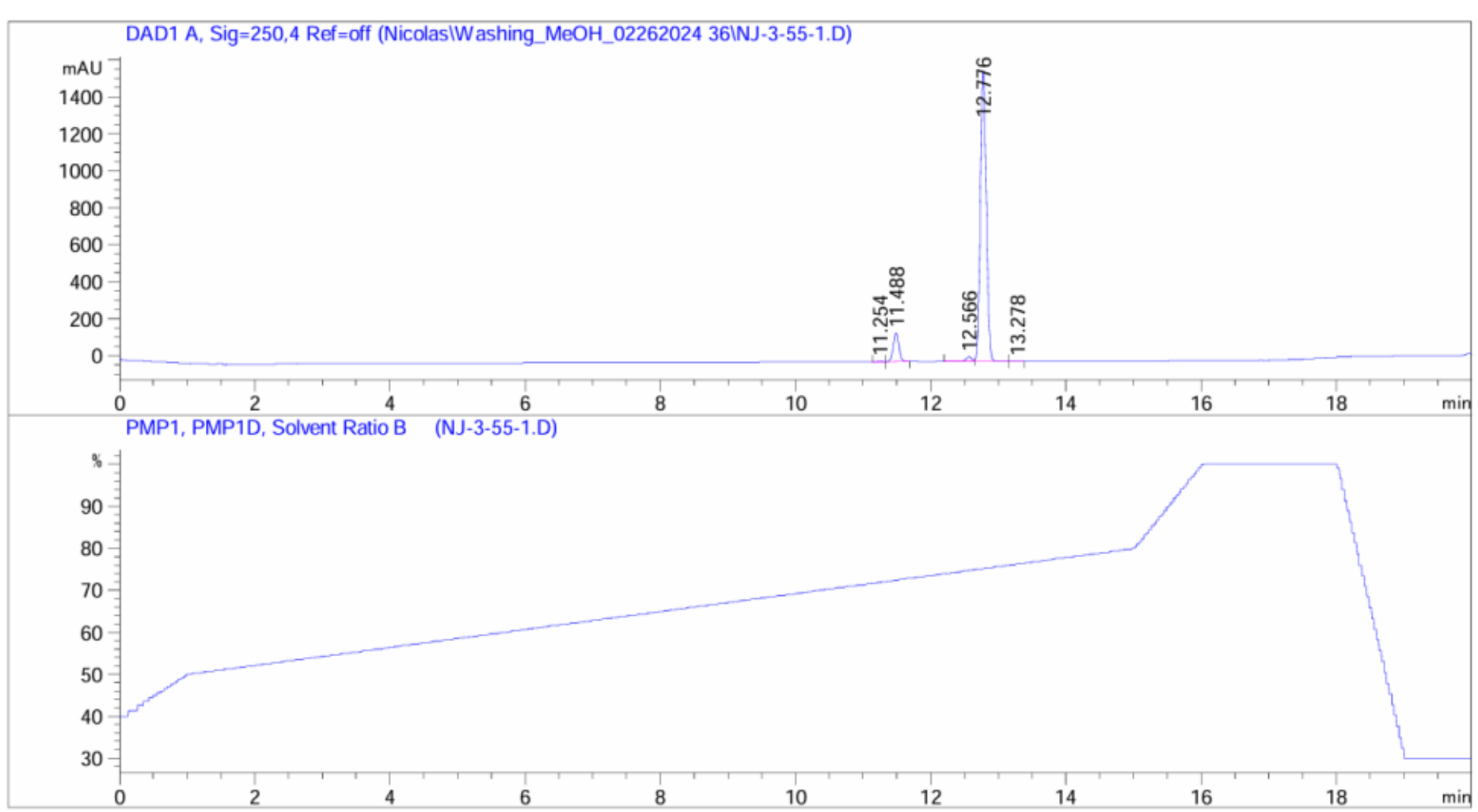


| Peak # | RetTime [min] | Type | Width [min] | Area [mAU*s] | Height [mAU] | Area % | |
|---|---|---|---|---|---|---|---|
| 1 | 11.254 | BB | 0.0862 | 13.45281 | 2.52374 | 0.1264 | |
| 2 | 11.488 | BB | 0.0932 | 918.94263 | 154.95291 | 8.6313 | (atropisomer) |
| 3 | 12.566 | BV E | 0.0896 | 151.31235 | 26.11888 | 1.4212 | |
| 4 | 12.776 | VV R | 0.0948 | 9555.91406 | 1573.86890 | 89.7552 | (atropisomer) |
| 5 | 13.278 | VB | 0.0897 | 7.02097 | 1.24679 | 0.0659 | |
| Totals : | | | | 1.06466e4 | 1758.71122 | | |
